# De-epimerizing DyKAT of β-lactones generated by isothiourea-catalysed enantioselective [2 + 2] cycloaddition†

**DOI:** 10.1039/d4sc01410c

**Published:** 2024-04-24

**Authors:** Aífe Conboy, Alister S. Goodfellow, Kevin Kasten, Joanne Dunne, David B. Cordes, Michael Bühl, Andrew D. Smith

**Affiliations:** a EaStCHEM, School of Chemistry, University of St Andrews St Andrews Fife KY16 9ST UK ads10@st-andrews.ac.uk mb105@st-andrews.ac.uk

## Abstract

An enantioselective isothiourea-catalysed [2 + 2] cycloaddition of C(1)-ammonium enolates with pyrazol-4,5-diones is used to construct spirocyclic β-lactones in good yields, excellent enantioselectivity (99 : 1 er) but with modest diastereocontrol (typically 70 : 30 dr). Upon ring-opening with morpholine or alternative nucleophilic amines and alcohols β-hydroxyamide and β-hydroxyester products are generated with enhanced diastereocontrol (up to >95 : 5 dr). Control experiments show that stereoconvergence is observed in the ring-opening of diastereoisomeric β-lactones, leading to a single product (>95 : 5 dr, >99 : 1 er). Mechanistic studies and DFT analysis indicate a substrate controlled Dynamic Kinetic Asymmetric Transformation (DyKAT) involving epimerisation at C(3) of the β-lactone under the reaction conditions, coupled with a hydrogen bond-assisted nucleophilic addition to the *Si*-face of the β-lactone and stereodetermining ring-opening. The scope and limitations of a one-pot protocol consisting of isothiourea-catalysed *enantio*-determining [2 + 2] cycloaddition followed by *diastereo*-determining ring-opening are subsequently developed. Variation within the anhydride ammonium enolate precursor, as well as N(1) and C(3) within the pyrazol-4,5-dione scaffold is demonstrated, giving a range of functionalised β-hydroxyamides with high diastereo- and enantiocontrol (>20 examples, up to >95 : 5 dr and >99 : 1 er) *via* this DyKAT.

## Abstract 2

A dynamic kinetic asymmetric transformation that couples epimerisation with a hydrogen bond-assisted nucleophilic addition and stereodetermining ring-opening is investigated.

## Graphical abstract

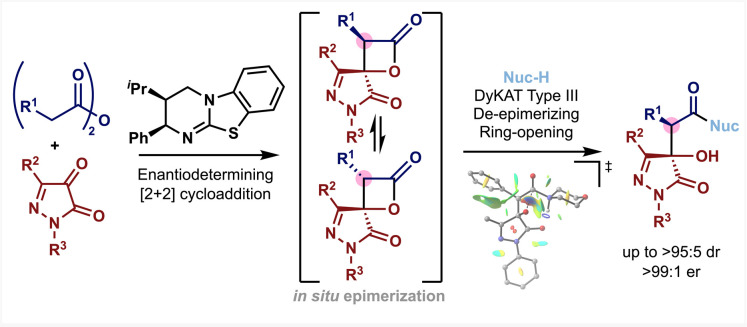

## Introduction

Asymmetric transformations are an essential tool in the synthesis of bioactive and pharmaceutically relevant compounds. Established chemical strategies towards this goal employ molecules from the natural chiral pool as starting materials, the use of chiral auxiliaries, and in particular the use of chiral reagents and catalysts.^1–12^ In most asymmetric transformations, the stereoselectivity that is observed in each reaction process is usually considered to rely upon a kinetically controlled irreversible bond-forming process within the reaction in question.^13^ In such a case, the energy differences between the diastereoisomeric transition states leading to the stereoisomeric products can be translated directly to the observed stereoisomeric product ratios. While this is commonly assumed in many asymmetric reactions, care should be taken to ensure that there is no *in situ* or post-reaction enhancement in the stereoselectivity of a given reaction process *via*, for example, selective epimerisation or crystallisation.^14^

**Fig. 1:**
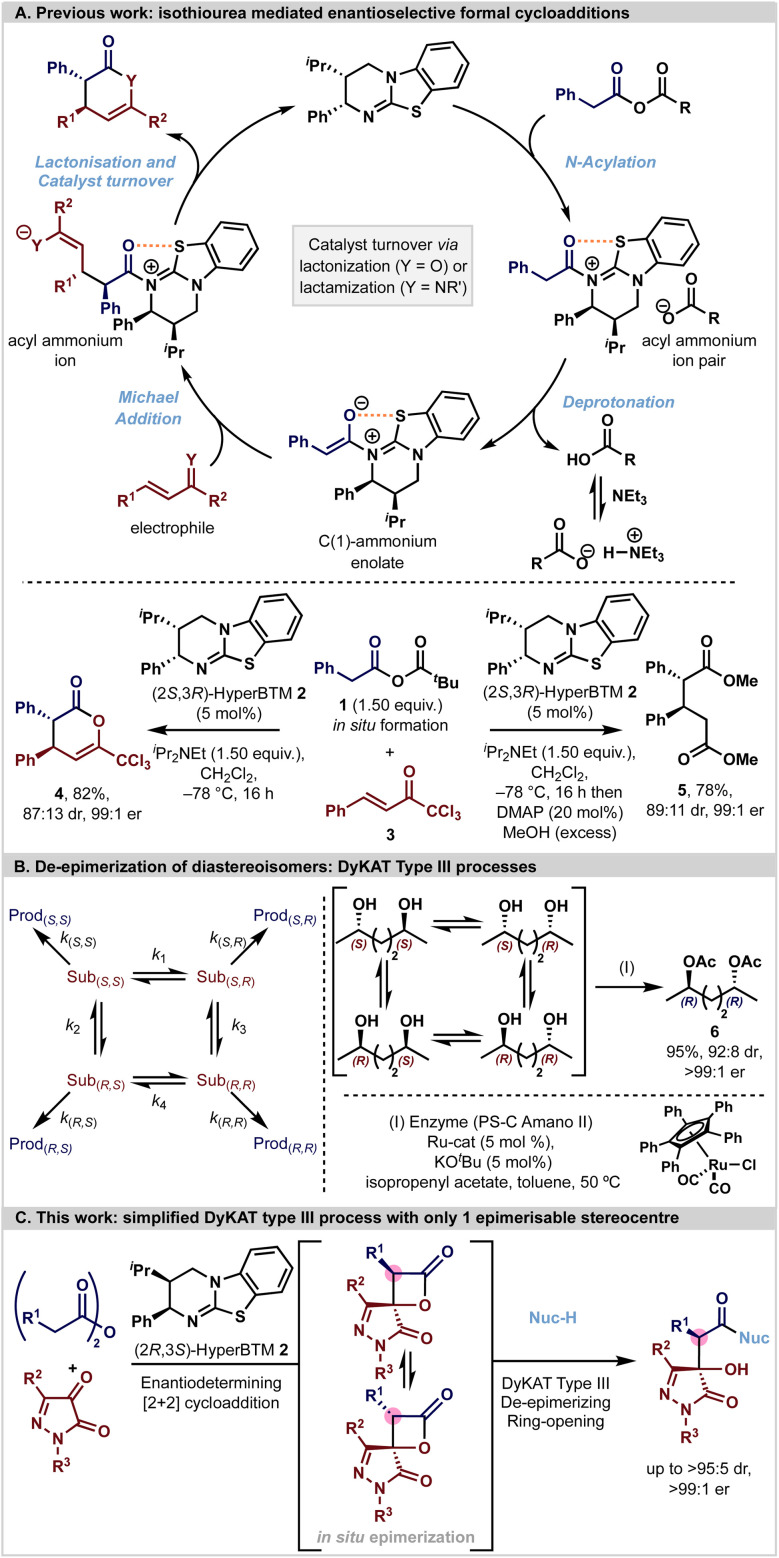
[A] Previous work. [B] DyKAT type III de-epimerization processes. [C] This work: coupling an enantioselective catalytic [2 + 2]-cycloaddition with a DyKAT type III ring-opening process.

In recent years enantiopure tertiary amine Lewis bases have been utilised as efficient catalysts for the synthesis of a range of enantioenriched chiral building blocks. Within this arena, isothioureas have been widely exploited through harnessing the reactivity of acyl ammonium,^6,15–17^ α,β-unsaturated acyl ammonium^18,19^ and C(1)-ammonium enolate intermediates.^20,21^ In particular, the ability to generate C(1)-ammonium enolate intermediates from carboxylic acid derivatives (*via* an *in situ* formed mixed anhydride), followed by stereoselective [*n* + 2]-cycloadditions (*n* = 2, 3, 4) has been exploited by ourselves and others (Fig. 1A).^21–35^ In such processes, catalyst turnover is typically reliant upon an intramolecular cyclisation event from a pendant heteroatom nucleophile (often either O or N) to generate a heterocyclic lactone or lactam product. While this catalytic strategy is powerful, in many circumstances the resultant heterocyclic lactone products can be difficult to isolate in high yields due to facile ring-opening and decomposition on attempted chromatographic purification. Consequently, a common tactic to allow for effective product isolation is to add a nucleophilic amine or alcohol to give an isolable product in high yield and stereoselectivity. For example, generation of the C(1)-ammonium enolate derived from mixed anhydride 1 with HyperBTM (2*S*,3*R*)-2 and subsequent Michael addition–lactonisation using trichloromethylenone (also known as trifluoromethyl 2-phenylvinyl ketone or 1,1,1-trichloro-4-phenyl-3-en-2-one) 3 generates δ-lactone 4 bearing two stereogenic centres in 87 : 13 dr and 99 : 1 er. *In situ* derivatisation of the δ-lactone with DMAP and MeOH generates the ring opened product 5 (89 : 11 dr, 99 : 1 er) consistent with MeOH promoted ring-opening proceeding without epimerisation.

In this context, the chemical community has become increasingly aware of the power of dynamic kinetic asymmetric transformations (DyKAT) that can be used to deliver compounds containing one or multiple defined stereogenic elements with high stereocontrol. In such reaction processes, diastereoisomeric interconversion of intermediates or starting materials, combined with subsequent preferential reaction of one diastereoisomer, can lead to enhanced product stereoselectivity. Since the term DyKAT was introduced by Trost in 2000,^36^ a plethora of applications of this strategy has been developed and recognised, and this area has been extensively reviewed. Four subtypes (I to IV) of DyKAT have been categorised, observed, and developed to date, that either involve the deracemization of enantiomers through diastereoisomeric intermediates (types I and II) or the de-epimerization of diastereoisomers (types III and IV). In type III and IV systems, diastereoisomer interconversion is harnessed alongside a (usually catalyst controlled) selective reaction that results in stereoisomeric resolution and determines the relative and absolute configuration within the desired product stereoisomer. A representative example of a DyKAT type III process is exemplified schematically in Fig. 1B. In this case epimerization of both stereogenic centres within the 1,4-diol substrate are promoted by reversible (de)hydrogenation using the Ru-catalyst, that is followed by consecutive enantioselective acylations, allowing the selective generation of the (*R*,*R*)-6 in 95% yield, 92 : 8 dr and >99 : 1 er from a 50 : 50 mixture of racemic and *meso*-starting materials.^37^

Building upon these principles and the growing impact of organocatalysis in the pharmaceutical industry,^38–42^ in this manuscript an effective synthetic strategy for the preparation of functionalised β-hydroxy-β-pyrazolone amides in high dr and er (up to >95 : 5 dr, >99 : 1 er) is described. Given that nitrogen-containing heterocycles are incorporated into an array of biologically relevant compounds,^43–46^ and with pyrazole derivatives a prevalent heterocycle of widespread relevance,^47–52^ the generation of catalytic enantioselective methods that would lead to chiral pyrazolone heterocycle derivatives was targeted.^53–56^ The developed process combines an isothiourea-mediated highly enantioselective formal [2 + 2]-cycloaddition of C(1)-ammonium enolates to generate diastereoisomeric spirocyclic β-lactones, coupled with a subsequent substrate-controlled DyKAT type III process where the absolute configuration at C(3) within the β-lactone is labile and that at C(4) is fixed (Fig. 1C). Although the [2 + 2]-cycloaddition proceeds with high enantioselectivity (typically 99 : 1 er), only moderate diastereoselectivity is observed upon β-lactone isolation (typically ≈ 70 : 30 dr) presumably due to facile epimerisation. However, *in situ* ring-opening of this diastereoisomeric mixture with a nucleophile generates functionalised β-hydroxy-β-pyrazolones exhibiting exceptional diastereocontrol (up to >95 : 5 dr). Mechanistic and DFT analysis is consistent with a post-catalysis DyKAT type III process leading to the observed enhanced stereoselectivity (products in up to 98% yield, >95 : 5 dr, >99 : 1 er).

## Results and discussion

**Scheme 1:**
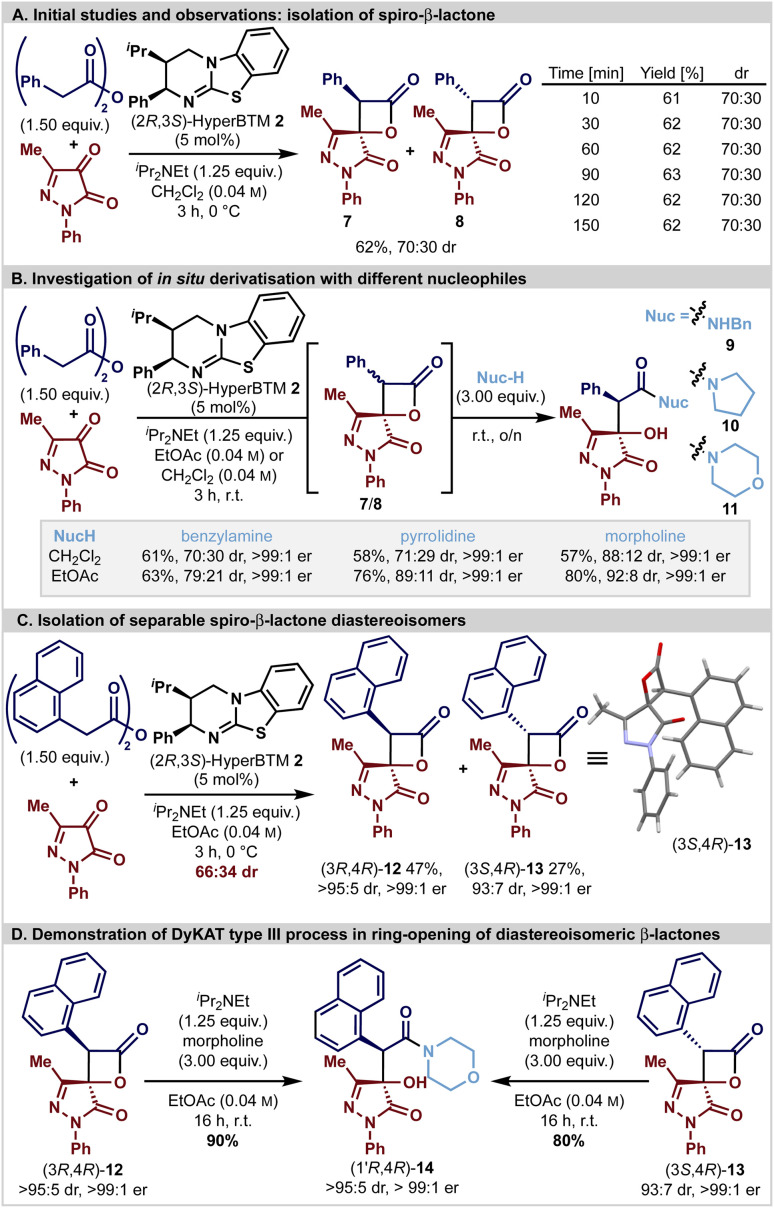
Reactions performed on 0.25 mmol scale with 1.0 equiv. of 3-methyl-1-phenylpyrazol-3,4-dione and 1.5 equiv. of homoanhydride. Product dr assessed by ^1^H NMR analysis of the crude reaction mixture. Yields are isolated yields after chromatographic purification; all product ers are determined by HPLC analysis on a chiral stationary phase.

Initial studies (Scheme 1A) focused upon the isothiourea HyperBTM-catalysed generation of spirocyclic β-lactone 7/8 derived from phenylacetic anhydride (1.5 equiv.) and 3-methyl-1-phenylpyrazol-4,5-dione in CH_2_Cl_2_ in the presence of ^*i*^Pr_2_NEt (1.25 equiv.). Good conversion to the desired product was observed after 3 hours, giving the desired spirocyclic β-lactones 7 and 8 as an inseparable 70 : 30 mixture of diastereoisomers in 62% yield. As previous work has shown that β-lactones are prone to epimerisation at C(3) under basic reaction conditions, *in situ* reaction monitoring in CD_2_Cl_2_ using ^1^H NMR spectroscopic analysis demonstrated that the product β-lactone dr was invariant with time, presumably due to rapid epimerisation leading to equilibration.^24–26,28,57,58^ Subsequent studies were aimed at *in situ* derivatisation to facilitate diastereoisomer separation and allow unambiguous determination of enantioselectivity. Interestingly, the use of benzylamine or pyrrolidine as a derivatisation agent gave an approximate 70 : 30 mixture of the corresponding diastereoisomeric β-hydroxyamides 9 and 10 (both in 99 : 1 er). Addition of morpholine gave β-hydroxyamide 11 with significantly improved diastereoselectivity (88 : 12 dr, >99 : 1 er) providing the first observation of a potential dynamic process being involved in the ring-opening of the β-lactone. Further optimisation of this two-step, one-pot protocol through solvent variation (see ESI†) revealed that improved diastereoselectivity could be achieved in ethyl acetate at room temperature; the addition of benzylamine or pyrrolidine gave 9 (79 : 21 dr, >99 : 1 er) or 10 (89 : 11 dr, >99 : 1 er), respectively, while morpholine gave 11 (92 : 8 dr, >99 : 1 er) (Scheme 1B). Intrigued by these observations, the potential involvement of a substrate controlled DyKAT type III process was probed. HyperBTM-mediated formal [2 + 2]-cycloaddition using 1-naphthylacetic anhydride and 3-methyl-1-phenylpyrazol-4,5-dione gave a separable 66 : 34 mixture of diastereomeric β-lactones (3*R*,4*R*)-12 and (3*S*,4*R*)-13 that were isolated in 47% yield (>95 : 5 dr, >99 : 1 er) and 27% yield (93 : 7 dr, >99 : 1 er), respectively (Scheme 1C). The relative configuration of both stereoisomers was initially confirmed by nOe analysis, and subsequently the absolute (3*S*,4*R*)-configuration within the minor diastereoisomer 13 was unambiguously assigned by single crystal X-ray diffraction. Independent treatment of both diastereoisomers (3*R*,4*R*)-12 and (3*S*,4*R*)-13 with morpholine (3 equiv.) and ^*i*^Pr_2_NEt (1.25 equiv.) both gave β-hydroxyamide (1′*R*,4*R*)-14 in 90% and 80% isolated yield as a single diastereoisomer (>95 : 5 dr, >99 : 1 er) (Scheme 1D). The stereoconvergence observed under these reaction conditions is consistent with an *in situ* epimerisation process at C(3) of the β-lactone, coupled with diastereoselective ring-opening with the amine nucleophile as expected for a DyKAT type III process.^59^ Consistent with this hypothesis, treatment of (3*S*,4*R*)-13 (97 : 3 dr) with ^*i*^Pr_2_NEt (10 equiv.) in CDCl_3_ led to C(3)-epimerisation, giving an 78 : 22 mixture of (3*R*,4*R*)-12 : (3*S*,4*R*)-13 after 21 hours.

**Fig. 2:**
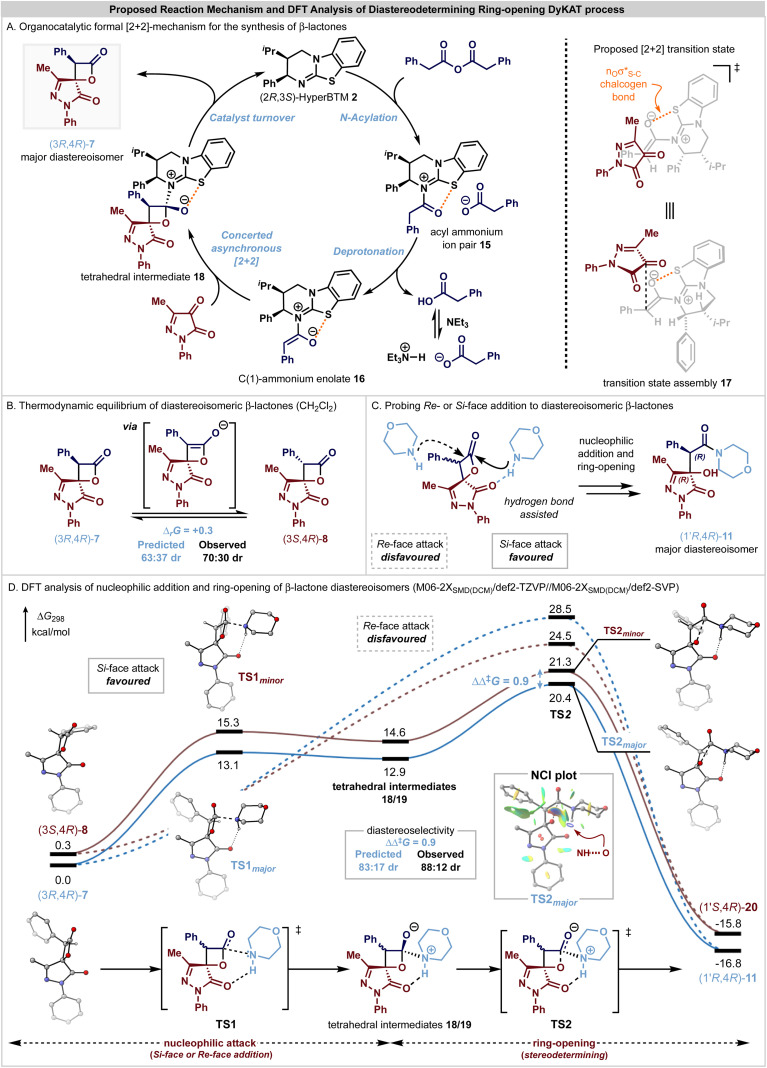
DFT analysis of pathways from the interconvertible (3*R*,4*R*)-7 and (3*S*,4*R*)-8 β-lactone diastereoisomers. M06-2X_SMD_/def2-TZVP//M06-2X_SMD_/def2-SVP Gibbs free energies (Δ*G*_298_) are shown in kcal mol^−1^ and selected hydrogen atoms have been removed for clarity.

Based upon these observations and following established precedent in this area, a catalytic cycle for the initial enantioselective formal [2 + 2]-cycloaddition, followed by a DyKAT type III ring-opening process upon addition of an amine is proposed to account for these observations (Fig. 2). The catalytic cycle to generate the β-lactone products (Fig. 2A) is initiated by *N*-acylation of the phenylacetic anhydride by the addition of the Lewis base (2*R*,3*S*)-HyperBTM 2 to generate acyl ammonium ion pair 15. Subsequent deprotonation generates the corresponding (*Z*)-ammonium enolate 16,^60^ with a stabilizing 1,5-O⋯S chalcogen bonding interaction 
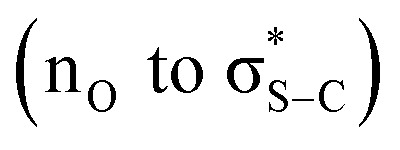
^61–80^ ensuring coplanarity between the 1,5-O- and S-atoms and providing a conformational bias. The relative and absolute configuration observed within the major diastereoisomer of the β-lactone products is consistent with that previously observed in related [2 + 2]-cycloadditions of C(1)-ammonium enolates with trifluoromethylketones,^22–25^ and so by analogy a similar concerted asynchronous [2 + 2]-cycloaddition pathway *via* transition state assembly 17 to give 7 is proposed. Subsequent catalyst release *via* collapse of the tetrahedral intermediate 18 generates the β-lactone 7 in high enantioselectivity, with *in situ* epimerization of the lactone at C(3) leading to the isolable mixture of β-lactone diastereoisomers 7 and 8. Under basic conditions, arising from treatment with a secondary amine nucleophile and ^*i*^Pr_2_NEt, epimerization and ring-opening from the addition of the secondary amine nucleophile promotes the DyKAT process to give the isolable β-hydroxyamide products in high er and with enhanced dr compared to the starting β-lactone mixture.

The enhancement in diastereocontrol observed upon ring opening of the β-lactone diastereomers was investigated through DFT calculations at the M06-2X_SMD_/def2-TZVP//M06-2X_SMD_/def2-SVP level using Gaussian16.^81–87^ The ring-opening of substrate diastereoisomers (3*R*,4*R*)-7 and (3*S*,4*R*)-8 with morpholine as a nucleophile were modelled, with calculations performed in CH_2_Cl_2_ solvation, in line with the initial experimental conditions that gave a thermodynamic ratio of β-lactone diastereoisomers in 70 : 30 dr (Scheme 1A) and gave the β-hydroxyamide products in 88 : 12 dr after ring-opening (Scheme 1B). CH_2_Cl_2_ was chosen due to its properties as a non-coordinating solvent, avoiding complexities arising from solvents that can accept hydrogen-bonds, which cannot be described accurately with only a continuum dielectric model. The enrichment in diastereocontrol observed upon ring-opening reflects a DyKAT process as the stereocontrol transitions from thermodynamic equilibrium of the β-lactone diastereoisomers to kinetic selectivity to achieve the β-hydroxyamide products in high dr. Calculation of the Gibbs free energies of the β-lactone diastereoisomers (3*R*,4*R*)-7 and (3*S*,4*R*)-8 resulted in a computed thermodynamic ratio of 63 : 37 (Δ_r_*G* = 0.3 kcal mol^−1^, Fig. 2B) that is in close agreement with the observed 70 : 30 dr. Consistent with the epimerization control experiments described above, under the basic reaction conditions it is assumed that diastereoisomers 7 and 8 rapidly interconvert by *in situ* epimerization due to the acidity of the β-lactone C(3)–H, contributing to the enrichment during the subsequent nucleophilic addition step.

The approach of the morpholine nucleophile from either the *Re*- or the *Si*-face of either β-lactone diastereoisomer was modelled (Fig. 2C and D), with calculations revealing a strong preference towards addition to the *Si*-face of both diastereoisomers, due to a stabilising hydrogen bonding interaction between the pyrazolone C=O̲ and morpholine N–H̲. This stabilising interaction was observed for all structures, with increasing elongation of the N–H bond relative to free morpholine as the reaction progresses (Table S4†). In contrast, reactivity through the disfavoured *Re*-face of the β-lactone (Fig. 2C and D, dashed lines) does not benefit from a stabilising intramolecular hydrogen-bond, and instead a strongly asynchronous concerted process is followed, with no distinct tetrahedral intermediate located connecting the reactant encounter complex to the product. This pathway is calculated to be significantly energetically disfavoured and predicts a reversal of selectivity towards the observed minor diastereoisomeric product. The stabilising hydrogen bond interaction mediates nucleophilic attack from the *Si*-face of the (3*R*,4*R*)-7 and (3*S*,4*R*)-8 β-lactones *via* TS1_major_ and TS1_minor_, respectively, to generate the corresponding tetrahedral intermediates 18/19. The presence of this hydrogen bond is also apparent from the non-covalent interaction (NCI) plot inset in Fig. 2. Subsequent ring opening *via* TS2_major_ and TS2_minor_ is predicted to be stereodetermining (ΔΔ^‡^*G* = 0.9 kcal mol^−1^), preferentially generating the β-hydroxyamide products (1′*R*,4*R*)-11 and (1′*S*,4*R*)-20 in a calculated 83 : 17 dr that is in close agreement with the observed 88 : 12 dr. Subsequent proton transfer, likely facilitated by an excess of morpholine, generates the product.

The observed diastereoselectivity is governed by kinetic control of the ring-opening process *via* TS2_major_ and TS2_minor_, with the C(3)–Ph unit preferentially orientated away from the morpholine nucleophile in the major pathway. Reactivity through this diastereoisomer reduces the reorganization required by the system prior to the ring-opening TS, which can be quantified by evaluating the energy change associated with geometry perturbation of the two reacting fragments from their relaxed to reactive conformations.^88^ Notably, the major transition state has a lower reorganisation energy (ΔΔ*E*_reorg_ = 2.3 kcal mol^−1^, see ESI† for details), contributing to the difference in free energy between TS2_major_ and TS2_minor_.

## Scope and limitations

**Scheme 2:**
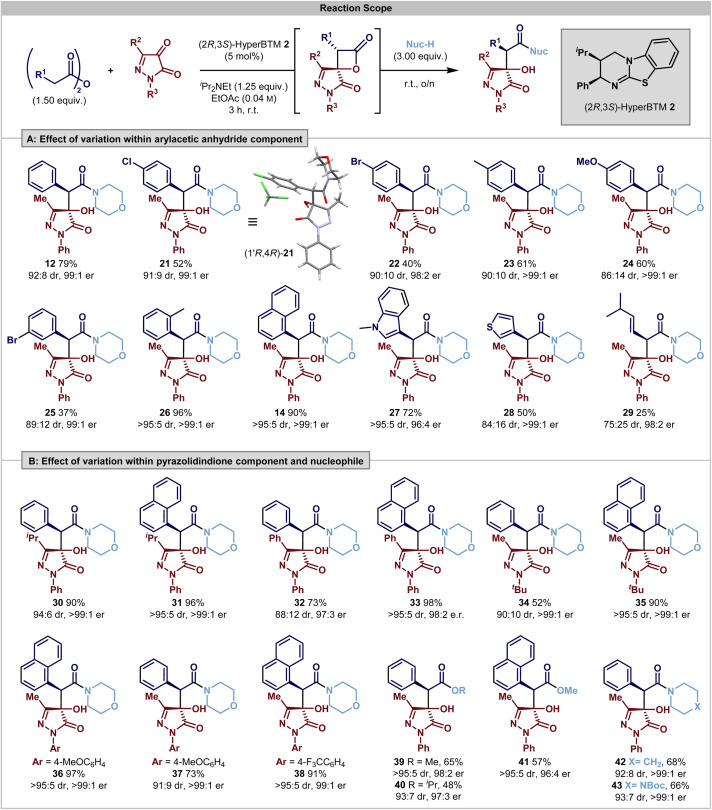
All reactions performed on 0.25 mmol scale with 1.0 equiv. of dione and 1.5 equiv. of anhydride. Product dr assessed by ^1^H NMR analysis of the crude reaction mixture. Yields are isolated yields after chromatographic purification; all product ers are determined by HPLC analysis on a chiral stationary phase.

Having identified the operation of a post-catalysis substrate controlled DyKAT type III process, the scope and limitations of this process were investigated. Using 3-methyl-1-phenylpyrazolidin-3,4-dione, initial studies probed the effect upon product selectivity of substituent variation within the anhydride reaction component (Scheme 1A). The incorporation of halogenated 4-ClC_6_H_4_ and 4-BrC_6_H_4_ substituents was tolerated, giving products 21 and 22 in acceptable yields and with excellent stereoselectivity (91 : 9 and 90 : 10 dr, 98 : 2 er). The relative and absolute configuration within (3*R*,4*R*)-21 was confirmed by single crystal X-ray diffraction, with the configuration within all other products assigned by analogy. Incorporation of electron-donating 4-MeC_6_H_4_ and 4-MeOC_6_H_4_ substituents were also tolerated, giving products 23 and 24 in good yields (61% and 60%, respectively) and with excellent enantioselectivity (>99 : 1 er), although with reduced diastereocontrol for the 4-MeOC_6_H_4_ substituent (86 : 14 dr), presumably reflecting the reduced acidity of the C(3)–H within the corresponding β-lactone, leading to a reduced rate of epimerisation. 3-BrC_6_H_4_-substitution resulted in reduced product yield, but good stereocontrol (25, 37%, 89 : 11 dr, 99 : 1 er) while notably 2-MeC_6_H_4_-substitution gave 26 in excellent yield and with improved stereocontrol (96%, >95 : 5 dr, >99 : 1 er). The trend for improved selectivity with *ortho*-substituted aromatic substituents was also observed with 1-naphthyl- and 1-methylindol-3-yl-substitution, to give products in excellent yields and selectivity (14, 90%, >95 : 5 dr, >99 : 1 er, 27; 72%, >95 : 5 dr, 96 : 4 er, respectively). However, the incorporation of anhydride substituents with reduced steric bulk led to reduced product diastereoselectivity; for example (3-thiophenyl 28 and 3-methylbuten-1-yl 29) both gave products in excellent enantioselectivity, but in 84 : 16 dr and 75 : 25 dr, respectively. This presumably reflects subtle changes in the effectiveness of the DyKAT process, including decreased acidity and thus reduced stereointegrity of the initially formed β-lactone coupled with changes in stereocontrol upon ring-opening. Further investigations probed the effect of substituent variation at both N(1) and C(3) within the pyrazolidine-3,4-dione reaction component using both phenylacetic anhydride and 1-naphthylacetic anhydride (Scheme 2B). Notable general trends within this series showed that variation of the steric and electronic effects of substituents at both N(1)- and C(3)-positions were tolerated, giving products with generally excellent enantioselectivity, with the use of 1-naphthylacetic anhydride generally giving rise to increased diastereocontrol. For example, with an N(1)–Ph substituent, C(3)–^*i*^Pr or C(3)–Ph substitution led to products 30–33 with excellent stereocontrol. Variation of the N(1)-substituent seemed to be inconsequential to reactivity. The effect of steric hindrance was probed through the incorporation of an N(1)–^*t*^Bu substituent, giving 34 and 35 with excellent enantiocontrol, while the incorporation of both electron-donating (4-MeOC_6_H_4_) and electron-withdrawing (4-CF_3_C_6_H_4_) N(1)-substitution was also tolerated, giving 36–38 in good to excellent yield and stereocontrol. Furthermore, expansion of the nucleophile used for the ring-opening of the lactone showed that simple alcohols could also be used in conjunction with DMAP as a catalyst to promote the DyKAT ring-opening process; the use of MeOH and 20 mol% DMAP gave 39 and 41 in reasonable yield with excellent diastereo- and enantiocontrol. Changing to ^*i*^PrOH in this process gave 40 in 41% yield (80 : 20 er, 93 : 7 dr) using 20 mol% of DMAP, with reduced quantities of DMAP (0.5 mol%) leading to 40 in 48% yield, 93 : 7 dr and with improved 97 : 3 er. This is consistent with the assumed lower nucleophilicity of ^*i*^PrOH compared to MeOH, combined with the DMAP catalyst promoting competitive reversible [2 + 2] cycloaddition. Alternative cyclic amine nucleophiles, piperidine and *N*-Boc piperazine, were also used in this process, generating 42 and 43 with excellent stereocontrol (92 : 8 dr and 97 : 3 dr respectively; >99 : 1 er).

## Conclusions

Although moderate diastereoselectivity (typically 70 : 30 dr) is observed in the isothiourea-catalysed [2 + 2]-cycloaddition of C(1)-ammonium enolates with pyrazol-4,5-diones, subsequent ring-opening with morpholine generates β-hydroxyamide products with enhanced stereoselectivity (up to >95 : 5 dr). Stereoconvergence is observed in the ring-opening of enantiopure diastereoisomeric β-lactones, leading to a single product (>95 : 5 dr, >99 : 1 er). Mechanistic studies and DFT analysis indicate a substrate controlled DyKAT involving epimerisation at C(3) of the β-lactone under the reaction conditions, coupled with a hydrogen bond-assisted nucleophilic addition to the *Si*-face of the β-lactone and stereodetermining ring-opening. The scope and limitations of a one-pot protocol consisting of isothiourea-catalysed *enantio*-determining [2 + 2] cycloaddition followed by ring-opening is subsequently developed. Variation within the anhydride ammonium enolate precursor, as well as N(1)- and C(3)- within the pyrazol-4,5-dione scaffold is demonstrated, giving a range of functionalised β-hydroxyamides with high diastereo- and enantiocontrol (>20 examples, up to >95 : 5 dr and >99 : 1 er) *via* this DyKAT. Further work from this laboratory will identify and exploit alternative dynamic kinetic asymmetric processes for the preparation of a range of functionalised stereodefined building blocks.

## Data availability

All data (experimental procedures, characterization data and Cartesian coordinates for all DFT calculations) that support the findings of this study are available within the article and its ESI.† Crystallographic data for compounds (3*S*,4*R*)-13 and (1′*R*,4*R*)-21 have been deposited with the Cambridge Crystallographic Data Centre under deposition numbers 2314276 and 2314277, respectively. The research data supporting this publication can be accessed from “De-epimerizing DyKAT of β-Lactones Generated by Isothiourea-Catalysed Enantioselective [2 + 2] Cycloaddition”. University of St Andrews Research Portal https://doi.org/10.17630/150e0963-c5ba-4fdc-8e59-90724e13ac8e.

## Author contributions

ADS conceived the project; AC and KK carried out all experimental studies and analysed data for all compounds in consultation with JD and KK. ADS, ASG and KK cowrote the manuscript. DBC carried out single crystal X-ray analysis. ASG carried out computational analysis in consultation with MB. All authors agreed on the finalised version of the manuscript.

## Conflicts of interest

The authors declare no competing interests.

## Supplementary Material

SC-015-D4SC01410C-s001

SC-015-D4SC01410C-s002
