# Supplementary material for: De-epimerizing DyKAT of β-lactones generated by isothiourea-catalysed enantioselective [2 + 2] cycloaddition
Source: Chem Sci. 2024 Apr 24;15(23):8896–904. doi: 10.1039/d4sc01410c (PMC11168096; doi:10.1039/d4sc01410c)
Supplement: SC-015-D4SC01410C-s001 [file SC-015-D4SC01410C-s001.pdf]

Electronic Supplementary Material (ESI) for Chemical Science.
This journal is © The Royal Society of Chemistry 2024

De-epimerizing DyKAT of β-Lactones Generated by Isothiourea-Catalysed Enantioselective [2+2]-Cycloaddition

Aífe Conboy, Alister S. Goodfellow, Kevin Kasten, Joanne Dunne, David B. Cordes, Michael Bühl,* Andrew D. Smith*

EaStCHEM, School of Chemistry, University of St Andrews, St Andrews, Fife, KY16 9ST, UK.

E-mail: ads10@st-andrews.ac.uk; buehl@st-andrews.ac.uk

Contents

1. **General experimental** 5
2. **Reaction Optimisation** 7
1. **Synthesis of Homoanhydrides** 8
   1.1. 2-Phenylacetic Anhydride **S1** 8
   1.2. 2-(α-Naphthyl)acetic Anhydride **S2** 8
   1.3. 2-(*p*-Chlorophenyl)acetic Anhydride **S3** 8
   1.4. 2-(*p*-Bromophenyl)acetic Anhydride **S4** 9
   1.5. 2-(*p*-Tolyl)acetic Anhydride **S5** 9
   1.6. 2-(*p*-Anisyl)acetic Anhydride **S6** 9
   1.7. 2-(*m*-Bromophenyl)acetic Anhydride **S7** 10
   1.8. 2-(*o*-Tolyl)acetic Anhydride **S8** 10
   1.9. 2-(*N*-Methylindol-3-yl)acetic Anhydride **S9** 10
   1.10. 2-(Thiophen-3-yl)acetic Anhydride **S10** 11
   1.11. (*E*)-5-Methylhex-3-enioc Anhydride **S11** 11
2. **Synthesis of Pyrazol-3-ones** 12
   2.1. 5-Methyl-2-phenyl-2,4-dihydro-3*H*-pyrazol-3-one **S12** 12
   2.2. 5-Isopropyl-2-phenyl-2,4-dihydro-3*H*-pyrazol-3-one **S13** 12
   2.3. 2,5-Diphenyl-2,4-dihydro-3*H*-pyrazol-3-one **S14** 12
   2.4. 2-(*tert*-Butyl)-5-methyl-2,4-dihydro-3*H*-pyrazol-3-one **S15** 13
   2.5. 2-(*p*-Anisyl)-5-methyl-2,4-dihydro-3*H*-pyrazole-3-one **S16** 13
   2.6. 5-Methyl-2-(4-(trifluoromethyl)phenyl)-2,4-dihydro-3*H*-pyrazol-3-one **S17** 14
3. **Synthesis of Pyrazol-3-one-derived ketimines** 14
   3.1. 5-Methyl-2-phyenyl-4-(phenylimino)-2,4-dihydro-3*H*-pyrazol-3-one **S18** 14
   3.2. 5-Isopropyl-2-phenyl-4-(phenylimino)-2,4-dihydro-3*H*-pyrazol-3-one **S19** 15
   3.3. 2,5-Diphenyl-4-(phenylimino)-2,4-dihydro-3*H*-pyrazol-3-one **S20** 15
   3.4. 2-(*tert*-Butyl)-5-methyl-4-(phenylimino)-2,4-dihydro-3*H*-pyrazol-3-one **S21** 16

4. Synthesis of Pyrazole-4,5-diones 17
4.1. 3-Methyl-1-phenyl-1*H*-pyrazole-4,5-dione **S22** 17
4.2. 3-Isopropyl-1-phenyl-1*H*-pyrazole-4,5-dione **S23** 17
4.3. 1,3-Diphenyl-1*H*-pyrazole-4,5-dione **S24** 18
4.4. 1-(*tert*-Butyl)-3-methyl-1*H*-pyrazole-4,5-dione **S25** 18
4.5. 1-(*p*-Anisyl)-3-methyl-1*H*-pyrazole-4,5-dione **S26** 19
4.6. 3-Methyl-1-(*p*-(trifluoromethyl)phenyl)-1*H*-pyrazole-4,5-dione **S27** 20
5. Isothiourea-catalysed formal [2+2] cycloaddition of homoanhydrides and pyrazole-4,5-diones 21
5.1. *N*-Benzyl (2'*R*,4*R*)-2-(4-hydroxy-3-methyl-5-oxo-1-phenyl-4,5-dihydro-1*H*-pyrazol-4-yl)-2-phenylacetamide **9** 21
5.2. (1'*R*,4*R*)- and (1'*S*,4*R*)-4-hydroxy-5-methyl-4-(2-oxo-1-phenyl-2-(pyrrolidine-1-yl)ethyl)-1-phenyl-2,4-dihydro-3*H*-pyrazol-3-one **10** 24
5.3. (1'*R*,4*R*)- and (1'*S*,4*R*)-4-Hydroxy-5-methyl-4-(2-mopholino-2-oxo-1-phenylethyl)-2-phenyl-2,4-dihydro-3*H*-pyrazol-3-one **11** 27
5.4. (3'*R*,4*R*)-3-Methyl-3'-(naphth-1-yl)-1-phenyl-spiro[pyrazolin[5]one-4.2-oxetan[4]one] **12** and (3'*S*,4*R*)-3-Methyl-3'-(naphth-1-yl)-1-phenyl-spiro[pyrazolin[5]one-4.2-oxetan[4]one] **13** 30
5.5. (1'*R*,4*R*)- and (1'*S*,4*R*)-4-hydroxy-5-methyl-4-(2-morpholino-1-(α-naphthyl)-2-oxoethyl)-2-phenyl-2,4-dihydro-3*H*-pyrazol-3-one **14** 36
5.6. (1'*R*,4*R*)- and (1'*S*,4*R*)-4-(1-(*p*-chlorophenyl)2-morpholino-2-oxoethyl)-4-hydroxy-5-methyl-2-phenyl-2,4-dihydro-3H-pyrazol-3-one **21** 39
5.7. (1'*R*,4*R*)- and (1'*S*,4*R*)-4-(1-(4-bromophenyl)-2-morpholino-2-oxoethyl)-4-hydroxy-5-methyl-2-phenyl-2,4-dihydro-3*H*-pyrazole-3-one **22** 42
5.8. (1'*R*,4*R*)- and (1'*S*,4*R*)-4-hydroxy-5-methyl-4-(2-morphline-2-oxo-1-(*p*-tolyl)ethyl)-2-phenyl-2,4-dihyro-3*H*-pyrazol-3-one **23** 45
5.9. (1'*R*,4*R*)-4-(1-(*p*-anisyl)-2-morpholine-2-oxoethyl)-4-hydroxy-5-methyl-2-phenyl-2,4-dihydro-3*H*-pyrazol-3-one **24** 48
5.10. (1'*R*,4*R*)- and (1'*S*,4*R*)-4-(1-(*m*-bromophenyl)-2-morpholine-2-oxoethyl)-4-hydroxy-5-methyl-2-phenyl-2,4-dihydro-3*H*-pyrazol-3-one **25** 51
5.11. (1'*R*,4*R*)- and (1'*S*,4*R*)-4-hydroxy-5-methyl-4-(2-morpholino-2-oxo-1-(*o*-tolyl)ethyl)-2-phenyl-2,4-dihydro-3*H*-pyrazol-3-one **26** 54
5.12. (1'*R*,4*R*)-4-hydroxy-5-methyl-4-(1-(1-methyl-1*H*-indol-3-yl)-2-morpholino-2-oxoethyl)-2-phenyl-2,4-dihydro-3*H*-pyrazol-3-one **27** 57
5.13. (1'*R*,4*R*)- and (1'*S*,4*R*)-4-hydroxy-5-methyl-4-(2-morpholino-2-oxo-1-thiophen-2-ylethyl)-2-phenyl-2,4-dihydro-3*H*-pyrazol-3-one **28** 60
5.14. (2'*R*,4*R*)- and (2'*S*,4*R*)-(*E*)-4-hydroxy-5-methyl-4-(5-methyl-1-morpholino-1-oxohex-3-en-2-yl)-2-phenyl-2,4-dihydro-3*H*-pyrazol-3-one **29** 62
5.15. (1'*R*,4*R*)- and (1'*S*,4*R*)-4-Hydroxy-5-isopropyl-4-(2-morpholino-2-oxo-1-phenylethyl)-2-phenyl-2,4-dihydro-3*H*-pyrazol-3-one **30** 65

5.16. (1'*R*,4*R*)- and (1'*S*,4*R*)-4-Hydroxy-5-isopropyl-4-(2-morpholino-1-(naphth-1-yl)-2-oxoethyl)-2-phenyl-2,4-dihydro-3*H*-pyrazol-3-one **31** ... 69

5.17. (1'*R*,4*R*)- and (1'*S*,4*R*)-4-Hydroxy-4-(2-morpholino-2-oxo-1-phenylethyl)-2,5-diphenyl-2,4-dihydro-3*H*-pyrazol-3-one **32** ... 73

5.18. (1'*R*,4*R*)- and (1'*S*,4*R*)-4-Hydroxy-4-(2-morpholino-1-(naphtha-1-yl)-2-oxoethyl)-2,5-diphenyl-2,4-dihydro-3*H*-pyrazol-3-one **33** ... 76

5.19. (1'*R*,4*R*)- and (1'*S*,4*R*)-2-(*tert*-Butyl)-4-hydroxy-5-methyl-4-(2-morpholino-2-oxo-1-phenylethyl)-2,4-dihydro-3*H*-pyrazol-3-one **34** ... 79

5.20. (1'*R*,4*R*)- and (1'*S*,4*R*)-2-(*tert*-Butyl)-4-hydroxy-5-methyl-4-(2-morpholino-1-(naphtha-1-yl)-2-oxoethyl)-2,4-dihydro-3*H*-pyrazol-3-one **35** ... 82

5.21. (1'*R*,4*R*)- and (1'*S*,4*R*)-2-(*p*-Anisyl)-4-hydroxy-5-methyl-4-(2-morpholino-1-(naphtha-1-yl)-2-oxoethyl)-2,4-dihydro-3*H*-pyrazol-3-one **36** ... 85

5.22. (1'*R*,4*R*)- and (1'*S*,4*R*)-2-(*p*-Anisyl)-4-hydroxy-5-methyl-4-(2-morpholino-2-oxo-1-phenylethyl)-2,4-dihydro-3*H*-pyrazol-3-one **37** ... 88

5.23. (1'*R*,4*R*)- and (1'*S*,4*R*)-4-hydroxy-5-methyl-4-(2-morpholino-1-(naphtha-1-yl)-2-oxoethyl)-2-(*p*-trifluoromethylphenyl)-2,4-dihydro-3*H*-pyrazol-3-one **38** ... 91

5.24. (1'*R*,4*R*)- and (1'*S*,4*R*)-2-(4-Hydroxy-3-methyl-5-oxo-1-phenyl-4,5-dihydro-1*H*-pyrazol-4-yl)-2-phenylacetate **39** ... 95

5.25. *iso*-Propyl (2*R*)-2-((4*R*)-4-Hydroxy-3-methyl-5-oxy-1-phenyl-4,5-dihydro-1*H*-pyrazol-4-yl)-2-phenylacetate **40** ... 98

5.26. (1'*R*,4*R*)- and (1'*S*,4*R*)-2-(4-Hydroxy-3-methyl-5-oxo-1-phenyl-4,5-dihydro-1*H*-pyrazol-4-yl)-2-(naphth-1-yl)-acetate **41** ... 103

6. Epimerisation Experiments ... 114

6.1. Epimerisation of Amide **14** ... 114

6.2. Epimerisation of Lactone **13** ... 114

7. Crystallographic Data ... 117

8. Computation ... 124

8.1. Computational details ... 124

8.2. Interaction and Reorganisation Energies ... 124

8.3. Computational Discussion ... 125

8.4. Treatment of Low-Lying Vibrational Frequencies ... 125

8.5. Computational Data ... 126

9. References ... 133

1. General experimental

Reactions involving moisture sensitive reagents were carried out in flame-dried glassware under an inert atmosphere (Ar or N_2) using standard vacuum line techniques. Anhydrous solvents (Et_2O, CH_2Cl_2, THF and PhMe) were obtained after passing through an alumina column (Mbraun SPS-800) or purchased in a sealed bottled under inert atomsphere. Organometallic reagents were titrated before use according to literature procedures.[1] Room temperature (r.t.) refers to 18 ± 3 °C, Petrol refers to petroleum ether with the boiling range of 40 – 60 °C, brine refers to saturated aqueous sodium chloride solution, ether refers to diethylether (Et_2O). All chemicals and solvents used were purchased by pertinent brands (Sigma Aldrich, Alfa Aesar, Acros, Apollo Scientific, TCI, STREM) and used without further purification unless stated. For reactions conducted during the day following cooling baths were applied: 0 °C (ice/water), −10 °C (ice/acetone), −20 °C (ice/NaCl), −45 °C (CO_2(s) or N_2(l)/MeCN), −60 °C (CO_2(s) or N_2(l)/$CHCl_3$) and −78 °C (CO_2(s)/acetone). Temperatures of 0 °C to −78 °C for overnight reactions were obtained using an immersion cooler (HAAKE EK 90). Reactions involving heating were performed using DrySyn blocks or oil baths and a contact thermocouple. Under reduced pressure refers to the use of either a Büchi Rotavapor R-200 with a Büchi V491 heating Bath and Büchi V-800 vacuum controller, a Büchi Rotavapor R-210 with a Büchi V-491 heating bath and Büchi V-850 vacuum controller, a Heidolph Laborota 4001 with vacuum controller, an IKA RV10 rotary evaporator with an IKA HB10 heating bath and ILMVAC vacuum controller, or an IKA RV10 rotary evaporator with an IKA HB10 heating bath and Vacuubrand CVC3000 vacuum controller. Rotary evaporator condensers are fitted to Julabo FL601 Recirculating Coolers filled with ethylene glycol and set to −6 °C.

Analytical thin layer chromatography (TLC)[2] was performed on pre-coated aluminium plates (Kieselgel 60 F_{254} silica) plates purchased from Merck. Visualisation was achieved using ultraviolet light (254 nm) and staining with aqueous $KMnO_4$ or ethanolic vanillin solution followed by heating. Flash column chromatography was performed in glass columns fitted with porosity 3 sintered discs over Silica gel 60 (0.043 – 0.060 mm) using standard techniques as reported in literature with the solvent system stated.[3] Automated chromatography was performed on a Biotage® Selekt™ SEL-2SV with a 200 – 400 nm UV-detector using the method stated and Biotage® Sfär™ Silica HC D or Biotage® Sfär™ Silica D columns.

HPLC analyses were obtained on either a Shimadzu HPLC consisting of a DGU-20A5 degassing unit, LC-20AT liquid chromatography pump, SIL-20AHT autosampler, CMB-20A communications bus module, SPD-M20A diode array detector and a CTO-20A column oven or a Shimadzu HPLC consisting of a DGU-20A5R degassing unit, LC-20AD liquid chromatography pump, SIL-20AHT autosampler, SPD-20A UV/Vis detector and a CTO-20A column oven. Separation was achieved using either DAICEL CHIRALCEL OD-H and OJ-H columns or DAICEL CHIRALPAK AD-H, AS-H, IA, IB, IC and ID columns using the method stated. HPLC traces of enantiomerically enriched compounds were compared with authentic racemic spectra. Racemic compounds were synthesised under analogous reaction conditions using achiral or racemic catalysts where necessary.

Optical rotations were determined using a Perkin Elmer Precisely/Model-341 Polarimeter with a Na/Hal lamp (Na D line, 589 nm) at 20 °C.

Infrared spectra were recorded on a Shimadzu IRAffinity-1 Fourier transform IR spectrophotometer fitted with a Specac Quest ATR accessory (diamond puck). Spectra were recorded of either thin films or solids, with characteristic absorption wavenumbers (ν_{max}) reported in cm^{-1}.

^{1}H, ^{13}C, ^{19}F and ^{32}P nuclear magnetic resonance (NMR) spectra were recorded with Bruker Avance™ 300 Cryomagnet with a BBFO probe, Bruker Avance II™ 400 Ultrashield with a BBFO probe, Bruker Avance™ 500 Ultrashield with a SmartProbe BBFO+ probe or Bruker Avance III™ 500 Ascend™ with a CryoProbe Prodigy BBO probe using deuterated solvents ($CDCl_3$, CD_2Cl_2, D_2O, CD_3OD, CD_3CN, $(CD_3)_2SO$, $(CD_3)_2CO$, $C_6D_5CD_3$) purchased from Sigma-Aldrich. Chemical shifts (δ) are quoted in ppm and referenced to residual solvent signals reported in literature.[4] $^{13}C\{^{1}H\}$ and $^{19}F\{^{1}H\}$ spectra were acquired using a proton broadband decoupling sequence. ^{13}C were recorded with DEPTQ or UDEFT sequences. Couplings were indicated by the use of conventional agreed abbreviations: s (singlet), d (doublet), t (triplet), q (quartet), m (multiplet), dd (doublet of doublets), td (triplet of doublets), etc. Coupling constants (*J*) are denoted with the number of bonds involved in the upper left and with the atoms coupling in the bottom right edge of the symbol, e.g. $^{3}J_{HH}$. The abbreviation *Ar* denotes aromatic and app denotes apparent.[5] NMR peak assignments were confirmed using 2D ^{1}H correlated spectroscopy (COSY), ^{1}D selective ^{1}H nuclear Overhauser effect spectroscopy (NOESY), 2D ^{1}H–^{13}C heteronuclear multiple-bond correlation spectroscopy (HMBC), and 2D ^{1}H–^{13}C heteronuclear single quantum coherence (HSQC) where necessary. For analysis of NMR-spectra MestReNova and tools therein were used.[6] For Karplus analysis transformed equation 2 was used.

Melting points were recorded on an Electrothermal 9100 melting point apparatus and are not corrected; (dec) refers to decomposition.

Mass spectrometry (*m*/*z*) data were acquired using ThermoFisher Exactive Orbitrap mass spectrometer or Micromass GCT (TOF) mass spectrometer with solids probe. Ionisation techniques used are indicated for each compound. Values are quoted as a ratio of mass to charge (*m*/*z*) in Daltons [Da].[7]

Common chemical abbreviations were used to indicate chemical groups or environments such as Ph (phenyl), *Ar* (aromatic, not confuse with Argon), Bn (benzyl), Et (ethyl), Me (methyl). To indicate atoms numbering schemes are displayed with the spectrum and deviate from IUPAC numbering for clarity. For names and numbering concerning stereodiscriptors IUPAC nomenclature was applied.[8]

Authentic racemic samples were prepared in an analogous fashion using racemic HyperBTM.

2. Reaction Optimisation

Table S1: Reaction Optimisation Data

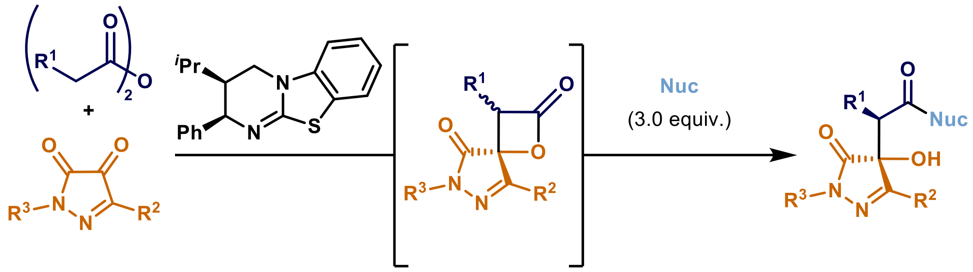

equiv. Catalyst [mol%]	anhydride equiv.	$^{i}Pr_2NEt$ equiv.	$BnNH_2$ equiv.	concentration [M]	time [h]	temperature [°C]	Yield (NMR) [%]	d.r.	e.r.	
5 HyperBTM	1.5	1.25	3	0.04	3	0	76	79:21	>99:1	ref.
5 HBTM	1.5	1.25	3	0.04	3	0	72	79:21	>99:1	cat.
5 BTM	1.5	1.25	3	0.04	3	0	12	N/D	N/D	cat.
5 TM·HCl	1.5	1.25	3	0.04	3	0	<5	N/D	N/D	cat.
5 HyperBTM	1.0	1.25	3	0.04	3	0	69	80:20	>99:1	anh.
5 HyperBTM	2.5	1.25	3	0.04	3	0	69	80:20	>99:1	anh.
5 HyperBTM	1.5	1.00	3	0.04	3	0	72	79:21	>99:1	base
5 HyperBTM	1.5	2.00	3	0.04	3	0	69	80:20	>99:1	base
5 HyperBTM	1.5	1.25	2	0.04	3	0	71	80:20	>99:1	Nuc
5 HyperBTM	1.5	1.25	4	0.04	3	0	73	79:21	>99:1	Nuc
5 HyperBTM	1.5	1.25	3	0.02	3	0	76	79:21	>99:1	conc.
5 HyperBTM	1.5	1.25	3	0.10	3	0	64	80:20	>99:1	conc.
5 HyperBTM	1.5	1.25	3	0.25	3	0	56	79:21	>99:1	conc.
5 HyperBTM	1.5	1.25	3	0.04	3	r.t.	77	79:21	>99:1	temp.
5 HyperBTM	1.5	1.25	3	0.04	3	–20	50	80:20	>99:1	temp.
5 HyperBTM	1.5	1.25	3	0.04	1	r.t.	73	79:21	>99:1	time
1 HyperBTM	1.5	1.25	3	0.04	3	r.t.	71	79:21	>99:1	cat.L.
10 HyperBTM	1.5	1.25	3	0.04	3	r.t.	75	79:21	98:2	cat.L.

1. Synthesis of Homoanhydrides

1.1. 2-Phenylacetic Anhydride **S1**

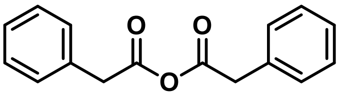

To a solution of 2-phenylacetic acid (6.81 g, 50.0 mmol) in toluene (167 ml, 0.3 M) was added *N*,*N*′-dicyclohexylcarbodiimide (5.16 g, 25.0 mmol) and the resulting mixture was stirred at room temperature for 15 minutes. The reaction mixture was filtered through Celite® and concentrated under reduced pressure to give the title compound as a white crystalline solid (4.30 g, 17.3 mmol, 68%) with data in accordance with the literature.[9] **mp** 68-71 °C (Et_2O) {Lit.[10] 70-72 °C}; **^{1}H NMR** (400 MHz, $CDCl_3$) δ_H: 3.72 (s, 4H, C*H*$_2$), 7.23-7.19 (4H, m, PhC^{2,6}*H*), 7.35-7.27 (6H, m, PhC^{3,4,5}*H*).

1.2. 2-(α-Naphthyl)acetic Anhydride **S2**

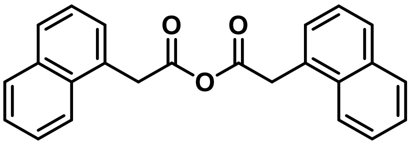

To a solution of 2-(α-naphthyl)acetic acid (2.00 g, 10.7 mmol) in toluene (36 ml, 0.3 M) was added *N*,*N*′-dicyclohexylcarbodiimide (1.22 g, 5.91 mmol) and the resulting mixture was stirred at room temperature for 15 minutes. The reaction mixture was filtered through Celite® and concentrated under reduced pressure to give the title compound as a white crystalline solid (1.13 g, 3.19 mmol, 59%) with data in accordance with the literature.[10] **mp** 116-118 °C (Et_2O) {Lit.[11] 116-117 °C}; **^{1}H NMR** (500 MHz, $CDCl_3$) δ_H: 4.10 (4H, s, C*H*$_2$), 7.20-7.24 (2H, m, ArC^2*H*), 7.31-7.37 (2H, m, ArC^3*H*), 7.45-7.53 (4H, m, ArC^{6,7}*H*), 7.76-7.84 (4H, m, ArC^{4,8}*H*), 7.84-7.90 (2H, m, ArC^5*H*).

1.3. 2-(*p*-Chlorophenyl)acetic Anhydride **S3**

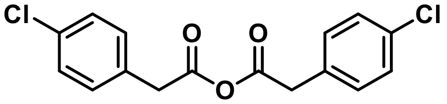

To a solution of 2-(*p*-chlorophenyl)acetic acid (853 mg, 5.00 mmol) in toluene (17 ml, 0.3 M) was added *N*,*N*′-dicyclohexylcarbodiimide (567 mg, 2.75 mmol) and the resulting mixture was stirred at room temperature for 15 minutes. The reaction mixture was filtered through Celite® and concentrated under reduced pressure to give the title compound as a white crystalline solid (407 mg, 1.26 mmol, 56%) with data in accordance with the literature.[9] **mp** 76-78 °C

(Et_2O) {Lit.[10] 62-64 °C}; **^{1}H NMR** (500 MHz, $CDCl_3$) δ_H: 3.70 (4H, s, C*H*$_2$), 7.13 (4H, m, ArC^{2,6}*H*), 7.29 (4H, m, ArC^{3,5}*H*).

1.4. 2-(*p*-Bromophenyl)acetic Anhydride **S4**

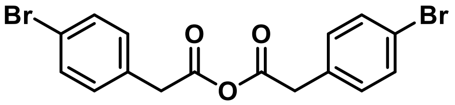

To a solution of 2-(*p*-bromophenyl)acetic acid (850 mg, 3.72 mmol) in toluene (12 ml, 0.3 M) was added *N*,*N*′-dicyclohexylcarbodiimide (422 mg, 2.05 mmol) and the resulting mixture was stirred at room temperature for 15 minutes. The reaction mixture was filtered through Celite® and concentrated under reduced pressure to give the title compound as a white crystalline solid (650 mg, 1.58 mmol, 85%) with data in accordance with the literature.[9] **mp** 92-94 °C (Et_2O) {Lit.[10] 75-77 °C}; **^{1}H NMR** (500 MHz, $CDCl_3$) δ_H: 3.68 (4H, s, C*H*$_2$), 7.04-7.09 (4H, m, ArC^{2,6}*H*), 7.42-7.48 (4H, m, ArC^{3,5}*H*).

1.5. 2-(*p*-Tolyl)acetic Anhydride **S5**

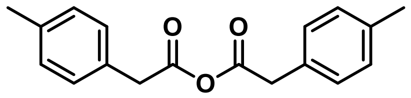

To a solution of 2-(*p*-tolyl)acetic acid (751 mg, 5.00 mmol) in toluene (17 ml, 0.3 M) was added *N*,*N*′-dicyclohexylcarbodiimide (567 mg, 2.75 mmol) and the resulting mixture was stirred at room temperature for 15 minutes. The reaction mixture was filtered through Celite® and concentrated under reduced pressure to give the title compound as a white crystalline solid (386 mg, 1.37 mmol, 55%) with data in accordance with the literature.[9] **mp** 52-55 °C (Et_2O) {Lit.[12] 56-57 °C}; **^{1}H NMR** (500 MHz, $CDCl_3$) δ_H: 2.34 (6H, s, C*H*$_3$), 3.68 (4H, s, C*H*$_2$), 7.07-7.11 (4H, m, ArC^{3,5}*H*), 7.11-7.15 (4H, m, ArC^{2,6}*H*).

1.6. 2-(*p*-Anisyl)acetic Anhydride **S6**

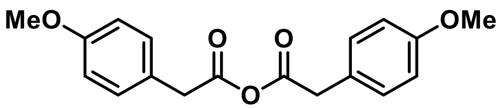

To a solution of 2-(*p*-anisyl)acetic acid (831 mg, 5.00 mmol) in toluene (17 ml, 0.3 M) was added *N*,*N*′-dicyclohexylcarbodiimide (516 mg, 2.50 mmol) and the resulting mixture was stirred at room temperature for 15 minutes. The reaction mixture was filtered through Celite® and concentrated under reduced pressure to give the title compound as a white crystalline solid (362 mg, 1.15 mmol, 46%) with data in accordance with the literature.[13] **mp** 74-76 °C

(Et_2O) {Lit.[13] 77-78 °C}; **^{1}H NMR** (400 MHz, $CDCl_3$) δ_H: 3.66 (4H, s, CH_2), 3.80 (6H, s, OCH_3), 6.81-6.89 (4H, m, ArC^{3,5}H), 7.08-7.15 (4H, m, ArC^{2,6}H).

1.7. 2-(*m*-Bromophenyl)acetic Anhydride **S7**

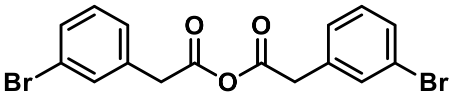

To a solution of 2-(*m*-bromophenyl)acetic acid (800 mg, 3.72 mmol) in toluene (12 ml, 0.3 M) was added *N*,*N*′-dicyclohexylcarbodiimide (422 mg, 2.05 mmol) and the resulting mixture was stirred at room temperature for 15 minutes. The reaction mixture was filtered through Celite® and concentrated under reduced pressure to give the title compound as a white solid (488 mg, 1.18 mmol, 64%) with data in accordance with the literature.[14] **mp** 44-46 °C **^{1}H NMR** (500 MHz, $CDCl_3$) δ_H: 3.70 (4H, s, CH_2), 7.12-7.17 (2H, m, ArC^6H), 7.21 (2H, app t, $^3J_{HH}$ = 7.8 Hz, ArC^5H), 7.38 (2H, app t, $^4J_{HH}$ = 1.9 Hz, ArC^2H), 7.44 (2H, ddd, $^3J_{HH}$ = 7.9 Hz, $^4J_{HH}$ = 1.9 Hz, 1.1 Hz, ArC^4H).

1.8. 2-(*o*-Tolyl)acetic Anhydride **S8**

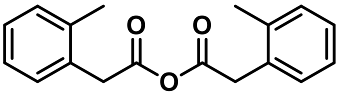

To a solution of 2-(*o*-tolyl)acetic acid (1.00 g, 6.66 mmol) in toluene (22 ml, 0.3 M) was added *N*,*N*′-dicyclohexylcarbodiimide (750 mg, 3.70 mmol) and the resulting mixture was stirred at room temperature for 15 minutes. The reaction mixture was filtered through Celite® and concentrated under reduced pressure to give the title compound as a colourless oil (365 mg, 1.37 mmol, 39%) with data in accordance with the literature.[10] **^{1}H NMR** (500 MHz, $CDCl_3$) δ_H: 2.24 (6H, s, CH_3), 3.72 (4H, s, CH_2), 7.10 (2H, dd, $^3J_{HH}$ = 7.6 Hz, $^4J_{HH}$ = 1.5 Hz, ArCH), 7.12-7.24 (6H, m, ArCH).

1.9. 2-(*N*-Methylindol-3-yl)acetic Anhydride **S9**

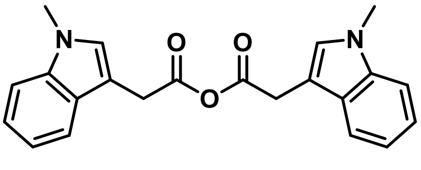

To a solution of 2-(*N*-methylindol-3-yl)acetic acid (1.00 g, 5.29 mmol) in toluene (18 ml, 0.3 M) was added *N*,*N*′-dicyclohexylcarbodiimide (600 mg, 2.90 mmol) and the resulting mixture was stirred at room temperature for 15 minutes. The reaction mixture was filtered through Celite® and concentrated under reduced pressure to give the title compound as a brown oil (810 mg,

2.22 mmol, 85%) with data in accordance with the literature.[15] **^{1}H NMR** (500 MHz, $CDCl_3$) δ_H: 3.70 (6H, s, NCH_3), 3.87 (4H, d, $^4J_{HH}$ = 0.9 Hz, CH_2), 6.93 (2H, d, $^4J_{HH}$ = 0.9 Hz, ArC^2*H*), 7.12 (2H, ddd, $^3J_{HH}$ = 8.0 Hz, 6.8 Hz, $^4J_{HH}$ = 1.2 Hz, Ar*H*), 7.21-7.25 (2H, m, Ar*H*), 7.28-7.30 (2H, m, Ar*H*), 7.50 (2H, app dt, $^3J_{HH}$ = 7.9 Hz, $^4J_{HH}$ = 1.9 Hz, Ar*H*).

1.10. 2-(Thiophen-3-yl)acetic Anhydride **S10**

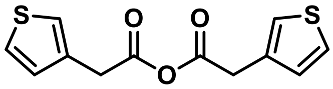

To a solution of 2-(thiophen-3-yl)acetic acid (1.00 g, 7.03 mmol) in toluene (23 ml, 0.3 M) was added *N*,*N*′-dicyclohexylcarbodiimide (798 mg, 3.90 mmol) and the resulting mixture was stirred at room temperature for 15 minutes. The reaction mixture was filtered through Celite® and concentrated under reduced pressure to give the title compound as a pale yellow crystalline solid (365 mg, 1.37 mmol, 39%) with data in accordance with the literature.[9] **mp** 39-40 °C {Lit.[9] 40-42 °C (PhMe)}; **^{1}H NMR** (500 MHz, $CDCl_3$) δ_H: 3.79 (4H, s, CH_2), 6.99 (2H, dd, $^3J_{HH}$ = 5.0 Hz, $^4J_{HH}$ = 1.3 Hz, ArC^5*H*), 7.15 (2H, m, ArC^2*H*), 7.31 (2H, dd, $^3J_{HH}$ = 5.0 Hz, $^4J_{HH}$ = 3.0 Hz, ArC^4*H*).

1.11. (*E*)-5-Methylhex-3-enioc Anhydride **S11**

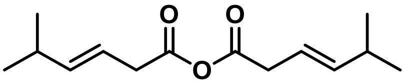

To a solution of (*E*)-5-methylhex-3-enoic acid (1.50 g, 11.70 mmol) in toluene (39 ml, 0.3 M) was added *N*,*N*′-dicyclohexylcarbodiimide (1.33 g, 6.44 mmol) and the resulting mixture was stirred at room temperature for 30 minutes. The reaction mixture was filtered through Celite® and concentrated under reduced pressure to give the title compound as a colourless oil as a 88:12 mixture of anhydride : acid (1.30 g, 5.46 mmol, 47%) with data in accordance with the literature.[10] **^{1}H NMR** (400 MHz, $CDCl_3$) δ_H: 0.99 (12H, $^3J_{HH}$ = 6.7 Hz, CH(CH_3)$_2$), 2.25 – 2.37 (2H, m, C*H*(CH$_3$)$_2$), 3.16 (4H, dt, $^3J_{HH}$ = 6.7 Hz, $^4J_{HH}$ = 1.3 Hz, CH_2COO), 5.44 (2H, dtd, $^3J_{HH}$ = 15.4 Hz, 6.7 Hz, $^4J_{HH}$ = 1.3 Hz, CH=C*H*-CH$_2$), 5.59 (2H, ddt, $^3J_{HH}$ = 15.4 Hz, 6.5 Hz, CH=C*H*-CH(CH$_3$)$_2$); the compound was used without further purification.

2. Synthesis of Pyrazol-3-ones

2.1. 5-Methyl-2-phenyl-2,4-dihydro-3*H*-pyrazol-3-one **S12**

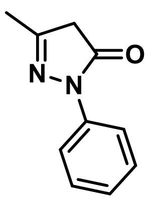

To ethyl acetoacetate (8.13 ml, 62.5 mmol) was slowly added phenylhydrazine (6.25 ml, 62.5 mmol). The mixture was stirred at 145 °C for 60 minutes to give the title compound as pale yellow solid (10.89 g, 62.5 mmol, 89%) with data in accordance with the literature.[16] **mp** 126-128 °C {Lit.[16] 125-128 °C}; **^{1}H NMR** (400 MHz, $CDCl_3$) δ_H: 2.21 (1H, s, CH_3), 3.44 (2H, q, $^4J_{HH}$ = 0.7 Hz, CH_2), 7.15-7.21 (1H, m, ArC^4*H*), 7.36-7.41 (2H, m, ArC^{3,5}*H*), 7.83-7.88 (2H, m, ArC^{2,6}*H*).

2.2. 5-Isopropyl-2-phenyl-2,4-dihydro-3*H*-pyrazol-3-one **S13**

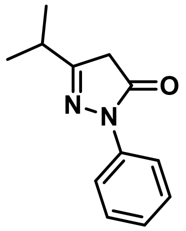

To ethyl 4-methyl-oxo-pentanoate (1.0 ml, 6.3 mmol) was slowly added phenylhydrazine (0.62 ml, 6.3 mmol). The mixture was heated at 145 °C for 60 minutes to give the title compound as yellow solid (797 mg, 3.9 mmol, 63%) with data in accordance with the literature.[17] **mp** 84-86 °C {Lit[18] 87 °C}; **^{1}H NMR** (500 MHz, $CDCl_3$) δ_H: 1.26 (6H, d, $^3J_{HH}$ = 6.9 Hz, CH(CH_3)$_2$), 2.80 (1H, hept, $^3J_{HH}$ = 6.9 Hz, C*H*(CH_3)$_2$), 3.43 (2H, s, CH_2), 7.14-7.21 (1H, m, ArC^4*H*), 7.35-7.43 (2H, m, ArC^{3,5}*H*), 7.83-7.93 (2H, m, ArC^{2,6}*H*).

2.3. 2,5-Diphenyl-2,4-dihydro-3*H*-pyrazol-3-one **S14**

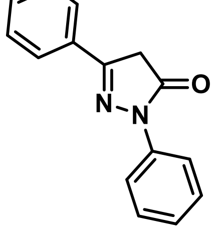

To ethyl 3-oxo-3-phenylpropanoate (0.9 ml, 5.2 mmol) was slowly added phenylhydrazine (0.51 ml, 5.2 mmol). The mixture was heated at 120 °C for 20 minutes to give the title compound as pale orange solid (985 mg, 4.2 mmol, 80%) with data in accordance with the

literature.[17] **mp** 134-136 °C {Lit.[17] 136-138 °C}; **^{1}H NMR** (400 MHz, $CDCl_3$) δ_H: 3.87 (2H, s, CH_2), 7.20-7.25 (1H, m, ArC*H*), 7.40-7.51 (5H, m, ArC*H*), 7.75-7.82 (2H, m, ArC*H*), 7.95-8.02 (2H, m, ArC*H*).

2.4. 2-(*tert*-Butyl)-5-methyl-2,4-dihydro-3*H*-pyrazol-3-one **S15**

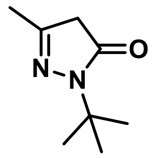

To a solution of ethyl acetoacetate (1.27 ml, 10.0 mmol) in EtOH (11.5 ml, 0.87 M), was added *tert*-butylhydrazine hydrochloride (2.49 g, 20.0 mmol) and sodium acetate (1.64 g, 20.0 mmol). The reaction mixture was refluxed overnight under a positive pressure of N_2. After cooling to room temperature, the mixture was diluted with water and extracted with CH_2Cl_2. The combined organic layers were dried over $MgSO_4$, filtered, and concentrated *in vacuo*. The resulting crude solid was suspended in Et_2O at −20 °C and filtered to give the title compound as a white solid (1.19 g, 7.7 mmol, 77%) with data in accordance with the literature.[19] **mp** 127-128 °C {Lit.[19] 125-127 °C}; **^{1}H NMR** (500 MHz, $CDCl_3$) δ_H: 1.49 (9H, s, C(CH_3)$_3$, 2.04 (3H, s, C^5CH_3), 3.17 (2H, s, CH_2).

2.5. 2-(*p*-Anisyl)-5-methyl-2,4-dihydro-3*H*-pyrazole-3-one **S16**

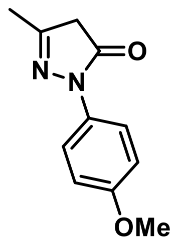

To *p*-anisylhydrazine hydrochloride (1.75 mg, 10.0 mmol) and triethylamine (2.1 ml, 15.0 mmol) in EtOH (30 ml, 0.5 M) was added ethyl acetoacetate (1.26 ml, 10.0 mmol). The mixture was heated at 60 °C overnight. The solvent was removed under reduced pressure. Further purification by column chromatography (petroleum ether : ethyl acetate 4:1 to 1:1) gave the title compound as pale yellow solid (1.34 g, 6.6 mmol, 66%) with data in accordance with the literature.[20] **mp** 130 °C (dec) (Hexane/EtOAc) {Lit.[21] 124-126 °C, 127-128 °C (Hexane:THF)}; **IR** ν_{max} (film) 2930 (C-H), 2357, 1508 (C=O), 1248, 1032, 831, 775; **^{1}H NMR** (500 MHz, $CDCl_3$) δ_H: 2.19 (3H, s, C^5CH_3), 3.41 (2H, s, CH_2), 3.81 (3H, s, OCH_3), 6.89-6.94 (2H, m, Ar$C^{3,5}$*H*), 7.69-7.75 (2H, m, Ar$C^{2,6}$*H*).

2.6. 5-Methyl-2-(4-(trifluoromethyl)phenyl)-2,4-dihydro-3*H*-pyrazol-3-one **S17**

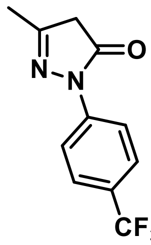

To *p*-(trifluoromethyl)phenylhydrazine hydrochloride (2.13 ml, 10.0 mmol) and triethylamine (2.1 ml, 15.0 mmol) in EtOH (30 ml, 0.5 M) was added ethyl acetoacetate (1.26 ml, 10.0 mmol). The mixture was heated at 60 °C overnight. The solvent was removed under reduced pressure. Further purification by column chromatography (petroleum ether : ethyl acetate 4:1 to 1:1) gave the title compound as pale yellow solid (871 mg, 3.6 mmol, 36%) with data in accordance with the literature.[22] **mp** 170 °C (dec) (Hexane:EtOAc) {Lit.[22] 183-185 °C}; **IR** ν_{max} (film) 2691 (O-H, enol), 2359, 1628 (C=O), 1607, 1327, 1109, 1072; **^{1}H NMR** (500 MHz, $CDCl_3$) δ_H: 2.22 (3H, s, CH_3), 3.47 (2H, s, CH_2), 7.64 (2H, app d, $^3J_{HH}$ = 8.6 Hz, ArC^{2,6}*H*), 8.05 (2H, app d, $^3J_{HH}$ = 8.6 Hz, ArC^{3,5}*H*).

3. Synthesis of Pyrazol-3-one-derived ketimines

3.1. 5-Methyl-2-phyenyl-4-(phenylimino)-2,4-dihydro-3*H*-pyrazol-3-one **S18**

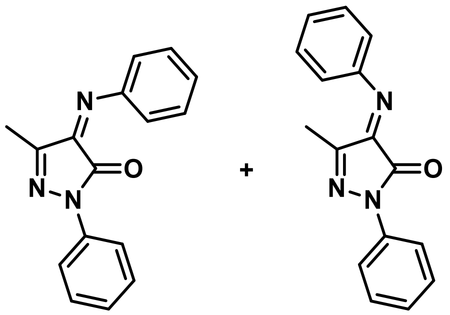

To a solution of 5-methyl-2-phenyl-2,4-dihydro-3*H*-pyrazol-3-one (8.50 g, 48.8 mmol) in MeOH (30 ml, 0.6 M) was added nitrosobenzene (5.22 g, 48.8 mmol) and K_2CO_3 (1.35 g, 9.8 mmol) and the resulting mixture was heated at reflux for 3 h. After cooling to room temperature, the solvent was removed under reduced pressure. Purification by column chromatography (petroleum ether : diethyl ether 1:0 to 3:1) gave the title compound as inseparable mixture of isomers as red solid (3.79 g, 14.4 mmol, 30%, 83:17 d.r.) with data in accordance with the literature.[23] **mp** 101-103 °C {Lit.[23] 101-103 °C}; **^{1}H NMR** (400 MHz, $CDCl_3$) (*major diastereomer*) δ_H: 2.35 (3H, s, CH_3), 7.17-7.23 (1H, m, ArC*H*), 7.28-7.48 (7H, m, ArC*H*), 7.84-

7.89 (2H, m, ArC*H*); **^{1}H NMR** (400 MHz, $CDCl_3$) (*minor diastereomer, selected*) δ_H: 1.80 (3H, s, CH_3), 6.94-6.99 (2H, m, ArC*H*), 7.92-7.96 (2H, m, ArC*H*).

3.2. 5-Isopropyl-2-phenyl-4-(phenylimino)-2,4-dihydro-3*H*-pyrazol-3-one **S19**

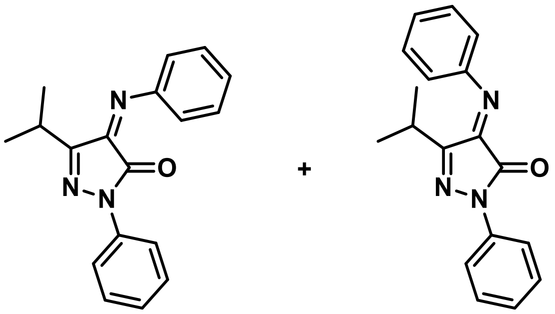

To a solution of 5-isopropyl-2-phenyl-2,4-dihydro-3*H*-pyrazol-3-one (2.50 g, 12.6 mmol) in MeOH (21 ml, 0.6 M) was added nitrosobenzene (1.35 g, 12.6 mmol) and K_2CO_3 (348 mg, 2.5 mmol) and the resulting mixture was heated at reflux for 3 h. After cooling to room temperature, the solvent was removed under reduced pressure. Purification by column chromatography (petroleum ether : diethyl ether 1:0 to 3:1) gave the title compound as an inseparable mixture of isomers as red solid (1.68 g, 5.8 mmol, 46%, 95:5 d.r.) with data in accordance with the literature.[23] **mp** 79-81 °C {Lit.[23] 77-79 °C}; **^{1}H NMR** (500 MHz, $CDCl_3$) (*major diastereomer*) δ_H: 1.41 (6H, d, $^3J_{HH}$ = 6.9 Hz, CH(CH_3)$_2$), 3.19 (1H, hept, $^3J_{HH}$ = 6.9 Hz, C*H*(CH_3)$_2$), 7.17-7.22 (1H, m, ArC*H*), 7.29-7.35 (3H, m, ArC*H*), 7.36-7.47 (4H, m, ArC*H*), 7.87-7.92 (2H, m, Ar*H*); **^{1}H NMR** (500 MHz, $CDCl_3$) (*minor diastereomer, selected*) δ_H: 0.98 (6H, d, $^3J_{HH}$ = 6.7 Hz, CH(CH_3)$_2$), 2.46 (1H, hept, $^3J_{HH}$ = 6.7 Hz, C*H*(CH_3)$_2$), 7.95-8.00 (2H, d, J_{HH} 8.0, ArC*H*).

3.3. 2,5-Diphenyl-4-(phenylimino)-2,4-dihydro-3*H*-pyrazol-3-one **S20**

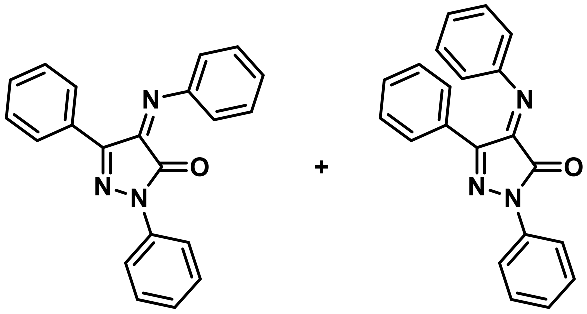

To a solution of 2,5-diphenyl-2,4-dihydro-3*H*-pyrazol-3-one (1.80 g, 7.6 mmol) in MeOH (4.5 ml, 0.6 M) was added nitrosobenzene (816 mg, 7.6 mmol) and K_2CO_3 (211 mg, 1.5 mmol) and the resulting mixture was heated at reflux for 3 h. After cooling to room temperature, the solvent was removed under reduced pressure. Purification by column chromatography

(petroleum ether : diethyl ether 1:0 to 3:1) gave the title compound as red solid (726 mg, 2.2 mmol, 29%) with data in accordance with the literature.[23] **mp** 178-181 °C {Lit.[23] 176-178 °C}; **^{1}H NMR** (500 MHz, $CDCl_3$) δ_H: 7.21-7.26 (1H, m, ArC*H*), 7.28-7.36 (3H, m, ArC*H*), 7.41-7.52 (7H, m, ArC*H*), 7.95-7.99 (2H, m, ArC*H*), 8.26-8.34 (2H, m, ArC*H*).

3.4. 2-(*tert*-Butyl)-5-methyl-4-(phenylimino)-2,4-dihydro-3*H*-pyrazol-3-one **S21**

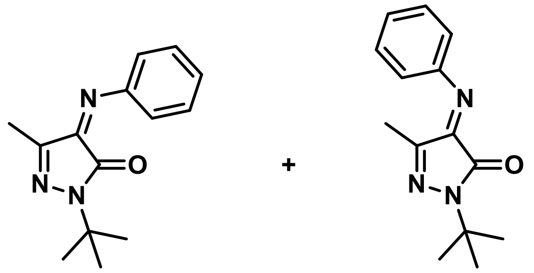

To a solution of 2-(*tert*-butyl)-5-methyl-2,4-dihydro-3*H*-pyrazol-3-one (1.00 g, 6.48 mmol) in MeOH (11 ml, 0.6 M) was added nitrosobenzene (695 g, 6.48 mmol) and K_2CO_3 (179 mg, 1.3 mmol) and the resulting mixture was heated at reflux for 3 h. After cooling to room temperature, the solvent was removed under reduced pressure. Purification by column chromatography (petroleum ether : diethyl ether 1:0 to 3:1) gave the title compound as an inseparable mixture of isomers as red solid (697 mg, 2.9 mmol, 44%, 75:25 d.r.) with data in accordance with the literature.[23] **mp** 60-62 °C; **IR** ν_{max} (film) 3062, 2978 (C-H), 2932 (C-H), 1703, 1699, 1364, 1279, 1217, 1099, 1022, 916, 795, 768; **HRMS** (ESI$^+$) $C_{14}H_{18}N_3O$ $[M+H]^+$ found 244.1439, requires 244.1444 (–2.3 ppm). *Data for major diastereomer*: **^{1}H NMR** (500 MHz, $CDCl_3$) δ_H: 1.49 (9H, s, C(C*H*$_3$)$_3$, 2.19 (3H, s C^{5}C*H*$_3$), 7.20-7.28 (3H, m, ArC^{2,4,6}*H*), 7.34-7.43 (2H, m, ArC^{3,5}*H*); **^{13}C{^{1}H} NMR** (126 MHz, $CDCl_3$) δ_C: 12.2 (C^{5}CH$_3$), 28.2 (C(*C*H$_3$)$_3$), 58.3 (*C*(CH$_3$)$_3$), 120.9 (Ar*C*^{2,6}H), 127.7 (Ar*C*^{4}H), 128.6 (Ar*C*^{3,5}H), 146.7 (Ar*C*^{1}), 147.8 (*C*^{5}CH$_3$), 154.1 (*C*^{4}=O), 154.7 (*C*^{3}=O); *Data for minor diastereomer*: **^{1}H NMR** (400 MHz, $CDCl_3$) (*selected*) δ_H: 1.56 (9H, s, C(C*H*$_3$)$_3$), 1.62 (3H, s, C^{5}C*H*$_3$), 6.93 (2H, d, $^3J_{HH}$ = 7.4 Hz, ArC^{2,6}*H*); **^{13}C{^{1}H} NMR** (126 MHz, $CDCl_3$) δ_C: 16.3 (C^{5}CH$_3$), 28.1 (C(*C*H$_3$)$_3$), 58.4 (*C*(CH$_3$)$_3$), 118.8 (Ar*C*^{2,6}H), 126.4 (Ar*C*^{4}H), 128.9 (Ar*C*^{3,5}H), 139.3 (*C*^{5}CH$_3$), 148.7 (Ar*C*^{1}), 152.8 (*C*^{4}(NPh)), 159.1 (*C*^{3}(O)N).

4. Synthesis of Pyrazole-4,5-diones

4.1. 3-Methyl-1-phenyl-1*H*-pyrazole-4,5-dione **S22**

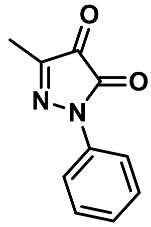

To a solution of 5-methyl-2-phenyl-4-(phenylimino)-2,4-dihydro-3*H*-pyrazol-3-one (3.56 g, 13.5 mmol) in THF (104 ml, 0.13 M) was added 2 M aq. HCl (13.5 ml, 27.0 mmol) and the resulting solution was stirred at room temperature until the reaction was complete by TLC. The reaction mixture was diluted with H_2O and extracted with CH_2Cl_2. The combined organic layers were concentrated *in vacuo.* Purification by column chromatography (petroleum ether : ethyl acetate 1:1) gave the title compound as red solid (2.01 g, 10.7 mmol, 81%).[24] **mp** 121-123 °C {Lit.[24] 119-121 °C}; **^{1}H NMR** (400 MHz, $CDCl_3$) δ_H: 2.24 (3H, s, CH_3), 7.24-7.33 (1H, m, ArC^4*H*), 7.41-7.51 (2H, m, ArC^{3,5}*H*), 7.84-7.92 (2H, m, ArC^{2,6}*H*).

4.2. 3-Isopropyl-1-phenyl-1*H*-pyrazole-4,5-dione **S23**

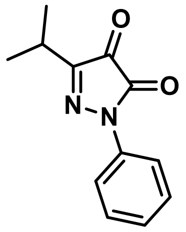

To a solution of 5-isopropyl-2-phenyl-4-(phenylimino)-2,4-dihydro-3*H*-pyrazol-3-one (1.65 g, 5.7 mmol) in THF (44 ml, 0.13 M) was added 2 M aq. HCl (5.7 ml, 11.4 mmol) and the resulting solution was stirred at room temperature until the reaction was complete by TLC. The reaction mixture was diluted with H_2O and extracted with CH_2Cl_2. The combined organic layers were concentrated *in vacuo.* Purification by column chromatography (petroleum ether : ethyl acetate 1:1) gave the title compound as red solid (1.13 g, 5.2 mmol, 92%).[24] **mp** 50-52 °C {Lit.[24] 51-53 °C}; **^{1}H NMR** (500 MHz, $CDCl_3$) δ_H: 1.34 (6H, d, $^3J_{HH}$ = 6.9 Hz, CH(CH_3)$_2$), 2.96 (1H, hept, $^3J_{HH}$ = 6.9 Hz, C*H*(CH_3)$_2$), 7.25-7.30 (1H, m, ArC^4*H*), 7.43-7.49 (2H, m, ArC^{3,5}*H*), 7.88-7.92 (2H, m, ArC^{2,6}*H*).

4.3. 1,3-Diphenyl-1*H*-pyrazole-4,5-dione **S24**

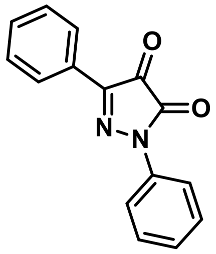

To a solution of 2,5-diphenyl-4-(phenylimino)-2,4-dihydro-3*H*-pyrazol-3-one (725 mg, 2.2 mmol) in THF (17 ml, 0.13 M) was added 2 M aq. HCl (2.2 ml, 4.4 mmol) and the resulting solution was stirred at room temperature until the reaction was complete by TLC. The reaction mixture was diluted with H_2O and extracted with CH_2Cl_2. The combined organic layers were concentrated *in vacuo.* Purification by column chromatography (petroleum ether : ethyl acetate 1:1) gave the title compound as red solid (558 mg, 2.2 mmol, 100%).[24] **mp** 161-163 °C {Lit.[24] 165-166 °C}; **^{1}H NMR** (500 MHz, $CDCl_3$) δ_H: 7.29-7.35 (1H, m, ArC^4*H*), 7.47-7.57 (5H, m, ArC*H*), 7.97-8.02 (2H, m, ArC*H*), 8.17-8.23 (2H, m, ArC*H*).

4.4. 1-(*tert*-Butyl)-3-methyl-1*H*-pyrazole-4,5-dione **S25**

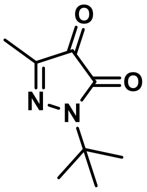

To a solution of 1-(*tert*-butyl)-5-methyl-4-(phenylimino)-2,4-dihydro-3*H*-pyrazol-3-one (600 mg, 2.4 mmol) in THF (19 ml, 0.13 M) was added 2 M aq. HCl (2.4 ml, 4.8 mmol) and the resulting solution was stirred at room temperature until the reaction was complete by TLC. The reaction mixture was diluted with H_2O and extracted with CH_2Cl_2. The combined organic layers were concentrated *in vacuo.* Purification by column chromatography (petroleum ether : ethyl acetate 1:1) gave the title compound as red oil (386 g, 2.3 mmol, 93%).[25] **IR** ν_{max} (film) 1306, 2980 (C-H), 2934 (C-H), 2359, 1757, 1724 (C=O), 1701, 1368, 1026; **^{1}H NMR** (500 MHz, $CDCl_3$) δ_H: 1.52 (9H, s, C(C*H*$_3$)$_3$), 2.06 (3H, s, C^{3}C*H*$_3$); **^{13}C{^{1}H} NMR** (126 MHz, $CDCl_3$) δ_C: 11.1 (C^3*C*H$_3$), 27.9 (C(*C*H$_3$)$_3$, 59.3 (*C*(CH$_3$)$_3$), 142.0 (*C*^{3}CH$_3$), 150.9 (*C*^{5}(O)N), 186.4 (*C*^{4}(O)); **HRMS** (ESI$^+$) $C_8H_{13}N_2O_2$ [M+H]$^+$ found 169.0970, requires 169.0972 (–0.9 ppm).

4.5. 1-(*p*-Anisyl)-3-methyl-1*H*-pyrazole-4,5-dione **S26**

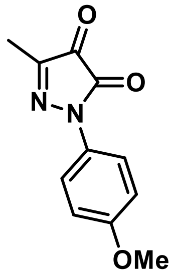

To a solution of 2-(*p*-anisyl)-5-methyl-2,4-dihydro-3*H*-pyrazol-3-one (1.40 g, 6.7 mmol) in MeOH (11 ml, 0.6 M) was added nitrosobenzene (735 mg, 6.7 mmol) and K_2CO_3 (190 mg, 1.4 mmol) and the resulting mixture was heated at reflux for 3 h. After cooling to room temperature, the solvent was removed under reduced pressure. Purification by column chromatography (petroleum ether : diethyl ether 1:0 to 3:1) gave the title compound as red solid (275 mg, 0.9 mmol, 14%) which was used directly without further purification. **^{1}H NMR** (500 MHz, $CDCl_3$) δ_H: 2.33 (3H, s, C^5CH_3), 3.81 (3H, s, OCH_3), 6.90-6.93 (2H, m, $N^2ArC^{3,5}H$), 7.30-7.34 (1H, m, C^4=$NArC^4H$), 7.24-7.38 (2H, m, C^4=$NArC^{2,6}H$), 7.40-7.44 (2H, m, C^4=$NArC^{3,5}H$), 7.73-7.78 (2H, m, $N^2ArC^{2,6}H$).

To a solution of crude 2-(*p*-anisyl)-5-methyl-4-(phenylimino)-2,4-dihydro-3*H*-pyrazol-3-one (275 mg, 0.9 mmol) in THF (7 ml, 0.13 M) was added 2 M aq. HCl (0.9 ml, 1.8 mmol) and the resulting solution was stirred at room temperature until the reaction was complete by TLC. The reaction mixture was diluted with H_2O and extracted with CH_2Cl_2. The combined organic layers were concentrated *in vacuo.* Purification by column chromatography (petroleum ether : ethyl acetate 1:1) gave the title compound as red solid (271 mg, 1.2 mmol, 76%). **mp** 121-123 °C; **IR** ν_{max} (film) 3327, 1771, 1699 (C=O, amide), 1514 (C=O, ketone), 1250, 831; **^{1}H NMR** (500 MHz, $CDCl_3$) δ_H: 2.21 (3H, s, C^3CH_3), 3.84 (3H, s, OCH_3), 6.94-7.00 (2H, m, $ArC^{3,5}H$), 7.73-7.79 (2H, m, $ArC^{2,6}H$); **^{13}C{^{1}H} NMR** (126 MHz, $CDCl_3$) δ_C: 11.2 (C^5CH_3), 55.7 (OCH_3), 114.4 ($ArC^{3,5}H$), 119.8 ($ArC^{2,6}H$), 130.3 (ArC^1), 144.3 (C^3CH_3), 148.9 (C^5=O), 158.0 (ArC^4OCH_3), 185.1 (C^4=O); **HRMS** (ESI^+) $C_{11}H_{11}N_2O_3$ $[M+H]^+$ found 219.0765, requires 219.0764 (+0.3 ppm).

4.6. 3-Methyl-1-(*p*-(trifluoromethyl)phenyl)-1*H*-pyrazole-4,5-dione **S27**

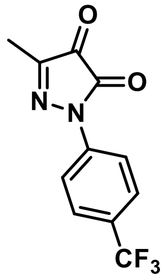

To a solution of 5-methyl-2-(*p*-trifluoromethyl)phenyl-2,4-dihydro-3*H*-pyrazol-3-one (712 mg, 2.9 mmol) in MeOH (5 ml, 0.6 M) was added nitrosobenzene (315 mg, 2.9 mmol) and K_2CO_3 (81 mg, 0.6 mmol) and the resulting mixture was heated at reflux for 3 h. After cooling to room temperature, the solvent was removed under reduced pressure. Purification by column chromatography (petroleum ether : diethyl ether 1:0 to 3:1) gave the title compound as red solid (195 mg, 0.6 mmol, 20%) which was used directly without further purification. **^{1}H NMR** (500 MHz, $CDCl_3$) δ_H: 2.37 (3H, s, C*H*$_3$), 7.33-7.39 (3H, m, C^4=NArC^{3,4,5}*H*), 7.42-7.46 (2H, m, C^4=NArC^{2,6}*H*), 7.64 (2H, d, $^3J_{HH}$ = 8.6 Hz, N^2ArC^{2,6}*H*), 8.05 (2H, d, $^3J_{HH}$ = 8.6 Hz, N^2ArC^{3,5}*H*).

To a solution of crude 5-methyl-2-(*p*-trifluoromethyl)phenyl-4-(phenylimino)-2,4-dihydro-3*H*-pyrazol-3-one (195 mg, 0.6 mmol) in THF (4.5 ml, 0.13 M) was added 2 M aq. HCl (0.6 ml, 1.2 mmol) and the resulting solution was stirred at room temperature until the reaction was complete by TLC. The reaction mixture was diluted with H_2O and extracted with CH_2Cl_2. The combined organic layers were concentrated *in vacuo.* Purification by column chromatography (petroleum ether : ethyl acetate 1:1) gave the title compound as red solid (90 mg, 0.35 mmol, 59%) with data in accordance with the literature.[25] **mp** 110 °C (dec) (Hexane:EtOAc); **^{1}H NMR** (500 MHz, $CDCl_3$) δ_H: 2.27 (3H, s, C*H*$_3$), 7.72 (2H, d, $^3J_{HH}$ = 8.6 Hz, ArC^{2,6}*H*), 8.06 (2H, d, $^3J_{HH}$ = 8.6 Hz, ArC^{3,5}*H*); **^{19}F{^{1}H} NMR** (377 MHz, $CDCl_3$) δ_F: −62.3 (C*F*$_3$).

5. Isothiourea-catalysed formal [2+2] cycloaddition of homoanhydrides and pyrazole-4,5-diones

5.1. *N*-Benzyl (2'*R*,4*R*)-2-(4-hydroxy-3-methyl-5-oxo-1-phenyl-4,5-dihydro-1*H*-pyrazol-4-yl)-2-phenylacetamide **9**

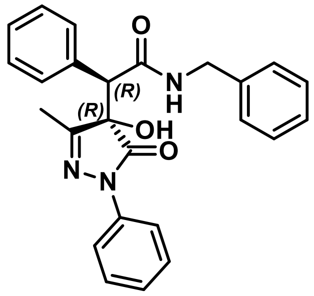

To a solution of 3-methyl-1-phenyl-1*H*-pyrazole-4,5-dione (47.1 mg, 0.25 mmol), 2-phenylacetic anhydride (95.4 mg, 0.375 mmol) and (2*R*,3*S*)-HyperBTM (3.9 mg, 0.125 mmol) were dissolved in ethyl acetate (6 ml, 0.04 M) at 0 °C. Diisopropylethylamine (54 µl, 0.31 mmol) was added and the reaction mixture was stirred at 0 °C for 3 h. Benzylamine (82 µl, 0.75 mmol) was added and the reaction mixture was stirred for 16 h at rt. The reaction mixture was diluted with ethyl acetate and washed sequentially with 1 M aq. HCl (×2), sat. aq. $NaHCO_3$ (×2), and brine (×1). The organic layer was dried over $MgSO_4$, filtered, and concentrated *in vacuo*. Purification by column chromatography (*n*-hexane : ethyl acetate 4:1 to 3:4) gave the title compound as single diastereomer as a white amorphous solid (62.2 mg, 0.15 mmol, 60%). $[\alpha]_D^{20}$ +190.7 (*c* 0.90, $CHCl_3$); **HPLC analysis**: Chiralpak AD-H (90:10 hexane:isopropanol, flow rate 1 ml·min^{-1}, 211 nm, 30 °C), t_R (2'*R*,4*R*)-**XX**: 26.4 min, t_R (2'*S*,4*S*)-**XX**: 31.2 min, >99:1 er; **IR** ν_{max} (film) 3316 (O-H), 3063 (C-H), 3032 (C-H), 2024 (C-H), 1717 (C=O, pyrazolone), 1645, 1595, 1499, 1362, 1265, 1128, 750; **^{1}H NMR** (500 MHz, $CDCl_3$) δ_H: 2.10 (3H, s, CH_3), 4.05 (1H, s, C*H*CONHBn), 4.46 (1H, dd, $^2J_{HH}$ = 17.8 Hz, $^3J_{HH}$ = 6.0 Hz, NHCH_AH$_B$Ph), 4.49 (1H, dd, $^2J_{HH}$ = 17.8 Hz, $^3J_{HH}$ = 5.8 Hz, NHCH$_A$$H_B$Ph), 5.97 (1H, app t, $^3J_{HH}$ = 5.9 Hz, N*H*), 7.04 (1H, s, O*H*), 7.11 (1H, app t, $^3J_{HH}$ = 7.4 Hz, NArC^4*H*), 7.21 (2H, app d, $^3J_{HH}$ = 7.1 Hz, CH$_2$ArC^{2,6}*H*), 7.23-7.35 (10H, m, CHArC^{2,3,4,5,6}*H*, NArC^{3,5}*H*, CH$_2$ArC^{3,4,5}*H*), 7.49 (2H, d, $^3J_{HH}$ = 8.0 Hz, NArC^{2,6}*H*); **^{13}C{^{1}H} NMR** (126 MHz, $CDCl_3$) δ_C: 15.4 (*C*H$_3$), 43.9 (*C*H$_2$Ph), 54.9 (*C*HCONHBn), 82.3 (*C*-OH), 119.3 (NAr*C*^{2,6}H), 125.5 (NAr*C*^{4}H), 127.8 (CH$_2$Ar*C*^{2,6}H), 128.0 (Ar*C*^{4}H), 128.8 (NAr*C*^{3,5}H), 129.0 (Ar*C*^{3,5}H), 129.3 (Ar*C*^{3,5}H), 129.4 (Ar*C*^{4}H), 129.8 (CHAr*C*^{2,6}H), 131.4 (CHAr*C*^{1}), 137.1 (CH$_2$Ar*C*^{1}), 137.2 (NAr*C*^{1}), 160.2 (*C*=N), 170.6 (C(OH)*C*=O), 172.6 (*C*ONHBn); **HRMS** (ESI$^+$) $C_{25}H_{22}N_3O_3Na$ [M+Na]$^+$ found 436.16206, requires 436.16316 (–0.3 ppm).

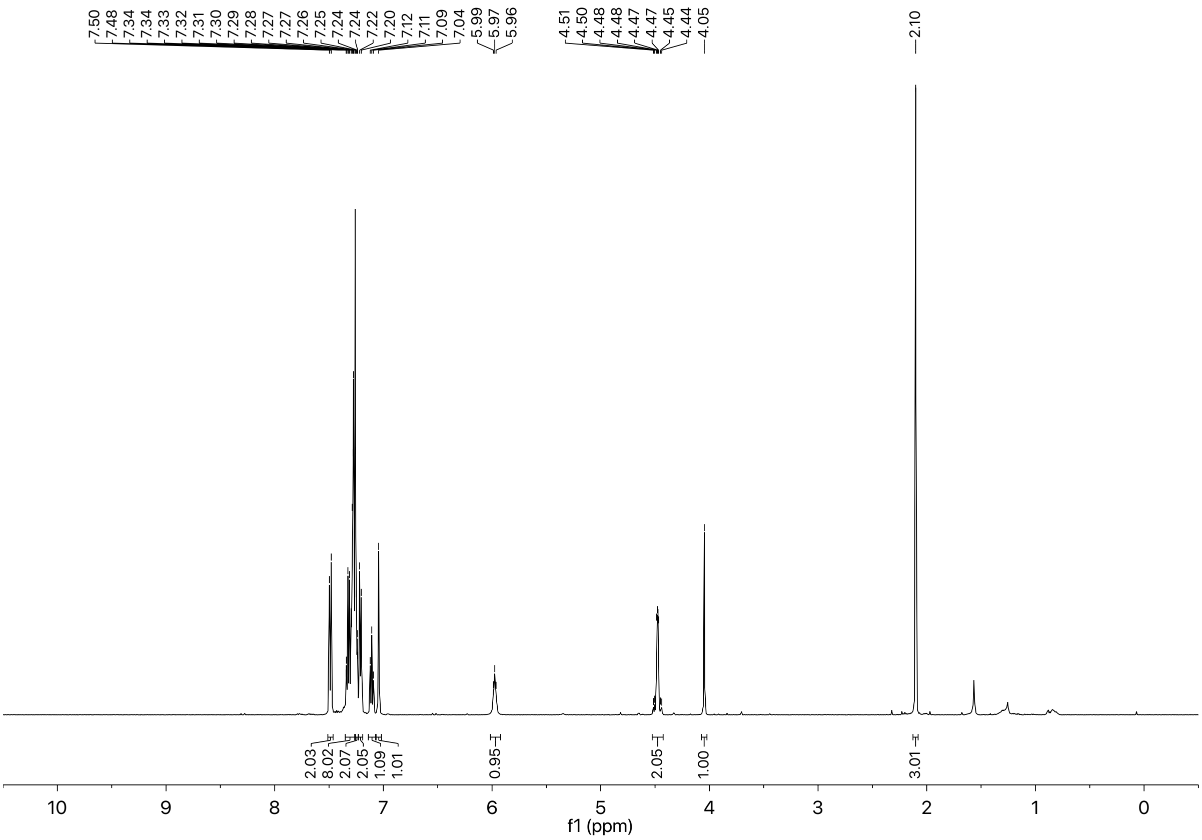
7.50
7.48
7.34
7.34
7.33
7.32
7.31
7.30
7.29
7.28
7.27
7.27
7.26
7.25
7.24
7.24
7.22
7.20
7.12
7.11
7.09
7.04
5.99
5.97
5.96
4.51
4.50
4.48
4.48
4.47
4.47
4.45
4.44
4.05
2.10
2.03
8.02
2.07
2.05
1.09
1.01
0.95
2.05
1.00
3.01
10
9
8
7
6
5
4
3
2
1
0
f1 (ppm)

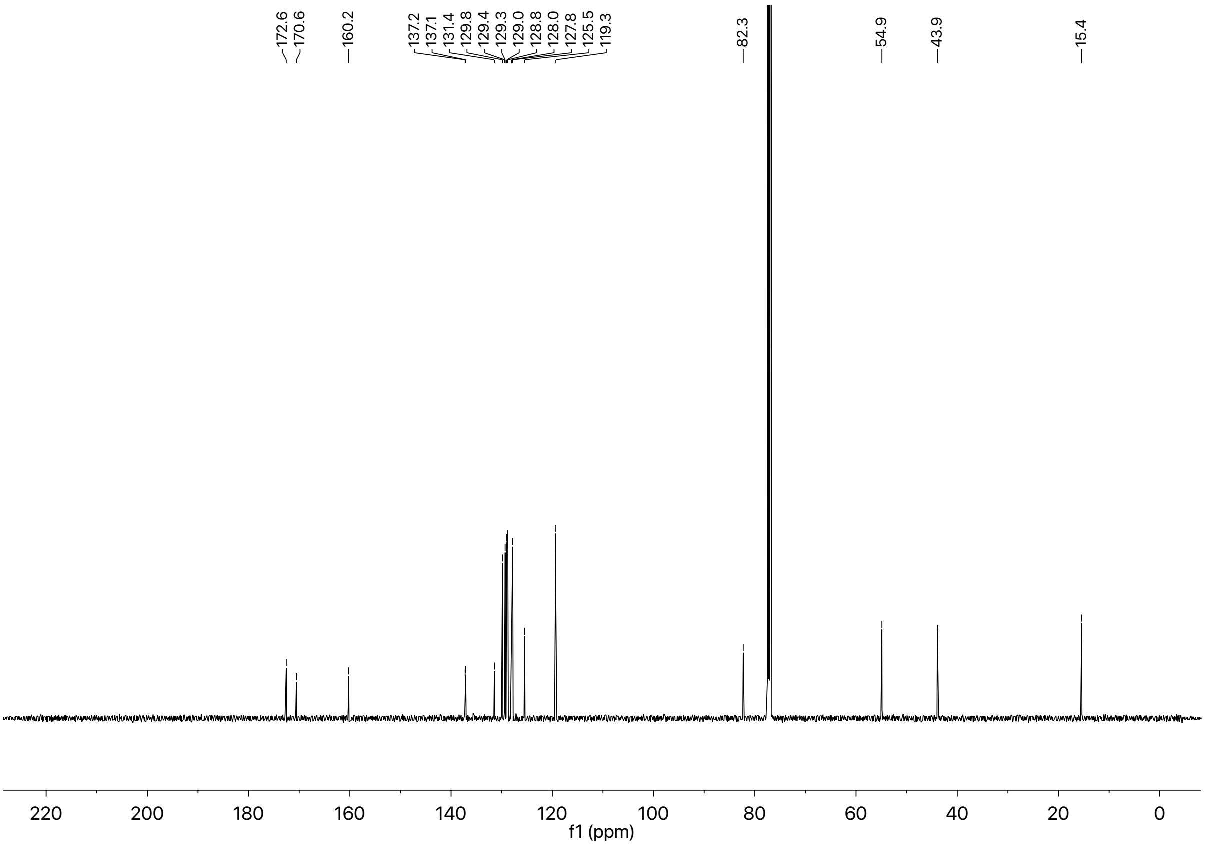
172.6
170.6
160.2
137.2
137.1
131.4
129.8
129.4
129.3
129.0
128.8
128.0
127.8
125.5
119.3
82.3
54.9
43.9
15.4
220
200
180
160
140
120
100
80
60
40
20
0
f1 (ppm)

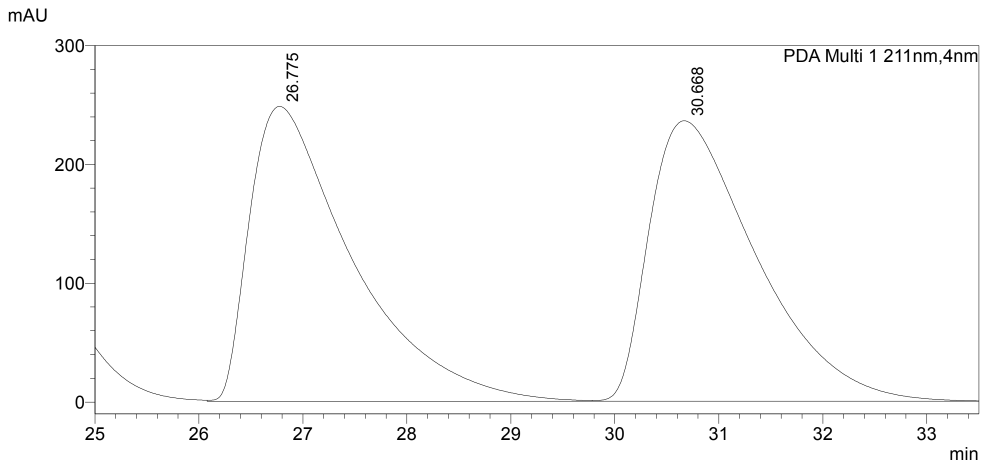

<Peak Table>

PDA Ch1 211nm

Peak#	Ret. Time	Area%
1	26.775	50.225
2	30.668	49.775
Total		100.000

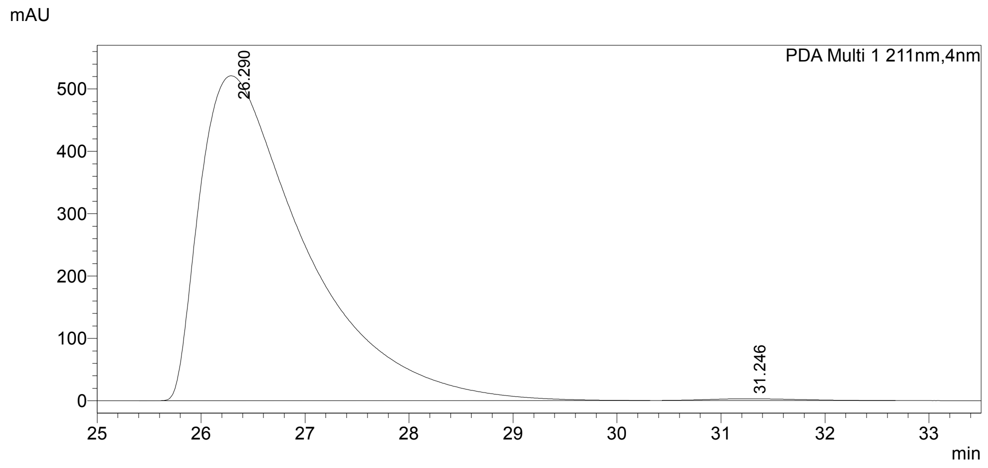

<Peak Table>

PDA Ch1 211nm

Peak#	Ret. Time	Area%
1	26.290	99.545
2	31.246	0.455
Total		100.000

5.2. (1′*R*,4*R*)- and (1′*S*,4*R*)-4-hydroxy-5-methyl-4-(2-oxo-1-phenyl-2-(pyrrolidine-1-yl)ethyl)-1-phenyl-2,4-dihydro-3*H*-pyrazol-3-one **10**

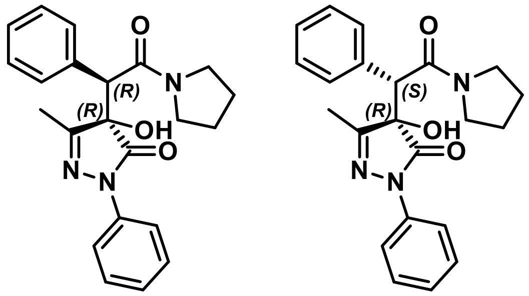

To a solution of 3-methyl-1-phenyl-1*H*-pyrazole-4,5-dione (47.1 mg, 0.25 mmol), 2-phenylacetic anhydride (95.4 mg, 0.375 mmol) and (2*R*,3*S*)-HyperBTM (3.9 mg, 0.125 mmol) were dissolved in ethyl acetate (6 ml, 0.04 M) at 0 °C. Diisopropylethylamine (54 µl, 0.31 mmol) was added and the reaction mixture was stirred at 0 °C for 3 h. Pyrrolidine (63 µl, 0.75 mmol) was added and the reaction mixture was stirred for 16 h at rt. The reaction mixture was diluted with ethyl acetate and washed sequentially with 1 M aq. HCl (×2), sat. aq. $NaHCO_3$ (×2), and brine (×1). The organic layer was dried over $MgSO_4$, filtered, and concentrated *in vacuo*. Purification by column chromatography (*n*-hexane : ethyl acetate 4:1 to 3:4) gave the title compound as a mixture of diastereomer as a white amorphous solid (72.0 mg, 0.19 mmol, 76%, 89:11 d.r.). $[\alpha]_D^{20}$ +254.3 (*c* 1.02, $CHCl_3$); **IR** ν_{max} (film) 3306 (O-H), 2974 (C-H), 2926 (C-H), 2878 (C-H), 1717 (C=O, pyrazolone), 1622, 1597, 1501, 1443, 1364, 912, 756; **HRMS** (ESI^+) $C_{22}H_{23}N_3O_3$ $[M+H]^+$ found 378.18122, requires 378.18020 (–2.7 ppm). *Data for major diastereomer*: **HPLC Analysis:** Chiralpak AD-H (95:5 hexane:isopropanol, flow rate 2 ml·min^{-1}, 211 nm, 30 °C), t_R (1′*R*,4*R*)-**10**: 18.1 min, t_R (1′*S*,4*S*)-**10**: 21.3 min, >99:1 er; **^{1}H NMR** (500 MHz, $CDCl_3$) δ_H: 1.74-1.91 (7H, m, CH_3, N(CH_2CH_2)$_2$), 2.99 (1H, app dt, $^2J_{HH}$ = 10.3 Hz, $^3J_{HH}$ = 6.3 Hz, NCH_AH_BCH$_2$), 3.38 (1H, app dt, $^2J_{HH}$ = 10.6 Hz, $^3J_{HH}$ = 6.4 Hz, NCH_AH_BCH$_2$), 3.45 (1H, app dt, $^2J_{HH}$ = 12.9 Hz, $^3J_{HH}$ = 6.6 Hz, NCH_CH_DCH$_2$), 3.67 (1H, app dt, $^2J_{HH}$ = 12.8 Hz, $^3J_{HH}$ = 6.5 Hz, NCH_CH_DCH$_2$), 3.99 (1H, s, C*H*CON(CH_2CH_2)$_2$), 7.07 (1H, br s, O*H*), 7.17 (1H, app t, $^3J_{HH}$ = 7.4 Hz, NArC^4*H*), 7.30-7.39 (7H, m, NArC^{3,5}H, CHArC^{2,3,4,5,6}*H*), 7.74 (2H, app d, $^3J_{HH}$ = 8.0 Hz, NArC^{2,6}*H*); **^{13}C{^{1}H} NMR** (126 MHz, $CDCl_3$) δ_C: 15.4 (*C*H_3), 24.1 (NCH$_2$*C*H$_C$H$_D$), 26.0 (NCH$_2$*C*H$_A$H$_B$), 46.4 (N*C*H$_C$H$_D$CH$_2$), 46.9 (N*C*H$_A$H$_B$CH$_2$), 53.2 (*C*HCON(CH_2H_2)$_2$), 81.9 (*C*-OH), 119.1 (NAr*C*^{2,6}H), 125.3 (NAr*C*^{4}H), 128.8 (CHAr*C*^{4}H), 128.9 (Ar*C*H), 129.2 (Ar*C*H), 129.2 (Ar*C*H), 132.2 (CHAr*C*^{1}), 137.7 (NAr*C*^{1}), 160.9 (*C*=N), 170.2 (*C*ON(CH_2CH_2)$_2$), 172.2 (*C*ONAr); *Data for minor diastereomer*: **^{1}H NMR** (500 MHz, $CDCl_3$) (*selected*) δ_H: 2.13 (3H, s, CH_3), 3.54 (1H, app dt, $^2J_{HH}$ = 12.7 Hz, $^3J_{HH}$ = 6.4 Hz, NCH_AH_BCH$_2$), 3.85 (1H, s, C*H*CON(CH_2CH_2)$_2$); **^{13}C{^{1}H} NMR** (126 MHz, $CDCl_3$) (*selected*) δ_C: 14.7 (*C*H_3), 53.0

(*C*H), 80.9 (*C*-OH), 118.9 (NAr$C^{2,6}$H), 125.0 (NArC^{4}H), 128.6 (Ar*C*H), 130.0 (Ar*C*H), 137.9 (NArC^{1}).

The minor diastereomer could not be resolved on HPLC.

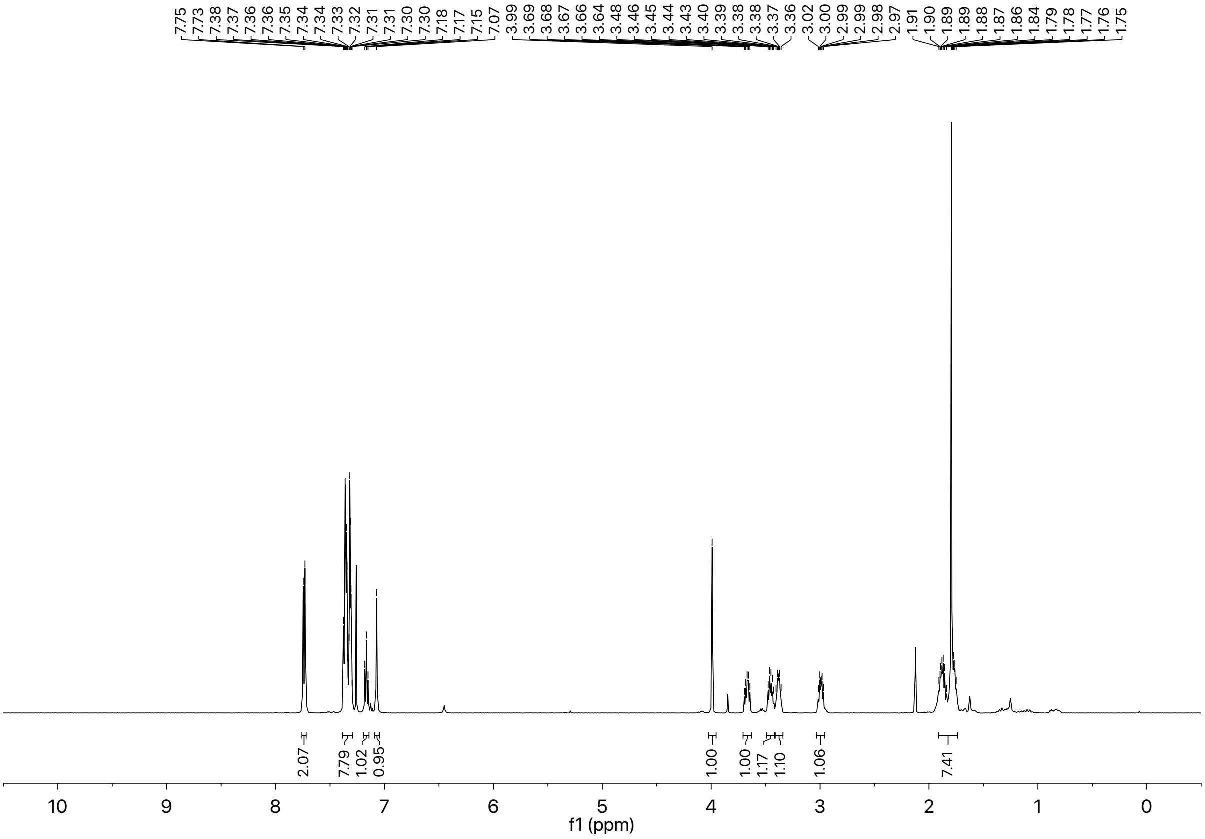

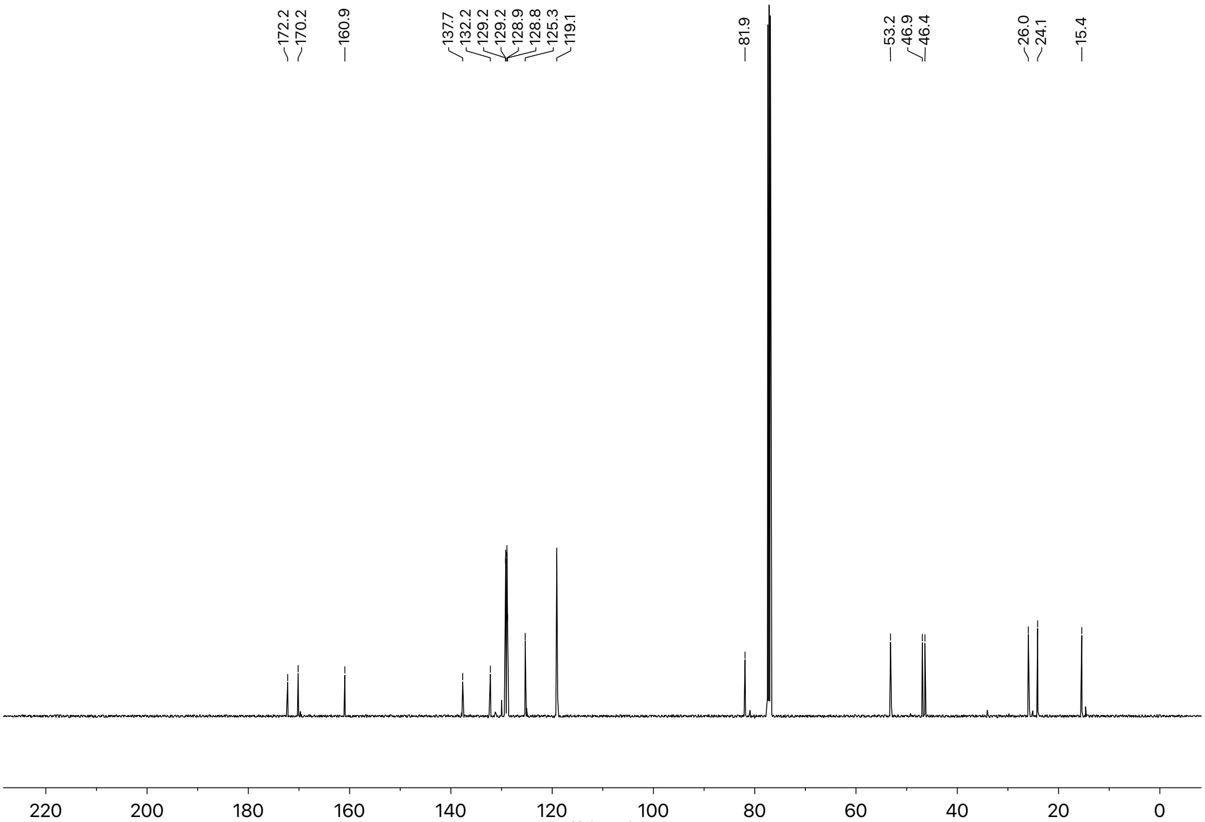

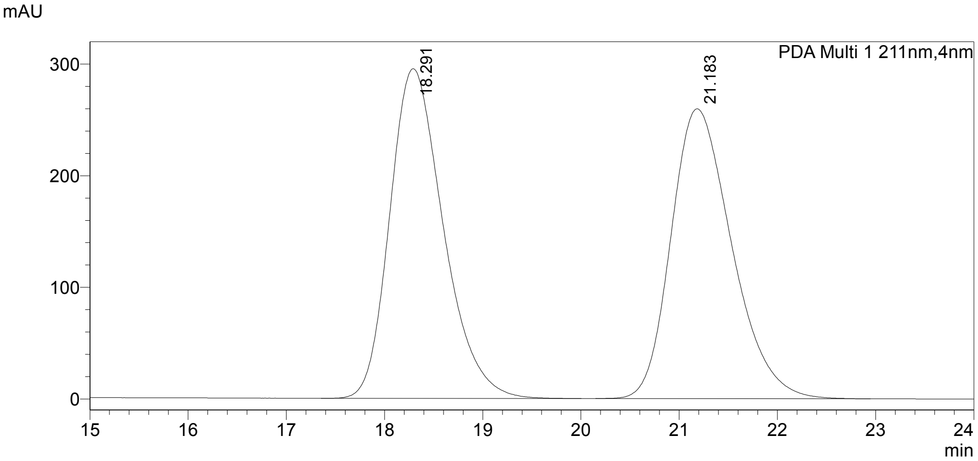

<Peak Table>

PDA Ch1 211nm

Peak#	Ret. Time	Area%
1	18.291	50.084
2	21.183	49.916
Total		100.000

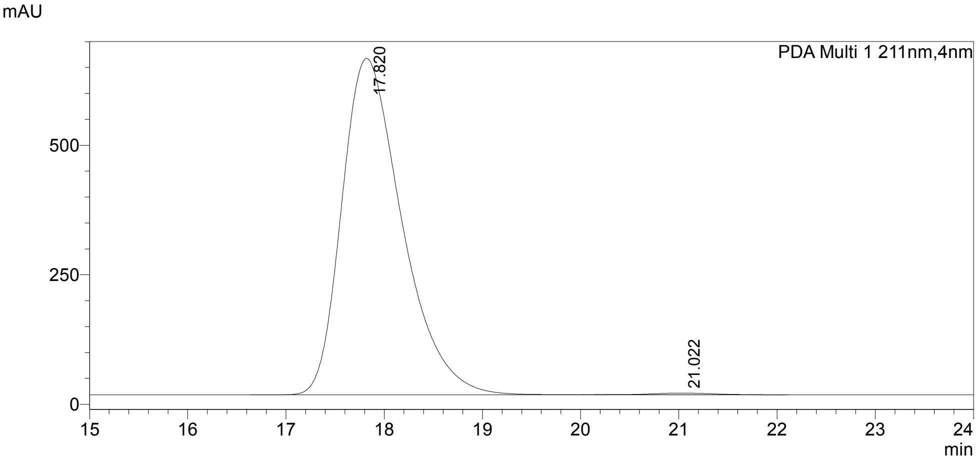

<Peak Table>

PDA Ch1 211nm

Peak#	Ret. Time	Area%
1	17.820	99.486
2	21.022	0.514
Total		100.000

5.3. (1′*R*,4*R*)- and (1′*S*,4*R*)-4-Hydroxy-5-methyl-4-(2-mopholino-2-oxo-1-phenylethyl)-2-phenyl-2,4-dihydro-3*H*-pyrazol-3-one **11**

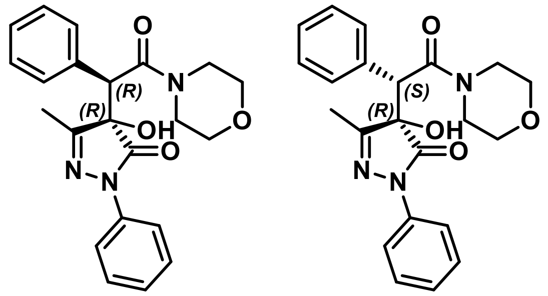

To a solution of 3-methyl-1-phenyl-1*H*-pyrazole-4,5-dione (47.1 mg, 0.25 mmol), 2-phenylacetic anhydride (95.4 mg, 0.375 mmol) and (2*R*,3*S*)-HyperBTM (3.9 mg, 0.125 mmol) were dissolved in ethyl acetate (6 ml, 0.04 M) at 0 °C. Diisopropylethylamine (54 µl, 0.31 mmol) was added and the reaction mixture was stirred at 0 °C for 3 h. Morpholine (66 µl, 0.75 mmol) was added and the reaction mixture was stirred for 16 h at rt. The reaction mixture was diluted with ethyl acetate and washed sequentially with 1 M aq. HCl (×2), sat. aq. $NaHCO_3$ (×2), and brine (×1). The organic layer was dried over $MgSO_4$, filtered, and concentrated *in vacuo*. Purification by column chromatography (*n*-hexane : ethyl acetate 4:1 to 3:4) gave the title compound as a mixture of diastereomer as a white amorphous solid (78.1 mg, 0.20 mmol, 79%, 92:8 d.r.). $[\alpha]_D^{20}$ +247.8 (*c* 1.00, $CDCl_3$); **IR** ν_{max} (film) 3356 (O-H), 2967 (C-H), 2924 (C-H), 2857 (C-H), 1717 (C=O, pyrazolone), 1639, 1622, 1597, 1501, 1115, 754; **HRMS** (ESI$^+$) $C_{22}H_{23}N_3O_4$ $[M+H]^+$ found 394.17540, requires 394.17613 (–0.2 ppm). *Data for major diastereomer*: **HPLC Analysis:** Chiralpak AD-H (85:15 hexane:isopropanol, flow rate 2.0 ml·min^{-1}, 211 nm, 30 °C) t_R (1′*R*,4*R*)-**11**: 8.0 min, t_R (1′*S*,4*S*)-**11**: 9.6 min, >99:1 er; **^{1}H NMR** (500 MHz, $CDCl_3$) δ_H: 1.95 (3H, s, C*H*$_3$), 3.07-3.19 (2H, m, $NCH_AH_BCH_2$, $NCH_2CH_AH_B$), 3.33 (1H, ddd, $^2J_{HH}$ = 14.7 Hz, $^3J_{HH}$ = 7.7 Hz, 4.2 Hz, $NCH_AH_BCH_2$), 3.45-3.51 (1H, m, $NCH_2CH_AH_B$), 3.54-3.68 (2H, m, $NCH_CH_DCH_2$, $NCH_2CH_CH_D$), 3.69-3.78 (2H, m, $NCH_CH_DCH_2$, $NCH_2CH_CH_D$), 4.22 (1H, s, C*H*Ph), 6.36 (1H, br s, O*H*), 7.17 (1H, app t, $^3J_{HH}$ = 7.4 Hz, NArC^4*H*), 7.24-7.28 (2H, m, CHArC^{3,5}*H*), 7.28-7.32 (3H, m, CHArC^{2,4,6}*H*), 7.35 (2H, app t, $^3J_{HH}$ = 7.9 Hz, NArC^{3,5}*H*), 7.70 (2H, app d, $^3J_{HH}$ = 8.0 Hz, NArC^{2,6}*H*); **^{13}C{^{1}H} NMR** (126 MHz, $CDCl_3$) δ_C: 15.7 (*C*H$_3$), 42.5 ($NCH_CH_DCH_2$), 46.6 ($NCH_AH_BCH_2$), 52.5 (*C*HPh), 66.1 ($NCH_2CH_AH_B$), 66.6 ($NCH_2CH_CH_D$), 81.7 (*C*-OH), 119.1 (NAr*C*^{2,6}H), 125.4 (NAr*C*^{4}H), 128.7 (CHAr*C*^{3,5}H), 128.9 (NAr*C*^{3,5}H), 129.0 (CHAr*C*^{4}H), 129.4 (CHAr*C*^{2,6}H), 132.0 (CHAr*C*^{1}), 137.5 (NAr*C*^{1}), 160.8 (*C*=N), 170.2 (*C*ON(CH_2CH_2)$_2$O), 171.7 (*C*ONAr); *Data for minor diastereomer*: **HPLC Analysis:** Chiralpak AD-H (85:15 hexane:isopropanol, flow rate 2.0 ml·min^{-1}, 211 nm, 30 °C) t_R (1′*S*,4*R*)-**11**: 6.9 min, t_R (1′*R*,4*S*)-**11**: 13.1 min, >99:1 er; **^{1}H NMR** (500 MHz, $CDCl_3$) (*selected*) δ_H: 2.00 (3H, s, C*H*$_3$), 3.02 (1H, ddd, $^2J_{HH}$ = 10.5 Hz, $^3J_{HH}$ = 5.1 Hz, 2.7 Hz, $NCH_2CH_CH_D$), 4.16 (1H, s, C*H*Ph), 5.36 (1H, br s, O*H*); **^{13}C{^{1}H} NMR** (126 MHz, $CDCl_3$) (*selected*)

δ_C: 14.8 (*C*H$_3$), 42.3 (N*C*H$_2$CH$_2$), 46.5 (N*C*H$_2$CH$_2$), 52.9 (*C*HPh), 80.3 (*C*-OH), 118.9 (NAr*C*^{2,6}H), 125.1 (NAr*C*^{4}H), 128.8 (Ar*C*H), 129.7 (Ar*C*H), 131.8 (CHAr*C*^{1}), 137.8 (NAr*C*^{1}), 160.3 (*C*=N), 169.2 (*C*ON(CH$_2$CH$_2$)$_2$O), 171.1 (*C*ONAr).

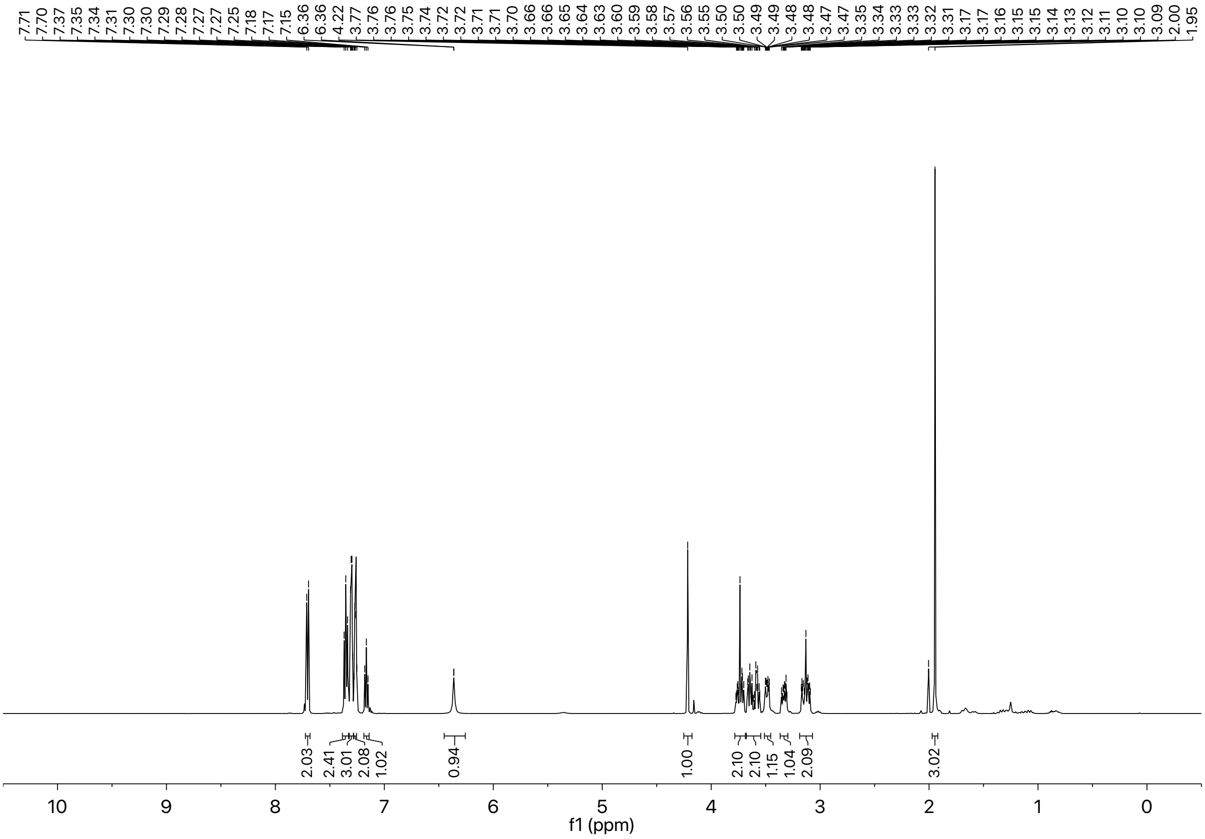

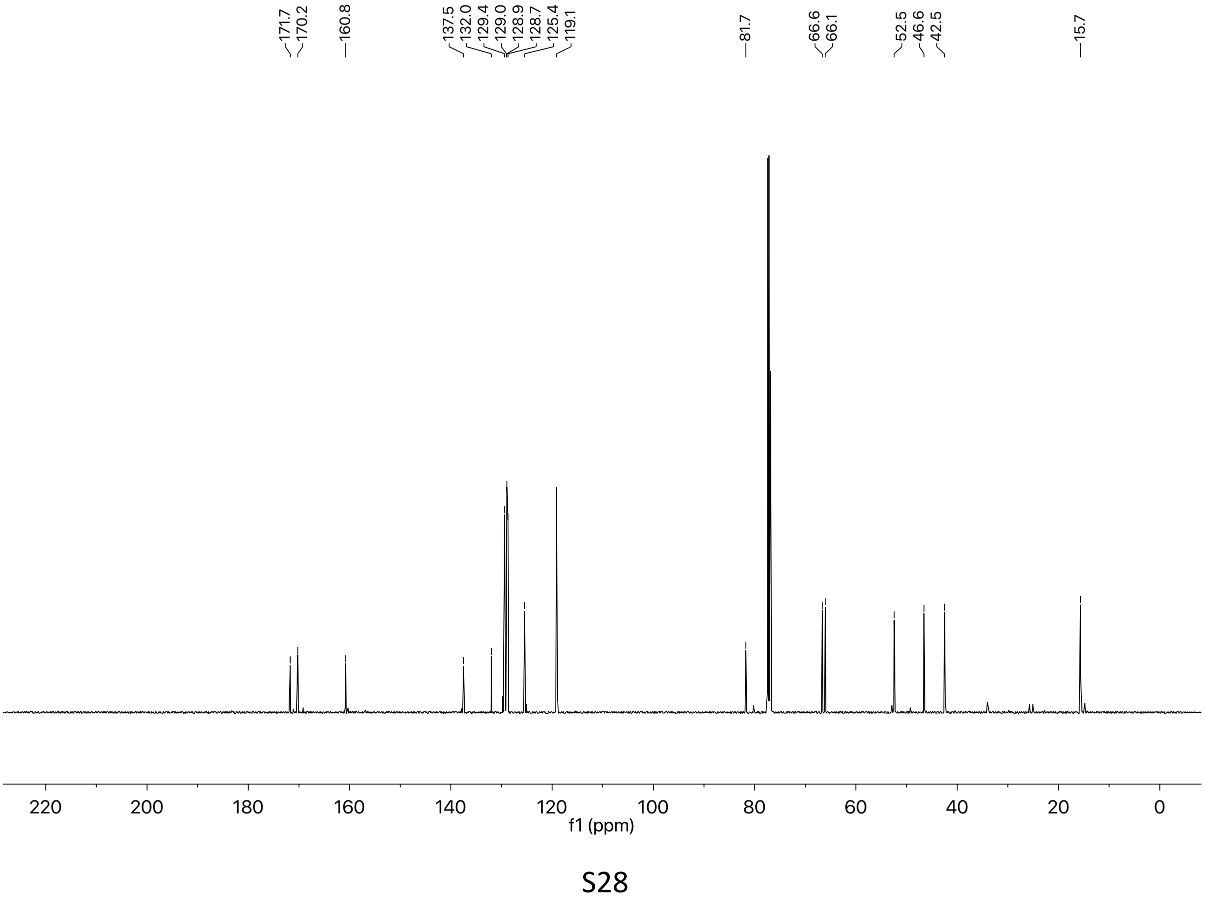

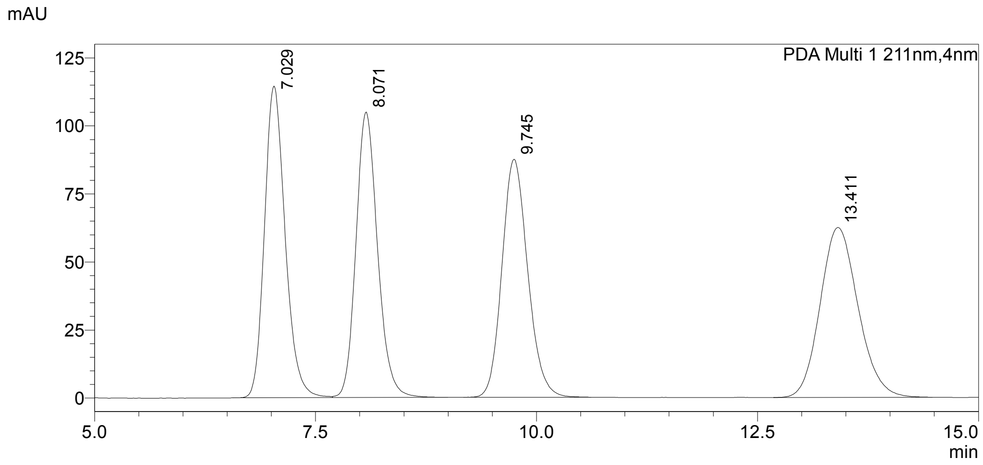

<Peak Table>

PDA Ch1 211nm

Peak#	Ret. Time	Area%
1	7.029	25.601
2	8.071	24.455
3	9.745	24.356
4	13.411	25.587
Total		100.000

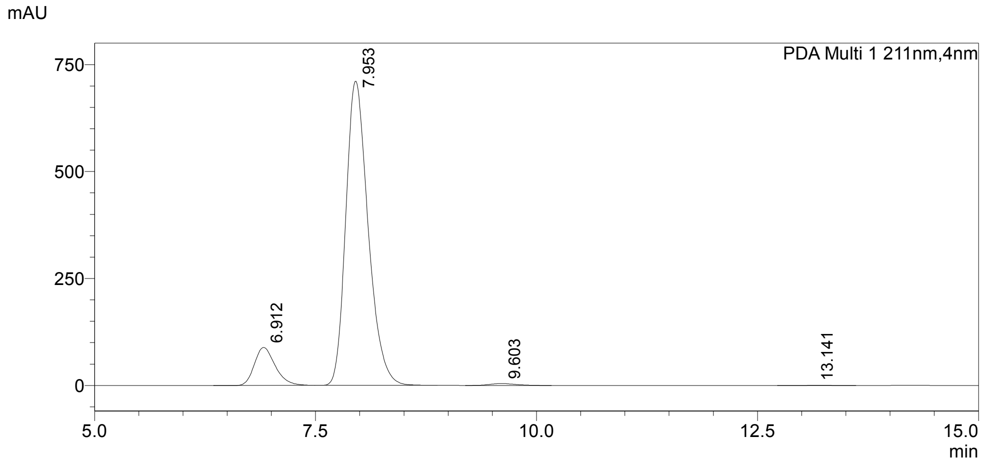

<Peak Table>

PDA Ch1 211nm

Peak#	Ret. Time	Area%
1	6.912	10.344
2	7.953	88.922
3	9.603	0.661
4	13.141	0.073
Total		100.000

5.4. (3'*R*,4*R*)-3-Methyl-3'-(naphth-1-yl)-1-phenyl-spiro[pyrazolin[5]one-4.2-oxetan[4]one] **12** and (3'*S*,4*R*)-3-Methyl-3'-(naphth-1-yl)-1-phenyl-spiro[pyrazolin[5]one-4.2-oxetan[4]one] **13**

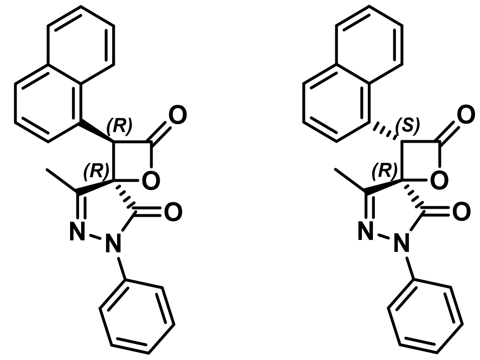

To a solution of 3-methyl-1-(naphth-1-yl)-1*H*-pyrazole-4,5-dione (47.1 mg, 0.25 mmol), 2-(naphth-1-yl)acetic anhydride (132.9 mg, 0.375 mmol) and (2*R*,3*S*)-HyperBTM (3.9 mg, 0.125 mmol) were dissolved in ethyl acetate (6 ml, 0.04 M) at 0 °C. Diisopropylethylamine (54 µl, 0.31 mmol) was added and the reaction mixture was stirred at 0 °C for 3 h. The solvent was removed and the crude reaction mixture was purified by column chromatography (*n*-hexane : ethyl acetate 100:0 to 4:1) to give the title compounds in two fractions: The major diastereomer (40.9 mg, 46%, >95:5 dr) as a white amorphous solid, and a mixture of diastereomers (23.7 mg, 27%, 7:93 dr) as a yellow crystalline solid; combined (64.6 mg, 73%, 66:34 dr). *Data for major diastereomer*: $[\alpha]_D^{20}$ +179.5 (*c* 1.00, $CHCl_3$); **HPLC Analysis:** Chiralpak AD-H (99:1 hexane:isopropanol, flow rate 2.00 ml·min^{-1}, 211 nm, 30 °C) t_R (3'*R*,4*R*)-**12**: 22.6 min, t_R (3'*S*,4*S*)-**12**: 19.6 min, 99:1 er; **IR** ν_{max} (film) 3063, 2359, 1854 (C=O, lactone), 1724 (C=O, pyrazolone), 1597, 1501, 1369, 1319, 1121, 930, 775, 758; **^{1}H NMR** (500 MHz, $CDCl_3$) δ_H: 1.41 (3H, s, C*H*$_3$), 6.01 (1H, s, C*H*COO), 7.17 (1H, app d, $^3J_{HH}$ = 8.4 Hz, CHArC^8*H*), 7.30 (1H, app tt, $^3J_{HH}$ = 7.3 Hz, $^4J_{HH}$ = 1.2 Hz, NArC^4*H*), 7.35 (1H, ddd, $^3J_{HH}$ = 8.4 Hz, 6.9 Hz, $^4J_{HH}$ = 1.2 Hz, CHArC^7*H*), 7.45-7.57 (4H, m, CHArC^3*H* + NArC^{3,5}*H* + CHArC^6*H*), 7.83 (1H, app dt, $^3J_{HH}$ = 7.1 Hz, $^4J_{HH}$ = 1.2 Hz, CHArC^2*H*), 7.84-7.87 (2H, m, NArC^{2,6}*H*), 7.91 (1H, app d, $^3J_{HH}$ = 8.5 Hz, CHArC^5*H*), 7.93(1H, app d, $^3J_{HH}$ = 8.4 Hz, CHArC^4*H*); **^{13}C{^{1}H} NMR** (126 MHz, $CDCl_3$) δ_C: 14.2 (*C*H$_3$), 63.6 (*C*HCOO), 78.0 (*C*C(O)N), 119.2 (NAr*C*^{2,6}H), 121.4 (CHAr*C*^{8}H), 124.7 (CHAr*C*^{1}), 125.4 (CHAr*C*^{2}H), 125.5 (CHAr*C*^{3}H), 126.3 (NAr*C*^{4}H), 127.1 (CHAr*C*^{6}H), 127.7 (CHAr*C*^{7}H), 129.3 (NAr*C*^{3,5}H), 129.5 (CHAr*C*^{5}H), 130.1 (CHAr*C*^{8a}), 130.3 (CHAr*C*^{4}H), 133.9 (CHAr*C*^{4a}), 137.3 (NAr*C*^{1}), 155.6 (*C*=N), 165.5 (*C*OO), 167.2 (*C*(O)N); **HRMS** (ESI$^+$) $C_{22}H_{16}N_2O_3Na$ [M+Na]$^+$ found 379.1045, requires 379.1053 (–2.2 ppm); *Data for minor diastereomer (characterised as a 90:10 dr mixture)*: **mp** 158-160 °C (*dec*); $[\alpha]_D^{20}$ +448.5 (*c* 0.36, $CHCl_3$); Chiralpak AD-H (99:1 hexane:isopropanol, flow rate 2.00 ml·min^{-1}, 211 nm, 30 °C) t_R (3'*S*,4*R*)-**13**: 58.9 min, t_R (3'*R*,4*S*)-**13**: 35.9 min, >99:1 er; **IR** ν_{max} (film) 3063, 1850 (C=O, lactone), 1732 (C=O, pyrazolone), 1597, 1501, 1371, 1314, 1119,

932, 781, 756; **^{1}H NMR** (500 MHz, $CDCl_3$) δ_H: 2.62 (3H, s, C*H*$_3$), 5.95 (1H, s, C*H*COO), 7.08 (1H, t, $^3J_{HH}$ = 7.4 Hz, NArC^4*H*), 7.13 (1H, d, $^3J_{HH}$ = 8.2 Hz, CHArC^8*H*), 7.21 (2H, app t, $^3J_{HH}$ = 8.0 Hz, NArC^{3,5}*H*), 7.29-7.33 (2H, m, NArC^{2,6}*H*), 7.36-7.44 (2H, m, CHArC^7*H* + CHArC^{6}H), 7.54 (1H, app t, $^3J_{HH}$ = 7.7 Hz, CHArC^3*H*), 7.83-7.91 (3H, m, CHArC^2*H* + CHArC^{4}H + CHArC^5*H*); **^{13}C{^{1}H} NMR** (126 MHz, $CDCl_3$) δ_C: 13.2 (*C*H$_3$), 61.6 (*C*HCOO), 77.9 (*C*C(O)N), 119.0 (NAr*C*^{2,6}H), 120.7 (CHAr*C*^{8}H), 123.7 (CHAr*C*^{1}), 125.6 (CHAr*C*^{3}H), 125.9 (NAr*C*^{4}*H*), 126.4 (CHAr*C*^{6}H), 127.4 (CHAr*C*^{7}H), 127.6 (CHAr*C*^{2}H), 128.9 (NAr*C*^{3,5}H), 129.6 (CHAr*C*^{5}H), 130.1 (CHAr*C*^{4}H), 130.2 (CHAr*C*^{8a}), 133.8 (CHAr*C*^{4a}), 136.9 (NAr*C*^{1}), 155.2 (*C*=N), 164.8 (*C*OO), 165.3 (*C*ONAr).

Note: *Upon storage of syn- and* **12** *under air at room temperature a gradual colour change from white to orange was observed. Following ^{1}H NMR analysis of these compounds after approximately one month some decomposition of* **12** *was observed, whilst no decomposition of* **13** *was apparent.*

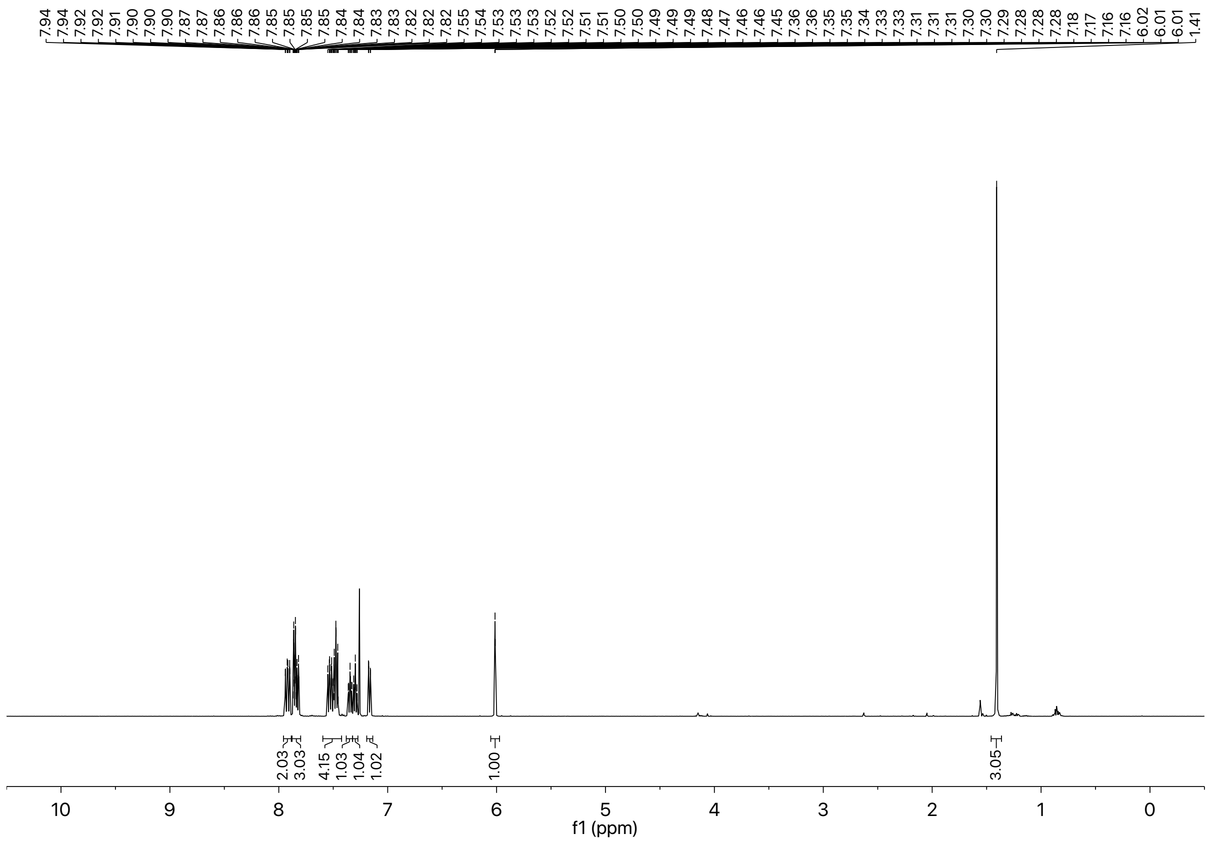
7.94
7.94
7.92
7.92
7.91
7.90
7.90
7.87
7.87
7.86
7.86
7.85
7.85
7.85
7.84
7.84
7.83
7.83
7.82
7.82
7.82
7.55
7.54
7.53
7.53
7.53
7.52
7.52
7.51
7.50
7.50
7.49
7.49
7.49
7.48
7.47
7.46
7.46
7.45
7.36
7.36
7.35
7.34
7.33
7.33
7.31
7.31
7.31
7.30
7.30
7.29
7.28
7.28
7.28
7.18
7.17
7.16
6.02
6.01
6.01
1.41
2.03
3.03
4.15
1.03
1.04
1.02
1.00
3.05
10
9
8
7
6
5
4
3
2
1
0
f1 (ppm)
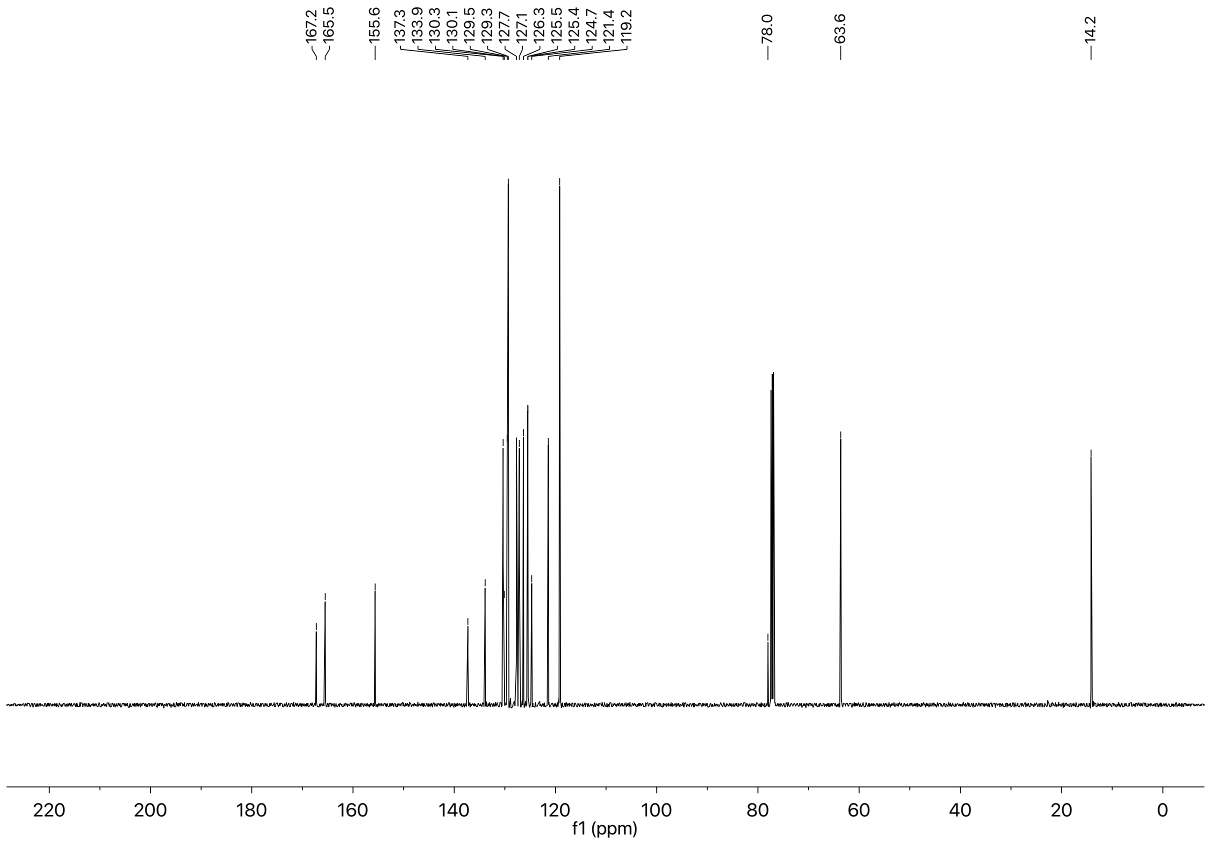
167.2
165.5
155.6
137.3
133.9
130.3
130.1
129.5
129.3
127.7
127.1
126.3
125.5
125.4
124.7
121.4
119.2
78.0
63.6
14.2
220
200
180
160
140
120
100
80
60
40
20
0
f1 (ppm)

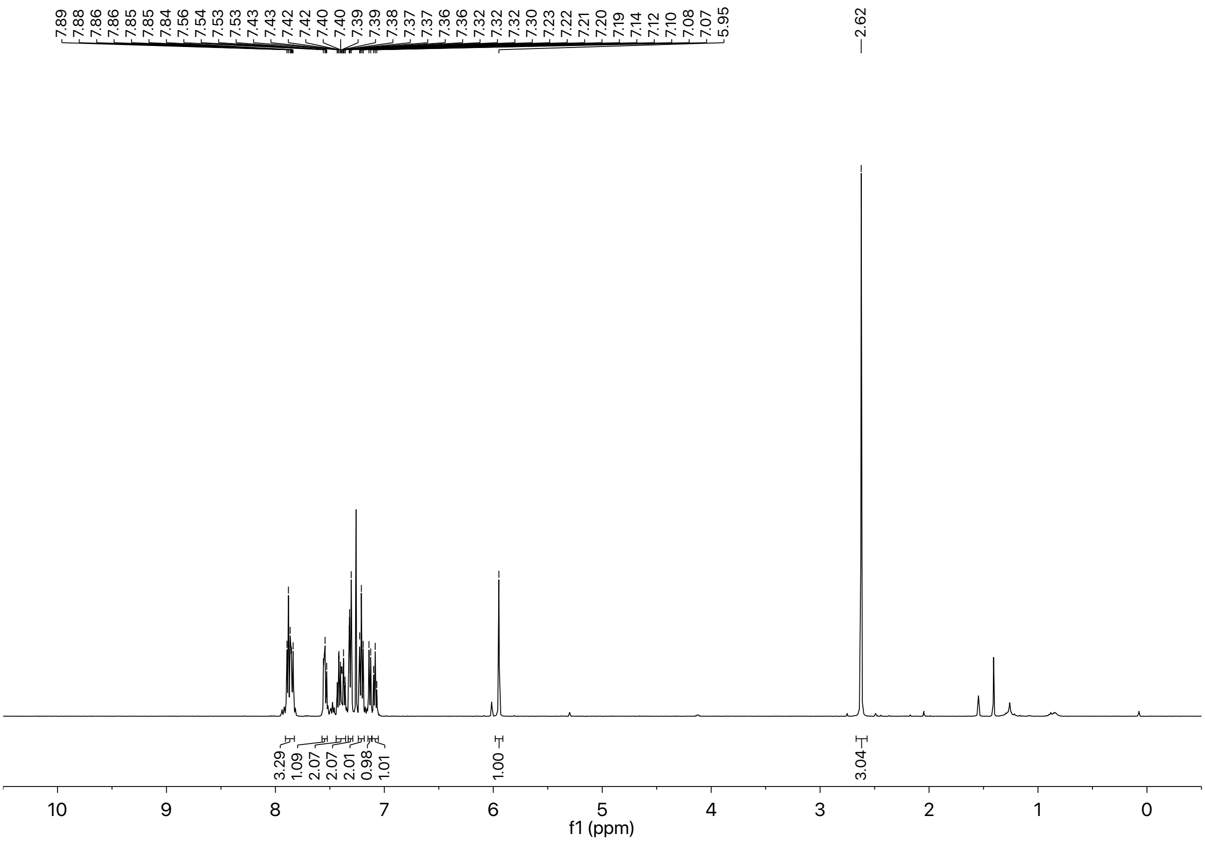
7.89
7.88
7.86
7.86
7.85
7.85
7.84
7.56
7.54
7.53
7.53
7.43
7.43
7.42
7.42
7.40
7.40
7.39
7.39
7.38
7.37
7.37
7.36
7.36
7.32
7.32
7.32
7.30
7.23
7.22
7.22
7.21
7.20
7.19
7.14
7.12
7.10
7.08
7.07
5.95
2.62
3.29
1.09
2.07
2.07
2.01
0.98
1.01
1.00
3.04
10 9 8 7 6 5 4 3 2 1 0
f1 (ppm)

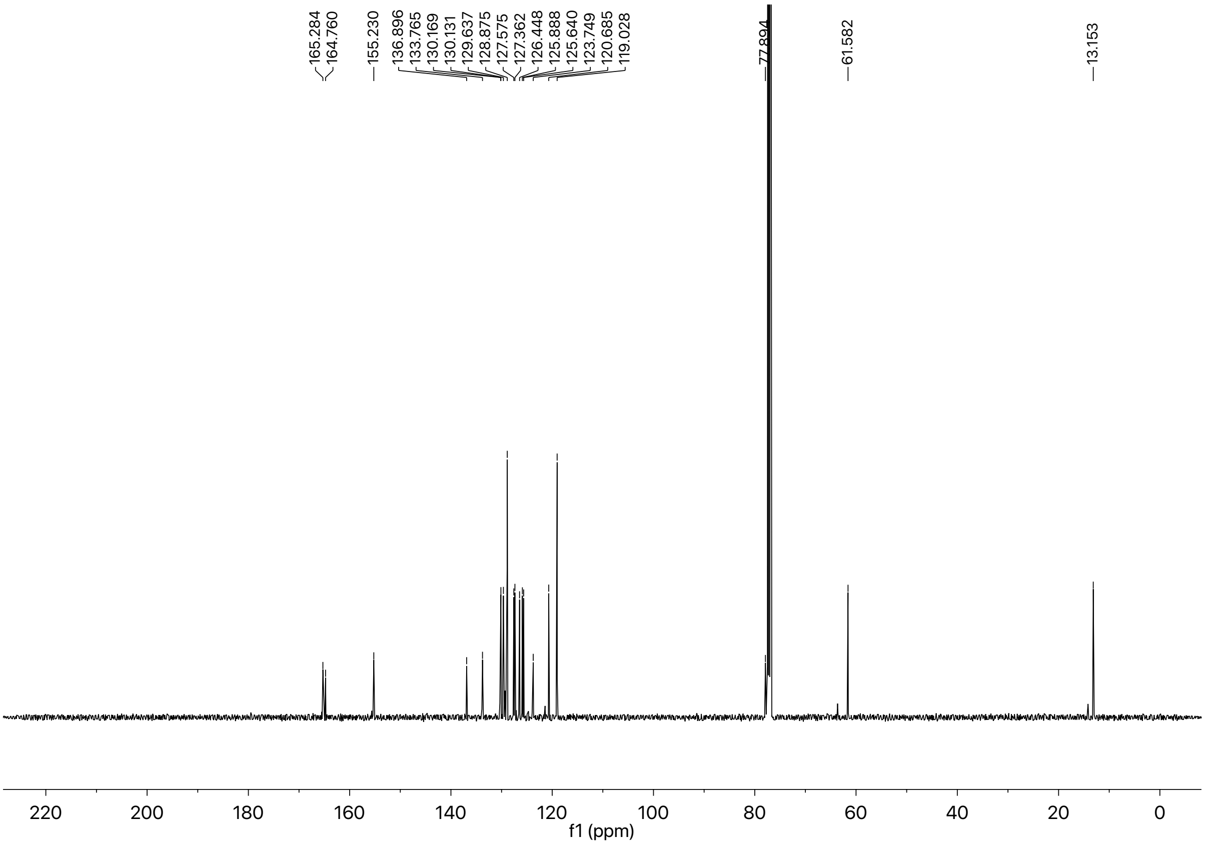
165.284
164.760
155.230
136.896
133.765
130.169
130.131
129.637
128.875
127.575
127.362
126.448
125.888
125.640
123.749
120.685
119.028
77.894
61.582
13.153
220 200 180 160 140 120 100 80 60 40 20 0
f1 (ppm)

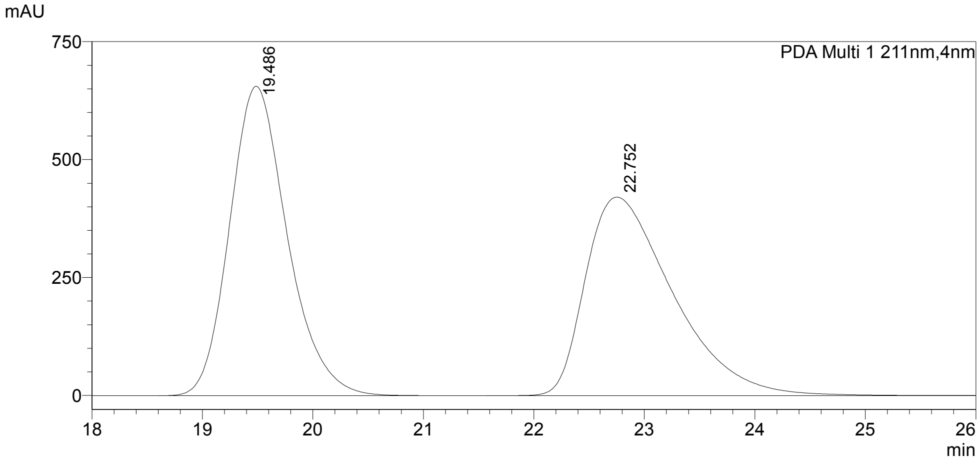

<Peak Table>

PDA Ch1 211nm

Peak#	Ret. Time	Area%
1	19.486	50.611
2	22.752	49.389
Total		100.000

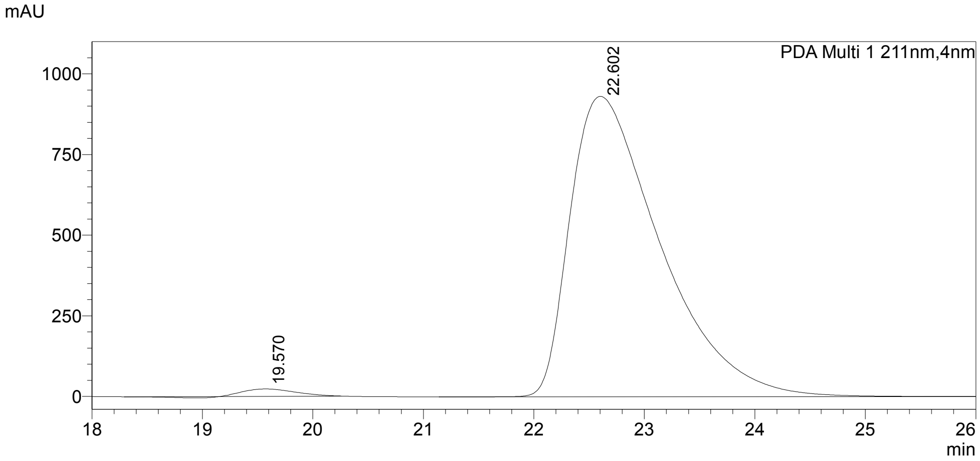

<Peak Table>

PDA Ch1 211nm

Peak#	Ret. Time	Area%
1	19.570	1.199
2	22.602	98.801
Total		100.000

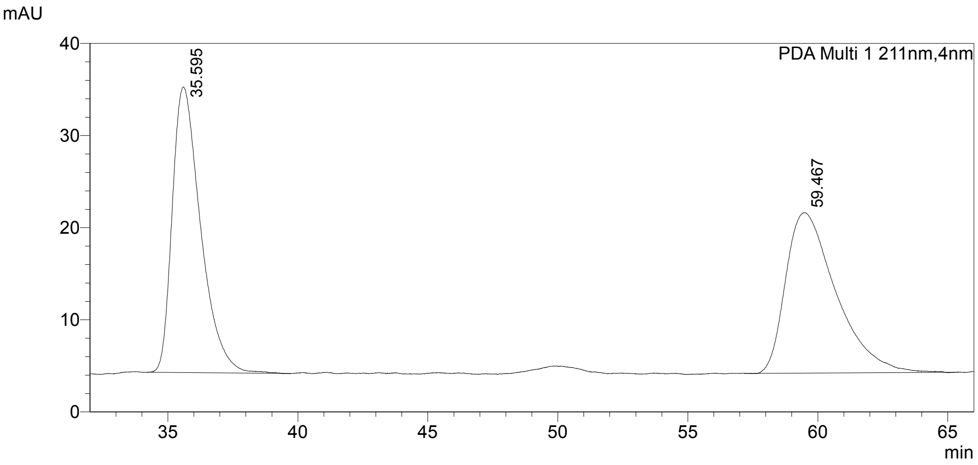

<Peak Table>

PDA Ch1 211nm

Peak#	Ret. Time	Area%
1	35.595	50.197
2	59.467	49.803
Total		100.000

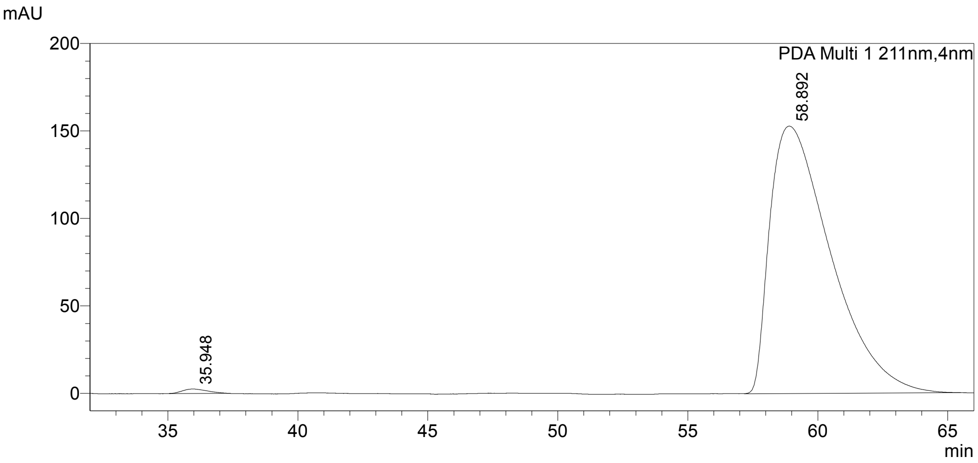

<Peak Table>

PDA Ch1 211nm

Peak#	Ret. Time	Area%
1	35.948	0.684
2	58.892	99.316
Total		100.000

5.5. (1′*R*,4*R*)- and (1′*S*,4*R*)-4-hydroxy-5-methyl-4-(2-morpholino-1-(α-naphthyl)-2-oxoethyl)-2-phenyl-2,4-dihydro-3*H*-pyrazol-3-one **14**

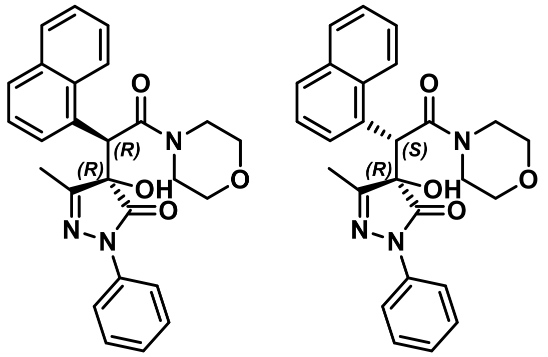

To a solution of 3-methyl-1-phenyl-1*H*-pyrazole-4,5-dione (47.1 mg, 0.25 mmol), 2-(α-naphthyl)acetic anhydride (132.9 mg, 0.375 mmol) and (2*R*,3*S*)-HyperBTM (3.9 mg, 0.125 mmol) were dissolved in ethyl acetate (6 ml, 0.04 M) at 0 °C. Diisopropylethylamine (54 µl, 0.31 mmol) was added and the reaction mixture was stirred at 0 °C for 3 h. Morpholine (66 µl, 0.75 mmol) was added and the reaction mixture was stirred for 16 h at rt. The reaction mixture was diluted with ethyl acetate and washed sequentially with 1 M aq. HCl (×2), sat. aq. $NaHCO_3$ (×2), and brine (×1). The organic layer was dried over $MgSO_4$, filtered, and concentrated *in vacuo*. Purification by column chromatography (*n*-hexane : ethyl acetate 4:1 to 1:1) gave the title compound as a mixture of diastereomers as white amorphous solid (99.7 mg, 0.22 mmol, 90%, >95:5 d.r.). $[\alpha]_D^{20}$ +336.4 (*c* 0.50, $CDCl_3$); **IR** ν_{max} (film) 3352 (O-H), 3059, 2922 (C-H), 2857 (C-H), 1717 (C=O, pyrazolone), 1639, 1620, 1595, 1501, 1360, 1113, 781; **HRMS** (ESI^+) $C_{26}H_{25}N_3O_4$ $[M+H]^+$ found 466.17231, requires 466.17373 (–3.0 ppm). *Data for major diastereomer*: **HPLC Analysis:** Chiralpak AD-H (95:5 hexane:isopropanol, flow rate 2.0 ml·min^{-1}, 211 nm, 30 °C) t_R (1′*R*,4*R*)-**14**: 31.9 min, t_R (1′*S*,4*S*)-**14**: 42.4 min, >99:1 er; **^{1}H NMR** (500 MHz, $CDCl_3$) δ_H: 1.49 (3H, s, C*H*$_3$), 2.76 (1H, ddd, $^2J_{HH}$ = 10.5 Hz, $^3J_{HH}$ = 7.2 Hz, 3.0 Hz, $NCH_2CH_AH_B$), 2.96 (1H, ddd, $^2J_{HH}$ =13.5 Hz, $^3J_{HH}$ = 6.0 Hz, 3.0 Hz, $NCH_AH_BCH_2$), 3.21-3.33 (2H, m, $NCH_AH_BCH_2$, $NCH_2CH_AH_B$), 3.49 (1H, ddd, $^2J_{HH}$ = 11.6 Hz, $^3J_{HH}$ = 7.4 Hz, 3.0 Hz, $NCH_2CH_CH_D$), 3.59 (1H, ddd, $^2J_{HH}$ = 13.3 Hz, $^3J_{HH}$ = 7.4 Hz, 3.0 Hz, $NCH_CH_DCH_2$), 3.65-3.69 (1H, m, $NCH_2CH_CH_D$), 3.81 (1H, ddd, $^2J_{HH}$ = 13.3 Hz, $^3J_{HH}$ = 5.8 Hz, 3.0 Hz, $NCH_CH_DCH_2$), 4.91 (1H, s, C*H*Ar), 7.13-7.17 (1H, m, NArC^4*H*), 7.31-7.36 (2H, m, NArC^{3,5}*H*), 7.48-7.55 (2H, m, CHArC^{3,7}*H*), 7.55-7.59 (1H, m, CHArC^6*H*) 7.66-7.72 (2H, m, NArC^{2,6}*H*), 7.83 (1H, d, $^3J_{HH}$ = 7.1 Hz, CHArC^2*H*), 7.85-7.91 (2H, m, CHArC^{4,8}*H*), 7.95 (1H, d, $^3J_{HH}$ = 8.6 Hz, CHAr^5*H*); **^{13}C{^{1}H} NMR** (126 MHz, $CDCl_3$) δ_C: 14.9 (*C*H$_3$), 42.8 (N*C*H$_C$H$_D$CH$_2$), 44.5 (*C*HAr), 46.3 (N*C*H$_A$H$_B$CH$_2$), 65.9 (NCH$_2$*C*H$_A$H$_B$), 66.6 (NCH$_2$*C*H$_C$H$_D$), 81.3 (*C*-OH), 119.0 (NAr*C*^{2,6}H), 121.3 (CHAr*C*^{5}H), 125.3 (NArC^4*H*), 125.6 (CHAr*C*^{3}H), 126.3 (CHAr*C*^{7}H), 127.7 (CHAr*C*^{1}), 127.9 (CHAr*C*^{6}H), 128.0 (CHAr*C*^{2}H), 128.9 (NAr*C*^{3,5}H), 129.8 (CHAr*C*^{4}H), 129.9 (CHAr*C*^{6}H), 130.9 (CHAr*C*^{8a}), 134.0 (CHAr*C*^{4a}), 137.7 (NAr*C*^{1}), 160.6 (*C*=N), 171.0

($CON(CH_2CH_2)_2O$), 172.7 ($CONAr$); *Data for minor diastereomer*: **^{1}H NMR** (500 MHz, $CDCl_3$) (*selected*) δ_H: 2.17 (3H, s, CH_3), 2.67 (1H, ddd, $^2J_{HH}$ = 10.6 Hz, $^3J_{HH}$ = 7.3 Hz, 2.8 Hz, $NCH_2CH_AH_B$), 4.87 (1H, s, $CHAr$).

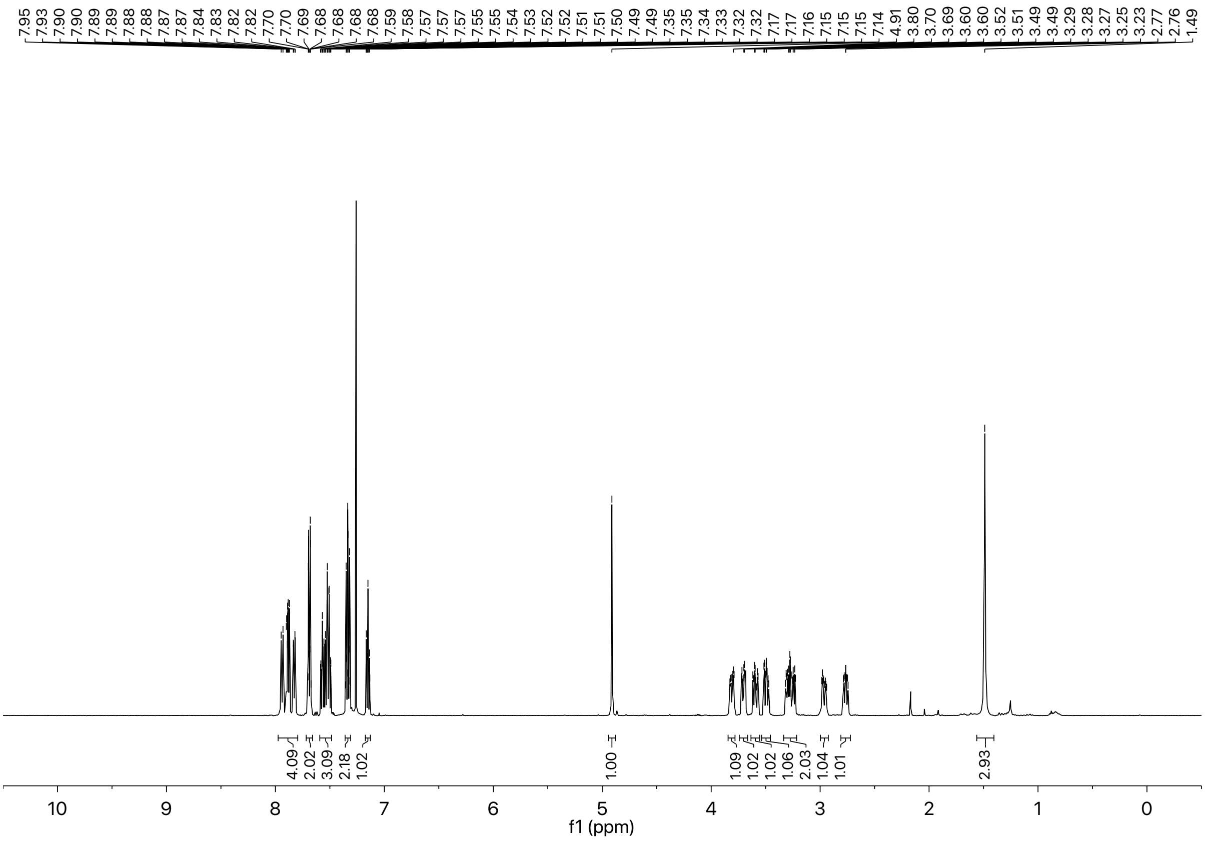

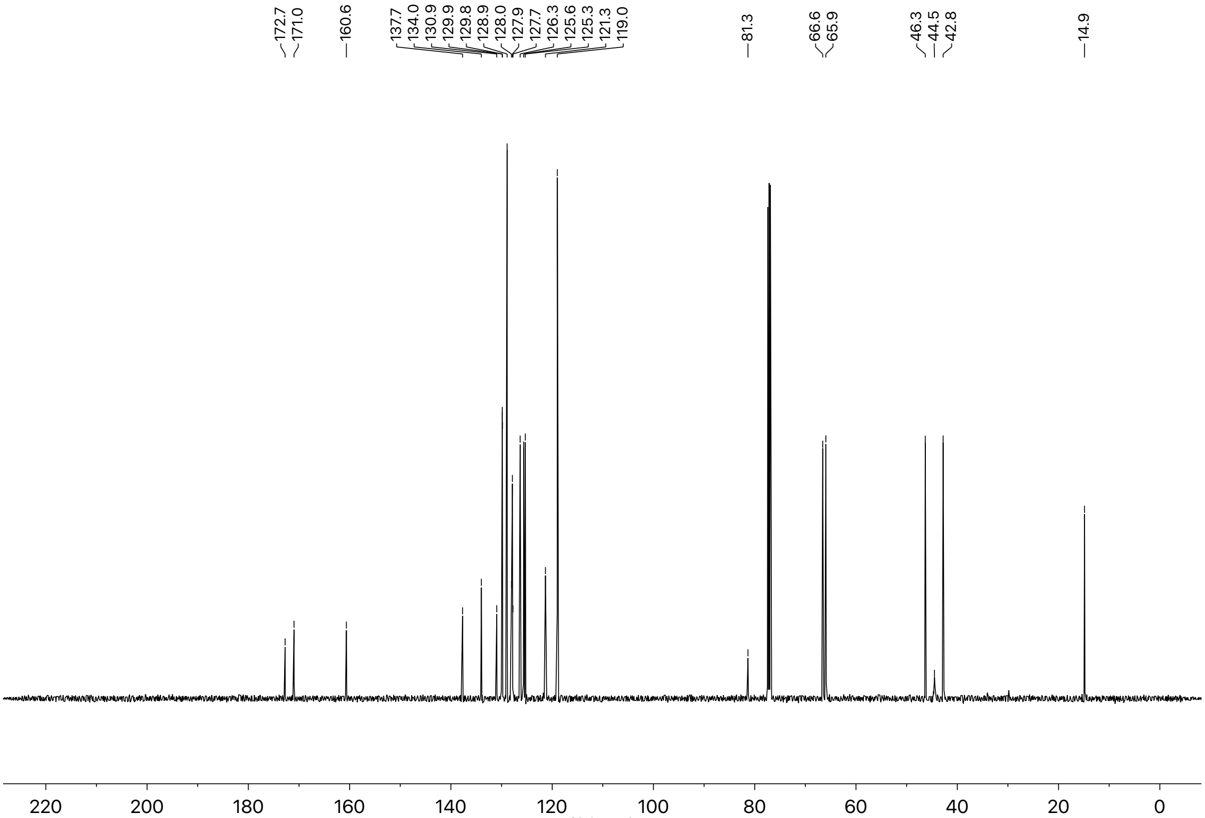

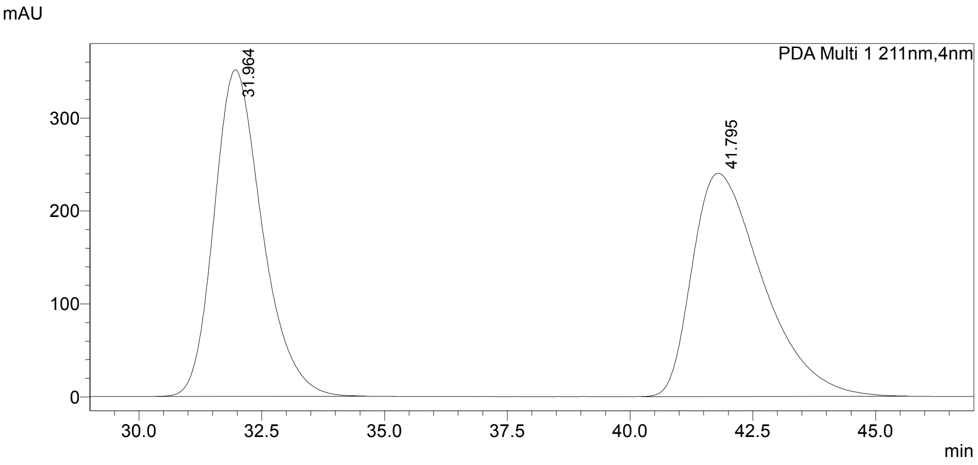

<Peak Table>

PDA Ch1 211nm

Peak#	Ret. Time	Area%
1	31.964	50.040
2	41.795	49.960
Total		100.000

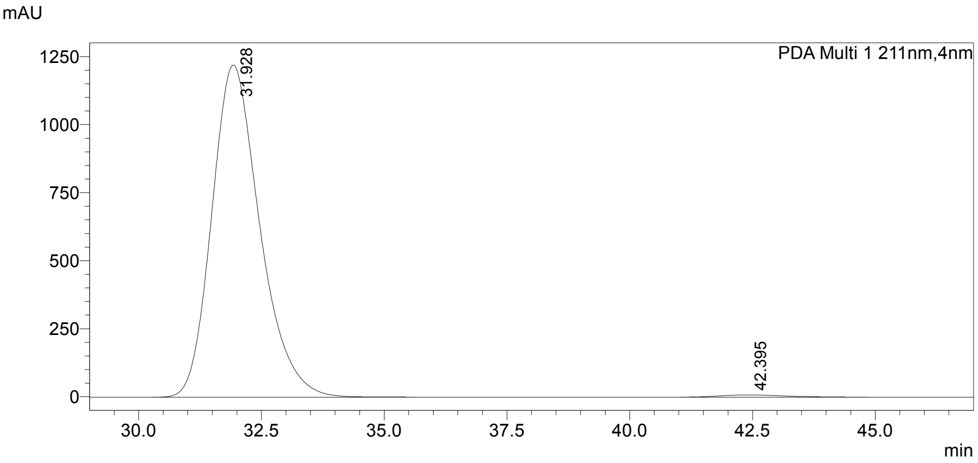

<Peak Table>

PDA Ch1 211nm

Peak#	Ret. Time	Area%
1	31.928	99.084
2	42.395	0.916
Total		100.000

5.6. (1′*R*,4*R*)- and (1′*S*,4*R*)-4-(1-(*p*-chlorophenyl)2-morpholino-2-oxoethyl)-4-hydroxy-5-methyl-2-phenyl-2,4-dihydro-3H-pyrazol-3-one **21**

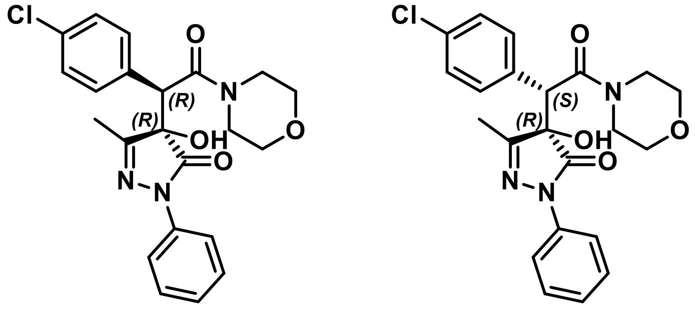

To a solution of 3-methyl-1-phenyl-1*H*-pyrazole-4,5-dione (47.1 mg, 0.25 mmol), 2-(*p*-chlorophenyl)acetic anhydride (121.2 mg, 0.375 mmol) and (2*R*,3*S*)-HyperBTM (3.9 mg, 0.125 mmol) were dissolved in ethyl acetate (6 ml, 0.04 M) at 0 °C. Diisopropylethylamine (54 µl, 0.31 mmol) was added and the reaction mixture was stirred at 0 °C for 3 h. Morpholine (66 µl, 0.75 mmol) was added and the reaction mixture was stirred for 16 h at rt. The reaction mixture was diluted with ethyl acetate and washed sequentially with 1 M aq. HCl (×2), sat. aq. $NaHCO_3$ (×2), and brine (×1). The organic layer was dried over $MgSO_4$, filtered, and concentrated *in vacuo*. Purification by column chromatography (*n*-hexane : ethyl acetate 3:1 to 1:1) gave the title compound as a mixture of diastereomer as a white solid (56.0 mg, 0.13 mmol, 52%, >95:5 d.r.). **mp** 215-218 °C (*dec*); $[\alpha]_D^{20}$ +188.5 (*c* 1.00, $CDCl_3$); **IR** ν_{max} (film) 3296 (O-H), 2920 (C-H), 2855 (C-H), 2361, 2342, 2330, 1717 (C=O, pyrazolone), 1622, 1595, 1489, 1111, 760; **HRMS** (ESI^+) $C_{22}H_{21}ClN_3O_4Na$ $[M+Na]^+$ found 450.11791, requires 450.11910 (–2.6 ppm). *Data for major diastereomer*: **HPLC Analysis:** Chiralpak AD-H (95:5 hexane:isopropanol, flow rate 2.00 ml·min^{-1}, 211 nm, 30 °C) t_R (1′*R*,4*R*)-**21**: 22.3 min, t_R (1′*S*,4*S*)-**21**: 35.1 min, 98.5:1.5 er; **^{1}H NMR** (500 MHz, $CDCl_3$) δ_H: 1.95 (3H, s, C*H*$_3$), 3.06-3.25 (2H, m, NC*H*$_A$H$_B$CH$_2$, NCH$_2$C*H*$_A$H$_B$), 3.34 (1H, ddd, $^2J_{HH}$ = 13.3 Hz, $^3J_{HH}$ = 6.7 Hz, 3.0 Hz, NCH$_A$*H*$_B$CH$_2$), 3.53 (1H, ddd, $^2J_{HH}$ = 11.3 Hz, $^3J_{HH}$ = 6.3 Hz, 3.0 Hz, NCH$_2$CH$_A$*H*$_B$), 3.56-3.62 (1H, m, NCH$_2$C*H*$_C$H$_D$), 3.65-3.77 (3H, m, NC*H*$_C$*H*$_D$CH$_2$,NCH$_2$CH$_C$*H*$_D$), 4.18 (1H, s, C*H*Ar) 6.23 (1H, br s, O*H*), 7.19 (1H, app t, $^3J_{HH}$ = 7.4 Hz, NArC^4*H*), 7.23 (2H, app d, $^3J_{HH}$ = 8.4 Hz, CHArC^{2,6}*H*), 7.30 (2H, app d, $^3J_{HH}$ = 8.4 Hz, CHArC^{3,5}*H*), 7.35-7.41 (2H, m, NArC^{3,5}*H*), 7.73 (2H, d, $^3J_{HH}$ = 7.6 Hz, NArC^{2,6}*H*); **^{13}C{^{1}H} NMR** (126 MHz, $CDCl_3$) δ_C: 15.7 (*C*H$_3$), 42.6 (N*C*H$_C$H$_D$CH$_2$), 46.6 (N*C*H$_A$H$_B$CH$_2$), 51.5 (*C*HAr), 66.1 (NCH$_2$*C*H$_A$H$_B$), 66.7 (NCH$_2$*C*H$_C$H$_D$), 81.5 (*C*-OH), 119.0 (NAr*C*^{2,6}H), 125.6 (NAr*C*^{4}H), 129.0 (NAr*C*^{3,5}H), 129.6 (CHAr*C*^{3,5}H), 130.1 (CHAr*C*^{2,6}H), 130.6 (CHAr*C*^{1}), 135.2 (CHAr*C*^{4}Cl), 137.4 (NAr*C*^{1}), 160.4 (*C*=N), 169.8 (*C*ON(CH$_2$CH$_2$)$_2$O), 171.6 (*C*ONAr); *Data for minor diastereomer*: **HPLC Analysis:** Chiralpak AD-H (95:5 hexane:isopropanol, flow rate 2.00 ml·min^{-1}, 211 nm, 30 °C) t_R (1′*S*,4*R*)-**21**: 27.9 min, t_R (1′*R*,4*S*)-**21**: 76.3 min (not detected); **^{1}H NMR** (500 MHz, $CDCl_3$) (*selected*) δ_H:

2.02 (3H, s, C*H*$_3$), 4.07 (1H, s, C*H*Ar), 5.34 (1H, br s, O*H*); **^{13}C{^{1}H} NMR** (126 MHz, CDCl$_3$) (*selected*) δ_C: 15.0 (*C*H$_3$), 118.9 (NAr*C*^{2,6}H), 125.3 (NAr*C*^{4}H), 129.2 (Ar*C*H), 131.1 (Ar*C*H).

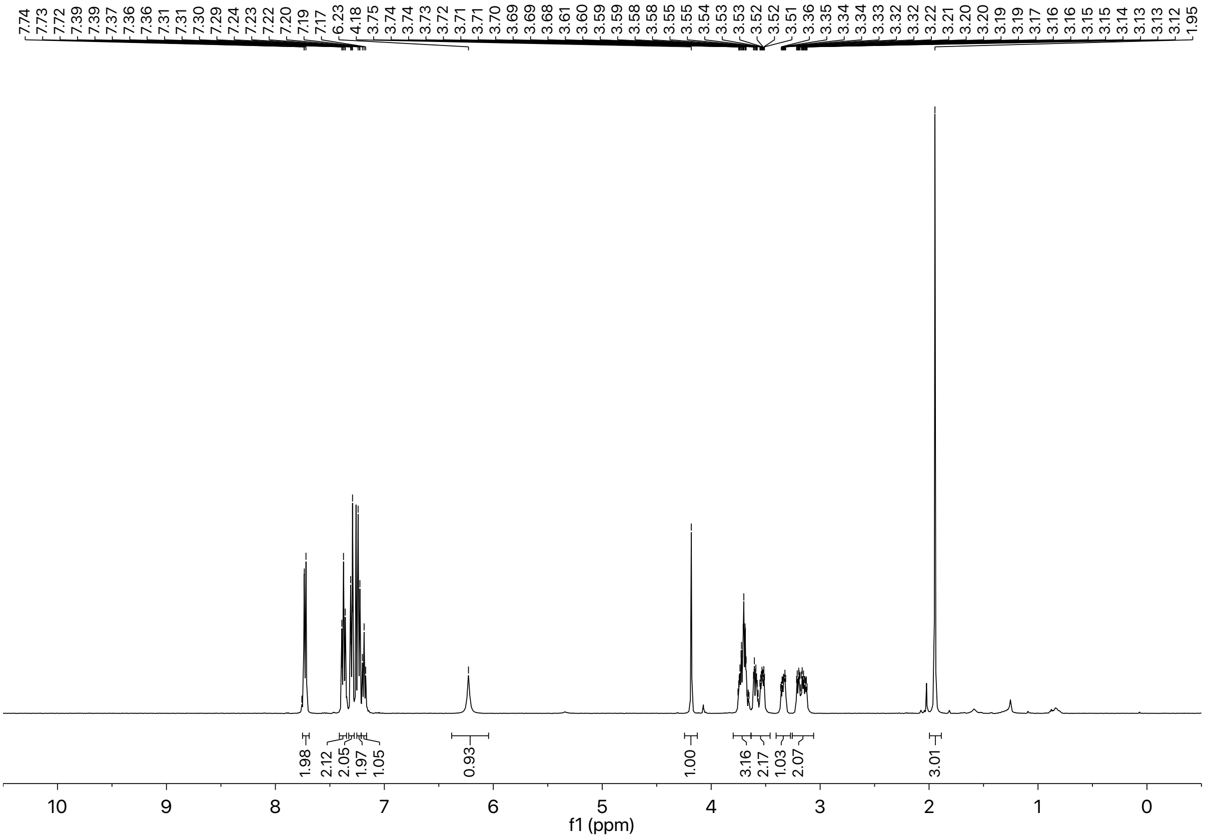

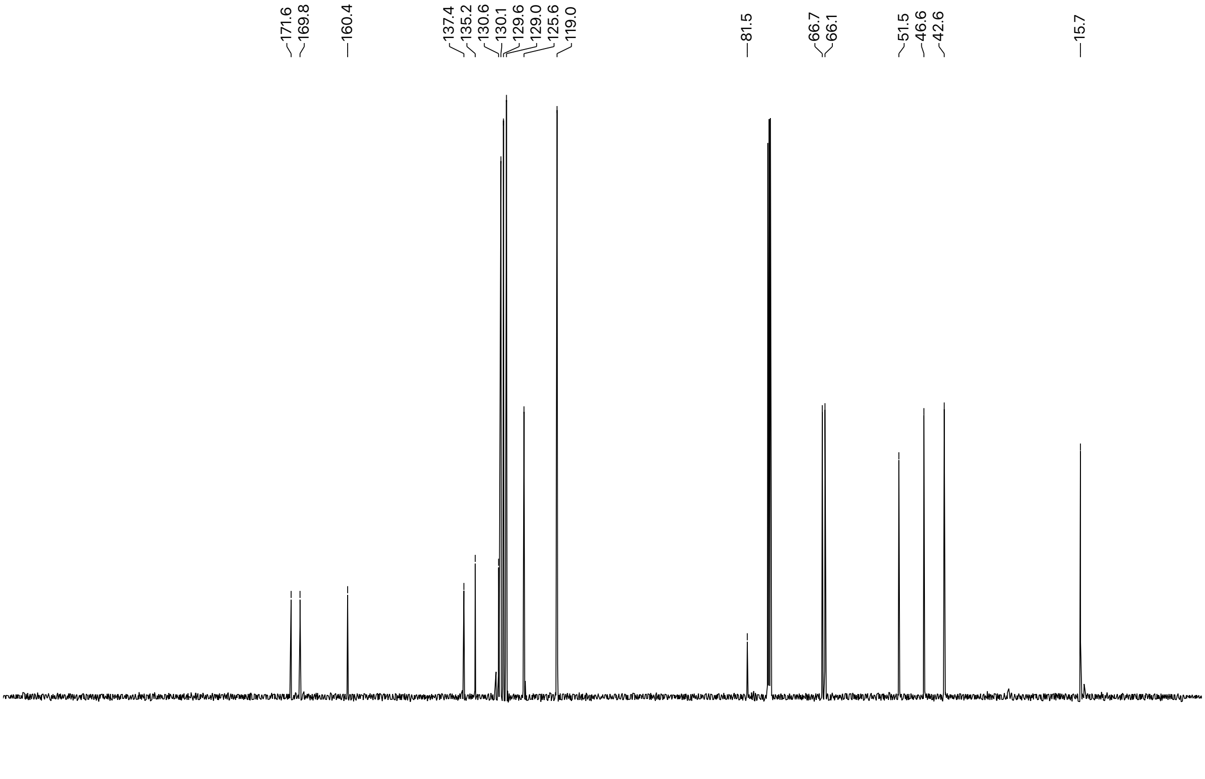

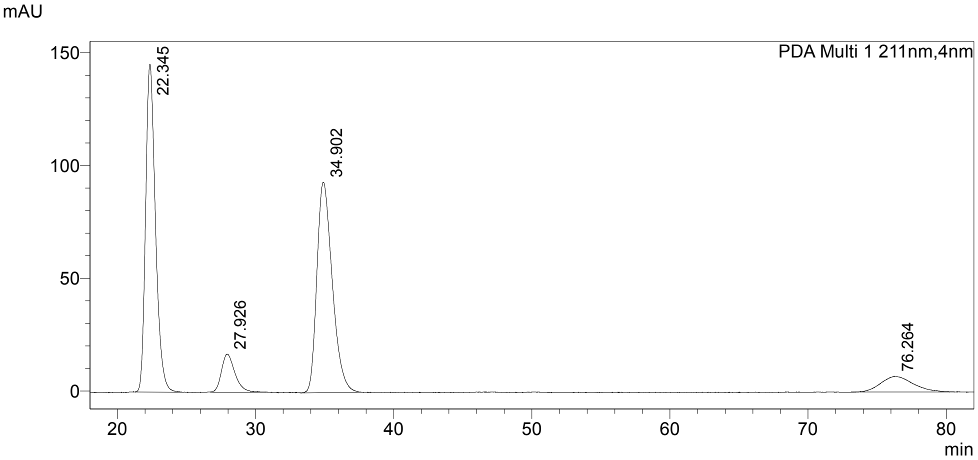

<Peak Table>

PDA Ch1 211nm

Peak#	Ret. Time	Area%
1	22.345	43.118
2	27.926	6.825
3	34.902	43.312
4	76.264	6.746
Total		100.000

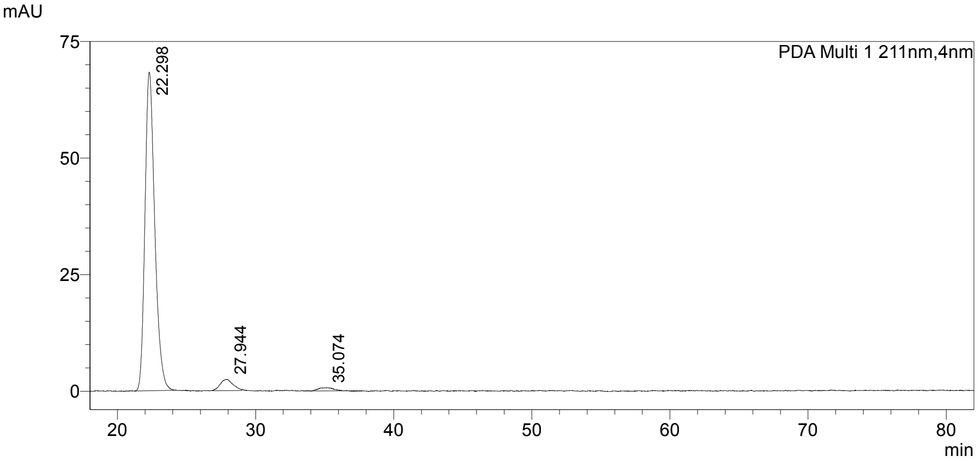

<Peak Table>

PDA Ch1 211nm

Peak#	Ret. Time	Area%
1	22.298	94.076
2	27.944	4.398
3	35.074	1.526
Total		100.000

5.7. (1′*R*,4*R*)- and (1′*S*,4*R*)-4-(1-(4-bromophenyl)-2-morpholino-2-oxoethyl)-4-hydroxy-5-methyl-2-phenyl-2,4-dihydro-3*H*-pyrazole-3-one **22**

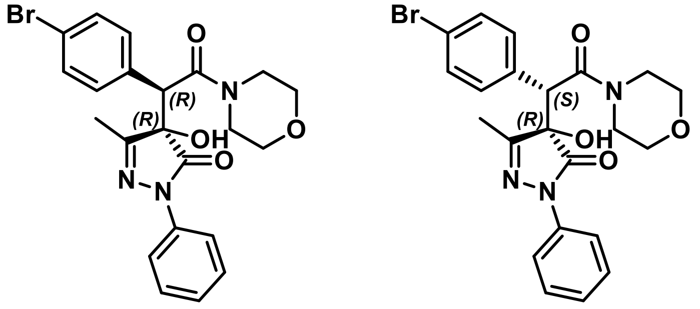

To a solution of 3-methyl-1-phenyl-1*H*-pyrazole-4,5-dione (47.1 mg, 0.25 mmol), 2-(*p*-bromophenyl)acetic anhydride (154.5 mg, 0.375 mmol) and (2*R*,3*S*)-HyperBTM (3.9 mg, 0.125 mmol) were dissolved in ethyl acetate (6 ml, 0.04 M) at 0 °C. Diisopropylethylamine (54 µl, 0.31 mmol) was added and the reaction mixture was stirred at 0 °C for 3 h. Morpholine (66 µl, 0.75 mmol) was added and the reaction mixture was stirred for 16 h at rt. The reaction mixture was diluted with ethyl acetate and washed sequentially with 1 M aq. HCl (×2), sat. aq. $NaHCO_3$ (×2), and brine (×1). The organic layer was dried over $MgSO_4$, filtered, and concentrated *in vacuo*. Purification by column chromatography (*n*-hexane : ethyl acetate 4:1 to 3:4) gave the title compound as a mixture of diastereomer as a white solid (47.4 mg, 0.10 mmol, 40%, >95:5 d.r.). **mp** 230-232 °C (*dec*); $[\alpha]_D^{20}$ +215.3 (*c* 1.00, $CDCl_3$); **IR** ν_{max} (film) 3335 (O-H), 2968 (C-H), 2922 (C-H), 2859 (C-H), 1717 (C=O, pyrazolone), 1643, 1626, 1489, 1115, 758; **HRMS** (ESI$^+$) $C_{22}H_{22}BrN_3O_4Na$ $[M(^{79}Br)+Na]^+$ found 494.0683, requires 494.0686 (–0.5 ppm). *Data for major diastereomer*: **HPLC Analysis:** Chiralpak AD-H (95:5 hexane:isopropanol, flow rate 2.00 ml·min^{-1}, 211 nm, 30 °C) t_R (1′*R*,4*R*)-**22**: 23.6 min, t_R (1′*S*,4*S*)-**22**: 36.1 min, 98:2 er; **^{1}H NMR** (500 MHz, $CDCl_3$) δ_H: 1.94 (3H, s, CH_3), 3.14 (1H, ddd, $^2J_{HH}$ = 13.3 Hz, $^3J_{HH}$ = 6.4 Hz, 3.0 Hz, NCH_AH$_B$CH$_2$), 3.20 (1H, ddd, $^2J_{HH}$ = 11.4 Hz, $^3J_{HH}$ = 6.7 Hz, 3.0 Hz, NCH$_2$CH_AH$_B$), 3.34 (1H, ddd, $^2J_{HH}$ = 13.3 Hz, $^3J_{HH}$ = 6.7 Hz, 3.1 Hz, NCH$_A$$H_BCH_2$), 3.53 (1H, ddd, $^2J_{HH}$ = 11.4 Hz, $^3J_{HH}$ = 6.4 Hz, 3.1 Hz, NCH$_2$CH$_A$$H_B$), 3.57-3.63 (1H, m, NCH$_2CH_CH_D$), 3.67-3.76 (3H, m, NC$H_C$$H_DCH_2$, NCH$_2CH_C$$H_D$), 4.17 (1H, s, C*H*Ar), 6.22 (1H, br s, O*H*), 7.14-7.21 (3H, m, CHArC^{2,4,6}*H*), 7.38 (2H, app t, $^3J_{HH}$ = 7.9 Hz, NArC^{3,5}*H*), 7.45 (2H, d, $^3J_{HH}$ = 8.4 Hz, CHArC^{3,5}*H*), 7.73 (2H, app d, $^3J_{HH}$ = 8.1 Hz, NArC^{2,6}*H*); **^{13}C{^{1}H} NMR** (126 MHz, $CDCl_3$) δ_C: 15.7 (*C*H$_3$), 42.6 (N*C*H$_C$H$_D$CH$_2$), 46.6 (N*C*H$_A$H$_B$CH$_2$), 51.6 (*C*HAr), 66.1 (NCH$_2$*C*H$_A$H$_B$), 66.7 (NCH$_2$*C*H$_C$H$_D$), 81.4 (*C*-OH), 119.1 (NAr*C*^{2,6}H), 123.3 (CHAr*C*^{4}Br), 125.6 (NAr*C*^{4}H), 129.0 (NAr*C*^{3,5}H), 130.4 (CHAr*C*^{2,6}H), 131.1 (CHAr*C*^{1}), 132.6 (CHAr*C*^{3,5}H), 137.4 (NAr*C*^{1}), 160.4 (*C*=N), 169.7 (*C*ON(CH$_2$CH$_2$)$_2$O), 171.5 (*C*ONAr); *Data for minor diastereomer*: **HPLC Analysis:** Chiralpak AD-H (95:5 hexane:isopropanol, flow rate 2.0 ml·min^{-1}, 211 nm, 30 °C) t_R (1′*S*,4*R*)-**22**: 28.6 min, t_R (1′*R*,4*S*)-

22: 77.6 min (not detected); **^{1}H NMR** (500 MHz, $CDCl_3$) (*selected*) δ_H: 2.02 (3H, s, C*H*$_3$), 4.06 (1H, s, C*H*Ar), 5.31 (1H, br s, O*H*).

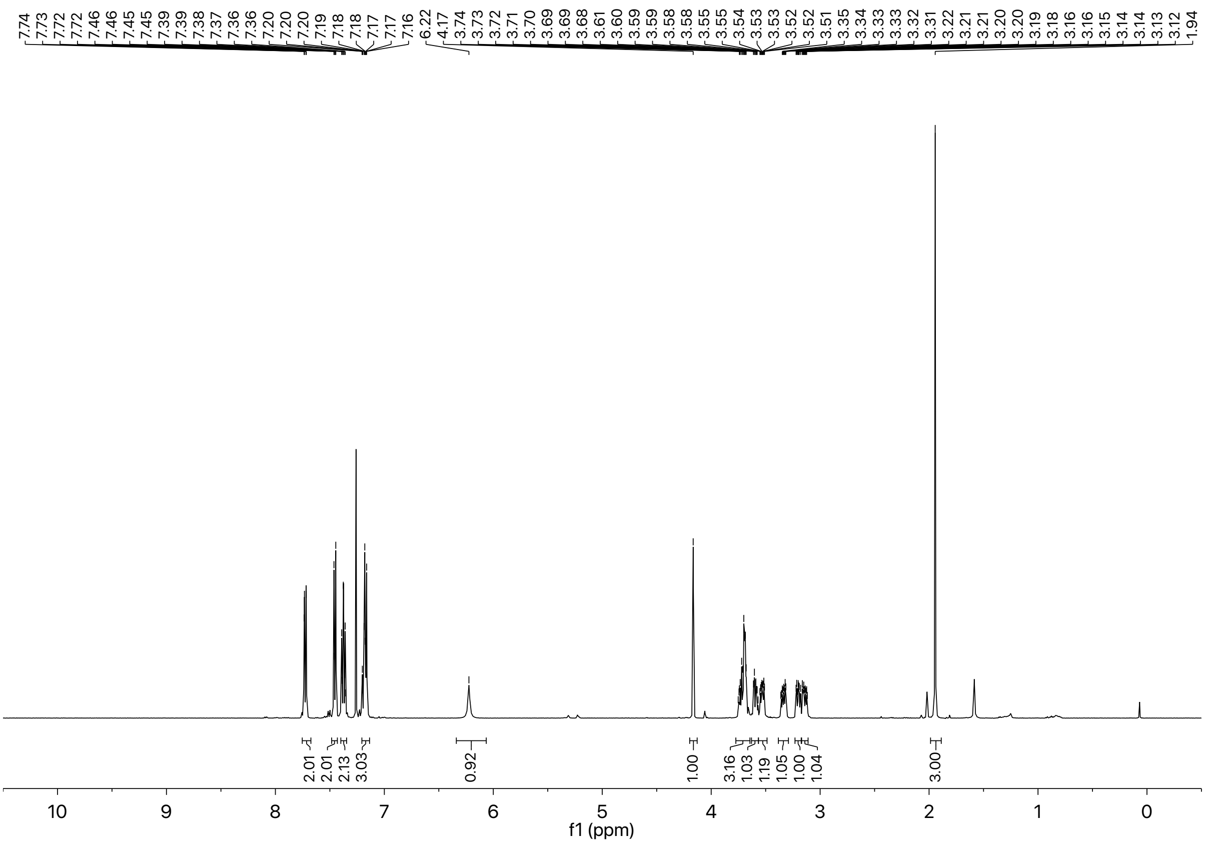

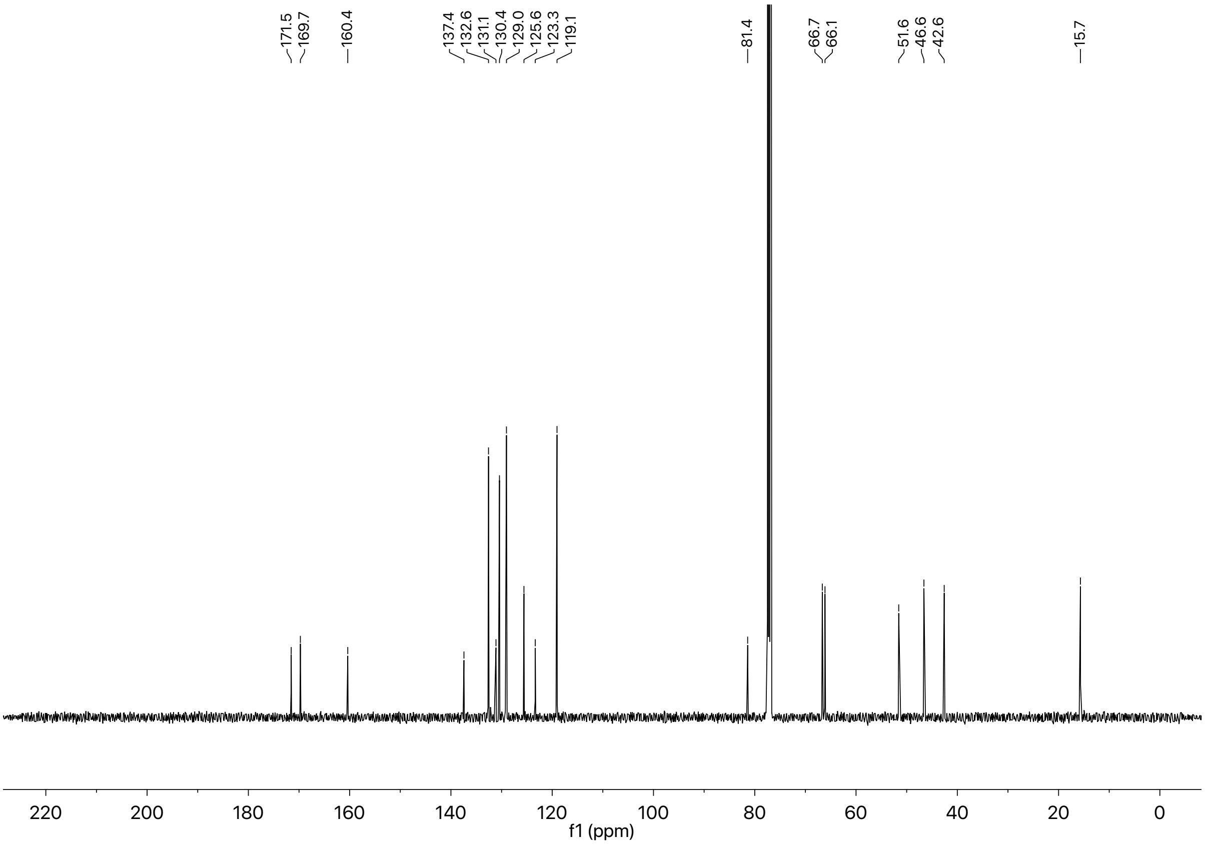

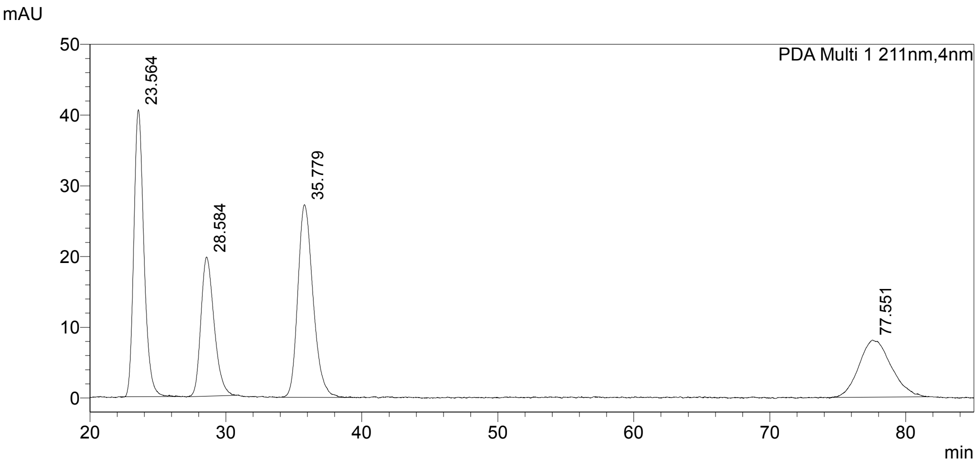

<Peak Table>

PDA Ch1 211nm

Peak#	Ret. Time	Area%
1	23.564	30.578
2	28.584	19.299
3	35.779	30.803
4	77.551	19.320
Total		100.000

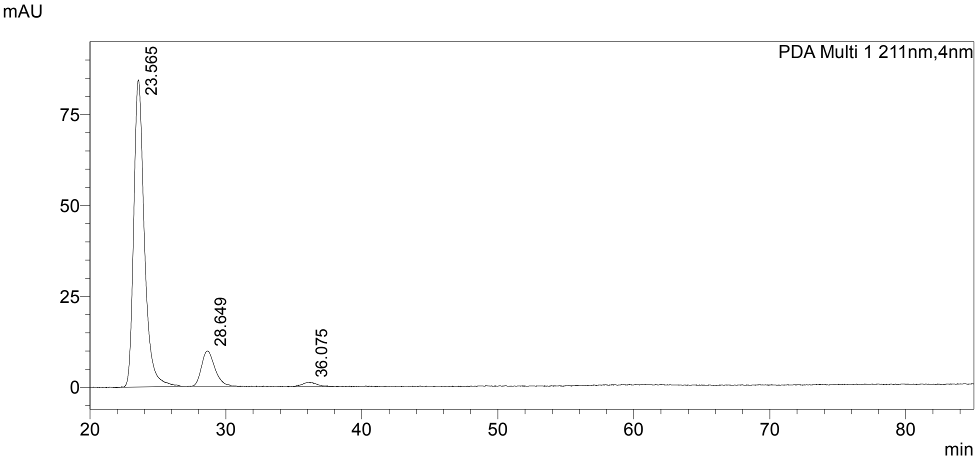

<Peak Table>

PDA Ch1 211nm

Peak#	Ret. Time	Area%
1	23.565	86.104
2	28.649	12.345
3	36.075	1.551
Total		100.000

5.8. (1′*R*,4*R*)- and (1′*S*,4*R*)-4-hydroxy-5-methyl-4-(2-morphline-2-oxo-1-(*p*-tolyl)ethyl)-2-phenyl-2,4-dihyro-3*H*-pyrazol-3-one **23**

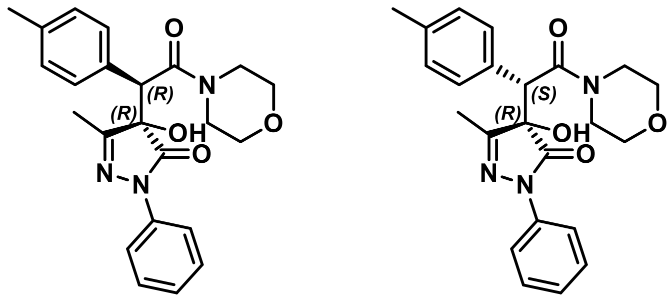

To a solution of 3-methyl-1-phenyl-1*H*-pyrazole-4,5-dione (47.1 mg, 0.25 mmol), 2-(*p*-tolyl)acetic anhydride (105.9 mg, 0.375 mmol) and (2*R*,3*S*)-HyperBTM (3.9 mg, 0.125 mmol) were dissolved in ethyl acetate (6 ml, 0.04 M) at 0 °C. Diisopropylethylamine (54 µl, 0.31 mmol) was added and the reaction mixture was stirred at 0 °C for 3 h. Morpholine (66 µl, 0.75 mmol) was added and the reaction mixture was stirred for 16 h at rt. The reaction mixture was diluted with ethyl acetate and washed sequentially with 1 M aq. HCl (×2), sat. aq. $NaHCO_3$ (×2), and brine (×1). The organic layer was dried over $MgSO_4$, filtered, and concentrated *in vacuo*. Purification by column chromatography (*n*-hexane : ethyl acetate 4:1 to 1:1) gave the title compound as a mixture of diastereomers as white solid (61.7 mg, 0.15 mmol, 61%, 91:9 d.r.). **mp** 197-199 °C (*dec*); $[\alpha]_D^{20}$ +253.9 (*c* 1.00, $CDCl_3$); **IR** ν_{max} (film) 3368 (O-H), 2965 (C-H), 2922 (C-H), 2857 (C-H), 1719 (C=O, pyrazolone), 1622, 1597, 1501, 1115, 758; **HRMS** (ESI^+) $C_{23}H_{24}N_3O_4Na$ $[M+Na]^+$ found 450.11791, requires 450.11910 (–2.6 ppm). *Data for major diastereomer*: **HPLC Analysis:** Chiralpak AD-H (80:20 hexane:isopropanol, flow rate 1.0 ml·min^{-1}, 211 nm, 30 °C) t_R (1′*R*,4*R*)-**23**: 10.7 min, t_R (1′*S*,4*S*)-**23**: 13.3 min, >99:1 er; **^{1}H NMR** (500 MHz, $CDCl_3$) δ_H: 1.96 (3H, s, C^5CH_3), 2.30 (3H, s, ArC^4CH_3), 3.09-3.19 (2H, m, $NCH_AH_BCH_2$, $NCH_2CH_AH_B$), 3.32 (1H, ddd, $^2J_{HH}$ = 14.0 Hz, $^3J_{HH}$ = 7.3 Hz, 3.0 Hz, $NCH_AH_BCH_2$), 3.48 (1H, ddd, $^2J_{HH}$ = 11.4 Hz, $^3J_{HH}$ = 6.3 Hz, 3.0 Hz, $NCH_2CH_AH_B$), 3.54-3.67 (2H, m, $NCH_CH_DCH_2$, $NCH_2CH_CH_D$), 3.69-3.78 (2H, m, $NCH_CH_DCH_2$, $NCH_2CH_CH_D$), 4.19 (1H, s, C*H*Ar), 6.33 (1H, br s, O*H*), 7.06-7.19 (5H, m, $CHArC^{2,3,5,6)}H$, $NArC^4H$), 7.32-7.40 (2H, m, $NArC^{3,5}H$), 7.72 (2H, d, $^3J_{HH}$ = 7.4 Hz, $NArC^{2,6}H$); **^{13}C{^{1}H} NMR** (126 MHz, $CDCl_3$) δ_C: 15.7 (C^5CH_3), 21.2 (ArC^4CH_3), 42.5 ($NCH_CH_DCH_2$), 46.5 ($NCH_AH_BCH_2$), 52.1 (*C*HAr), 66.1 ($NCH_2CH_AH_B$), 66.6 ($NCH_2CH_CH_D$), 81.7 (*C*-OH), 119.1 ($NArC^{2,6}H$), 125.4 ($NArC^4H$), 128.5 ($CHArC^{2,6}H$), 128.8 ($NArC^1$), 128.9 ($NArC^{3,5}H$), 130.0 ($CHArC^{3,5}H$), 137.5 ($CHArC^1$), 138.9 ($CHArC^4CH_3$), 160.9 (*C*=N), 170.4 ($CON(CH_2CH_2)_2O$), 171.8 (*C*ONAr); *Data for minor diastereomer*: **HPLC Analysis:** Chiralpak AD-H (80:20 hexane:isopropanol, flow rate 1.0 ml·min^{-1}, 211 nm, 30 °C) t_R (1′*S*,4*R*)-**23**: 9.6 min, t_R (1′*R*,4*S*)-**23**: 17.4 min, 99:1 er; **^{1}H NMR** (500 MHz, $CDCl_3$) (*selected*) δ_H: 2.01 (3H, s, C^5CH_3), 2.32 (3H, s, ArC^4CH_3), 3.04 (1H, ddd, $^2J_{HH}$ = 10.4 Hz, $^3J_{HH}$ = 7.3 Hz, 2.5 Hz, $NCH_2CH_AH_B$), 4.11 (1H, s, C*H*Ar);

$^{13}C\{^{1}H\}$ NMR (126 MHz, $CDCl_3$) (*selected*) δ_C: 14.9 (C^5CH_3), 42.3 (NCH_2CH_2), 52.5 (*C*HAr), 80.3 (*C*-OH), 119.0 (NAr$C^{2,6}$H), 125.1 (NArC^4H), 129.6 (Ar*C*H), 129.7 (Ar*C*H), 137.8 (CHArC^1), 138.6 (CHArC^4CH_3), 160.5 (*C*=N), 169.4 (*C*ON(CH_2CH_2)$_2$O), 171.1 (*C*ONAr).

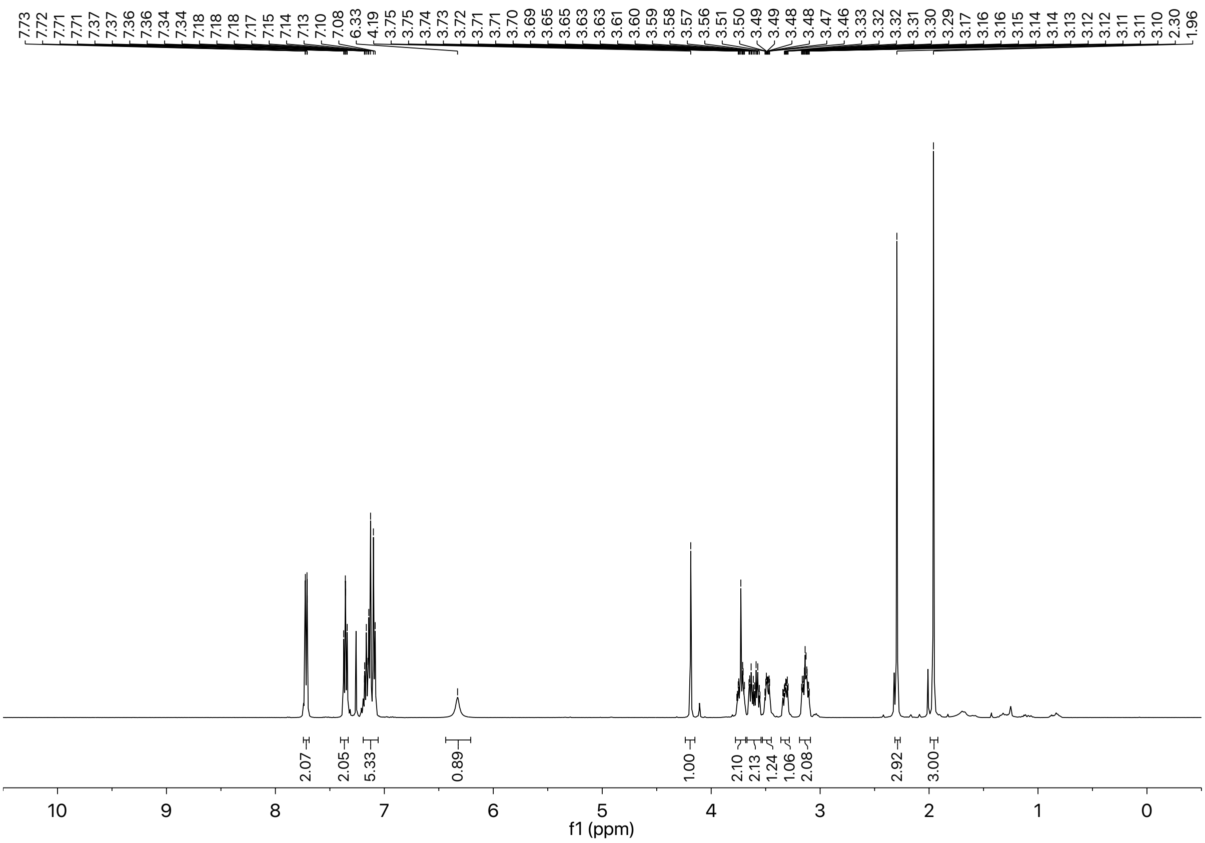

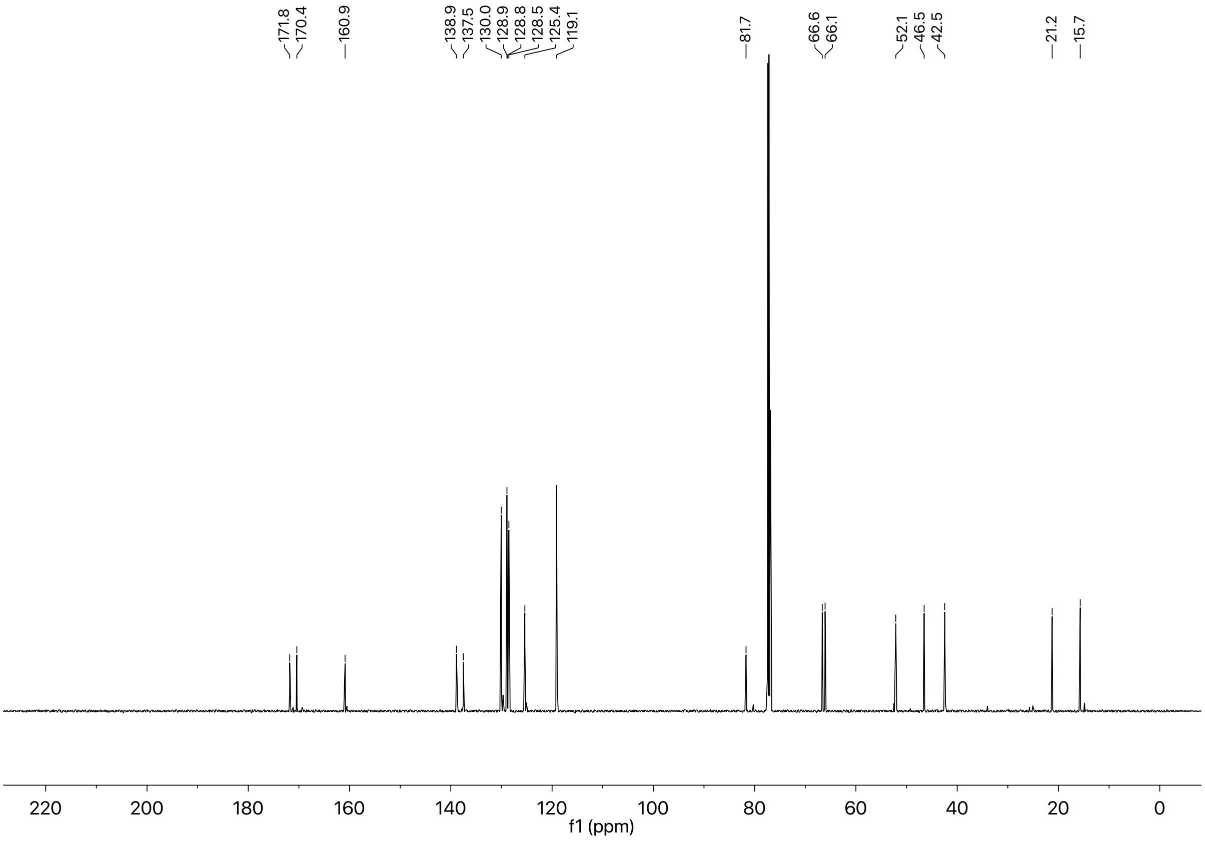

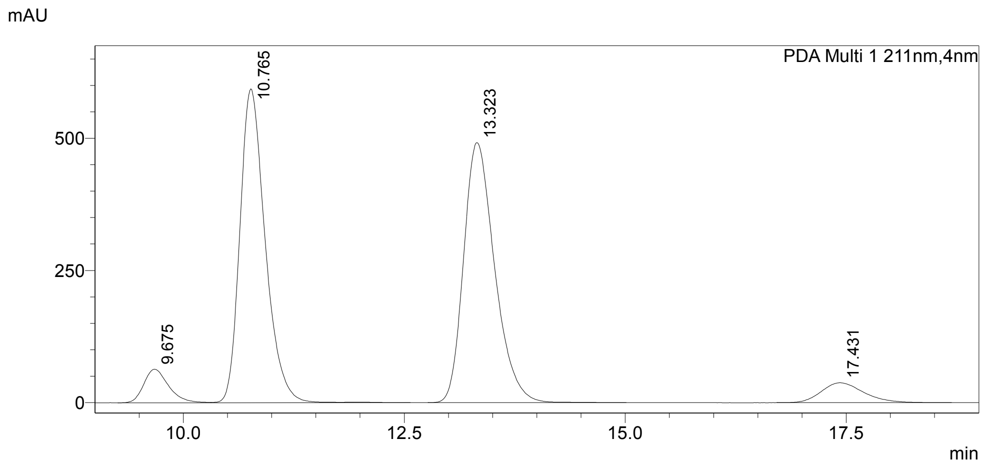

<Peak Table>

PDA Ch1 211nm

Peak#	Ret. Time	Area%
1	9.675	4.696
2	10.765	44.883
3	13.323	45.642
4	17.431	4.778
Total		100.000

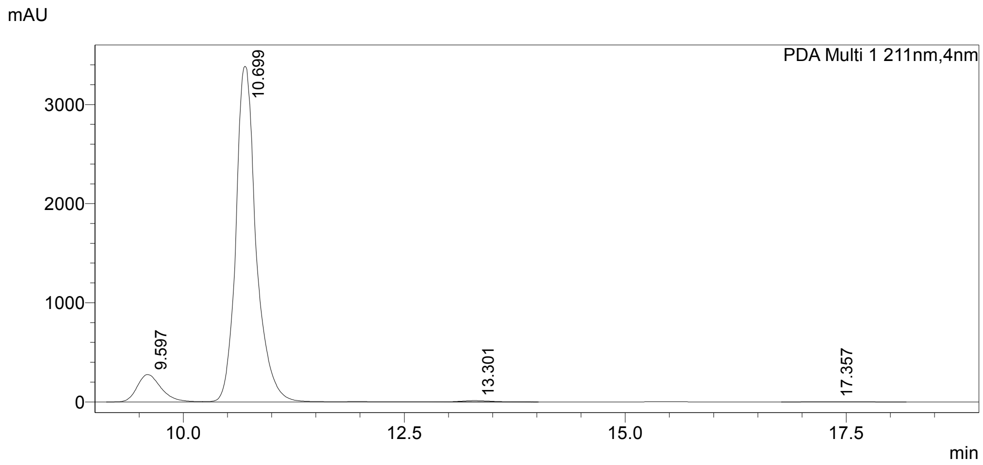

<Peak Table>

PDA Ch1 211nm

Peak#	Ret. Time	Area%
1	9.597	8.744
2	10.699	90.677
3	13.301	0.477
4	17.357	0.102
Total		100.000

5.9. (1′*R*,4*R*)-4-(1-(*p*-anisyl)-2-morpholine-2-oxoethyl)-4-hydroxy-5-methyl-2-phenyl-2,4-dihydro-3*H*-pyrazol-3-one **24**

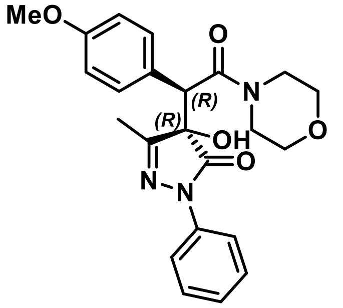

To a solution of 3-methyl-1-phenyl-1*H*-pyrazole-4,5-dione (47.1 mg, 0.25 mmol), 2-(*p*-anisyl)acetic anhydride (117.9 mg, 0.375 mmol) and (2*R*,3*S*)-HyperBTM (3.9 mg, 0.125 mmol) were dissolved in ethyl acetate (6 ml, 0.04 M) at 0 °C. Diisopropylethylamine (54 µl, 0.31 mmol) was added and the reaction mixture was stirred at 0 °C for 3 h. Morpholine (66 µl, 0.75 mmol) was added and the reaction mixture was stirred for 16 h at rt. The reaction mixture was diluted with ethyl acetate and washed sequentially with 1 M aq. HCl (×2), sat. aq. $NaHCO_3$ (×2), and brine (×1). The organic layer was dried over $MgSO_4$, filtered, and concentrated *in vacuo*. Purification by column chromatography (*n*-hexane : ethyl acetate 4:1 to 1:1) gave the title compound as a white solid (60.7 mg, 0.14 mmol, 57%). **mp** 193-195 °C (*dec*); $[\alpha]_D^{20}$ +147.0 (*c* 0.50, $CDCl_3$); **HPLC Analysis:** Chiralpak AD-H (85:15 hexane:isopropanol, flow rate 2.0 ml·min^{-1}, 211 nm, 30 °C) t_R (1′*R*,4*R*)-**24**: 9.8 min, t_R (1′*S*,4*S*)-**24**: 13.5 min, >99:1 er; **IR** ν_{max} (film) 3372 (O-H), 2963 (C-H), 2924 (C-H), 2855 (C-H), 2363, 1717 (C=O, pyrazolone), 1612, 1512, 1501, 1252, 1115, 758; **^{1}H NMR** (500 MHz, $CDCl_3$) δ_H: 1.96 (3H, s, CCH_3), 3.10-3.21 (2H, m, $NCH_AH_BCH_2$, $NCH_2CH_AH_B$), 3.33 (1H, ddd, $^2J_{HH}$ = 14.0 Hz, $^3J_{HH}$ = 7.4 Hz, 3.0 Hz, $NCH_AH_BCH_2$), 3.50 (1H, ddd, $^2J_{HH}$ = 11.3 Hz, $^3J_{HH}$ = 6.3 Hz, 3.0 Hz, $NCH_2CH_AH_B$), 3.55-3.67 (2H, m, $NCH_CH_DCH_2$, $NCH_2CH_CH_D$), 3.69-3.80 (5H, m, $NCH_CH_DCH_2$, $NCH_2CH_CH_D$, OCH_3), 4.17 (1H, s, C*H*Ar), 6.30 (1H, br s, O*H*), 6.81 (2H, app d, $^3J_{HH}$ = 8.7 Hz, $CHArC^{3,5}H$), 7.13-7.22 (3H, m, $NArC^4H$, $CHArC^{2,6}H$), 7.36 (3H, app t, $^3J_{HH}$ = 7.9 Hz, $NArC^{3,5}H$), 7.73 (2H, app d, $^3J_{HH}$ = 7.9 Hz, $NArC^{2,6}H$); **^{13}C{^{1}H} NMR** (126 MHz, $CDCl_3$) δ_C: 15.7 (CCH_3), 42.5 ($NCH_CH_DCH_2$), 46.6 ($NCH_AH_BCH_2$), 51.6 (*C*HAr), 55.4 (OCH_3), 66.1 ($NCH_2CH_AH_B$), 66.7 ($NCH_2CH_CH_D$), 81.7 (*C*-OH), 114.7 ($CHArC^{3,5}H$), 119.1 ($NArC^{2,6}H$), 123.7 ($CHArC^1$), 125.4 ($NArC^4H$), 128.9 ($NArC^{3,5}H$), 129.9 ($CHArC^{2,6}H$), 137.6 ($NArC^1$), 159.9 ($CHArC^4OCH_3$), 160.9 (*C*=N), 170.5 ($CON(CH_2CH_2)_2O$), 171.8 (*C*ONAr); **HRMS** (ESI$^+$) $C_{23}H_{24}N_3O_5Na$ $[M+Na]^+$ found 446.16763, requires 446.16864 (–2.3 ppm).

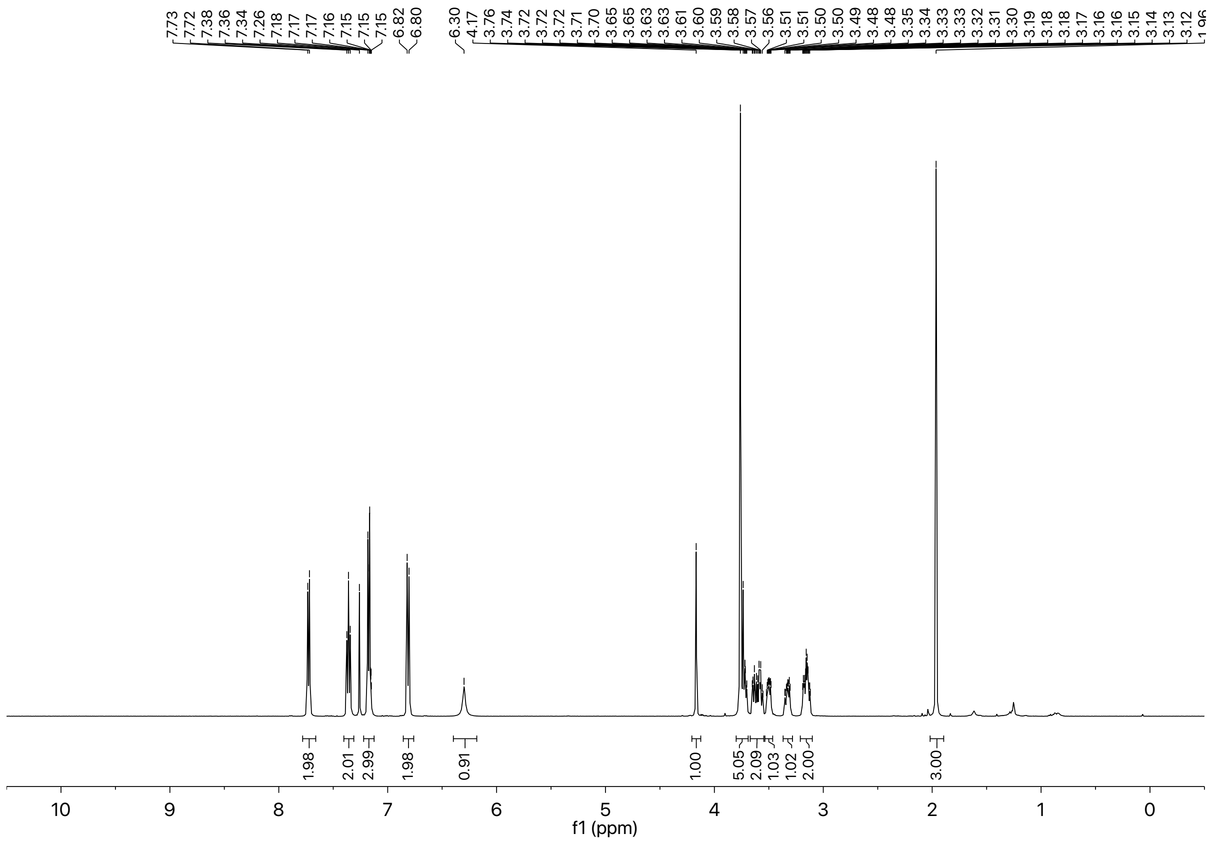
7.73
7.72
7.38
7.36
7.34
7.26
7.18
7.17
7.17
7.16
7.15
7.15
7.15
6.82
6.80
6.30
4.17
3.76
3.74
3.72
3.72
3.72
3.71
3.70
3.65
3.65
3.63
3.63
3.61
3.60
3.59
3.58
3.57
3.56
3.51
3.51
3.50
3.50
3.49
3.48
3.48
3.35
3.34
3.33
3.33
3.32
3.31
3.30
3.19
3.18
3.18
3.17
3.16
3.16
3.15
3.14
3.13
3.12
1.98
2.01
2.99
1.98
0.91
1.00
5.05
2.09
1.03
1.02
2.00
3.00
10
9
8
7
6
5
4
3
2
1
0
f1 (ppm)

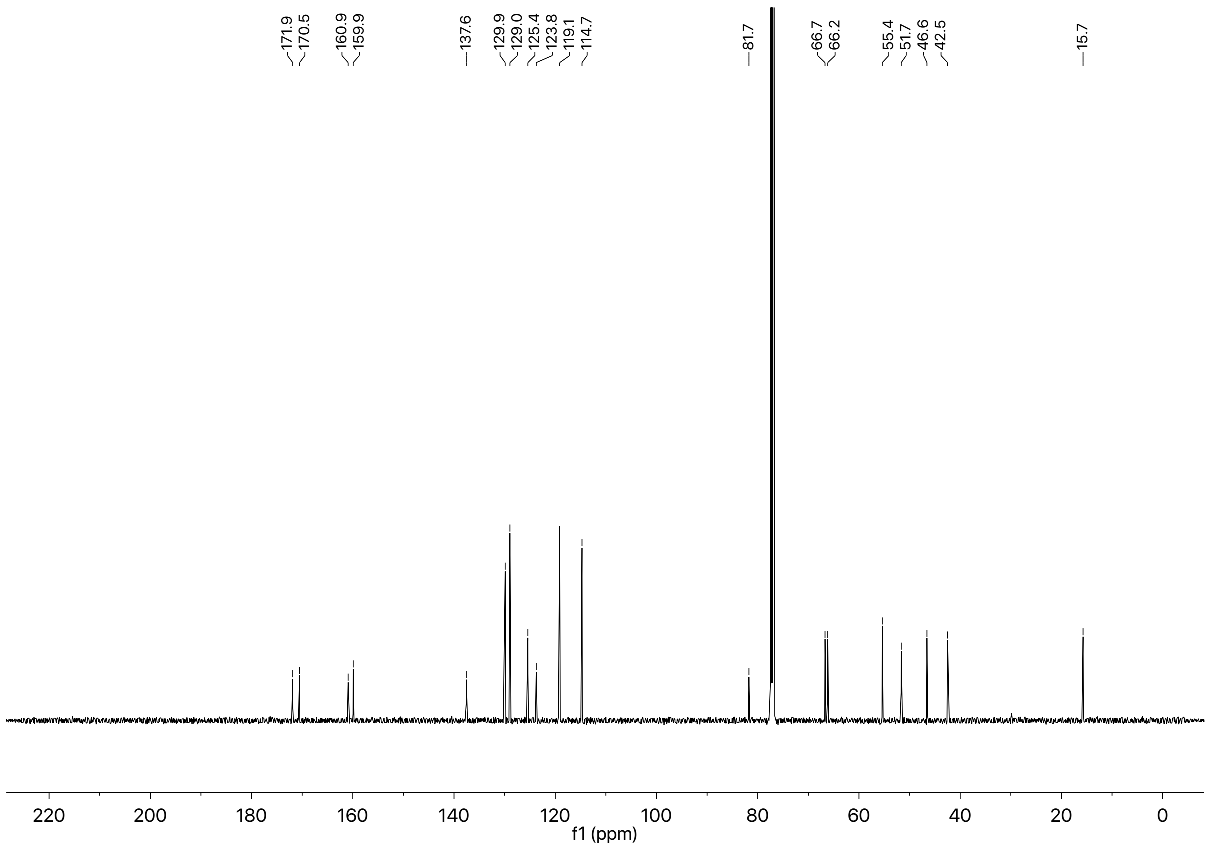
171.9
170.5
160.9
159.9
137.6
129.9
129.0
125.4
123.8
119.1
114.7
81.7
66.7
66.2
55.4
51.7
46.6
42.5
15.7
220
200
180
160
140
120
100
80
60
40
20
0
f1 (ppm)

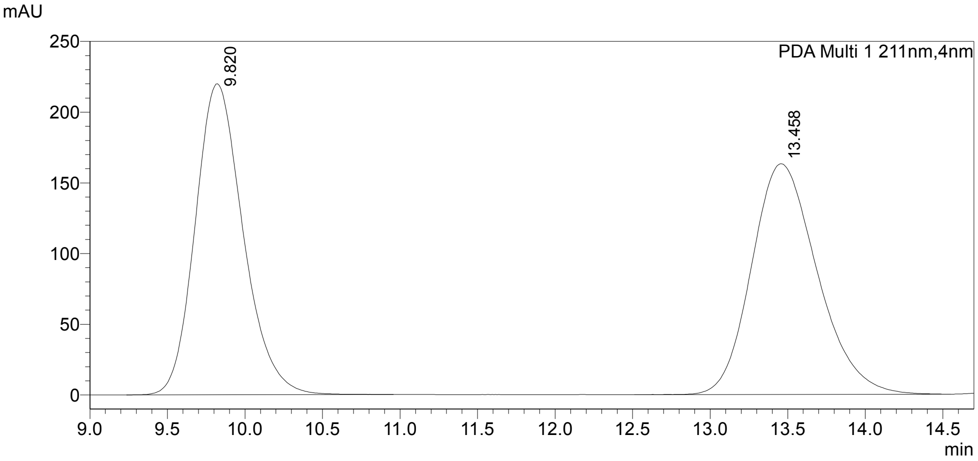

<Peak Table>

PDA Ch1 211nm

Peak#	Ret. Time	Area%
1	9.820	49.597
2	13.458	50.403
Total		100.000

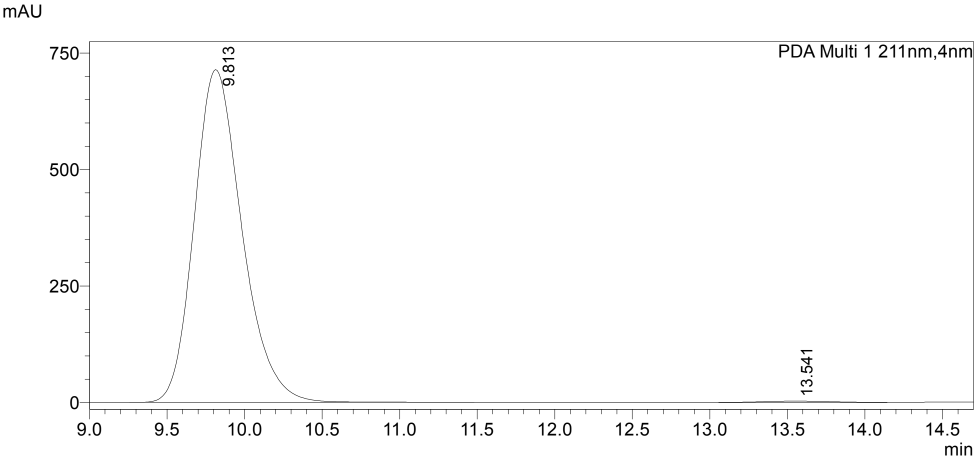

<Peak Table>

PDA Ch1 211nm

Peak#	Ret. Time	Area%
1	9.813	99.462
2	13.541	0.538
Total		100.000

5.10. (1′*R*,4*R*)- and (1′*S*,4*R*)-4-(1-(*m*-bromophenyl)-2-morpholine-2-oxoethyl)-4-hydroxy-5-methyl-2-phenyl-2,4-dihydro-3*H*-pyrazol-3-one **25**

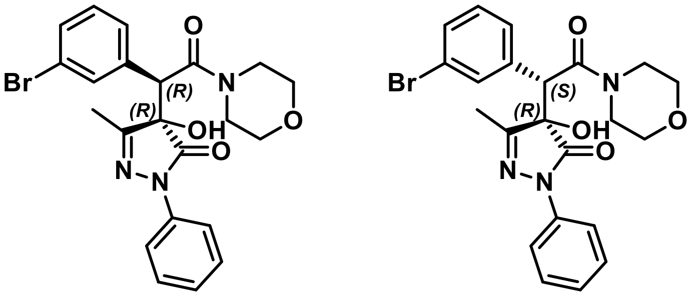

To a solution of 3-methyl-1-phenyl-1*H*-pyrazole-4,5-dione (47.1 mg, 0.25 mmol), 2-(*m*-bromophenyl)acetic anhydride (154.5 mg, 0.375 mmol) and (2*R*,3*S*)-HyperBTM (3.9 mg, 0.125 mmol) were dissolved in ethyl acetate (6 ml, 0.04 M) at 0 °C. Diisopropylethylamine (54 µl, 0.31 mmol) was added and the reaction mixture was stirred at 0 °C for 3 h. Morpholine (66 µl, 0.75 mmol) was added and the reaction mixture was stirred for 16 h at rt. The reaction mixture was diluted with ethyl acetate and washed sequentially with 1 M aq. HCl (×2), sat. aq. $NaHCO_3$ (×2), and brine (×1). The organic layer was dried over $MgSO_4$, filtered, and concentrated *in vacuo*. Purification by column chromatography (*n*-hexane : ethyl acetate 4:1 to 1:1) gave the title compound as a mixture of diastereomer as a white amorphous solid (44.2 mg, 0.09 mmol, 37%, 89:11 d.r.). $[\alpha]_D^{20}$ +153.3 (*c* 0.50, $CDCl_3$); **IR** ν_{max} (film) 3323 (O-H), 3057 (C-H), 2970 (C-H), 2922 (C-H), 2857 (C-H), 1715 (C=O, pyrazolone), 1622, 1595, 1364, 1113, 758; **HRMS** (ESI^+) $C_{22}H_{21}BrN_3O_4Na$ $[M(^{81}Br)+Na]^+$ found 496.06554, requires 496. 06684 (–2.6 ppm). *Data for major diastereomer*: **HPLC Analysis:** Chiralpak AD-H (85:15 hexane:isopropanol, flow rate 2.0 ml·min^{-1}, 211 nm, 30 °C) t_R (1′*R*,4*R*)-**25**: 7.1 min, t_R (1′*S*,4*S*)-**25**: 11.8 min, 98.5:1.5 er; **^{1}H NMR** (500 MHz, $CDCl_3$) δ_H: 1.93 (3H, s, CH_3), 3.16 (1H, ddd, $^2J_{HH}$ = 13.3 Hz, $^3J_{HH}$ = 6.4 Hz, 3.0 Hz, NCH_AH$_B$CH$_2$), 3.22 (1H, ddd, $^2J_{HH}$ = 11.5 Hz, $^3J_{HH}$ = 6.6 Hz, 3.0 Hz, NCH$_2$CH_AH$_B$), 3.35 (1H, ddd, $^2J_{HH}$ = 13.3 Hz, $^3J_{HH}$ = 6.6 Hz, 3.0 Hz, NCH$_A$$H_BCH_2$), 3.54 (1H, ddd, $^2J_{HH}$ = 11.5 Hz, $^3J_{HH}$ = 6.4 Hz, 3.0 Hz, NCH$_2$CH$_A$$H_B$), 3.61 (1H, ddd, $^2J_{HH}$ = 11.1 Hz, $^3J_{HH}$ = 5.6 Hz, 3.6 Hz, NCH$_2$CH_CH$_D$), 3.68-3.77 (3H, m, NC$H_C$$H_DCH_2$, NCH$_2CH_C$$H_D$), 4.16 (1H, s, C*H*Ar), 6.29 (1H, br s, O*H*), 7.16-7.25 (3H, m, NArC^4*H*, 2×CHArC*H*), 7.35-7.40 (2H, m, NArC^{3,5}*H*), 7.44-7.49 (2H, m, 2×CHArC*H*), 7.70-7.75 (2H, m, NArC^{2,6}*H*); **^{13}C{^{1}H} NMR** (126 MHz, $CDCl_3$) δ_C: 15.6 (CH_3), 42.6 (N*C*H$_C$H$_D$CH$_2$), 46.6 (N*C*H$_A$H$_B$CH$_2$), 51.6 (*C*HAr), 66.1 (NCH$_2$*C*H$_A$H$_B$), 66.7 (NCH$_2$*C*H$_C$H$_D$), 81.5 (*C*-OH), 119.2 (NAr*C*^{2,6}H), 123.3 (CHAr*C*^{3}Br), 125.6 (NAr*C*^{4}H), 127.4 (CHAr*C*H), 129.0 (NAr*C*^{3,5}H), 130.8 (CHAr*C*H), 131.8 (CHAr*C*H), 132.2 (CHAr*C*H), 134.3 (CHAr*C*^{1}), 137.3 (NAr*C*^{1}), 160.3 (*C*=N), 169.5 (*C*ON(CH$_2$CH$_2$)$_2$O), 171.5 (*C*ONAr); *Data for minor diastereomer*: **HPLC Analysis:** Chiralpak AD-H (85:15 hexane:isopropanol, flow rate 2.0 ml·min^{-1}, 211 nm, 30 °C) t_R (1′*S*,4*R*)-**25**: 6.3 min, t_R (1′*R*,4*S*)-**25**: 15.7 min, 97.5:2.5 er; **^{1}H NMR** (500 MHz, $CDCl_3$) (*selected*) δ_H: 1.96

(3H, s, CH_3), 4.14 (1H, s, C*H*Ar), 7.54 (1H, m, ArC*H*), 7.74-7.78 (1H, m, NAr$C^{2,6}$H); **^{13}C{^{1}H} NMR** (126 MHz, $CDCl_3$) (*selected*) δ_C: 15.0 (*C*H_3), 42.4 (N*C*H_2CH_2), 46.5 (N*C*H_2CH_2), 52.4 (*C*HAr), 80.0 (*C*-OH), 118.9 (NAr$C^{2,6}$H), 122.9 (CHArC^3Br), 125.3 (NArC^4H), 128.5 (Ar*C*H), 128.9 (NAr$C^{3,5}$H), 130.3 (Ar*C*H), 131.9 (Ar*C*H), 132.8 (Ar*C*H), 134.2 (CHArC^1), 137.7 (NArC^1), 168.5 (*C*ON(CH_2CH_2)$_2$O), 171.0 (*C*ONAr).

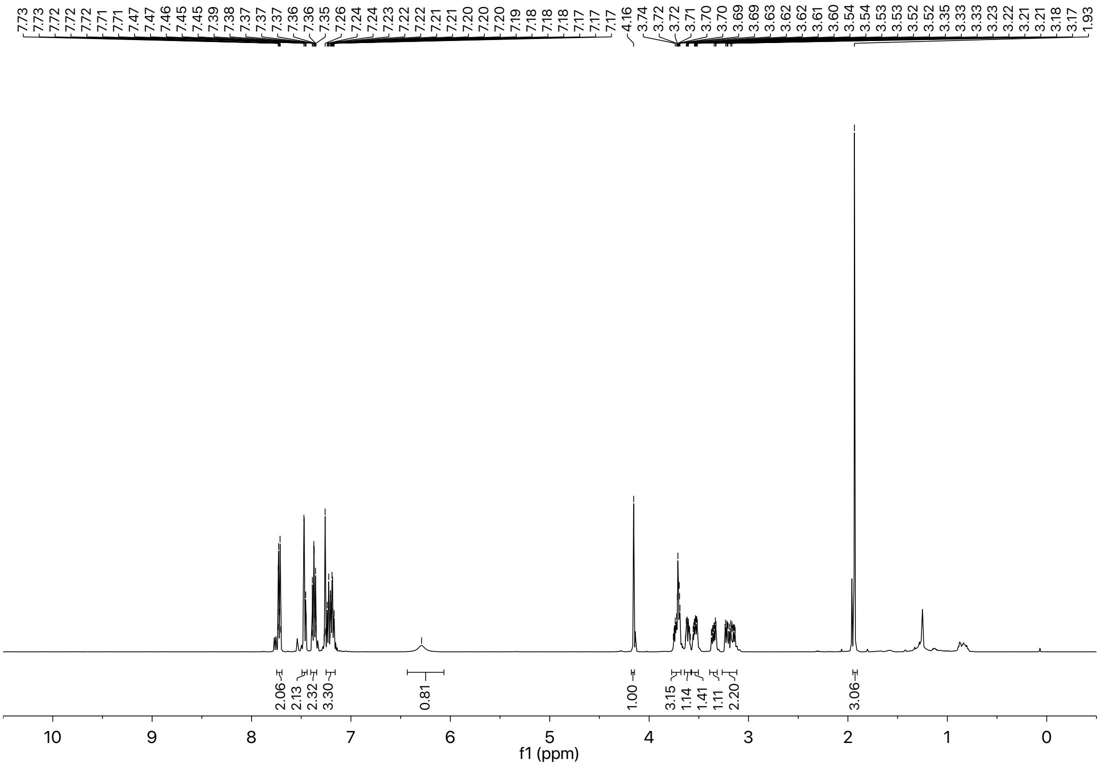

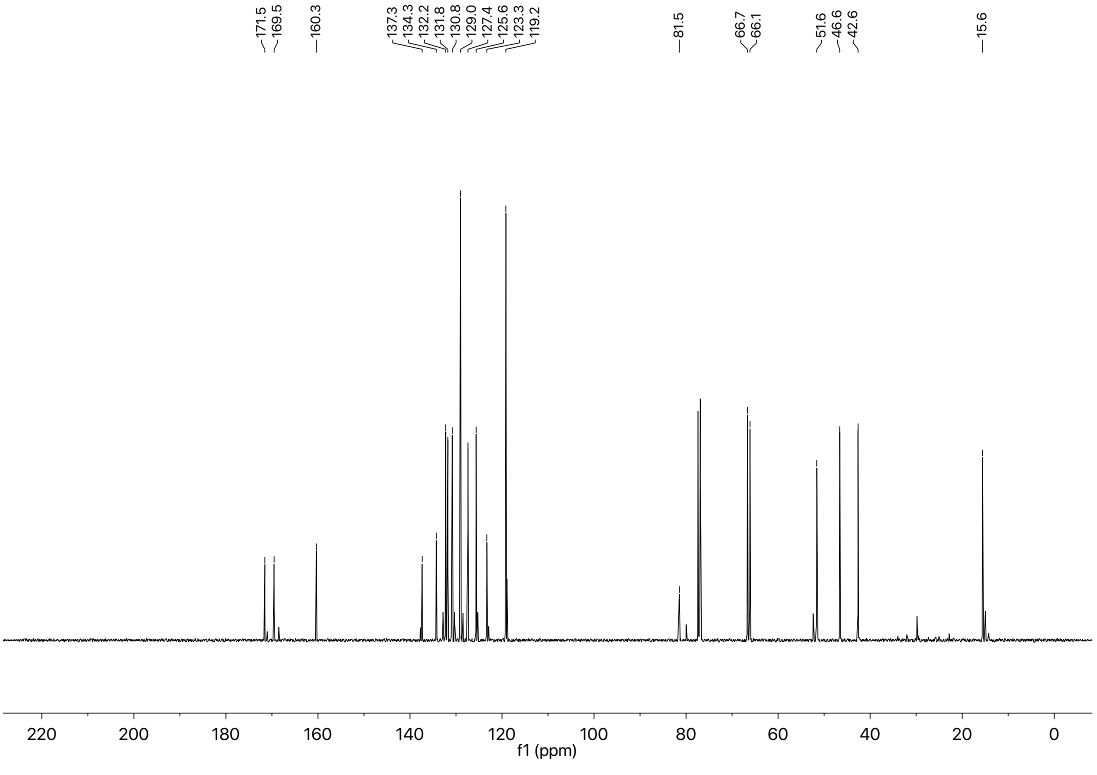

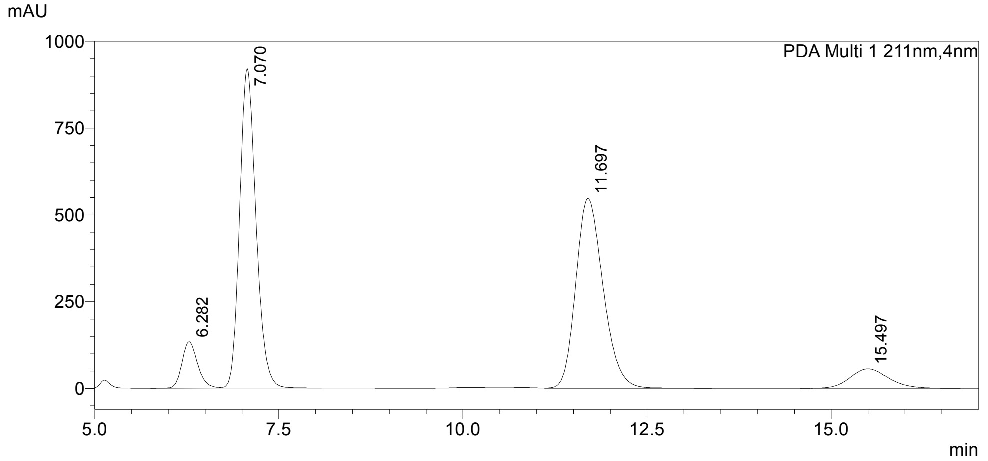

<Peak Table>

PDA Ch1 211nm

Peak#	Ret. Time	Area%
1	6.282	6.111
2	7.070	43.961
3	11.697	43.693
4	15.497	6.235
Total		100.000

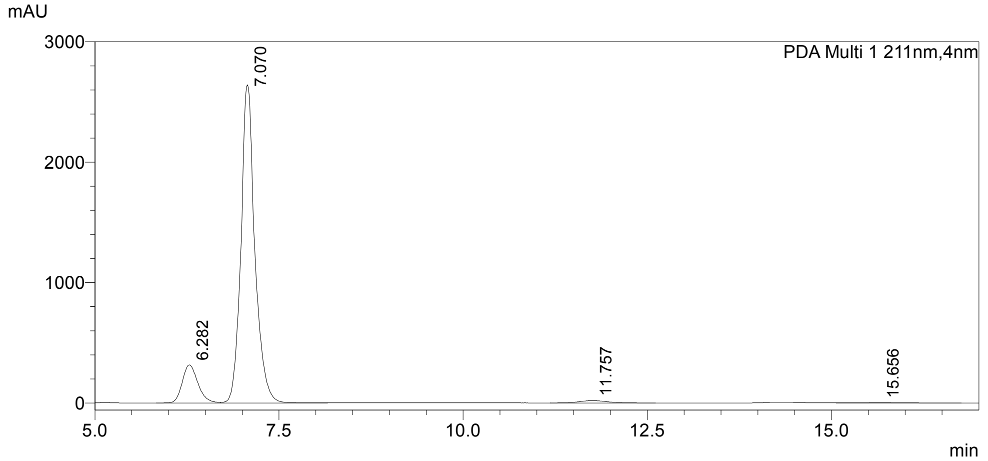

<Peak Table>

PDA Ch1 211nm

Peak#	Ret. Time	Area%
1	6.282	11.638
2	7.070	86.773
3	11.757	1.262
4	15.656	0.327
Total		100.000

5.11. (1'*R*,4*R*)- and (1'*S*,4*R*)-4-hydroxy-5-methyl-4-(2-morpholino-2-oxo-1-(*o*-tolyl)ethyl)-2-phenyl-2,4-dihydro-3*H*-pyrazol-3-one **26**

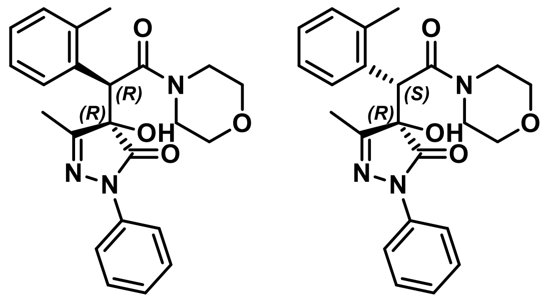

To a solution of 3-methyl-1-phenyl-1*H*-pyrazole-4,5-dione (47.1 mg, 0.25 mmol), 2-(*o*-tolyl)acetic anhydride (105.9 mg, 0.375 mmol) and (2*R*,3*S*)-HyperBTM (3.9 mg, 0.125 mmol) were dissolved in ethyl acetate (6 ml, 0.04 M) at 0 °C. Diisopropylethylamine (54 µl, 0.31 mmol) was added and the reaction mixture was stirred at 0 °C for 3 h. Morpholine (66 µl, 0.75 mmol) was added and the reaction mixture was stirred for 16 h at rt. The reaction mixture was diluted with ethyl acetate and washed sequentially with 1 M aq. HCl (×2), sat. aq. $NaHCO_3$ (×2), and brine (×1). The organic layer was dried over $MgSO_4$, filtered, and concentrated *in vacuo*. Purification by column chromatography (*n*-hexane : ethyl acetate 4:1 to 1:1) gave the title compound as a mixture of diastereomers as white amorphous solid (98.0 mg, 0.24 mmol, 96%, >95:5 d.r.). $[\alpha]_D^{20}$ +238.7 (*c* 0.82, $CHCl_3$); **IR** ν_{max} (film) 3312 (O-H), 2965 (C-H), 2922 (C-H), 2857 (C-H), 2249, 1717 (C=O, pyrazolone), 1639, 1620, 1597, 1499, 1435, 1360, 1225, 1113, 908, 754; **HRMS** (ESI^+) $C_{23}H_{26}N_3O_4$ $[M+H]^+$ found 408.1910, requires 408.19178 (–1.9 ppm). *Data for major diastereomer*: **HPLC Analysis:** Chiralpak AD-H (95:5 hexane:isopropanol, flow rate 2.0 ml·min^{-1}, 211 nm, 30 °C) t_R (1'*R*,4*R*)-**26**: 16.9 min, t_R (1'*S*,4*S*)-**26**: 36.0 min, >99:1 er; **^{1}H NMR** (500 MHz, $CDCl_3$) δ_H: 1.61 (3H, s, C^5CH_3), 2.30 (3H, s, ArC^2CH_3), 2.98 (1H, ddd, $^2J_{HH}$ = 10.9 Hz, $^3J_{HH}$ = 7.4 Hz, 2.9 Hz, $NCH_2CH_AH_B$), 3.08 (1H, ddd, $^2J_{HH}$ = 13.5 Hz, $^3J_{HH}$ = 5.8 Hz, 2.9 Hz, $NCH_AH_BCH_2$), 3.31 (1H, ddd, $^2J_{HH}$ = 13.5 Hz, $^3J_{HH}$ = 7.4 Hz, 3.0 Hz, $NCH_AH_BCH_2$), 3.45-3.54 (2H, m, $NCH_2CH_AH_B$, $NCH_2CH_CH_D$), 3.59 (1H, ddd, $^2J_{HH}$ = 13.2 Hz, $^3J_{HH}$ = 7.5 Hz, 2.9 Hz, $NCH_CH_DCH_2$), 3.72 (1H, ddd, $^2J_{HH}$ = 11.6 Hz, $^3J_{HH}$ = 5.8 Hz, 2.9 Hz, $NCH_2CH_CH_D$), 3.79 (1H, ddd, $^2J_{HH}$ = 13.2 Hz, $^3J_{HH}$ = 5.8 Hz, 2.8 Hz, $NCH_CH_DCH_2$), 4.21 (1H, s, *CH*Ar), 7.16-7.21 (2H, m, $NArC^4H$, $CHArC^3H$), 7.26-7.30 (2H, m, $CHArC^{4,5}H$), 7.36-7.44 (3H, m, O*H*, $NArC^{3,5}H$), 7.61-7.68 (1H, m, $CHArC^6H$), 7.80-7.87 (2H, m, $NArC^{2,6}H$); **^{13}C{^{1}H} NMR** (126 MHz, $CDCl_3$) δ_C: 14.5 (C^5CH_3), 20.0 (ArC^2CH_3), 42.8 ($NCH_CH_DCH_2$), 44.8 (*C*HAr), 46.2 ($NCH_AH_BCH_2$), 66.0 ($NCH_2CH_AH_B$), 66.6 ($NCH_CH_DCH_2$), 81.0 (*C*-OH), 118.8 ($NArC^{2,6}H$), 125.2 ($NArC^4H$), 127.2 ($CHArC^5H$), 129.0 ($NArC^{3,5}H$, $CHArC^4H$), 129.6 ($CHArC^6H$), 130.5 ($CHArC^1$), 131.2 ($CHArC^3H$), 135.6 ($CHArC^2CH_3$), 137.9 ($NArC^1$), 160.7 (*C*=N), 170.8 ($CON(CH_2CH_2)_2O$), 173.0 (*C*ONAr); *Data for minor diastereomer*: **HPLC Analysis:** Chiralpak AD-H (95:5 hexane:isopropanol, flow rate

2.0 ml·min^{-1}, 211 nm, 30 °C) t_R (1′*S*,4*R*)-**26**: 24.2 min, t_R (1′*R*,4*S*)-**26**: 41.0 min (not detected); **^{1}H NMR** (500 MHz, $CDCl_3$) (*selected*) δ_H: 2.15 (3H, s, C^5CH_3), 2.25 (3H, s, ArC^2CH_3), 2.79 (1H, ddd, $^2J_{HH}$ = 11.2 Hz, $^3J_{HH}$ = 7.9 Hz, 2.8 Hz, NCH_2CH_2), 3.23 (1H, ddd, $^2J_{HH}$ = 11.9 Hz, $^3J_{HH}$ = 7.8 Hz, 3.0 Hz, NCH_2CH_2), 4.12 (1H, s, C*H*Ar); **^{13}C{^{1}H} NMR** (126 MHz, $CDCl_3$) (*selected*) δ_C: 15.4 (C^5CH_3), 42.6 (N*C*H_2CH_2), 46.0 (NCH_2*C*H_2), 47.0 (*C*HAr), 80.9 (*C*-OH), 118.6 ($NArC^{2,6}H$), 125.1 ($NArC^4H$), 126.9 (Ar*C*H), 128.8 (Ar*C*H).

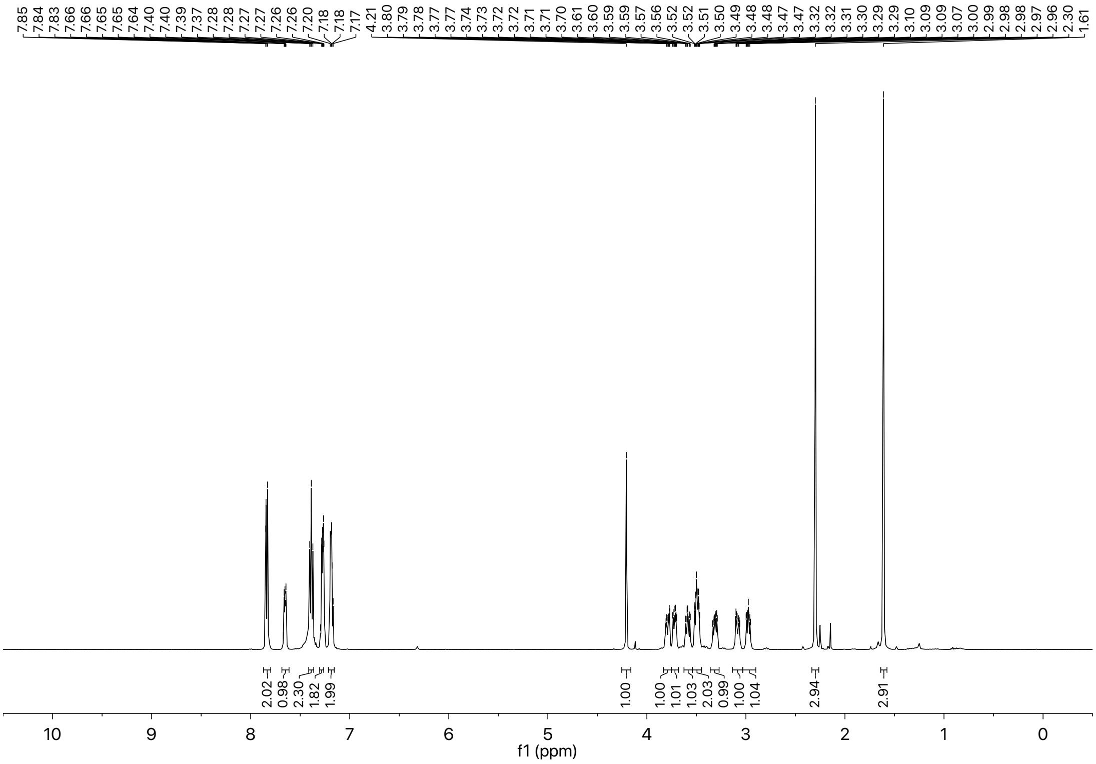

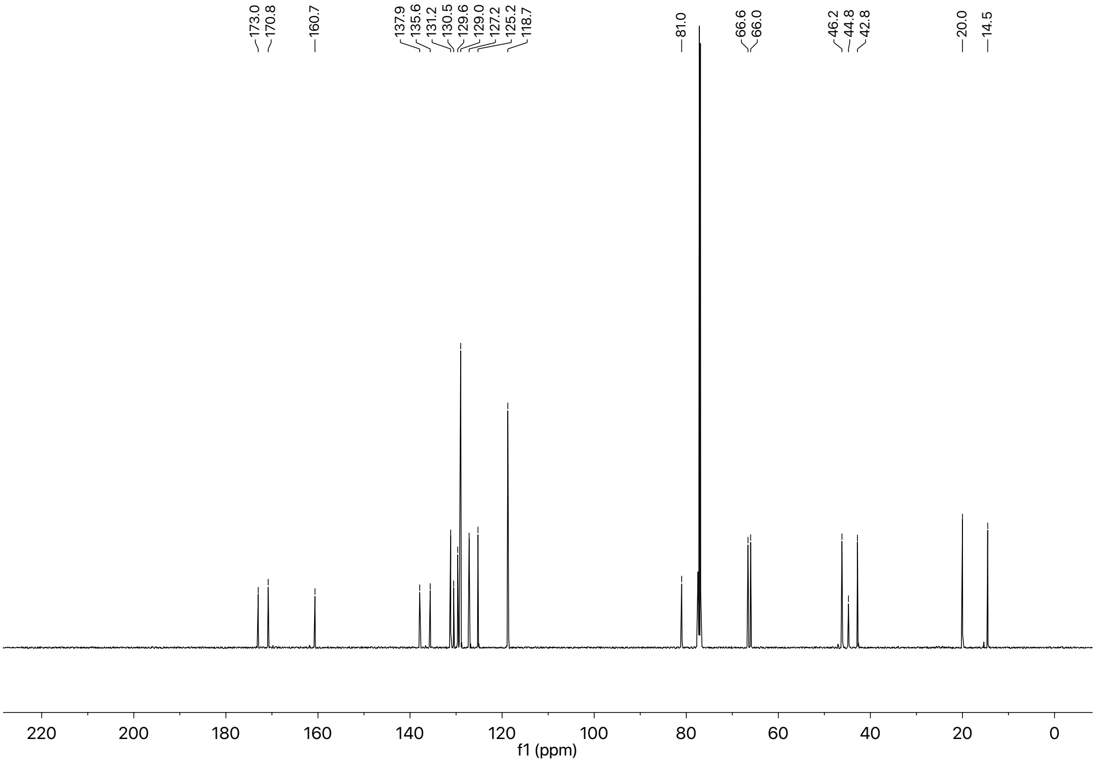

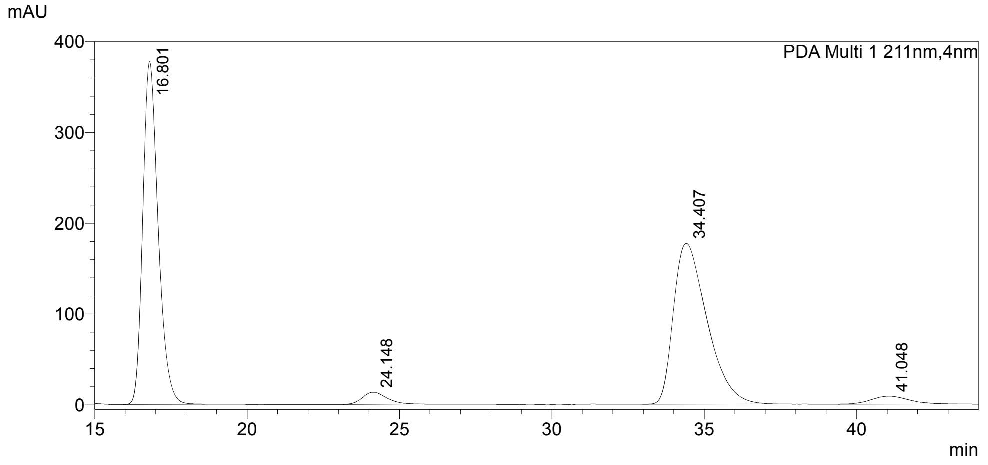

<Peak Table>

PDA Ch1 211nm

Peak#	Ret. Time	Area%
1	16.801	46.799
2	24.148	2.575
3	34.407	47.943
4	41.048	2.683
Total		100.000

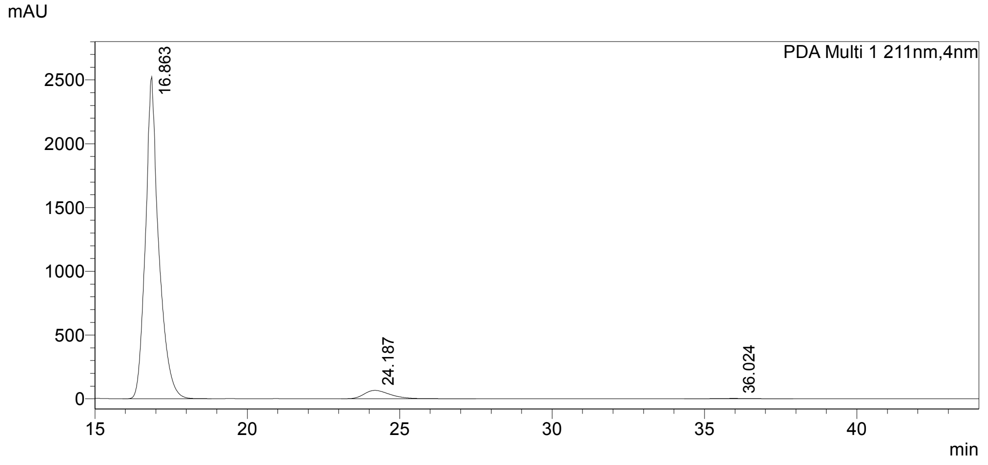

<Peak Table>

PDA Ch1 211nm

Peak#	Ret. Time	Area%
1	16.863	94.867
2	24.187	4.897
3	36.024	0.236
Total		100.000

5.12. (1′*R*,4*R*)-4-hydroxy-5-methyl-4-(1-(1-methyl-1*H*-indol-3-yl)-2-morpholino-2-oxoethyl)-2-phenyl-2,4-dihydro-3*H*-pyrazol-3-one **27**

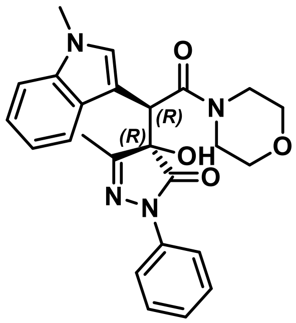

To a solution of 3-methyl-1-phenyl-1*H*-pyrazole-4,5-dione (47.1 mg, 0.25 mmol), 2-(1-methyl-1*H*-indol-3-yl)acetic anhydride (135.2 mg, 0.375 mmol) and (2*R*,3*S*)-HyperBTM (3.9 mg, 0.125 mmol) were dissolved in ethyl acetate (6 ml, 0.04 M) at 0 °C. Diisopropylethylamine (54 µl, 0.31 mmol) was added and the reaction mixture was stirred at 0 °C for 3 h. Morpholine (66 µl, 0.75 mmol) was added and the reaction mixture was stirred for 16 h at rt. The reaction mixture was diluted with ethyl acetate and washed sequentially with 1 M aq. HCl (×2), sat. aq. $NaHCO_3$ (×2), and brine (×1). The organic layer was dried over $MgSO_4$, filtered, and concentrated *in vacuo*. Purification by column chromatography (*n*-hexane : ethyl acetate 4:1 to 1:1) gave the title compound as white amorphous solid (80.6 mg, 0.18 mmol, 72%). $[\alpha]_D^{20}$ +262.9 (*c* 0.48, $CHCl_3$); **HPLC Analysis:** Chiralpak AD-H (85:15 hexane:isopropanol, flow rate 2.0 ml·min^{-1}, 211 nm, 30 °C) t_R (1′*R*,4*R*)-**27**: 7.1 min, t_R (1′*S*,4*S*)-**27**: 22.9 min, 96:4 er; **IR** ν_{max} (film) 3354 (O-H), 3059, 2922 (C-H), 2859 (C-H), 2247, 1717 (C=O, pyrazolone), 1620, 1595, 1501, 1362, 1115, 908; **^{1}H NMR** (500 MHz, $CDCl_3$) δ_H: 1.81 (3H, s, C^5CH_3), 3.11 (1H, ddd, $^2J_{HH}$ = 11.4 Hz, $^3J_{HH}$ = 6.5 Hz, 3.0 Hz, $NCH_2CH_AH_B$), 3.19 (1H, ddd, $^2J_{HH}$ = 13.4 Hz, $^3J_{HH}$ = 6.6 Hz, 3.0 Hz, $NCH_AH_BCH_2$), 3.40 (1H, ddd, $^2J_{HH}$ = 13.4 Hz, $^3J_{HH}$ = 6.5 Hz, 3.0 Hz, $NCH_AH_BCH_2$), 3.46 (1H, ddd, $^2J_{HH}$ = 11.4 Hz, $^3J_{HH}$ = 6.6 Hz, 3.0 Hz, $NCH_2CH_AH_B$), 3.56-3.63 (1H, m, $NCH_2CH_CH_D$), 3.64-3.77 (6H, m, $NCH_CH_DCH_2$, $NCH_2CH_CH_D$, NCH_3), 4.48 (1H, s, C*H*Ar), 6.61 (1H, br s, O*H*), 7.12 (1H, t, $^3J_{HH}$ = 7.5 Hz, $CHArC^5H$), 7.14-7.20 (2H, m, $NArC^4H$, $CHArC^2H$), 7.24 (1H, t, $^3J_{HH}$ = 7.5 Hz, $CHArC^6H$), 7.31 (1H, d, $^3J_{HH}$ = 8.2 Hz, $CHArC^7H$), 7.36 (2H, app t, $^3J_{HH}$ = 8.0 Hz, $NArC^{3,5}H$), 7.50 (1H, d, $^3J_{HH}$ = 8.0 Hz, $CHArC^4H$), 7.74 (2H, d, $^3J_{HH}$ = 7.9 Hz, $NArC^{2,6}H$); **^{13}C{^{1}H} NMR** (126 MHz, $CDCl_3$) δ_C: 15.3 (C^5CH_3), 33.2 (NCH_3), 42.5 ($NCH_CH_DCH_2$), 42.6 (*C*HAr), 46.6 ($NCH_AH_BCH_2$), 66.2 ($NCH_2CH_AH_B$), 66.6 ($NCH_2CH_CH_D$), 81.3 (*C*-OH), 104.3 ($CHArC^3$), 110.0 ($CHArC^7H$), 117.8 ($CHArC^4H$), 119.0 ($NArC^{2,6}H$), 120.3 ($CHArC^5H$), 122.5 ($CHArC^6H$), 125.3 ($NArC^4H$), 126.6 ($CHArC^{3a}$), 128.9 ($CHArC^2H$), 129.1 ($NArC^{3,5}H$), 136.5 ($CHArC^{7a}$), 137.7 ($NArC^1$), 161.3 (*C*=N), 171.2 ($CON(CH_2CH_2)_2O$), 172.6 (*C*ONAr); **HRMS** (ESI$^+$) $C_{25}H_{27}N_4O_4$ $[M+H]^+$ found 447.2018, requires 447.20275 (–1.9 ppm).

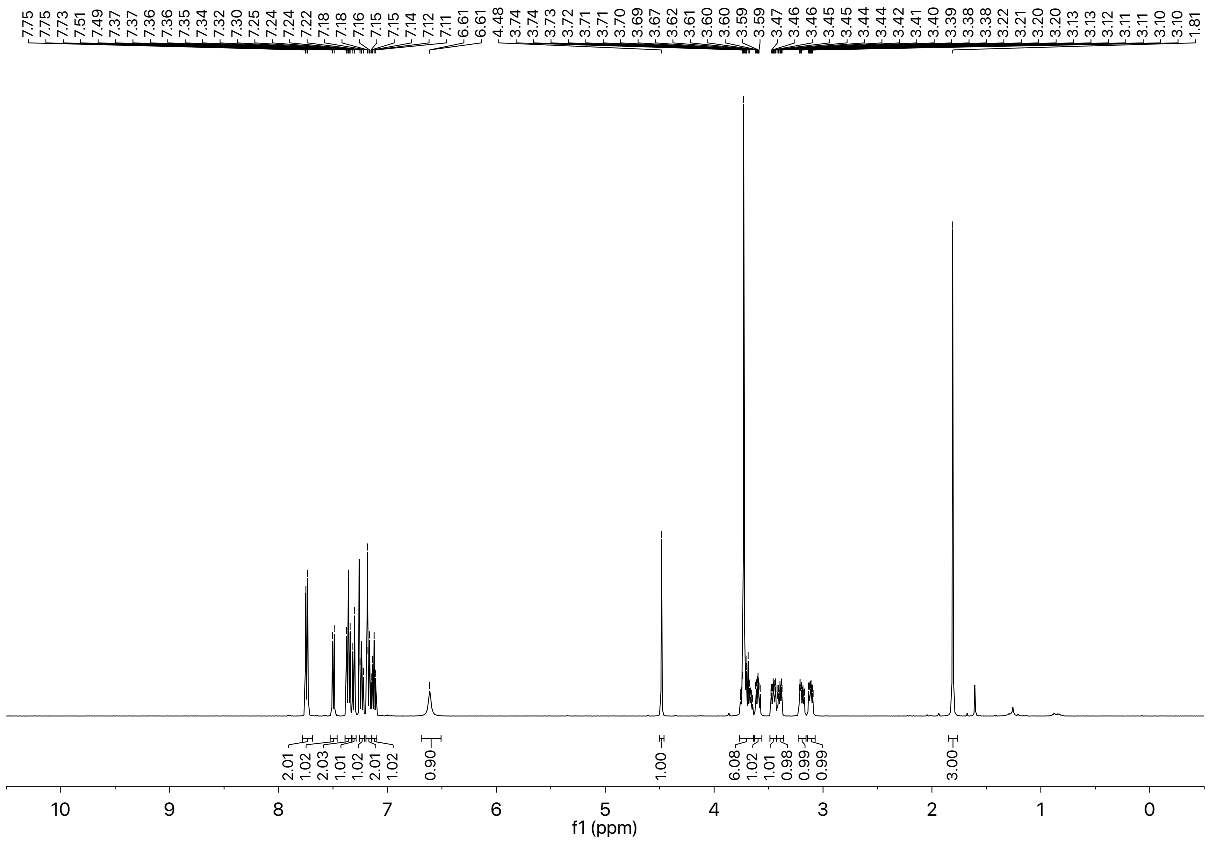
7.75
7.75
7.73
7.51
7.49
7.37
7.37
7.36
7.36
7.35
7.34
7.32
7.30
7.25
7.24
7.24
7.22
7.18
7.18
7.16
7.15
7.15
7.14
7.12
7.11
6.61
6.61
4.48
3.74
3.74
3.73
3.72
3.71
3.71
3.70
3.69
3.67
3.62
3.61
3.60
3.60
3.59
3.59
3.47
3.46
3.46
3.45
3.45
3.44
3.44
3.42
3.41
3.40
3.39
3.38
3.38
3.22
3.21
3.20
3.20
3.13
3.13
3.12
3.11
3.11
3.10
3.10
1.81
2.01
1.02
2.03
1.01
1.02
2.01
1.02
0.90
1.00
6.08
1.02
1.01
0.98
0.99
0.99
3.00
10
9
8
7
6
5
4
3
2
1
0
f1 (ppm)

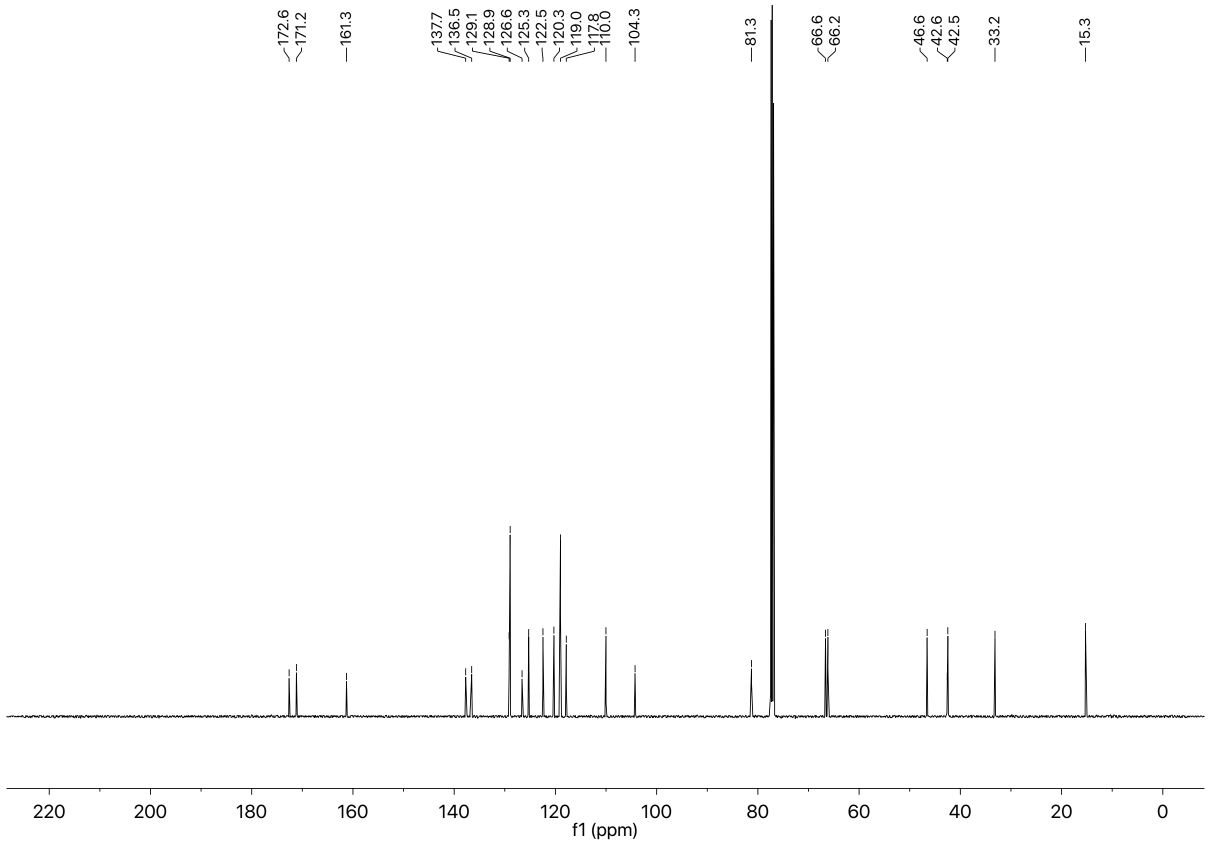
172.6
171.2
161.3
137.7
136.5
129.1
128.9
126.6
125.3
122.5
120.3
119.0
117.8
110.0
104.3
81.3
66.6
66.2
46.6
42.6
42.5
33.2
15.3
220
200
180
160
140
120
100
80
60
40
20
0
f1 (ppm)

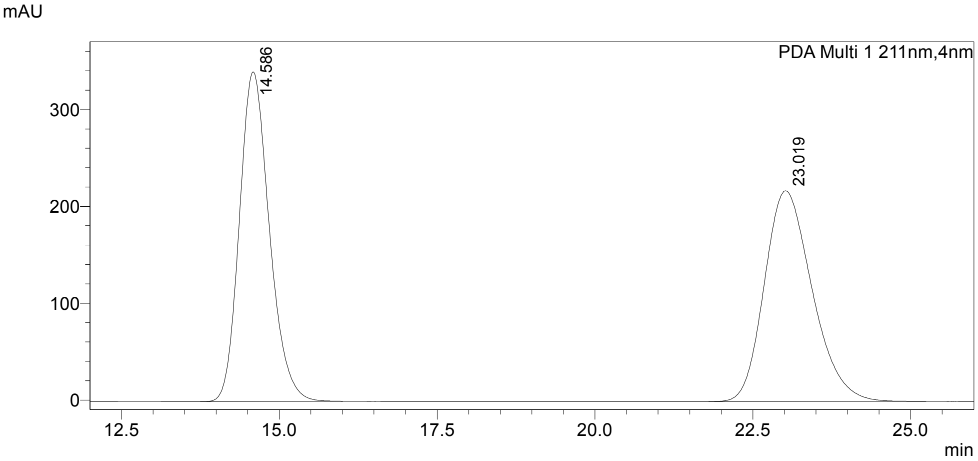

<Peak Table>

PDA Ch1 211nm

Peak#	Ret. Time	Area%
1	14.586	49.523
2	23.019	50.477
Total		100.000

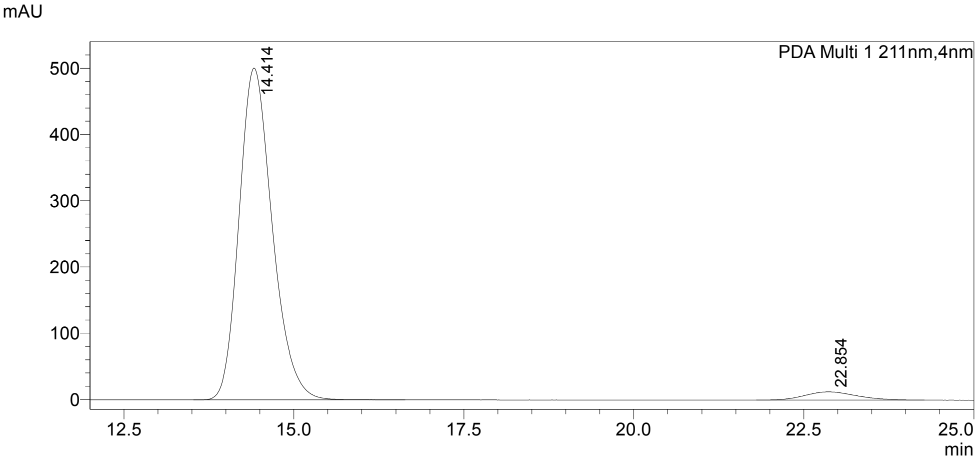

<Peak Table>

PDA Ch1 211nm

Peak#	Ret. Time	Area%
1	14.414	96.239
2	22.854	3.761
Total		100.000

5.13. (1′*R*,4*R*)- and (1′*S*,4*R*)-4-hydroxy-5-methyl-4-(2-morpholino-2-oxo-1-thiophen-2-ylethyl)-2-phenyl-2,4-dihydro-3*H*-pyrazol-3-one **28**

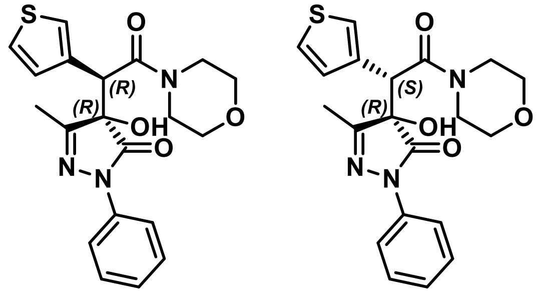

To a solution of 3-methyl-1-phenyl-1*H*-pyrazole-4,5-dione (47.1 mg, 0.25 mmol), 2-(thiophen-3-yl)acetic anhydride (99.9 mg, 0.375 mmol) and (2*R*,3*S*)-HyperBTM (3.9 mg, 0.125 mmol) were dissolved in ethyl acetate (6 ml, 0.04 M) at 0 °C. Diisopropylethylamine (54 µl, 0.31 mmol) was added and the reaction mixture was stirred at 0 °C for 3 h. Morpholine (66 µl, 0.75 mmol) was added and the reaction mixture was stirred for 16 h at rt. The reaction mixture was diluted with ethyl acetate and washed sequentially with 1 M aq. HCl (×2), sat. aq. $NaHCO_3$ (×2), and brine (×1). The organic layer was dried over $MgSO_4$, filtered, and concentrated *in vacuo*. Purification by column chromatography (*n*-hexane : ethyl acetate 4:1 to 1:1) gave the title compound as a mixture of diastereomers as white amorphous solid (49.9 mg, 0.125 mmol, 50%, 83:17 d.r.). $[\alpha]_D^{20}$ +193.1 (*c* 1.00, $CHCl_3$); **IR** ν_{max} (film) 3347 (O-H), 2967 (C-H), 2922 (C-H), 2857 (C-H), 1715 (C=O, pyrazolone), 1639, 1622, 1595, 1501, 1364, 1233, 1115, 756; **HRMS** (ESI^+) $C_{20}H_{20}N_3O_4SNa$ $[M+Na]^+$ found 422.11337, requires 422.11450 (–2.7 ppm). *Data for major diastereomer*: **HPLC Analysis:** Chiralpak AD-H (98:2 hexane:isopropanol, flow rate 2.0 ml·min^{-1}, 211 nm, 30 °C) t_R (1′*R*,4*R*)-**28**: 25.6 min, t_R (1′*S*,4*S*)-**28**: 43.6 min, >99:1 er; **^{1}H NMR** (500 MHz, $CDCl_3$) δ_H: 1.95 (3H, s, CH_3), 3.20-3.26 (2H, m, NC$H_A$$H_BCH_2$, NCH$_2CH_AH_B$), 3.36 (1H, ddd, $^2J_{HH}$ = 12.3 Hz, $^3J_{HH}$ = 6.6 Hz, 3.0 Hz, NCH$_A$$H_BCH_2$), 3.51-3.76 (5H, m, NC$H_C$$H_DCH_2$, NCH$_2CH_A$$H_B$, NCH$_2CH_C$$H_D$), 4.36 (1H, s, C*H*Ar), 6.36 (1H, br s, O*H*), 7.01 (1H, dd, $^3J_{HH}$ = 5.0 Hz, $^4J_{HH}$ = 1.4 Hz, CHArC^4*H*), 7.18 (1H, tt, $^3J_{HH}$ 7.4, $^4J_{HH}$ = 1.2, NArC^4*H*), 7.21 (1H, dd, $^4J_{HH}$ = 3.0 Hz, 1.4 Hz, CHArC^2*H*), 7.31 (1H, dd, $^3J_{HH}$ 5.0 Hz, $^4J_{HH}$ = 3.0 Hz, CHArC^5*H*), 7.35-7.40 (2H, m, NArC^{3,5}*H*), 7.74-7.77 (2H, m, NArC^{2,6}*H*); **^{13}C{^{1}H} NMR** (126 MHz, $CDCl_3$) δ_C: 15.4 (*C*H$_3$), 42.6 (N*C*H$_C$H$_D$CH$_2$), 46.6 (N*C*H$_A$H$_B$CH$_2$), 47.2 (*C*HAr), 66.2 (NCH$_2$*C*H$_A$H$_B$), 66.7 (NCH$_2$*C*H$_C$H$_D$), 81.3 (*C*-OH), 119.0 (NAr*C*^{2,6}H), 124.5 (CHAr*C*^{2}H), 125.5 (NAr*C*^{4}H), 127.3 (CHAr*C*^{5}H), 127.4 (CHAr*C*^{4}H), 129.0 (NAr*C*^{3,5}H), 132.0 (CHAr*C*^{3}), 137.6 (NAr*C*^{1}), 160.8 (*C*=N), 170.0 (*C*ON(CH$_2$CH$_2$)$_2$O), 171.8 (*C*ONAr); *Data for minor diastereomer*: **HPLC Analysis:** Chiralpak AD-H (98:2 hexane:isopropanol, flow rate 2.0 ml·min^{-1}, 211 nm, 30 °C) t_R (1′*S*,4*R*)-**28**: 29.4 min, t_R (1′*R*,4*S*)-**28**: 89.2 min, >99:1 er; **^{1}H NMR** (500 MHz, $CDCl_3$) (*selected*) δ_H: 1.97 (3H, s, CH_3), 4.35 (1H, s C*H*Ar), 4.93 (1H, br s, OH), 7.10 (1H, dd, $^3J_{HH}$ = 5.0, $^4J_{HH}$ = 1.4 Hz, CHArC^4*H*), 7.27-7.29 (1H, m, ArC*H*); **^{13}C{^{1}H} NMR** (126 MHz, $CDCl_3$)

(*selected*) δ_C: 14.6 (CH_3), 42.2 (NCH_2CH_2), 48.4 (*C*HAr), 66.3 (NCH_2CH_2), 79.8 (*C*-OH), 118.9 (NAr$C^{2,6}$H), 125.2 (Ar*C*H), 126.6 (Ar*C*H), 128.5 (Ar*C*H), 128.9 (NAr$C^{3,5}$H), 131.9 (CHArC^3H), 137.8 (NArC^1), 160.4 (*C*=N), 168.8 ($CON(CH_2CH_2)_2O$), 171.2 (*C*ONAr).

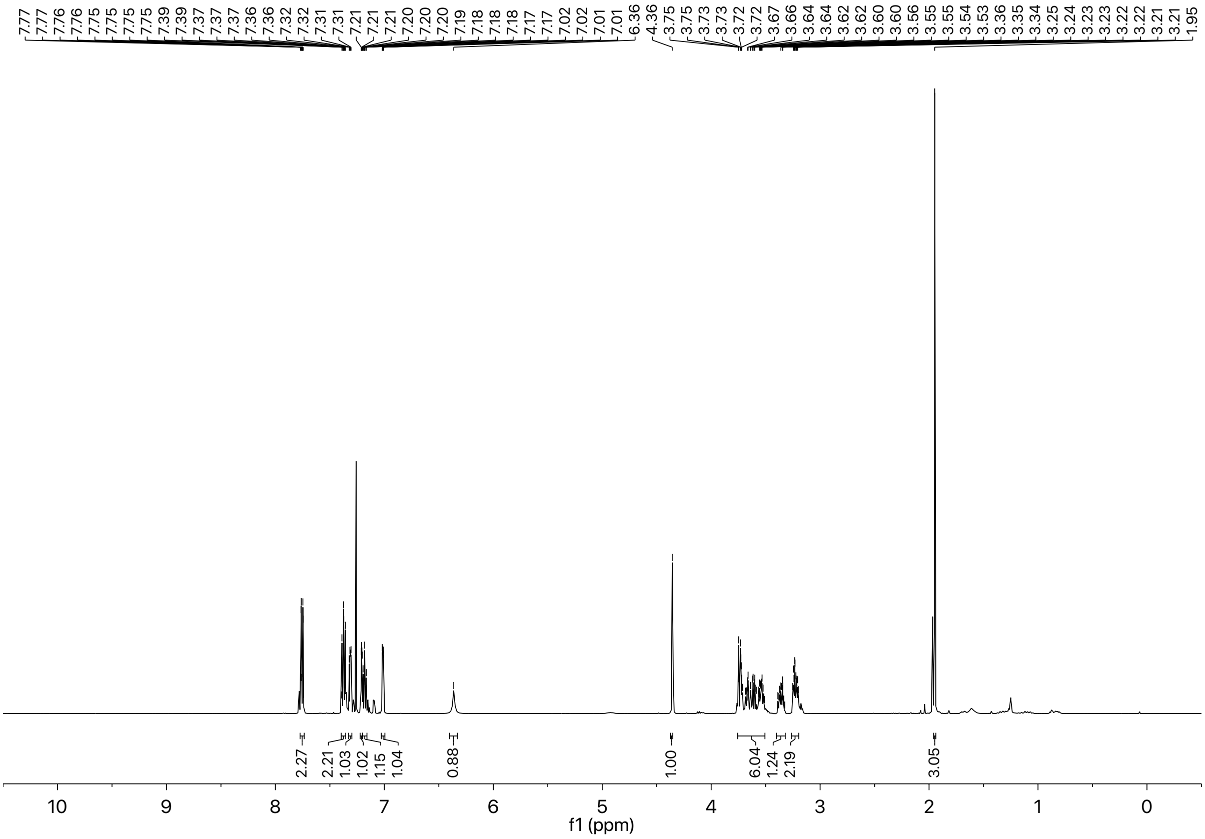

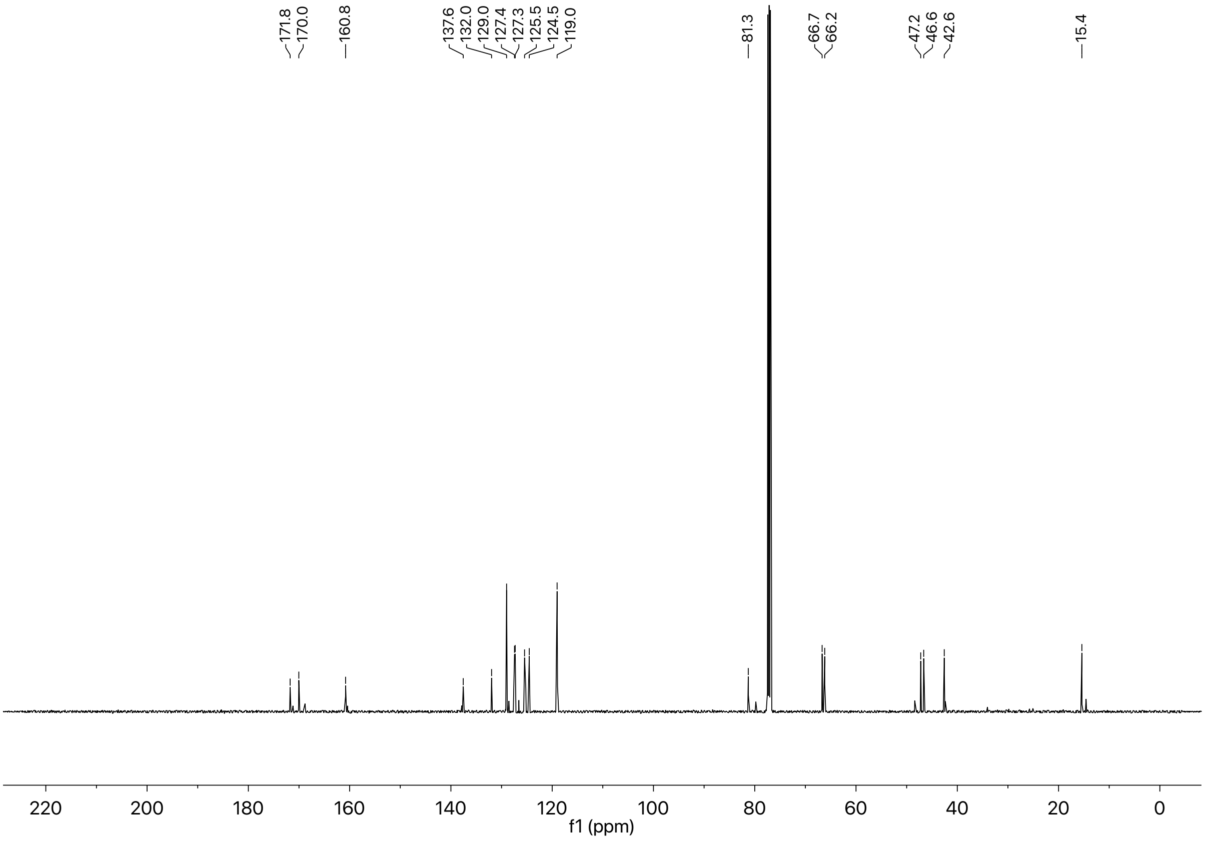

5.14. (2′*R*,4*R*)- and (2′*S*,4*R*)-(*E*)-4-hydroxy-5-methyl-4-(5-methyl-1-morpholino-1-oxohex-3-en-2-yl)-2-phenyl-2,4-dihydro-3*H*-pyrazol-3-one **29**

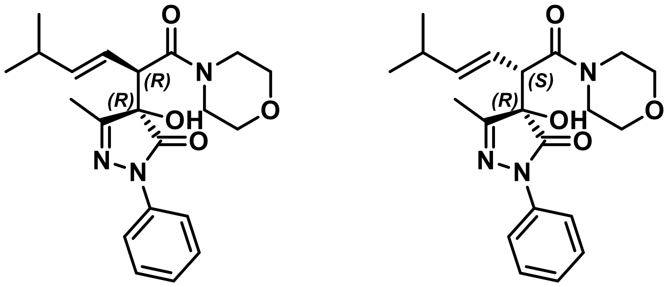

To a solution of 3-methyl-1-phenyl-1*H*-pyrazole-4,5-dione (47.1 mg, 0.25 mmol), (*E*)-5-methylhex-3-enoic anhydride (89.4 mg, 0.375 mmol) and (2*R*,3*S*)-HyperBTM (3.9 mg, 0.125 mmol) were dissolved in ethyl acetate (6 ml, 0.04 M) at 0 °C. Diisopropylethylamine (54 µl, 0.31 mmol) was added and the reaction mixture was stirred at 0 °C for 3 h. Morpholine (66 µl, 0.75 mmol) was added and the reaction mixture was stirred for 16 h at rt. The reaction mixture was diluted with ethyl acetate and washed sequentially with 1 M aq. HCl (×2), sat. aq. $NaHCO_3$ (×2), and brine (×1). The organic layer was dried over $MgSO_4$, filtered, and concentrated *in vacuo*. Purification by column chromatography (*n*-hexane : ethyl acetate 4:1 to 1:1) gave the title compound as white amorphous solid (23.9 mg, 0.06 mmol, 25%, 89:11 d.r.). $[\alpha]_D^{20}$ +183.8 (*c* 0.25, $CHCl_3$); **IR** ν_{max} (film) 3296 (O-H), 2961 (C-H), 2926 (C-H), 2866 (C-H), 1719 (C=O, pyrazolone), 1616, 1501, 1366, 1227, 1117, 756; **HRMS** (ESI$^+$) $C_{21}H_{27}N_3O_4Na$ $[M+Na]^+$ found 408.1889, requires 408.1894 (–1.2 ppm). *Data for major diastereomer*: **HPLC Analysis:** Chiralpak AD-H (85:15 hexane:isopropanol, flow rate 2.0 ml·min^{-1}, 211 nm, 30 °C) t_R (2′*R*,4*R*)-**29**: 4.4 min, t_R (2′*S*,4*S*)-**29**: 8.7 min, 98:2 er; **^{1}H NMR** (500 MHz, $CDCl_3$) δ_H: 0.87 (3H, d, $^3J_{HH}$ = 6.7 Hz, $CH(CH_3)_A(CH_3)_B$), 0.91 (3H, d, $^3J_{HH}$ = 6.7 Hz, $CH(CH_3)_A(CH_3)_B$), 2.20-2.30 (4H, m, C^5CH_3, $CH(CH_3)_2$), 3.38-3.51 (2H, m, $NCH_AH_BCH_2$), 3.51-3.79 (7H, m, $CHCONR_2$, $NCH_CH_DCH_2$, $N(CH_2CH_2)_2$), 5.40 (1H, ddd, $^3J_{HH}$ = 15.5 Hz, 9.2 Hz, $^4J_{HH}$ = 1.3 Hz, $HC{=}CHCH(CH_3)_2$), 5.63 (1H, dd, $^3J_{HH}$ = 15.5 Hz, 6.7 Hz, $HC{=}CHCH(CH_3)_2$), 5.69 (1H, br s, O*H*), 7.17 (1H, t, $^3J_{HH}$ = 7.4 Hz, $NArC^4H$), 7.33-7.43 (2H, m, $NArC^{3,5}H$), 7.86 (2H, d, $^3J_{HH}$ = 7.8 Hz, $NArC^{2,6}H$); **^{13}C{^{1}H} NMR** (126 MHz, $CDCl_3$) δ_C: 16.0 (C^5CH_3), 21.9 ($CH(CH_3)_A(CH_3)_B$), 22.2 ($CH(CH_3)_A(CH_3)_B$), 31.4 ($CH(CH_3)_2$), 42.5 ($NCH_CH_DCH_2$), 46.6 ($NCH_AH_BCH_2$), 49.8 ($CHCONR_2$), 66.5 ($NCH_2CH_AH_B$), 66.9 ($NCH_2CH_CH_D$), 81.0 (*C*-OH), 118.3 ($HC{=}CHCH(CH_3)_2$), 118.8 ($NArC^{2,6}H$), 125.3 ($NArC^4H$), 129.0 ($NArC^{3,5}H$), 137.8 ($NArC^1$), 146.0 ($HC{=}CHCH(CH_3)_2$), 160.7 (*C*=N), 170.1 ($CON(CH_2CH_2)_2O$), 171.8 (*C*ONAr); *Data for minor diastereomer*: **HPLC Analysis:** Chiralpak AD-H (85:15 hexane:isopropanol, flow rate 2.0 ml·min^{-1}, 211 nm, 30 °C) t_R (2′*S*,4*R*)-**29**: 4.0 min, t_R (2′*R*,4*S*)-**29**: 5.8 min, 94.5:5.5 er **^{1}H NMR** (500 MHz, $CDCl_3$) (*selected*) δ_H: 2.18 (3H, s, C^5CH_3), 2.35-3.43 (1H, m, $CH(CH_3)_2$).

Note: *As the dr could not be determined from the crude reaction mixture, the two diastereomers were initially isolated together by column chromatography to yield a crude mixture with 75:25 dr. This was further purified by column chromatography to give the pure product in 89:11 dr.*

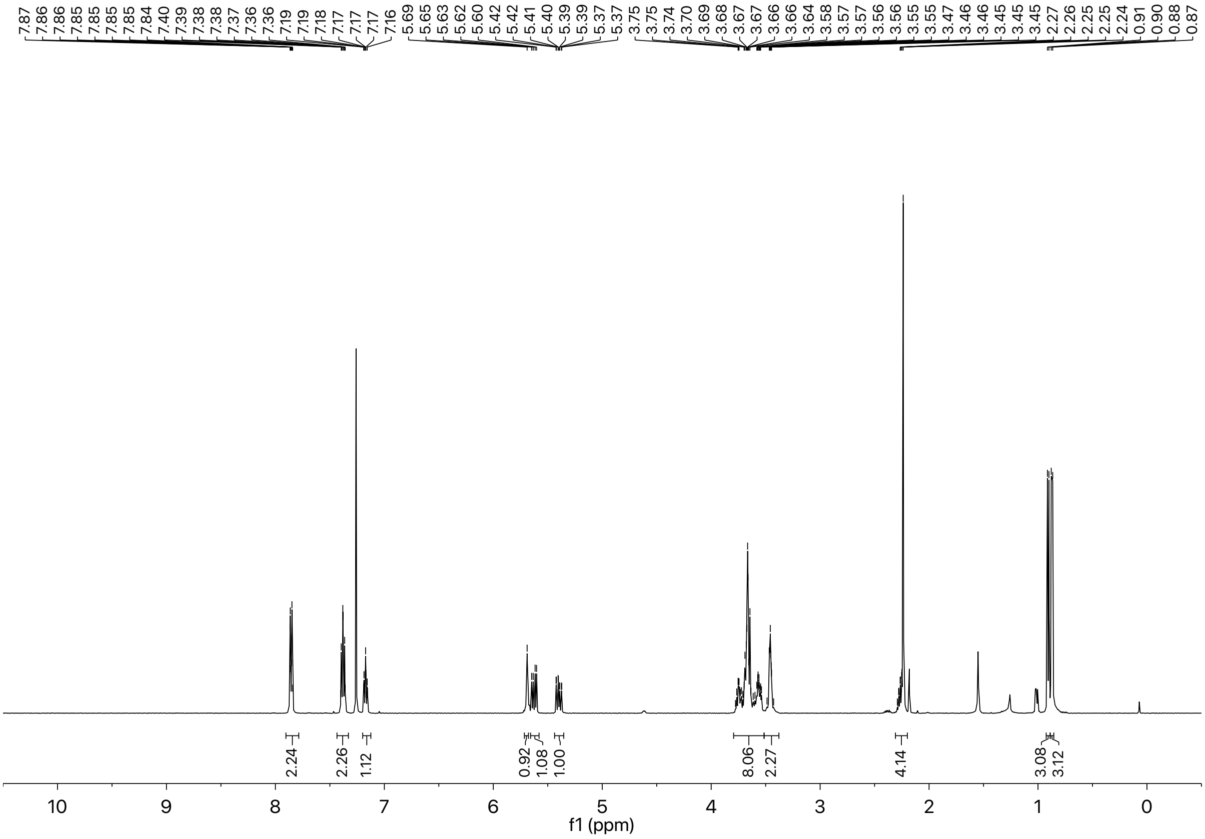

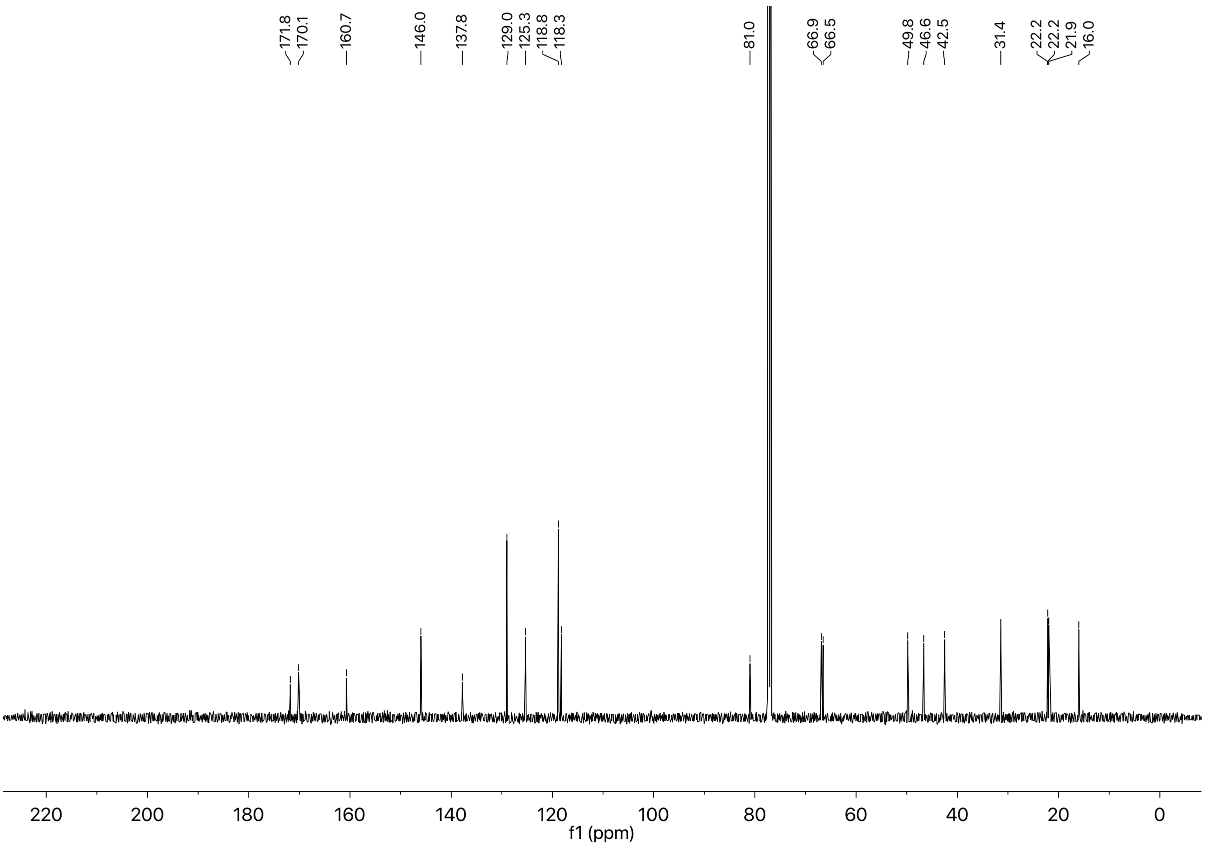

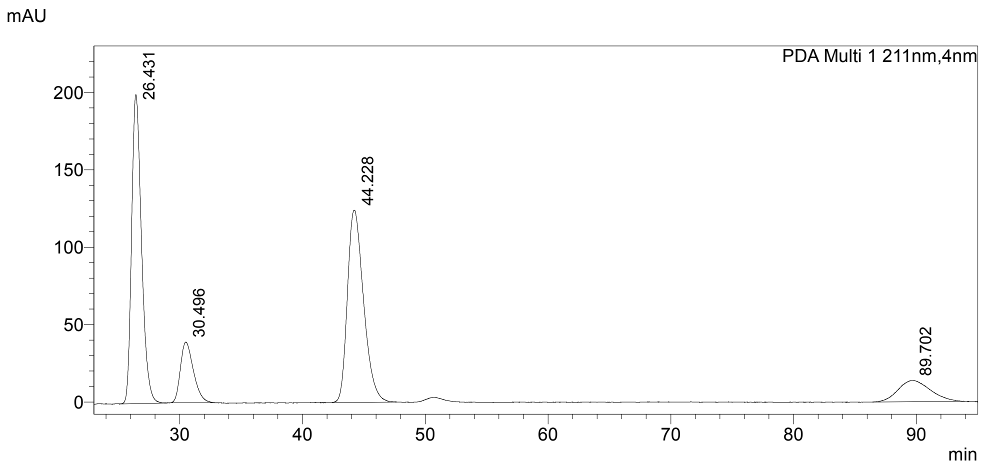

<Peak Table>

PDA Ch1 211nm

Peak#	Ret. Time	Area%
1	26.431	40.023
2	30.496	10.429
3	44.228	40.521
4	89.702	9.027
Total		100.000

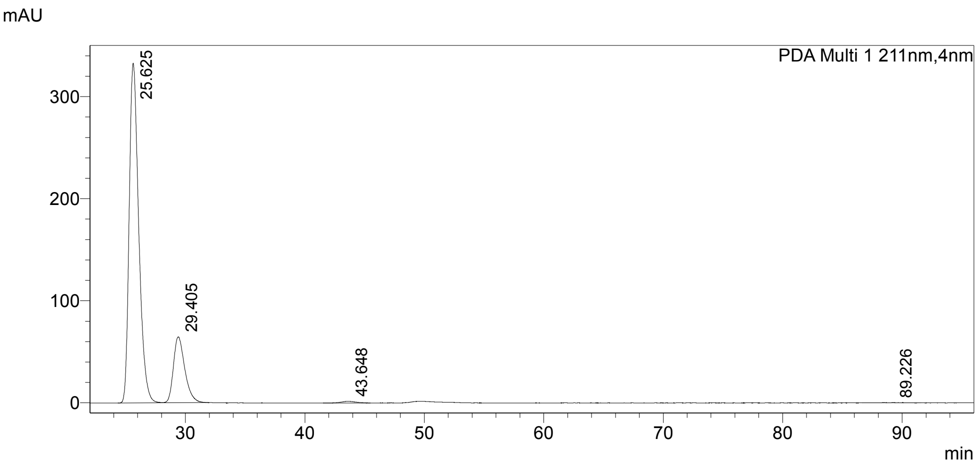

<Peak Table>

PDA Ch1 211nm

Peak#	Ret. Time	Area%
1	25.625	80.628
2	29.405	18.687
3	43.648	0.595
4	89.226	0.090
Total		100.000

5.15. (1′*R*,4*R*)- and (1′*S*,4*R*)-4-Hydroxy-5-isopropyl-4-(2-morpholino-2-oxo-1-phenylethyl)-2-phenyl-2,4-dihydro-3*H*-pyrazol-3-one **30**

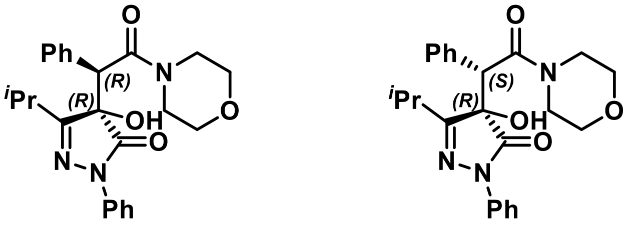

To a solution of 3-isopropyl-1-phenyl-1*H*-pyrazole-4,5-dione (54.1 mg, 0.25 mmol), 2-phenylacetic anhydride (95.4 mg, 0.375 mmol) and (2*R*,3*S*)-HyperBTM (3.9 mg, 0.125 mmol) were dissolved in ethyl acetate (6 ml, 0.04 M) at 0 °C. Diisopropylethylamine (54 µl, 0.31 mmol) was added and the reaction mixture was stirred at 0 °C for 3 h. Morpholine (66 µl, 0.75 mmol) was added and the reaction mixture was stirred for 16 h at rt. The reaction mixture was diluted with ethyl acetate and washed sequentially with 1 M aq. HCl (×2), sat. aq. $NaHCO_3$ (×2), and brine (×1). The organic layer was dried over $MgSO_4$, filtered, and concentrated *in vacuo*. Purification by column chromatography (*n*-hexane : ethyl acetate 4:1 to 1:1) gave the major and minor diastereomer (94.9 mg, 90%, 94:6 dr) as an inseparable mixture as a white amorphous solid. $[\alpha]_D^{20}$ +226.6 (*c* 1.00, $CHCl_3$); **IR** ν_{max} (film) 3360 (O-H), 3063, 2970 (C-H), 2928 (C-H), 2859 (C-H), 1717 (C=O, pyrazolone), 1643, 1622, 1597, 1493, 1348, 1225, 1113, 972, 864, 756; **HRMS** (ESI^+) $C_{24}H_{27}N_3O_4Na$ $[M+Na]^+$ found 444.1883, requires 444.1894 (–2.4 ppm). *Data for major diastereomer*: **HPLC Analysis:** Chiralpak AD-H (95:5 hexane:isopropanol, flow rate 2.00 ml·min^{-1}, 211 nm, 30 °C) t_R (1′*R*,4*R*)-**30**: 16.3 min, t_R (1′*S*,4*S*)-**30**: 31.3 min, >99:1 er; **^{1}H NMR** (500 MHz, $CDCl_3$) δ_H: 1.04 (3H, d, $^3J_{HH}$ = 6.8 Hz, $CH(CH_3)_A(CH_3)_B$), 1.26 (3H, d, $^3J_{HH}$ = 6.8 Hz, $CH(CH_3)_A(CH_3)_B$), 2.67 (1H, hept, $^3J_{HH}$ = 6.8 Hz, $CH(CH_3)_2$), 3.07-3.22 (2H, m, $NCH_AH_BCH_2$ + $NCH_2CH_AH_B$), 3.34 (1H, ddd, $^2J_{HH}$ = 14.5 Hz, $^3J_{HH}$ = 7.6 Hz, 3.0 Hz, $NCH_AH_BCH_2$), 3.50 (1H, ddd, $^2J_{HH}$ = 11.5 Hz, $^3J_{HH}$ = 6.6 Hz, 3.0 Hz, $NCH_2CH_AH_B$), 3.59 (1H, ddd, $^2J_{HH}$ = 11.2 Hz, $^3J_{HH}$ = 6.9 Hz, 2.5 Hz, $NCH_2CH_CH_D$), 3.66 (1H, ddd, $^2J_{HH}$ = 12.1 Hz, $^3J_{HH}$ = 6.9 Hz, 2.3 Hz, $NCH_CH_DCH_2$), 3.70-3.81 (2H, m, $NCH_CH_DCH_2$ + $NCH_2CH_CH_D$), 4.22 (1H, s, CHAr), 6.21 (1H, br s, OH), 7.19 (1H, tt, $^3J_{HH}$ = 7.5 Hz, $^4J_{HH}$ = 1.2 Hz, $NArC^4H$), 7.26-7.36 (5H, m, $CHArC^{2,3,4,5,6}H$), 7.38 (2H, app t, $^3J_{HH}$ = 7.9 Hz, $NArC^{3,5}H$), 7.78 (2H, d, $^3J_{HH}$ = 7.9 Hz, $NArC^{2,6}H$); **^{13}C{^{1}H} NMR** (126 MHz, $CDCl_3$) δ_C: 19.1 ($CH(CH_3)_A(CH_3)_B$), 22.9 ($CH(CH_3)_A(CH_3)_B$), 29.5 ($CH(CH_3)_2$), 42.5 ($NCH_CH_DCH_2$), 46.5 ($NCH_AH_BCH_2$), 53.0 (CHAr), 66.0 ($NCH_2CH_AH_B$), 66.6 ($NCH_2CH_CH_D$), 82.6 (*C*(4)-OH), 119.1 ($NArC^{2,6}H$), 125.2 ($NArC^4H$), 128.8 ($CHArC^{2,6}H$), 128.9 ($NArC^{3,5}H$), 129.0 ($CHArC^4H$), 129.2 ($CHArC^{3,5}H$), 132.1 ($CHArC^1$), 137.8 ($NArC^1$), 167.5 (*C*=N), 170.3 (*C*OO), 171.8 (*C*ONAr); *Data for minor diastereomer*: **HPLC Analysis:** Chiralpak AD-H (95:5 hexane:isopropanol, flow rate 2.00 ml·min^{-1}, 211 nm, 30 °C) t_R (1′*S*,4*R*)-**30**: 21.6 min, t_R (1′*R*,4*S*)-**30**: 43.7 min (not detected); **^{1}H**

NMR (500 MHz, $CDCl_3$) (*selected*) δ_H: 1.12 (3H, d, $^3J_{HH}$ = 6.9 Hz, $CH(CH_3)_A(CH_3)_B$), 1.35 (3H, d, $^3J_{HH}$ = 6.9 Hz, $CH(CH_3)_A(CH_3)_B$), 3.04 (1H, ddd, $^2J_{HH}$ = 10.8 Hz, $^3J_{HH}$ = 7.2 Hz, 2.6 Hz, $NCH_2CH_AH_B$), 4.18 (1H, s, C*H*Ar), 5.42 (1H, br s, O*H*), 7.14 (1H, t, $^3J_{HH}$ = 7.5 Hz, $^4J_{HH}$ = 1.1 Hz, NArC(4)*H*), 7.74 (2H, d, $^3J_{HH}$ = 7.8 Hz, $NArC^{2,6}H$); ^{13}**C{**^{1}**H} NMR** (126 MHz, $CDCl_3$) (*selected*) δ_C: 19.8 $CH(CH_3)_A(CH_3)_B$), 21.8 ($CH(CH_3)_A(CH_3)_B$), 29.0 ($CH(CH_3)_2$), 42.3 (NCH_2CH_2), 53.1 (*C*HAr), 81.5 (*C*-OH), 118.9 ($NArC^{2,6}H$), 125.0 ($NArC^4H$), 129.6 (Ar*C*H), 132.0 ($CHArC^1$), 137.9 ($NArC^1$), 166.7 (*C*=N), 169.3 (*C*OO), 171.3 (*C*ONAr).

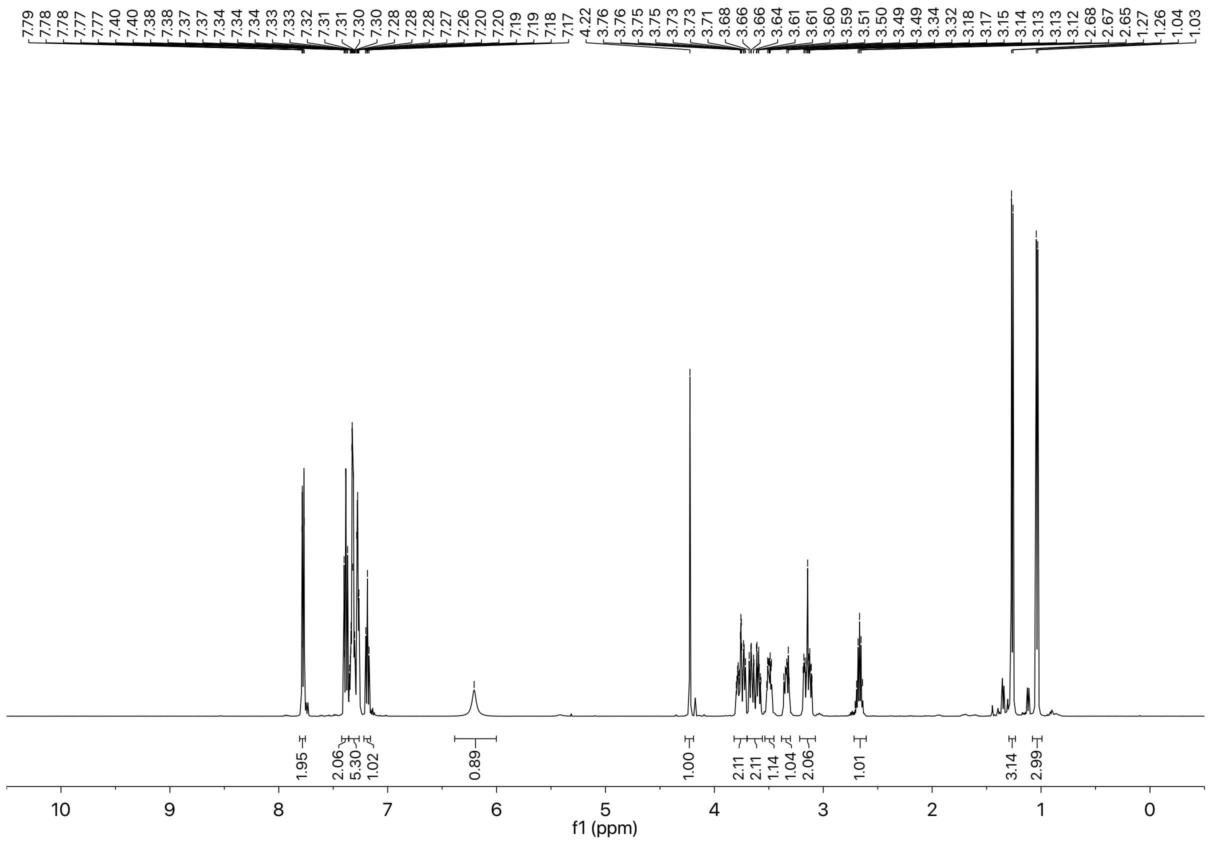

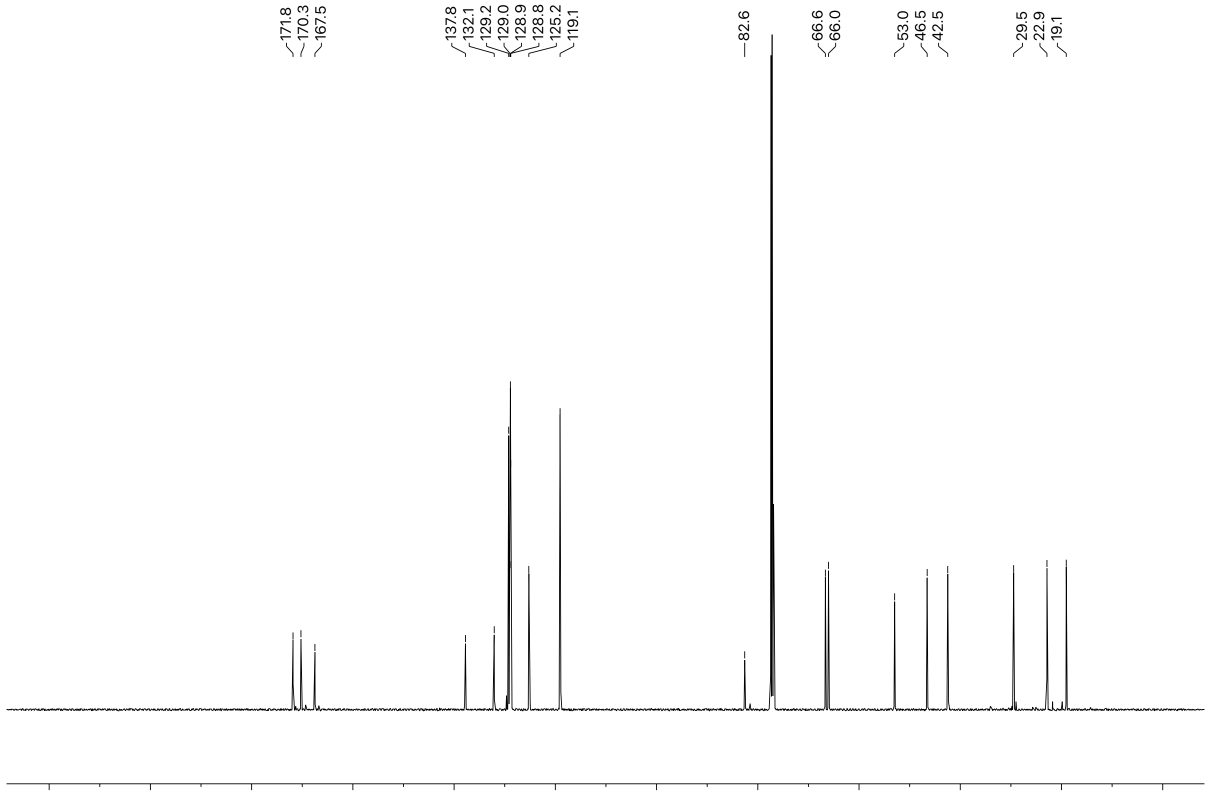

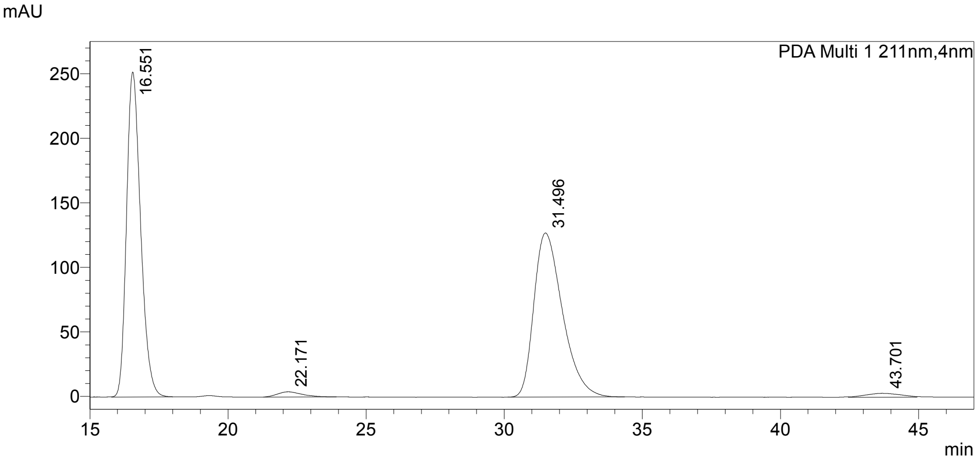

<Peak Table>

PDA Ch1 211nm

Peak#	Ret. Time	Area%
1	16.551	48.026
2	22.171	1.345
3	31.496	49.196
4	43.701	1.434
Total		100.000

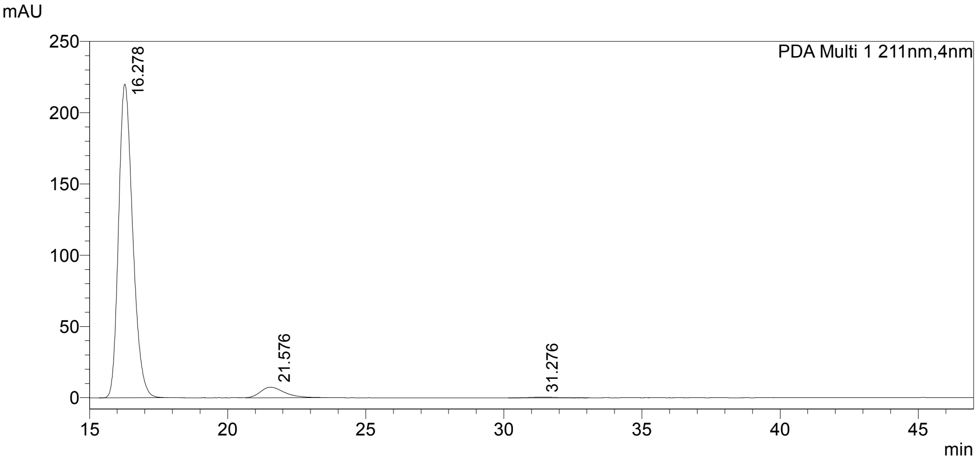

<Peak Table>

PDA Ch1 211nm

Peak#	Ret. Time	Area%
1	16.278	94.138
2	21.576	5.461
3	31.276	0.401
Total		100.000

5.16. (1′*R*,4*R*)- and (1′*S*,4*R*)-4-Hydroxy-5-isopropyl-4-(2-morpholino-1-(naphth-1-yl)-2-oxoethyl)-2-phenyl-2,4-dihydro-3*H*-pyrazol-3-one **31**

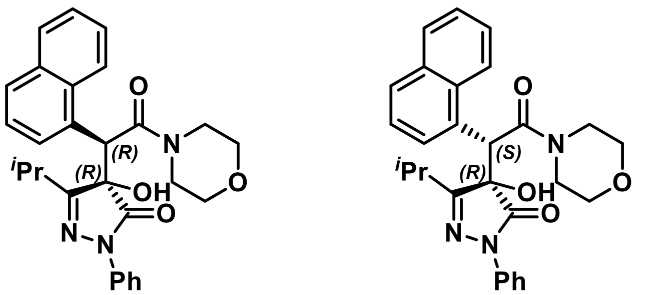

To a solution of 3-isopropyl-1-phenyl-1*H*-pyrazole-4,5-dione (54.1 mg, 0.25 mmol), 2-(naphtha-1-yl)acetic anhydride (132.9 mg, 0.375 mmol) and (2*R*,3*S*)-HyperBTM (3.9 mg, 0.125 mmol) were dissolved in ethyl acetate (6 ml, 0.04 M) at 0 °C. Diisopropylethylamine (54 µl, 0.31 mmol) was added and the reaction mixture was stirred at 0 °C for 3 h. Morpholine (66 µl, 0.75 mmol) was added and the reaction mixture was stirred for 16 h at rt. The reaction mixture was diluted with ethyl acetate and washed sequentially with 1 M aq. HCl (×2), sat. aq. $NaHCO_3$ (×2), and brine (×1). The organic layer was dried over $MgSO_4$, filtered, and concentrated *in vacuo*. Purification by column chromatography (*n*-hexane : ethyl acetate 4:1 to 1:1) gave the major and minor diastereomer (112.5 mg, 96%, >95:5 dr) as an inseparable mixture as a white amorphous solid. $[\alpha]_D^{20}$ +286.5 (*c* 1.00, $CHCl_3$); **IR** ν_{max} (film) 3319 (O-H), 3061, 2970, 2928, 2859, 1717 (C=O, pyrazolone), 1645, 1628, 1597, 1491, 1435, 1346, 1223, 1113, 791, 781; **HRMS** (ESI^+) $C_{28}H_{30}N_3O_4$ $[M+H]^+$ found 472.2219, requires 472.22309 (−2.5 ppm). *Data for major diastereomer anti*-**31**: **HPLC Analysis:** Chiralpak AD-H (85:15 hexane:isoproanol, flow rate 2.00 ml·min^{-1}, 211 nm, 30 °C) t_R (1′*R*,4*R*)-**31**: 6.5 min, t_R (1′*S*,4*S*)-**31**: 31.5 min, >99:1 er; **^{1}H NMR** (500 MHz, $CDCl_3$) δ_H: 0.65 (3H, d, J_{HH} 6.6, C(5)CH(CH_3)^A(CH_3)^B), 1.11 (3H, d, J_{HH} 6.8, C(5)CH(CH_3)^A(CH_3)^B), 1.97 (1H, app br s, C(5)C*H*(CH_3)$_2$), 2.76 (1H, ddd, J_{HH} 10.6, 7.2, 3.0, $NCH_2CH^AH^B$), 2.94 (1H, ddd, J_{HH} 13.5, 5.8, 3.0, $NCH^AH^BCH_2$), 3.18-3.32 (2H, m, $NCH^AH^BCH_2$ + $NCH_2CH^AH^B$), 3.48 (1H, ddd, J_{HH} 11.5, 7.4, 3.0, $NCH_2CH^CH^D$), 3.57 (1H, ddd, J_{HH} 13.3, 7.4, 3.0, $NCH^CH^DCH_2$), 3.67 (1H, ddd, J_{HH} 11.5, 5.8, 3.0, $NCH_2CH^CH^D$), 3.79 (1H, ddd, J_{HH} 13.3, 5.8, 3.0, $NCH^CH^DCH_2$), 4.95 (1H, s, C(1′)*H*), 6.88 (1H, br s, O*H*), 7.14 (1H, t, J_{HH} 7.4, NArC(4)*H*), 7.34 (2H, app t, J_{HH} 8.0, NArC(3,5)*H*), 7.45-7.58 (3H, m, C(1′)HArC(3)*H* + C(1′)HArC(7)*H* + C(1′)HArC(6)*H*), 7.74 (2H, d, J_{HH} 7.8, NArC(2,6)*H*), 7.81 (1H, d, J_{HH} 7.3, C(1′)HArC(2)*H*), 7.88 (2H, app d, J_{HH} 8.1, C(1′)HArC(4)*H* + C(1′)HArC(8)*H*), 7.95 (1H, d, J_{HH} 8.6, C(1′)HArC(5)*H*); **^{13}C{^{1}H} NMR** (126 MHz, $CDCl_3$) δ_C: 18.7 (C(5)CH(CH_3)^A(CH_3)^B), 22.9 (C(5)CH(CH_3)^A(CH_3)^B), 29.5 (C(5)*C*H(CH_3)$_2$), 42.7 ($NCH^CH^DCH_2$), 45.5 (*C*(1′)H), 46.2 ($NCH^AH^BCH_2$), 65.9 ($NCH_2CH^AH^B$), 66.5 ($NCH_2CH^CH^D$), 82.1 (*C*(4)-OH), 118.9 (NAr*C*(2,6)H), 121.2

(C(1′)HAr*C*(5)H), 125.1 (NAr*C*(4)H), 126.3 (C(1′)HAr*C*(7)H), 127.6 (C(1′)HAr*C*(3)H), 127.8 (C(1′)HAr*C*(6)H), 128.1 (C(1′)HAr*C*(2)H), 128.8 (NAr*C*(3,5)H), 129.7 (C(1′)HAr*C*(4)H), 129.8 (C(1′)HAr*C*(8)H), 130.8 (Ar*C*), 134.0 (Ar*C*), 138.0 (NAr*C*(1)), 167.4 (*C*(5)=N), 170.9 (*C*(2′)=O), 172.9 (*C*(3)=O); *Data for minor diastereomer syn-***31**: **HPLC Analysis:** Chiralpak AD-H (85:15 hexane:isopropanol, flow rate 2.00 ml·min^{-1}, 211 nm, 30 °C) t_R (1′*S*,4*R*)-**31**: 9.4 min, t_R (1′*R*,4*S*)-**103**: 14.5 min (not detected); **^{1}H NMR** (500 MHz, $CDCl_3$) (*selected*) δ_H: 1.20 (3H, d, J_{HH} 6.9, C(5)CH(C*H*$_3$)^A(CH_3)^B), 1.36 (3H, d, J_{HH} 6.7, C(5)CH(CH_3)^A(C*H*$_3$)^B), 2.86 (1H, ddd, J_{HH} 12.9, 5.2, 2.2, NCH_2C*H*$_2$), 4.81 (1H, s, C(1′)*H*), 6.27 (1H, br s, O*H*); **^{13}C{^{1}H} NMR** (126 MHz, $CDCl_3$) (*selected*) δ_C: 20.4 (C(5)CH(*C*H$_3$)^A(CH_3)^B), 22.2 (C(5)CH(CH_3)^A(*C*H$_3$)^B).

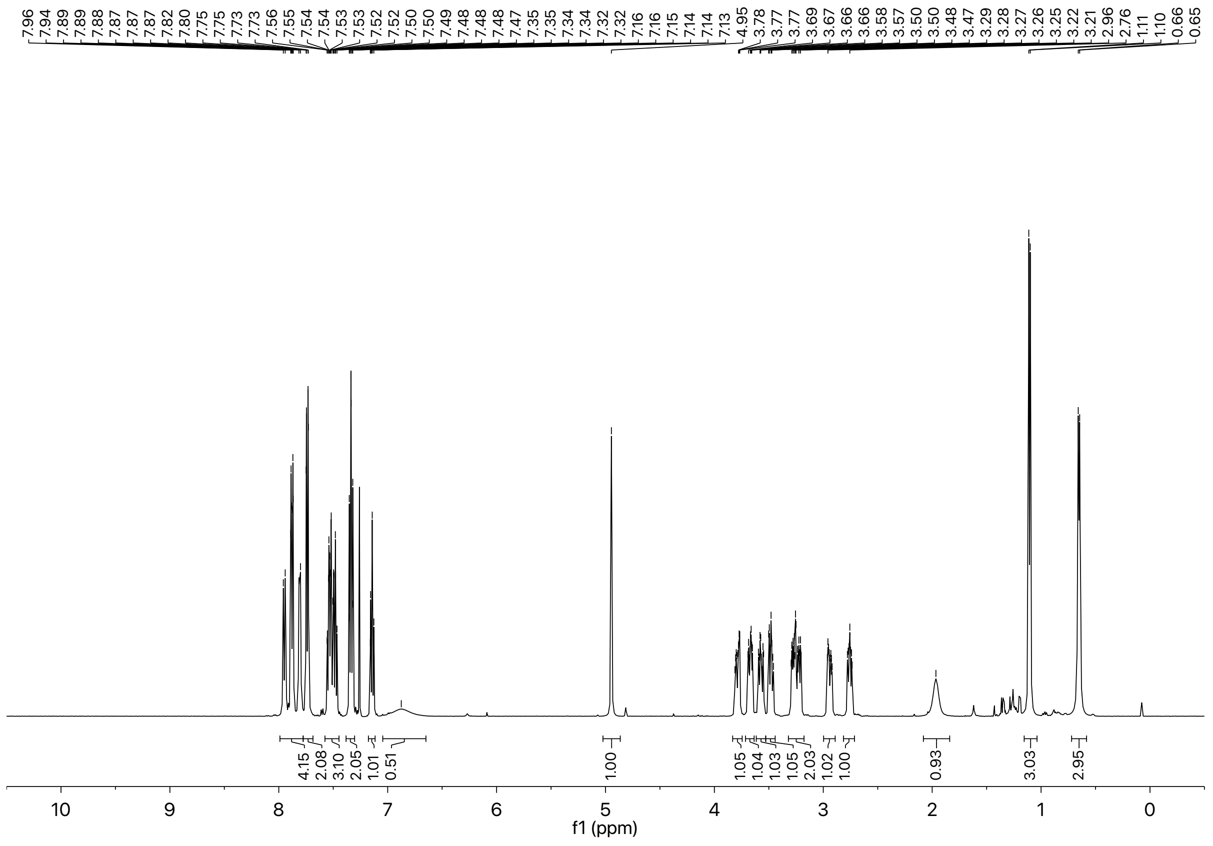

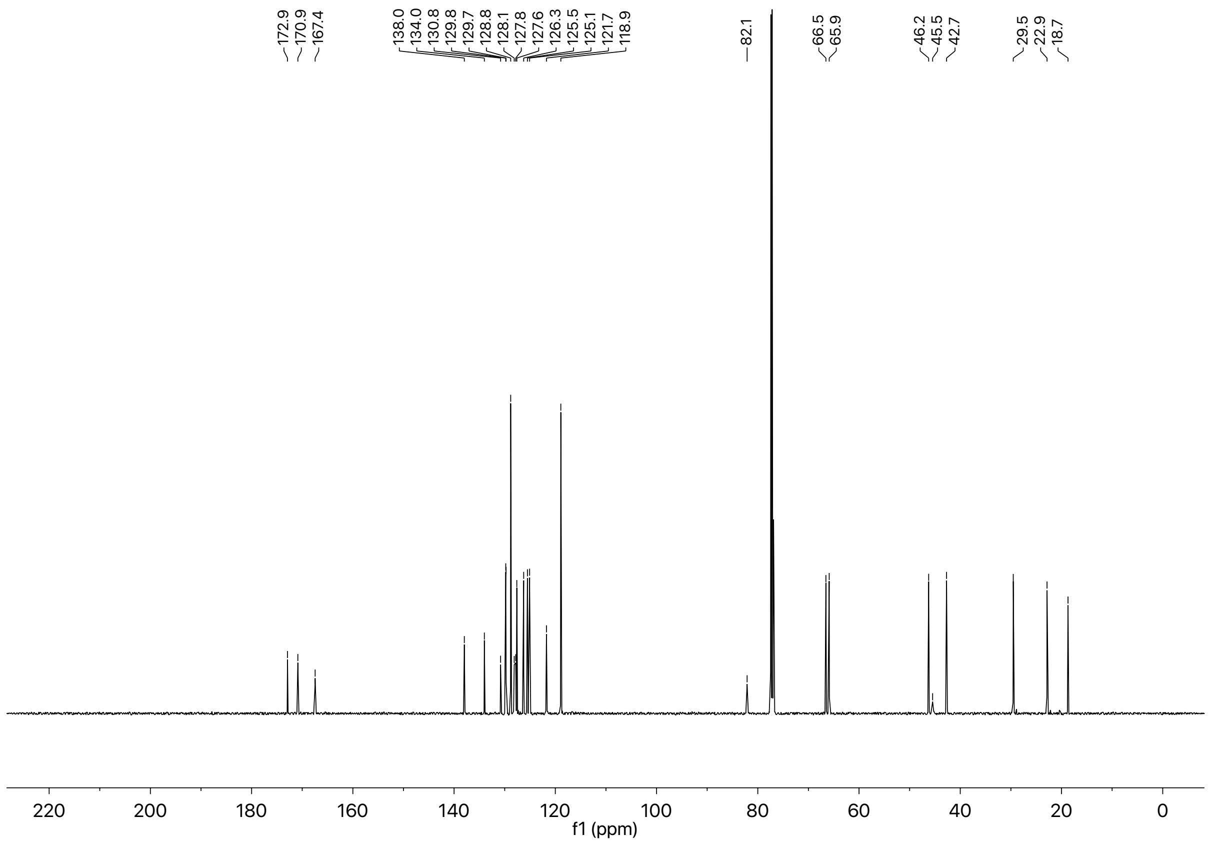

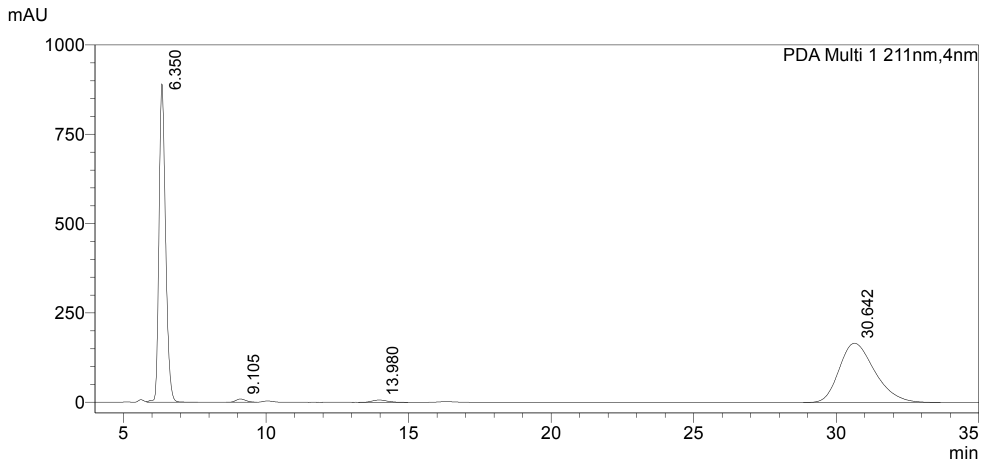

<Peak Table>

PDA Ch1 211nm

Peak#	Ret. Time	Area%
1	6.350	49.030
2	9.105	0.790
3	13.980	0.824
4	30.642	49.356
Total		100.000

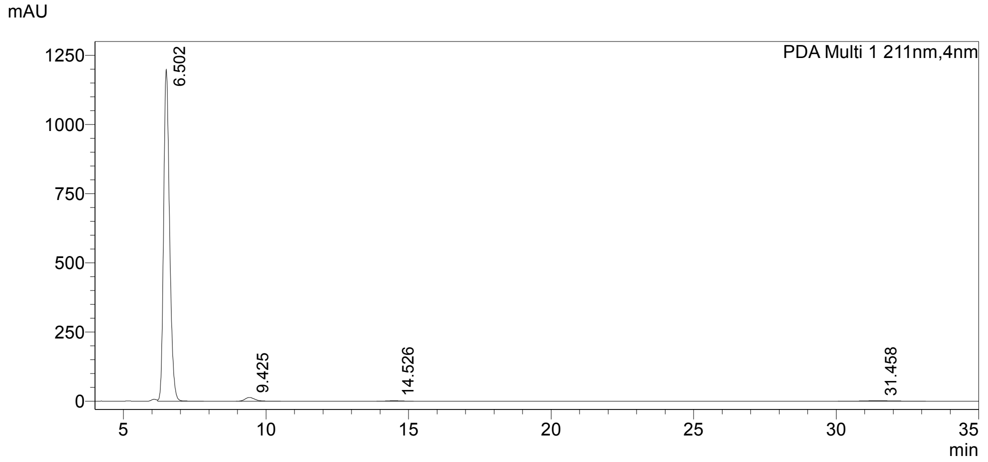

<Peak Table>

PDA Ch1 211nm

Peak#	Ret. Time	Area%
1	6.502	97.071
2	9.425	1.847
3	14.526	0.328
4	31.458	0.754
Total		100.000

5.17. (1′*R*,4*R*)- and (1′*S*,4*R*)-4-Hydroxy-4-(2-morpholino-2-oxo-1-phenylethyl)-2,5-diphenyl-2,4-dihydro-3*H*-pyrazol-3-one **32**

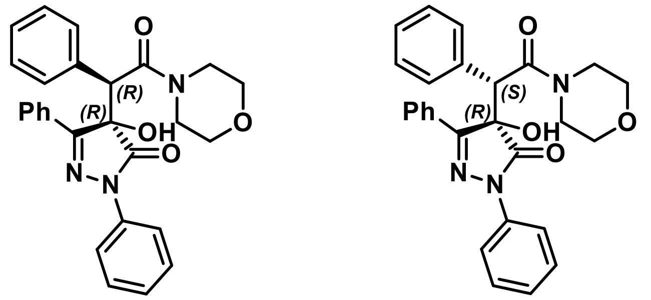

To a solution of 1,3-diphenyl-1*H*-pyrazole-4,5-dione (62.6 mg, 0.25 mmol), 2-phenylacetic anhydride (95.4 mg, 0.375 mmol) and (2*R*,3*S*)-HyperBTM (3.9 mg, 0.125 mmol) were dissolved in ethyl acetate (6 ml, 0.04 M) at 0 °C. Diisopropylethylamine (54 µl, 0.31 mmol) was added and the reaction mixture was stirred at 0 °C for 3 h. Morpholine (66 µl, 0.75 mmol) was added and the reaction mixture was stirred for 16 h at rt. The reaction mixture was diluted with ethyl acetate and washed sequentially with 1 M aq. HCl (×2), sat. aq. $NaHCO_3$ (×2), and brine (×1). The organic layer was dried over $MgSO_4$, filtered, and concentrated *in vacuo*. Purification by column chromatography (*n*-hexane : ethyl acetate 4:1 to 1:1) gave the major and minor diastereomer (83.0 mg, 73%, 88:12 dr) as an inseparable mixture as a pale yellow amorphous solid. $[\alpha]_D^{20}$ +205.1 (*c* 0.75, $CHCl_3$); **IR** ν_{max} (film) 3316 (O-H), 3061, 2967 (C-H), 2922 (C-H), 2857 (C-H), 1724 (C=O, pyrazolone), 1639, 1597, 1493, 1111, 752; **HRMS** (ESI^+) $C_{27}H_{24}N_3O_4Na$ $[M+Na]^+$ found 478.17267, requires 478.17373 (–2.2 ppm). *Data for major diastereomer anti-***32**: **HPLC Analysis:** Chiralpak AD-H (95:5 hexane:isopropanol, flow rate 2.00 ml·min^{-1}, 211 nm, 30 °C) t_R (1′*R*,4*R*)-**32**: 17.7 min, t_R (1′*S*,4*S*)-**32**: 19.2 min, 96.5:3.5 er; **^{1}H NMR** (500 MHz, $CDCl_3$) δ_H: 2.97-3.03 (1H, m, $NCH_2CH^AH^B$), 3.06 (1H, ddd, J_{HH} 13.9, 6.4, 2.8, $NCH^AH^BCH_2$), 3.10-3.20 (2H, m, $NCH^AH^BCH_2$ + $NCH_2CH^AH^B$), 3.44-3.51 (1H, m, $NCH^CH^DCH_2$), 3.51-3.61 (3H, m, $NCH^CH^DCH_2$ + $NCH_2CH^CH^D$ + $NCH_2CH^CH^D$), 4.17 (1H, s, C(1′)*H*), 5.82 (1H, br s, O*H*), 7.17-7.25 (6H, m, C(5)ArC(3,5)*H* + C(5)ArC(4)*H* + C(1′)HArC(2,6)*H +* NArC(4)*H*), 7.27-7.34 (3H, m, C(1′)HArC(3,5)*H* + C(1′)HArC(4)*H*), 7.43 (2H, app t, J_{HH} 7.8, NArC(3,5)*H*), 7.77 (2H, d, J_{HH} 7.4, C(5)ArC(2,6)*H*), 8.0 (2H, d, J_{HH} 8.0, NArC(2,6)*H*); **^{13}C{^{1}H} NMR** (126 MHz, $CDCl_3$) δ_C: 42.5 ($NCH^CH^DCH_2$), 46.4 ($NCH^AH^BCH_2$), 52.8 (*C*(1′)H), 66.1 ($NCH_2CH^AH^B$), 66.6 ($NCH_2CH^CH^D$), 80.5 (*C*(4)-OH), 119.1 (NAr*C*(2,6)H), 125.4 (C(1′)HAr*C*(2,6)H), 127.2 (C(5)Ar*C*(2,6)H), 128.3 (Ar*C*H), 128.6 (Ar*C*H), 128.8 (Ar*C*H), 129.0 (NAr*C*(3,5)H), 130.0 (C(1′)HAr*C*(4)H), 130.4 (C(1′)HAr*C*(2,5)H), 131.0 (Ar*C*), 131.0 (Ar*C*), 138.1 (NAr*C*(1)), 156.7 (*C*(5)=N), 168.8 (*C*(2′)=O) 172.7 (*C*(3)=O); *Data for minor diastereomer syn-***32**: **HPLC Analysis:** Chiralpak AD-H (95:5 hexane:isopropanol, flow rate 2.00 ml·min^{-1}, 211 nm, 30 °C) t_R (1′*S*,4*R*)-**32**: 41.3 min, t_R (1′*R*,4*S*)-**32**: 46.8 min, 97:3 er; **^{1}H NMR** (500 MHz, $CDCl_3$) (*selected*) δ_H: 2.87 (1H, ddd, J_{HH} 10.8, 7.6, 2.8, NCH_2CH_2), 2.91-2.91 (1H, m,

N*C*H_2CH_2), 3.28 (1H, ddd, J_{HH} 11.6, 5.6, 3.1, NCH_2C*H_2*), 3.75-3.85 (2H, m, NC*H_2*CH_2), 4.33 (1H, s, C(1′)*H*), 6.34 (1H, br s, O*H*), 7.02-7.06 (2H, m, ArH), 7.57 (2H, d, J_{HH} 8.1, C(5)Ar*C*(2,6)H); **^{13}C{^{1}H} NMR** (126 MHz, $CDCl_3$) (*selected*) δ_C: 42.34 (N*C*H_2CH_2), 53.7 (*C*(1′)H), 65.7 (NCH_2*C*H_2), 82.0 (*C*(4)-OH), 119.4 (NAr*C*(2,6)H), 127.4 (Ar*C*H), 129.4 (Ar*C*H), 131.8 (Ar*C*), 137.4 (NAr*C*(1)), 155.7 (*C*(5)=N), 169.3 (*C*(2′)=O), 171.8 (*C*(3)=O).

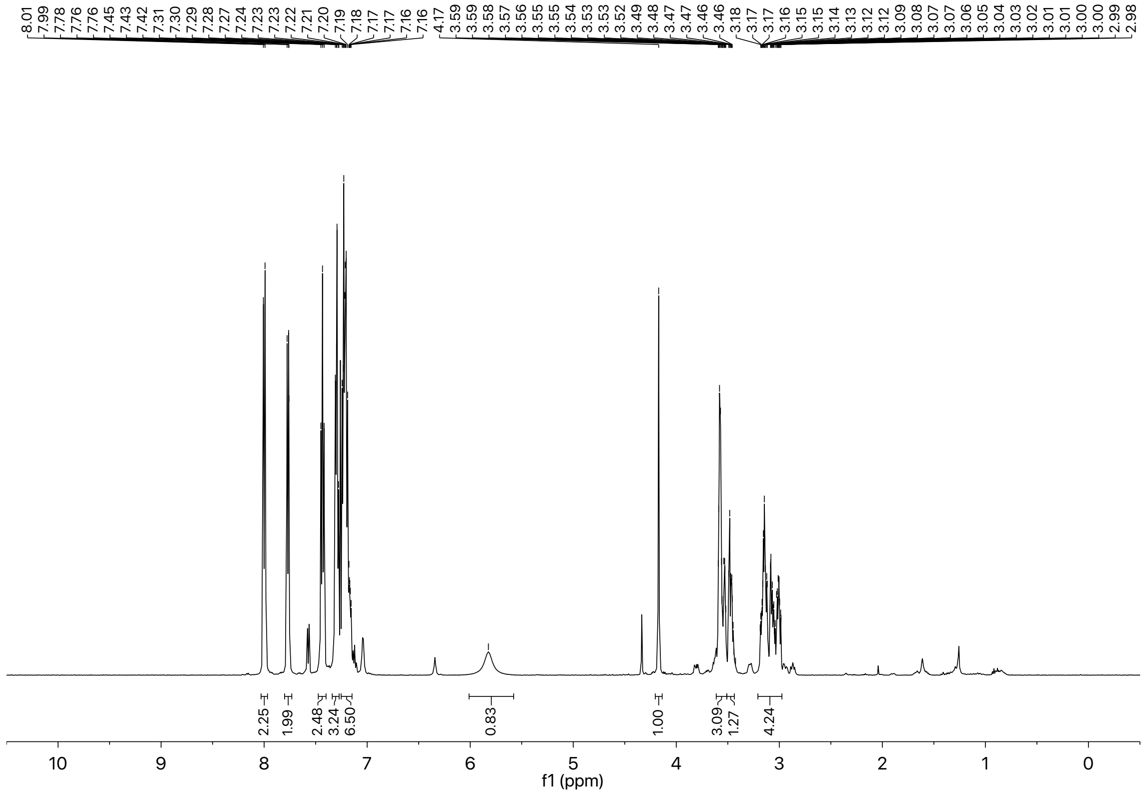

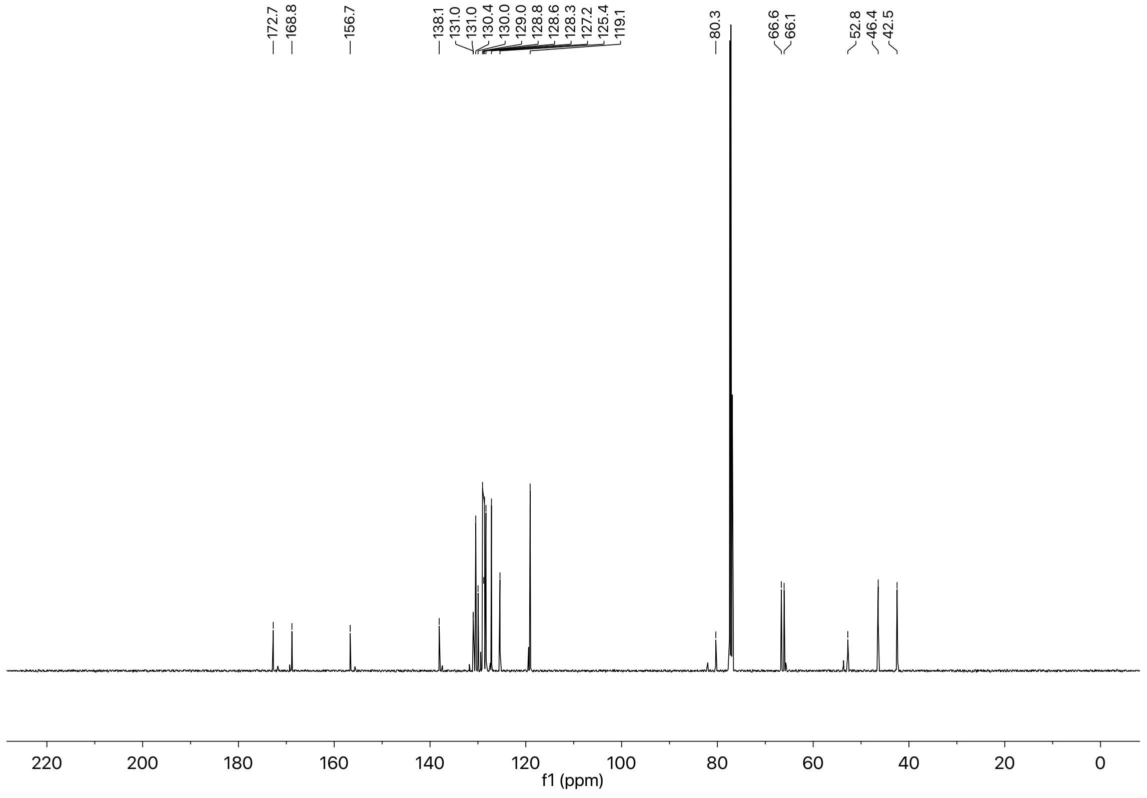

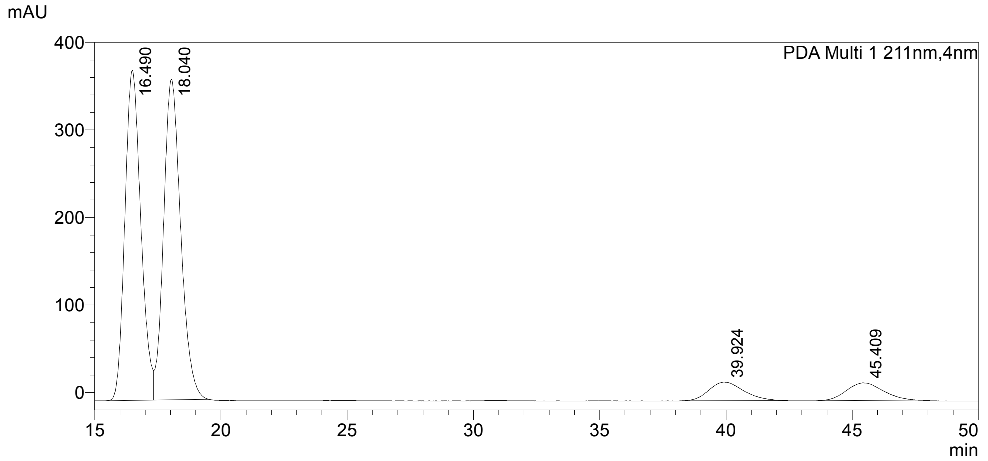

<Peak Table>

PDA Ch1 211nm

Peak#	Ret. Time	Area%
1	16.490	43.738
2	18.040	45.586
3	39.924	5.385
4	45.409	5.291
Total		100.000

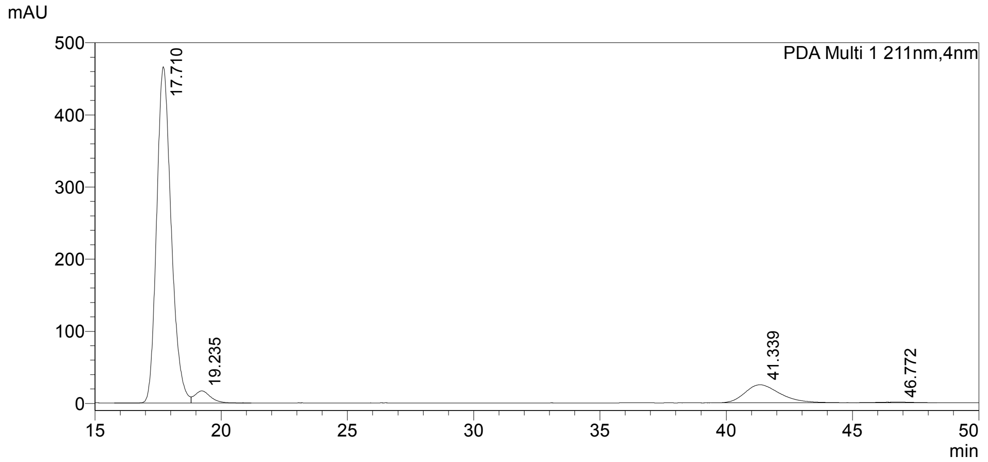

<Peak Table>

PDA Ch1 211nm

Peak#	Ret. Time	Area%
1	17.710	85.275
2	19.235	3.350
3	41.339	11.008
4	46.772	0.368
Total		100.000

5.18. (1′*R*,4*R*)- and (1′*S*,4*R*)-4-Hydroxy-4-(2-morpholino-1-(naphtha-1-yl)-2-oxoethyl)-2,5-diphenyl-2,4-dihydro-3*H*-pyrazol-3-one **33**

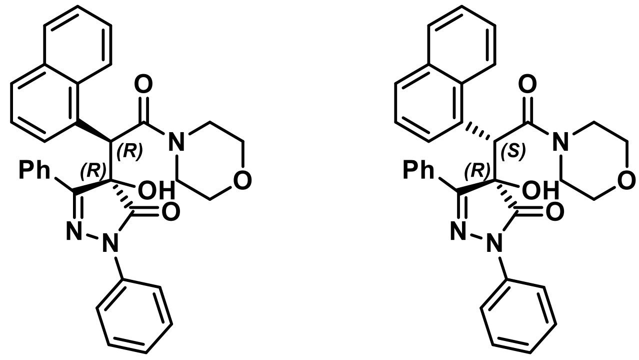

To a solution of 1,3-diphenyl-1*H*-pyrazole-4,5-dione (62.6 mg, 0.25 mmol), 2-(naphth-1-yl)acetic anhydride (132.9 mg, 0.375 mmol) and (2*R*,3*S*)-HyperBTM (3.9 mg, 0.125 mmol) were dissolved in ethyl acetate (6 ml, 0.04 M) at 0 °C. Diisopropylethylamine (54 µl, 0.31 mmol) was added and the reaction mixture was stirred at 0 °C for 3 h. Morpholine (66 µl, 0.75 mmol) was added and the reaction mixture was stirred for 16 h at rt. The reaction mixture was diluted with ethyl acetate and washed sequentially with 1 M aq. HCl (×2), sat. aq. $NaHCO_3$ (×2), and brine (×1). The organic layer was dried over $MgSO_4$, filtered, and concentrated *in vacuo*. Purification by column chromatography (*n*-hexane : ethyl acetate 4:1 to 1:1) gave the major and minor diastereomer (123.6 mg, 98%, >95:5 dr) as an inseparable mixture as a pale yellow amorphous solid. $[\alpha]_D^{20}$ +325.2 (*c* 1.00, $CHCl_3$); **IR** ν_{max} (film) 3271 (O-H), 3057, 2967 (C-H), 2920 (C-H), 2857 (C-H), 1721 (C=O, pyrazolone), 1639, 1595, 1491, 1111, 793, 779; **HRMS** (ESI^+) $C_{31}H_{26}N_3O_4Na$ $[M+Na]^+$ found 528.18787, requires 528.18938 (–2.9 ppm). *Data for major diastereomer anti-***33**: **HPLC Analysis:** Chiralpak IB (90:10 hexane:isopropanol, flow rate 1.00 ml·min^{-1}, 211 nm, 30 °C) t_R (2′*R*,4*R*): 18.4 min, t_R (2′*S*,4*S*): 10.9 min, 98:2 er; **^{1}H NMR** (500 MHz, $CDCl_3$) δ_H: 2.75 (1H, ddd, J_{HH} 10.9, 7.6, 3.0, $NCH_2CH^AH^B$), 2.88 (1H, ddd, J_{HH} 14.2, 6.5, 3.0, $NCH^AH^BCH_2$), 3.05-3.19 (2H, m, $NCH^AH^BCH_2$ + $NCH_2CH^AH^B$), 3.41-3.52 (1H, m, $NCH_2CH^CH^D$), 3.53-3.66 (2H, m, $NCH^CH^DCH_2$ + $NCH_2CH^CH^D$), 3.66-3.80 (1H, m, $NCH^CH^DCH_2$), 4.94 (1H, s, C(1′)*H*), 6.87 (2H, app t, J_{HH} 7.8, C(5)ArC(3,5)*H*), 6.99 (1H, t, J_{HH} 7.4, C(5)ArC(4)*H*), 7.23-7.27 (1H, m, NArC(4)*H*), 7.36 (1H, t, J_{HH} 7.4, C(1′)HArC(6)*H*), 7.41-7.51 (6H, m, C(5)ArC(2,6)*H* + C(1′)HArC(3)*H* + NArC(3,5)*H* + C(1′)HArC(7)*H*), 7.59-7.67 (2H, m, C(1′)HArC(4)*H* + C(1′)HArC(5)*H*), 7.81 (1H, d, J_{HH} 8.6, C(1′)HArC(8)*H*), 7.88 (1H, d, J_{HH} 7.2, C(1′)HArC(2)*H*), 8.05 (2H, d, J_{HH} 7.8, NArC(2,6)*H*); **^{13}C{^{1}H} NMR** (126 MHz, $CDCl_3$) δ_C: 42.7 ($NCH^CH^DCH_2$), 44.6 (*C*(1′)H), 46.2 ($NCH^AH^BCH_2$), 65.9 ($NCH_2CH^AH^B$), 66.6 ($NCH_2CH^CH^D$), 81.7 (*C*(4)-OH), 119.1 (NAr*C*(2,6)H), 121.5 (C(1′)HAr*C*(8)H), 125.3 (Ar*CH*), 125.4 (NAr*C*(4)H), 125.6 (C(1′)HAr*C*(6)H), 126.5 (C(5)Ar*C*(3,5)H), 127.2 (C(5)Ar*C*(2,6)H), 128.9 (C(1′)HAr*C*(2)H), 129.1 (NAr*C*(3,5)H), 129.4

(C(5)Ar*C*(4)H), 129.5 (C(1′)HAr*C*(5)H), 129.6 (C(1′)HAr*C*(4)H), 130.6 (C(5)Ar*C*(1)), 131.4 (Ar*C*), 133.9 (C(1′)HAr*C*(4a)), 138.2 (NAr*C*(1)), 157.1 (*C*(5)=N), 170.2 (*C*(2′)=O), 173.3 (*C*(3)=O); *Data for minor diastereomer syn-***33**: **HPLC Analysis:** Chiralpak IB (90:10 hexane:isopropanol, flow rate 1.00 ml·min^{-1}, 211 nm, 30 °C) t_R (2′*S*,4*R*): 9.6 min, t_R (2′*R*,4*S*): 14.3 min, 61:39 er; **^{1}H NMR** (500 MHz, $CDCl_3$) (*selected*) δ_H: 5.20 (1H, s, C(1′)*H*), 7.70 (1H, d, J_{HH} 8.1, Ar*H*); **^{13}C{^{1}H} NMR** (126 MHz, $CDCl_3$) (*selected*) δ_C: 119.3 (NAr*C*(2,6)H), 128.6 (Ar*C*H), 128.7 (Ar*C*H).

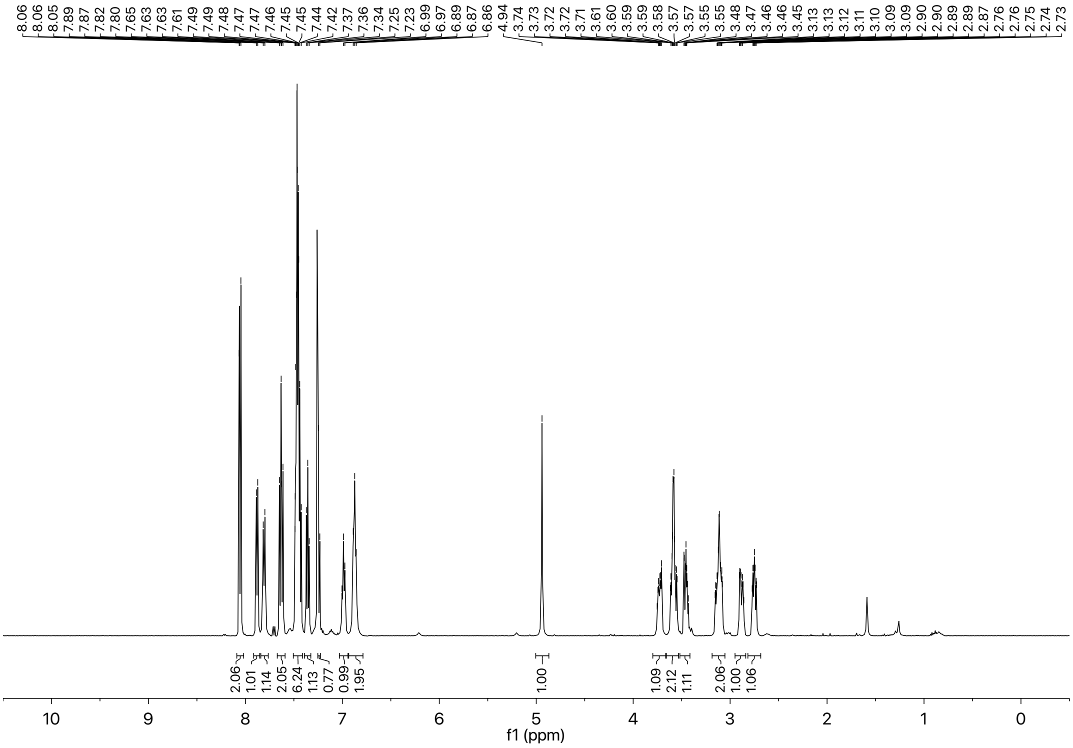

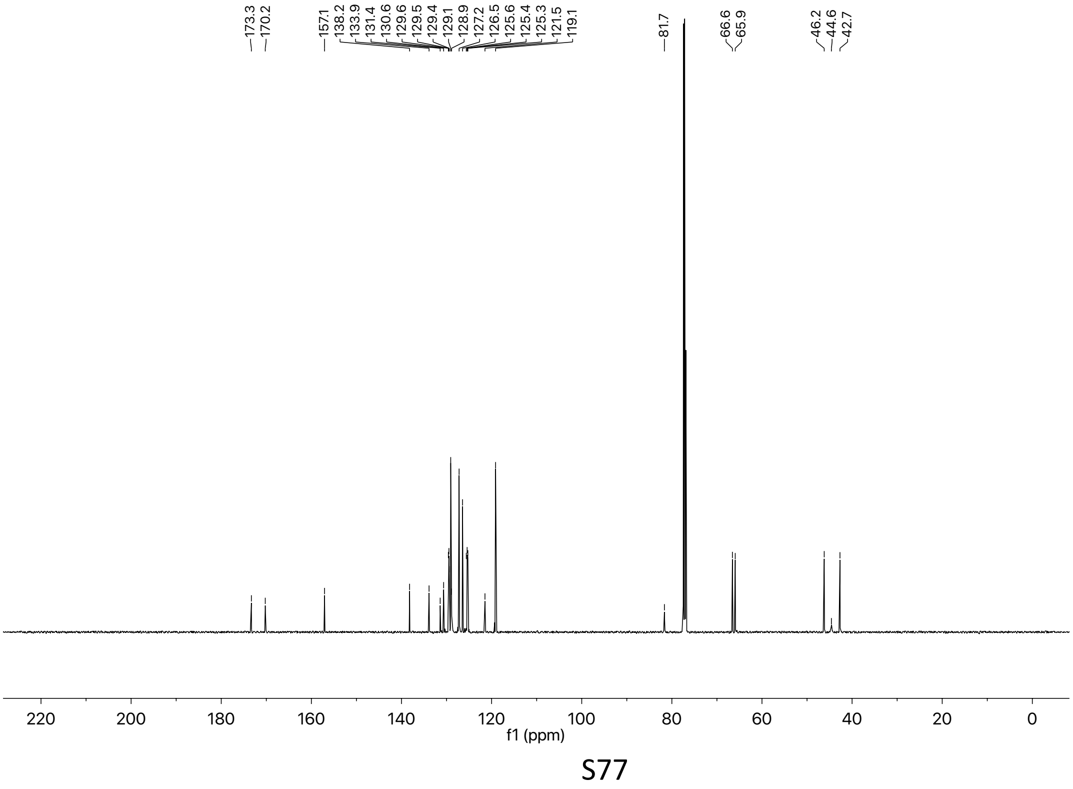

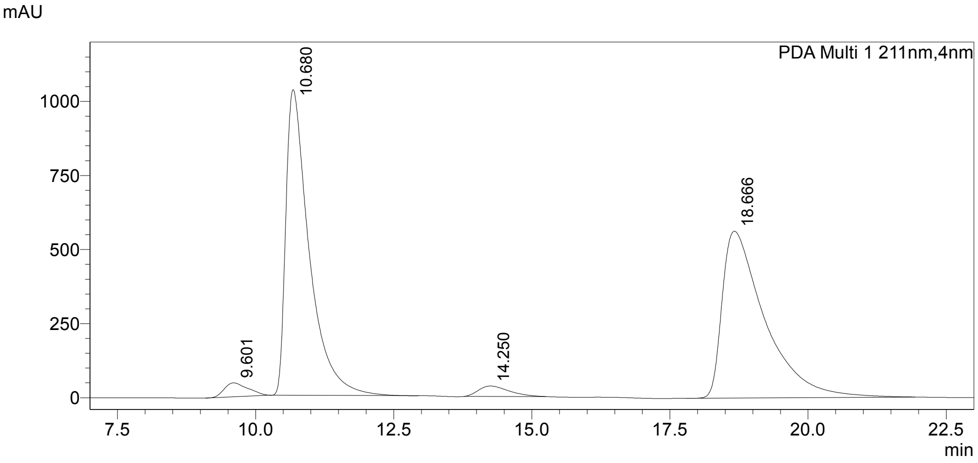

<Peak Table>

PDA Ch1 211nm

Peak#	Ret. Time	Area%
1	9.601	2.207
2	10.680	47.929
3	14.250	2.143
4	18.666	47.721
Total		100.000

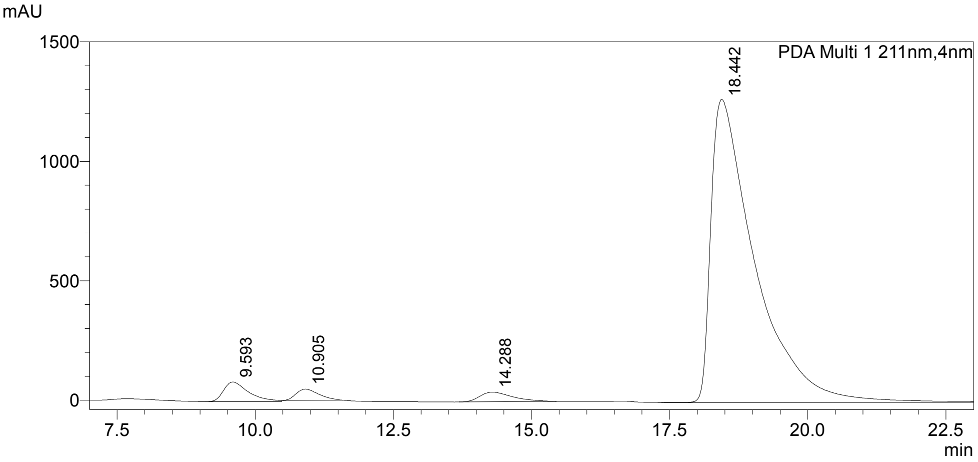

<Peak Table>

PDA Ch1 211nm

Peak#	Ret. Time	Area%
1	9.593	3.410
2	10.905	1.766
3	14.288	2.166
4	18.442	92.658
Total		100.000

5.19. (1′*R*,4*R*)- and (1′*S*,4*R*)-2-(*tert*-Butyl)-4-hydroxy-5-methyl-4-(2-morpholino-2-oxo-1-phenylethyl)-2,4-dihydro-3*H*-pyrazol-3-one **34**

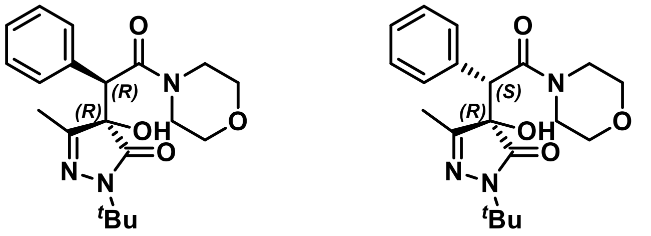

To a solution of 1-(*tert*-butyl)-3-methyl-1*H*-pyrazole-4,5-dione (42.1 mg, 0.25 mmol), 2-phenylacetic anhydride (95.4 mg, 0.375 mmol) and (2*R*,3*S*)-HyperBTM (3.9 mg, 0.125 mmol) were dissolved in ethyl acetate (6 ml, 0.04 M) at 0 °C. Diisopropylethylamine (54 µl, 0.31 mmol) was added and the reaction mixture was stirred at 0 °C for 3 h. Morpholine (66 µl, 0.75 mmol) was added and the reaction mixture was stirred for 16 h at rt. The reaction mixture was diluted with ethyl acetate and washed sequentially with 1 M aq. HCl (×2), sat. aq. $NaHCO_3$ (×2), and brine (×1). The organic layer was dried over $MgSO_4$, filtered, and concentrated *in vacuo*. Purification by column chromatography (*n*-hexane : ethyl acetate 4:1 to 1:1) gave the product as a single diastereomer (48.5 mg, 52%) as a colourless semi-solid. $[\alpha]_D^{20}$ +180.1 (*c* 0.49, $CHCl_3$); **HPLC Analysis:** Chiralpak AD-H (85:15 hexane:isopropanol, flow rate 2.00 ml·min^{-1}, 211 nm, 30 °C) t_R (1′*R*,4*R*)-**34**: 4.6 min, t_R (1′*S*,4*S*)-**34**: 3.8 min, >99:1 er; **IR** ν_{max} (film) 3366 (O-H), 2974 (C-H), 2926 (C-H), 2859 (C-H), 1705 (C=O, pyrazolone), 1624, 1435, 1366, 1225, 1217, 1113; **^{1}H NMR** (500 MHz, $CDCl_3$) δ_H: 1.32 (9H, s, NC(C*H*$_3$)$_3$), 1.95 (3H, s, C(5)C*H*$_3$), 3.05-3.16 (2H, m, NC*H*^{A}H^{B}CH$_2$ + NCH$_2$C*H*^{A}H^B), 3.30 (1H, ddd, J_{HH} 14.0, 7.2, 3.1, NCH^A*H*^{B}CH$_2$), 3.46 (1H, ddd, J_{HH} 11.3, 6.4, 3.1, NCH$_2$CH^A*H*^{B}), 3.52-3.66 (2H, m, NC*H*^{C}H^{D}CH$_2$ + NCH$_2$C*H*^{C}H^D), 3.66-3.75 (2H, m, NCH^C*H*^{D}CH$_2$ + NCH$_2$CH^C*H*^{D}), 4.16 (1H, s, C(1′)*H*), 5.67 (1H, br s, O*H*), 7.23 (2H, dd, J_{HH} 6.7, 3.0, C(1′)HArC(2,6)*H*), 7.28-7.34 (3H, m, C(1′)HArC(3,4,5)*H*); **^{13}C{^{1}H} NMR** (126 MHz, $CDCl_3$) δ_C: 16.0 (C(5)*C*H$_3$), 28.0 (NC(*C*H$_3$)$_3$, 42.4 (N*C*H^{C}H^{D}CH$_2$), 46.5 (N*C*H^{A}H^{B}CH$_2$), 53.4 (*C*(1′)H), 57.4 (N*C*(CH$_3$)$_3$), 66.0 (NCH$_2$*C*H^{A}H^B), 66.7 (NCH$_2$*C*H^{C}H^D), 81.6 (*C*(4)-OH), 128.7 (C(1′)HAr*C*(4)H), 128.9 (C(1′)HAr*C*(2,6)H), 129.1 (C(1′)HAr*C*(3,5)H), 132.3 (C(1′)HAr*C*(1)), 158.2 (*C*(5)=N), 170.4 (C(2′)=O), 172.9 (*C*(3)=O); **HRMS** (ESI$^+$) $C_{20}H_{28}N_3O_4$ [M+H]$^+$ found 374.2064, requires 374.20733 (–2.8 ppm).

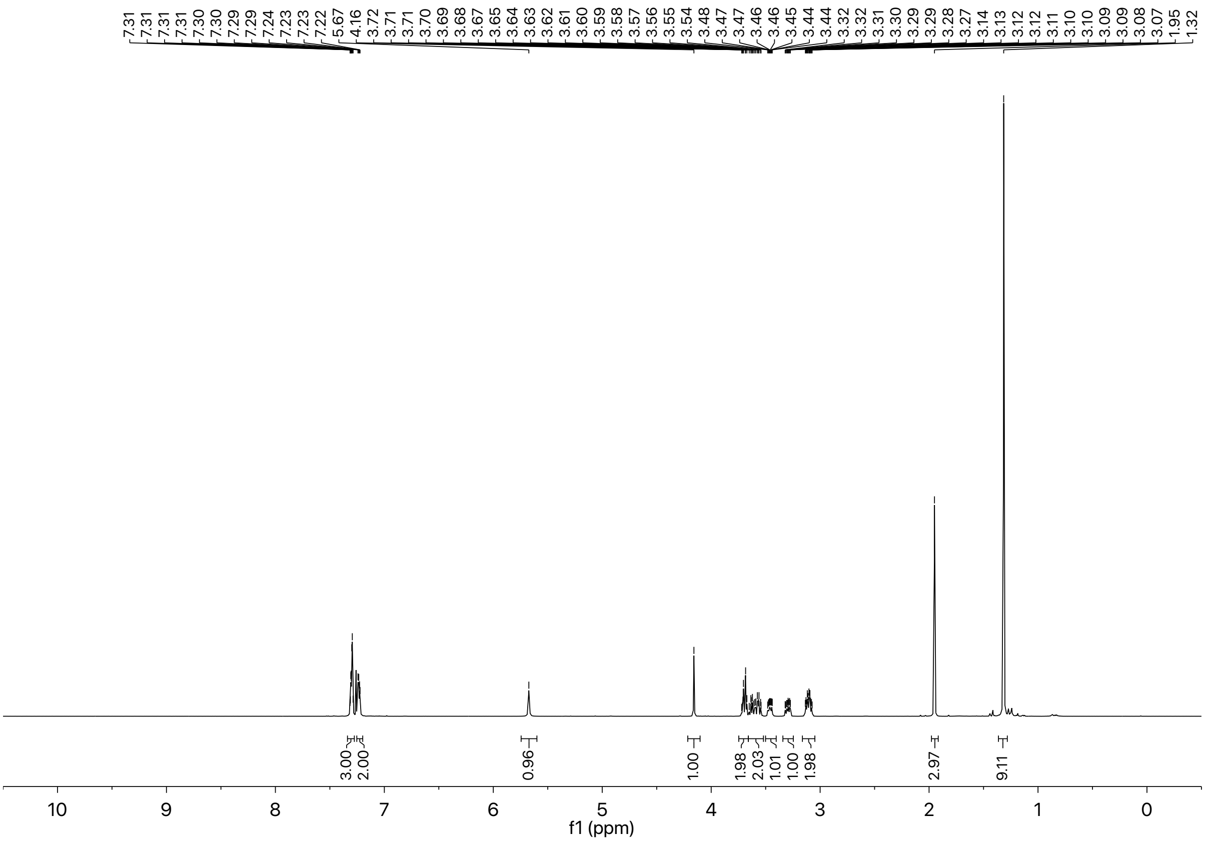
7.31
7.31
7.31
7.30
7.30
7.29
7.29
7.24
7.23
7.23
7.22
5.67
4.16
3.72
3.71
3.70
3.69
3.68
3.67
3.65
3.64
3.63
3.62
3.61
3.60
3.59
3.58
3.57
3.56
3.55
3.54
3.48
3.47
3.46
3.46
3.45
3.44
3.44
3.32
3.32
3.31
3.30
3.29
3.29
3.28
3.27
3.14
3.13
3.12
3.12
3.11
3.10
3.10
3.09
3.09
3.08
3.07
1.95
1.32
3.00
2.00
0.96
1.00
1.98
2.03
1.01
1.00
1.98
2.97
9.11
f1 (ppm)

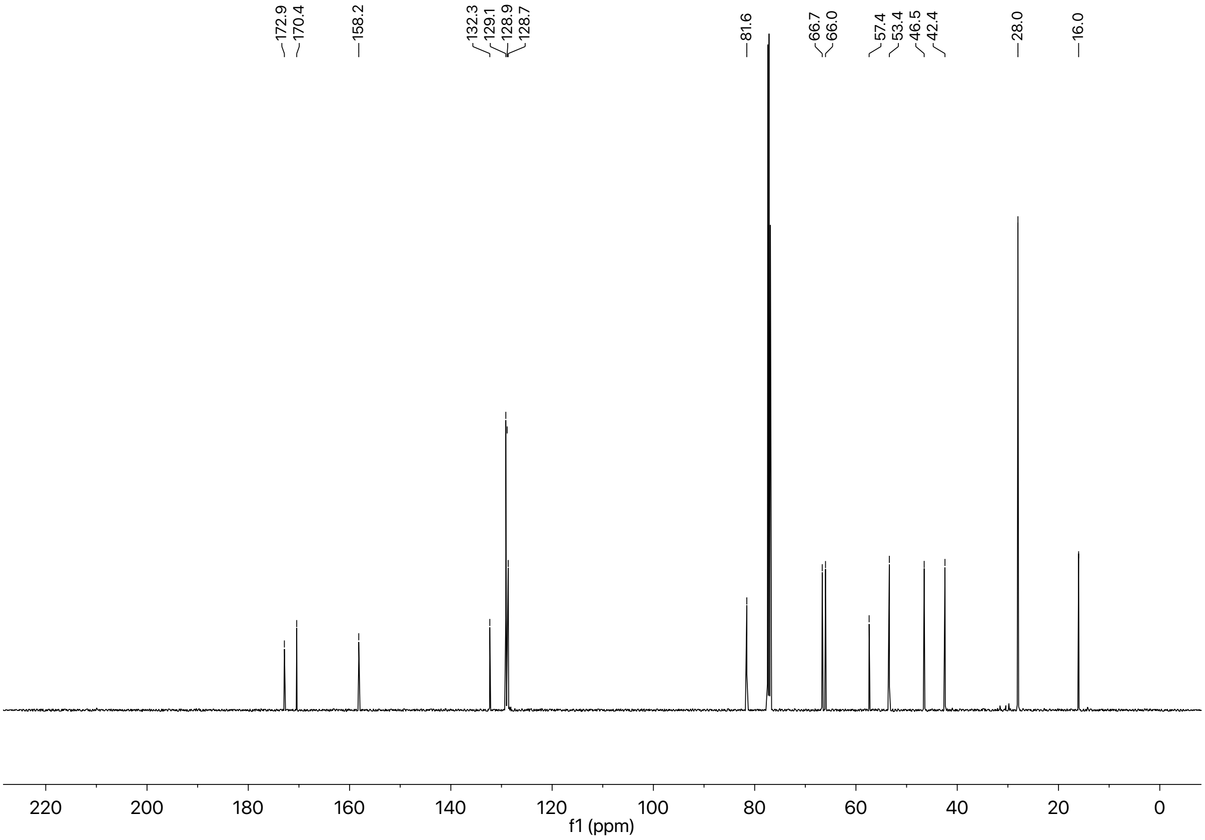
172.9
170.4
158.2
132.3
129.1
128.9
128.7
81.6
66.7
66.0
57.4
53.4
46.5
42.4
28.0
16.0
f1 (ppm)

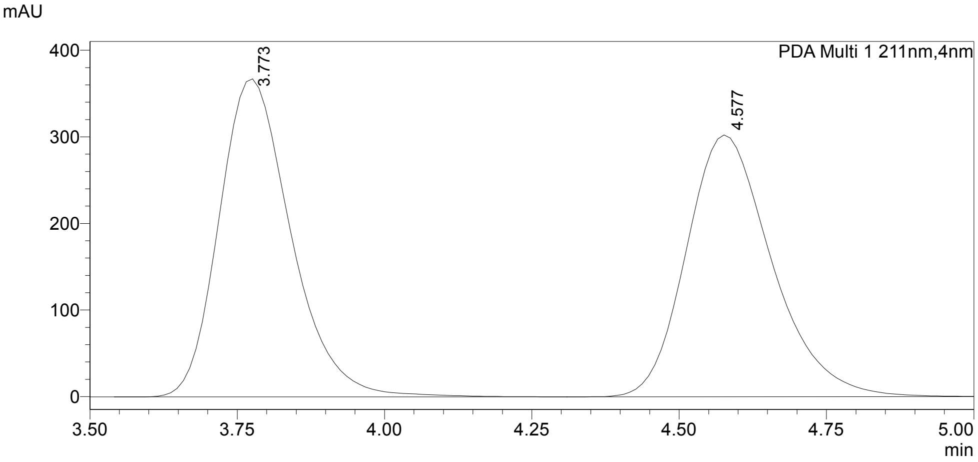

<Peak Table>

PDA Ch1 211nm

Peak#	Ret. Time	Area%
1	3.773	50.489
2	4.577	49.511
Total		100.000

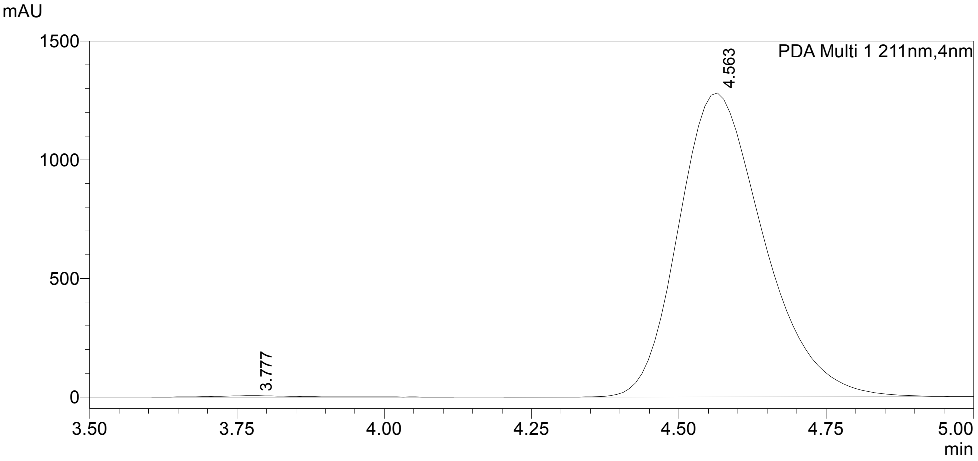

<Peak Table>

PDA Ch1 211nm

Peak#	Ret. Time	Area%
1	3.777	0.349
2	4.563	99.651
Total		100.000

5.20. (1′*R*,4*R*)- and (1′*S*,4*R*)-2-(*tert*-Butyl)-4-hydroxy-5-methyl-4-(2-morpholino-1-(naphtha-1-yl)-2-oxoethyl)-2,4-dihydro-3*H*-pyrazol-3-one **35**

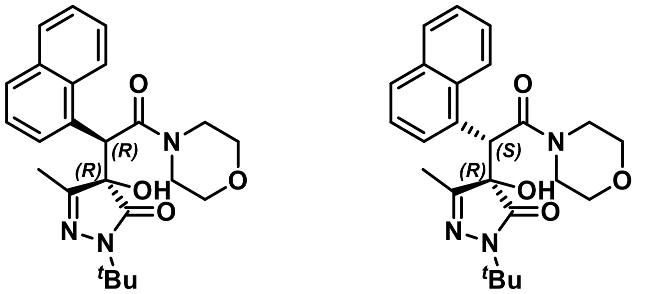

To a solution of 1-(*tert*-butyl)-3-methyl-1*H*-pyrazole-4,5-dione (42.1 mg, 0.25 mmol), 2-(naphth-1-yl)acetic anhydride (132.9 mg, 0.375 mmol) and (2*R*,3*S*)-HyperBTM (3.9 mg, 0.125 mmol) were dissolved in ethyl acetate (6 ml, 0.04 M) at 0 °C. Diisopropylethylamine (54 µl, 0.31 mmol) was added and the reaction mixture was stirred at 0 °C for 3 h. Morpholine (66 µl, 0.75 mmol) was added and the reaction mixture was stirred for 16 h at rt. The reaction mixture was diluted with ethyl acetate and washed sequentially with 1 M aq. HCl (×2), sat. aq. $NaHCO_3$ (×2), and brine (×1). The organic layer was dried over $MgSO_4$, filtered, and concentrated *in vacuo*. Purification by column chromatography (*n*-hexane : ethyl acetate 4:1 to 1:1) gave the major and minor diastereomer (95.7 mg, 90%, >95:5 dr) as an inseparable mixture as a white amorphous solid. $[\alpha]_D^{20}$ +233.4 (*c* 1.00, $CHCl_3$) **IR** ν_{max} (film) 3387 (O-H), 3051, 2974 (C-H), 2926 (C-H), 2857 (C-H), 1707 (C=O, pyrazolone), 1626, 1435, 1366, 1223, 1215, 1113, 783; **HRMS** (ESI^+) $C_{24}H_{30}N_3O_4$ $[M+H]^+$ found 424.2220, requires 424.2231 (–2.7 ppm). *Data for major diastereomer anti*-**106**: **HPLC Analysis:** Chiralpak AD-H (85:15 hexane:isopropanol, flow rate 2.00 ml·min^{-1}, 211 nm, 30 °C) t_R (1′*R*,4*R*)-**35**: 6.3 min, t_R (1′*S*,4*S*)-**35**: 4.9 min, >99:1 er; **^{1}H NMR** (500 MHz, $CDCl_3$) δ_H: 1.02 (9H, s, $NC(CH_3)_3$), 1.84 (3H, s, $C(5)CH_3$), 2.77 (1H, ddd, J_{HH} 11.5, 7.2, 3.0, $NCH_2CH^AH^B$), 2.91 (1H, ddd, J_{HH} 13.5, 6.1, 3.0, $NCH^AH^BCH_2$), 3.20 (1H, ddd, J_{HH} 13.5, 7.2, 3.1, $NCH^AH^BCH_2$), 3.27 (1H, ddd, J_{HH} 11.5, 6.1, 3.1, $NCH_2CH^AH^B$), 3.50 (1H, ddd, J_{HH} 11.3, 7.1, 2.9, $NCH_2CH^CH^D$), 3.59 (1H, ddd, J_{HH} 13.0, 7.1, 2.8, $NCH^CH^DCH_2$), 3.67 (1H, ddd, J_{HH} 11.3, 5.9, 2.8, $NCH_2CH^CH^D$), 3.73 (1H, ddd, J_{HH} 13.0, 5.9, 2.9, $NCH^CH^DCH_2$), 4.94 (1H, s, C(1′)*H*), 6.13 (1H, br s, O*H*), 7.45 (1H, app t, J_{HH} 7.7, C(1′)HArC(3)*H*), 7.51 (1H, app t, J_{HH} 7.3, C(1′)HArC(7)*H*), 7.55-7.62 (2H, m, C(1′)HArC(2)*H* + ArC(6)*H*), 7.83 (1H, d, J_{HH} 8.2, C(1′)HArC(4)*H*), 7.86 (1H, d, J_{HH} 8.0, C(1′)HArC(8)*H*), 8.07 (1H, d, J_{HH} 8.6, C(1′)HArC(5)*H*); **^{13}C{^{1}H} NMR** (126 MHz, $CDCl_3$) δ_C: 15.9 (C(5)*C*H_3), 27.7 (NC(*C*H_3)$_3$), 42.6 (N*C*$H^CH^DCH_2$), 46.3 (N*C*$H^AH^BCH_2$), 47.4 (*C*(1′)H), 57.1 (N*C*(CH_3)$_3$), 65.8 (NCH_2*C*H^AH^B), 66.6 (NCH_2*C*H^CH^D), 81.6 (*C*(4)=O), 122.1 (C(1′)HAr*C*(5)H), 125.3 (C(1′)HAr*C*(3)H), 126.3 (C(1′)HAr*C*(7)H), 127.2 (C(1′)HAr*C*(6)H), 127.6 (C(1′)HAr*C*(2)H), 128.3 (C(1′)HAr*C*(1)), 129.5 (C(1′)HAr*C*(8)H), 129.5

(C(1′)HAr*C*(4)H), 130.9 (C(1′)HAr*C*(8a)), 133.9 (C(1′)HAr*C*(4a)), 158.4 (*C*(5)=N), 171.3 (*C*(2′)=O), 173.3 (*C*(3)=O); *Data for minor diastereomer syn*-**106**: **HPLC Analysis:** Chiralpak AD-H (85:15 hexane:isopropanol, flow rate 2.00 ml·min^{-1}, 211 nm, 30 °C) t_R (1′*S*,4*R*)-**35**: 3.9 min, t_R (1′*R*,4*S*)-**35**: 3.6 min (not detected); **^{1}H NMR** (500 MHz, $CDCl_3$) (*selected*) δ_H: 1.31 (9H, s, NC(C*H*$_3$)$_3$, 2.03 (3H, s, C(5)C*H*$_3$), 4.72 (1H, s, C(1′)*H*).

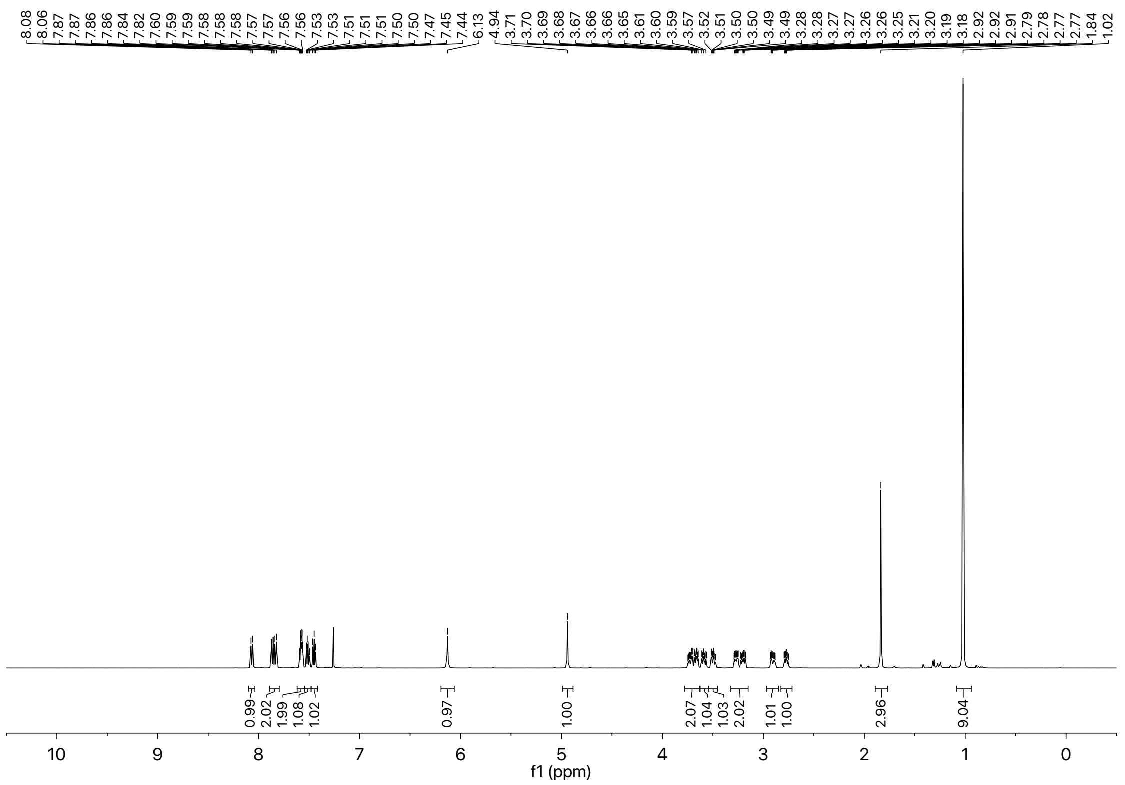

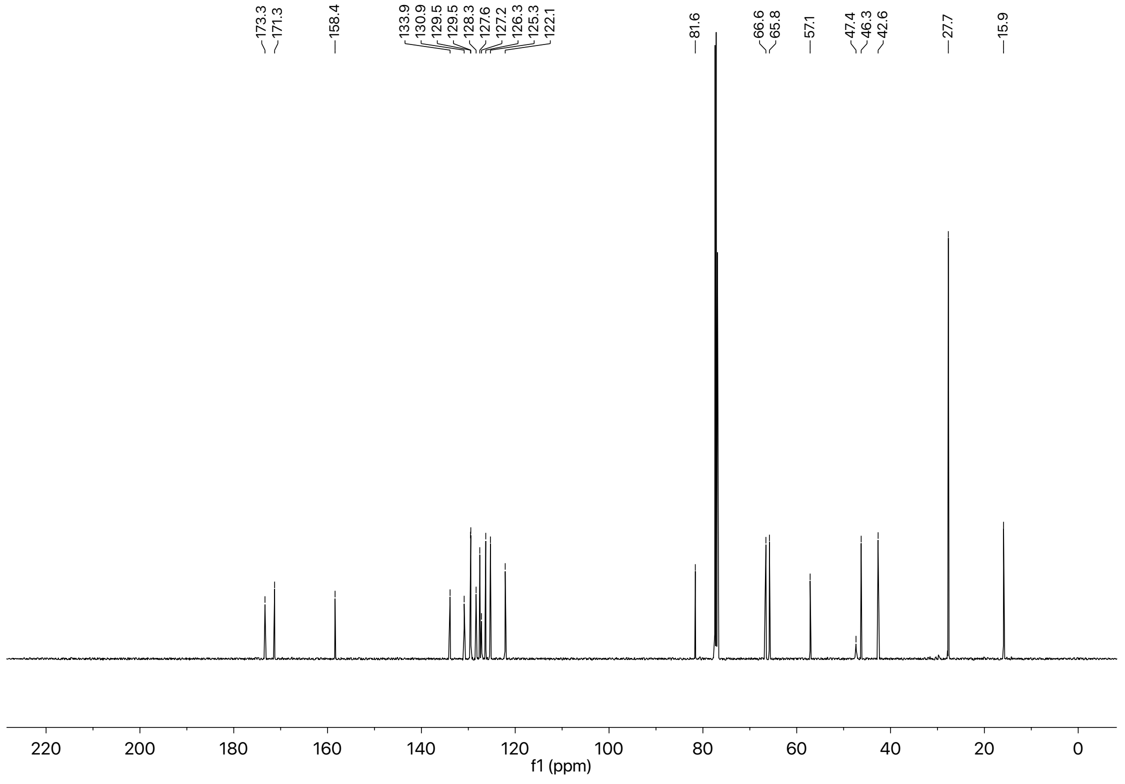

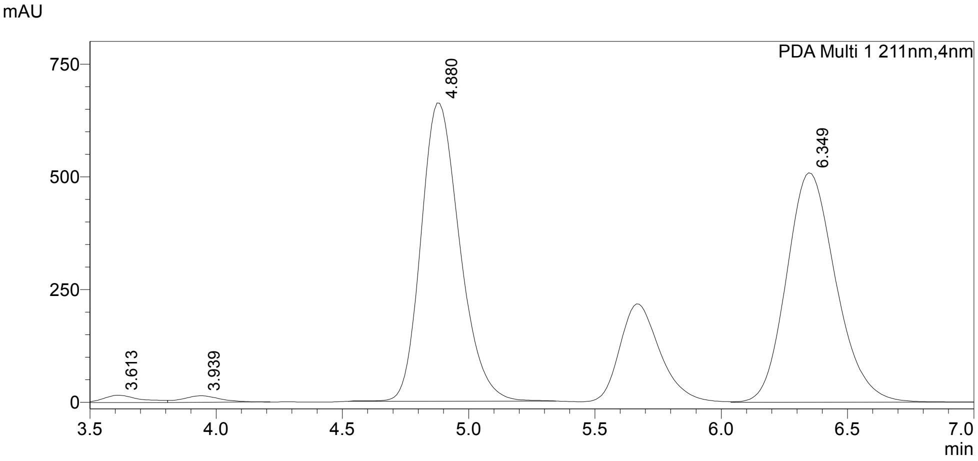

<Peak Table>

PDA Ch1 211nm

Peak#	Ret. Time	Area%
1	3.613	1.148
2	3.939	0.996
3	4.880	49.382
4	6.349	48.475
Total		100.000

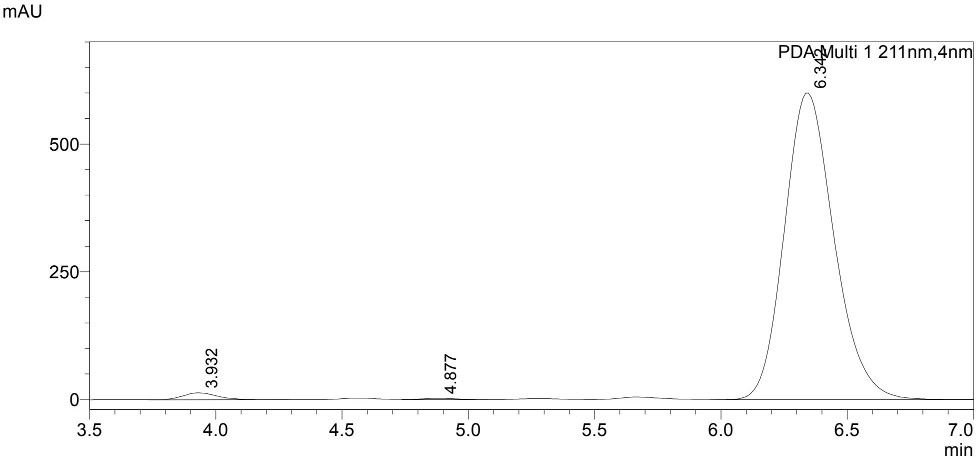

<Peak Table>

PDA Ch1 211nm

Peak#	Ret. Time	Area%
1	3.932	1.552
2	4.877	0.321
3	6.342	98.127
Total		100.000

5.21. (1′*R*,4*R*)- and (1′*S*,4*R*)-2-(*p*-Anisyl)-4-hydroxy-5-methyl-4-(2-morpholino-1-(naphtha-1-yl)-2-oxoethyl)-2,4-dihydro-3*H*-pyrazol-3-one **36**

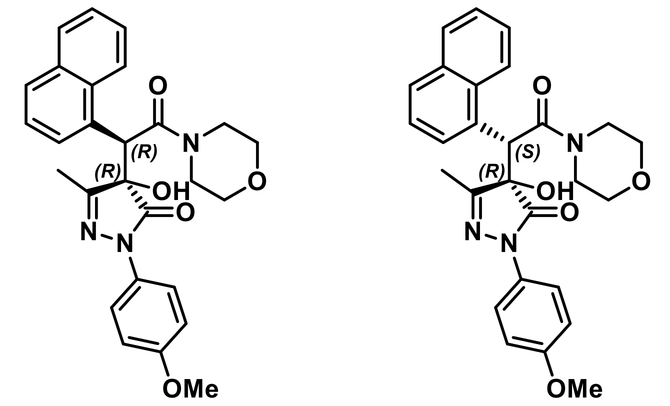

To a solution of 1-(*p*-anisyl)-3-methyl-1*H*-pyrazole-4,5-dione (54.6 mg, 0.25 mmol), 2-(naphth-1-yl)acetic anhydride (132.9 mg, 0.375 mmol) and (2*R*,3*S*)-HyperBTM (3.9 mg, 0.125 mmol) were dissolved in ethyl acetate (6 ml, 0.04 M) at 0 °C. Diisopropylethylamine (54 µl, 0.31 mmol) was added and the reaction mixture was stirred at 0 °C for 3 h. Morpholine (66 µl, 0.75 mmol) was added and the reaction mixture was stirred for 16 h at rt. The reaction mixture was diluted with ethyl acetate and washed sequentially with 1 M aq. HCl (×2), sat. aq. $NaHCO_3$ (×2), and brine (×1). The organic layer was dried over $MgSO_4$, filtered, and concentrated *in vacuo*. Purification by column chromatography (*n*-hexane : ethyl acetate 4:1 to 1:1) gave the major and minor diastereomer (115.3 mg, 97%, >95:5 dr) as an inseparable mixture as a white amorphous solid. $[\alpha]_D^{20}$ +319.9 (*c* 1.00, $CHCl_3$); **IR** ν_{max} (film) 3333 (O-H), 3053, 2963 (C-H), 2918 (C-H), 2857 (C-H), 1713 (C=O, pyrazolone), 1639, 1628, 1508, 1439, 1244, 1113, 1032, 831, 783; **HRMS** (ESI^+) $C_{27}H_{26}N_3O_5Na$ $[M+Na]^+$ found 496.18299, requires 496.18429 (–2.6 ppm). *Data for major diastereomer anti-***36**: **HPLC Analysis:** Chiralpak AD-H (85:15 hexane:isopropanol, flow rate 2.00 ml·min^{-1}, 211 nm, 30 °C) t_R (1′*R*,4*R*)-**36**: 18.1 min, t_R (1′*S*,4*S*)-**36**: 30.0 min, >99:1 er; **^{1}H NMR** (500 MHz, $CDCl_3$) δ_H: 1.52 (3H, s, C(5)CH_3), 2.76 (1H, ddd, J_{HH} 10.7, 7.3, 2.9, $NCH_2CH^AH^B$), 2.96 (1H, ddd, J_{HH} 13.6, 5.8, 2.9, $NCH^AH^BCH_2$), 3.21-3.34 (2H, m, $NCH^AH^BCH_2$ + $NCH_2CH^AH^B$), 3.49 (1H, ddd, J_{HH} 11.5, 7.4, 3.0, $NCH_2CH^CH^D$), 3.59 (1H, ddd, J_{HH} 13.3, 7.4, 3.0, $NCH^CH^DCH_2$), 3.70 (1H, ddd, J_{HH} 11.5, 5.8, 3.0, $NCH_2CH^CH^D$), 3.76-3.85 (2H, m, OCH_3 + $NCH^CH^DCH_2$), 4.92 (1H, s, C(1′)*H*), 6.81-6.88 (2H, m, NAr(3,5)*H*), 7.18 (1H, br s, O*H*), 7.46-7.54 (4H, m, NAr(2,6)*H* + C(1′)HAr*C*(3)H + C(1′)HArC(7)*H*), 7.57 (1H, app t, J_{HH} 7.2, C(1′)HArC(6)*H*), 7.80 (1H, d, J_{HH} 7.3, C(1′)HArC(2)*H*), 7.88 (2H, app t, J_{HH} 7.9, C(1′)HArC(4)*H* + C(1′)HArC(8)*H*), 7.96 (1H, d, J_{HH} 8.5, C(1′)HArC(5)*H*); **^{13}C{^{1}H} NMR** (126 MHz, $CDCl_3$) δ_C: 15.0 (C(5)CH_3), 42.8 (NCH^CH^D), 44.9 (*C*(1′)H), 46.3 (NCH^AH^B), 55.6 (OCH_3), 65.9 ($NCH_2CH^AH^B$), 66.6 ($NCH_2CH^CH^D$), 81.3 (*C*(4)-OH), 114.0 (NAr*C*(3,5)H), 120.9 (NAr*C*(2,6)H), 121.4 (C(1′)HAr*C*(5)H),

125.6 (C(1′)HAr*C*(3)H), 126.3 (C(1′)HAr*C*(7)H), 127.8 (C(1′)HAr*C*(6)H), 127.9 (C(1′)HAr*C*(2)H), 129.8 (C(1′)HAr*C*(4)H), 129.8 (C(1′)HAr*C*(8)H), 130.9 (C(1′)HAr*C*), 130.9 (NAr*C*(1)), 134.0 (C(1′)HAr*C*(4a)), 157.2 (NAr*C*(4)), 160.5 (*C*(5)=N), 171.1 (*C*(2′)=O), 172.3 (*C*(3)=O); *Data for minor diastereomer syn*-**36**: **HPLC Analysis:** Chiralpak AD-H (85:15 hexane:IPA, flow rate 2.00 ml·min^{-1}, 211 nm, 30 °C) t_R (1′*S*,4*R*)-**36**: 13.5 min, t_R (1′*R*,4*S*)-**36**: 15.1 min (not detected); **^{1}H NMR** (500 MHz, $CDCl_3$) (*selected*) δ_H: 2.15 (3H, s, CH_3), 4.86 (1H, s, C(1′)*H*).

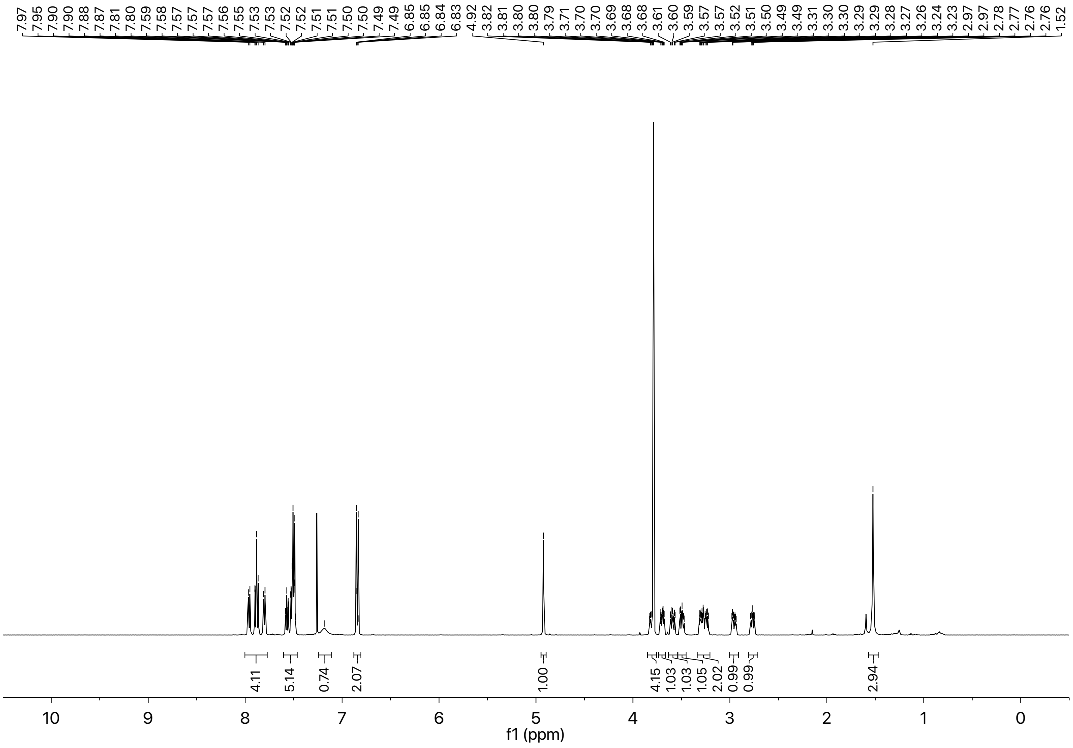

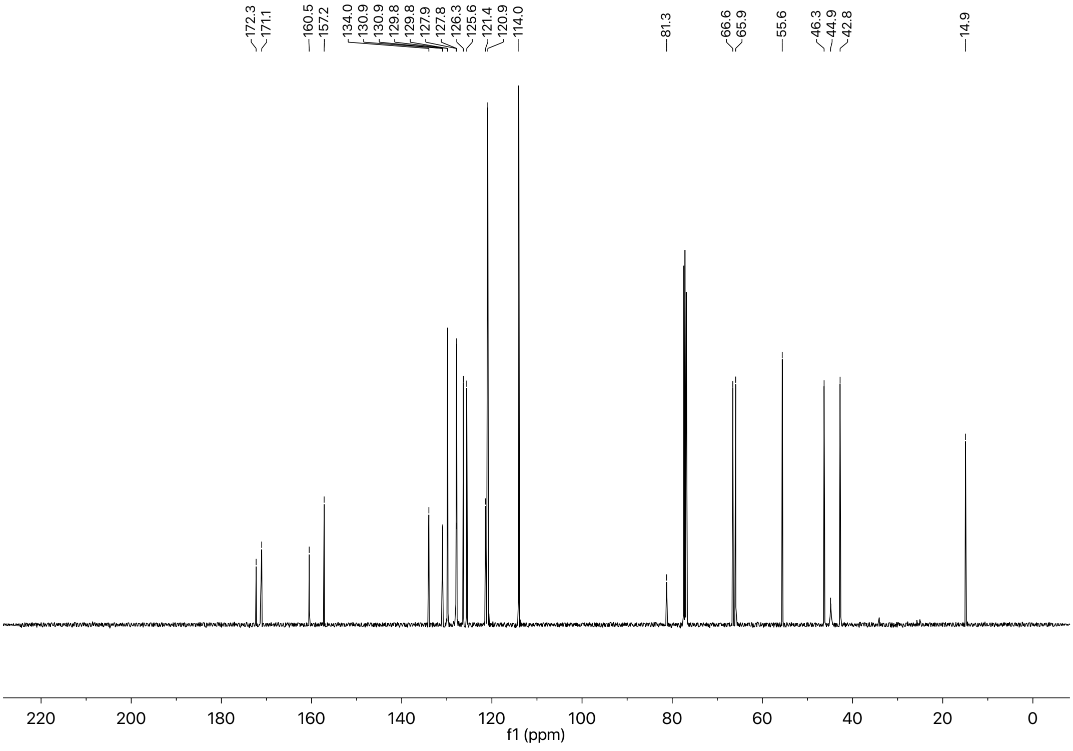

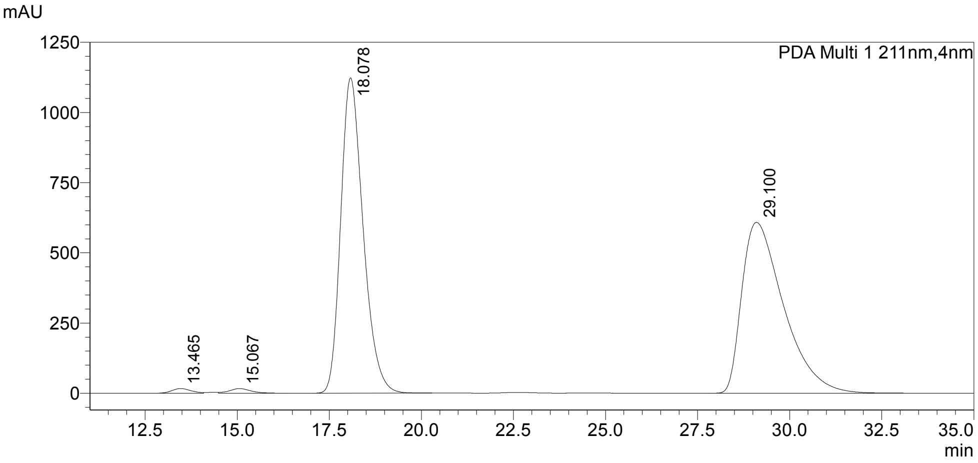

<Peak Table>

PDA Ch1 211nm

Peak#	Ret. Time	Area%
1	13.465	0.622
2	15.067	0.692
3	18.078	49.068
4	29.100	49.618
Total		100.000

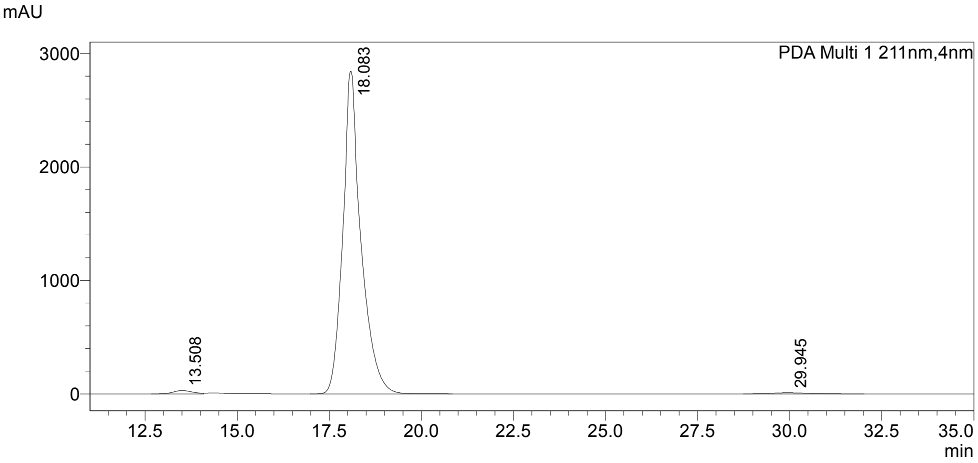

<Peak Table>

PDA Ch1 211nm

Peak#	Ret. Time	Area%
1	13.508	1.054
2	18.083	98.232
3	29.945	0.714
Total		100.000

5.22. (1′*R*,4*R*)- and (1′*S*,4*R*)-2-(*p*-Anisyl)-4-hydroxy-5-methyl-4-(2-morpholino-2-oxo-1-phenylethyl)-2,4-dihydro-3*H*-pyrazol-3-one **37**

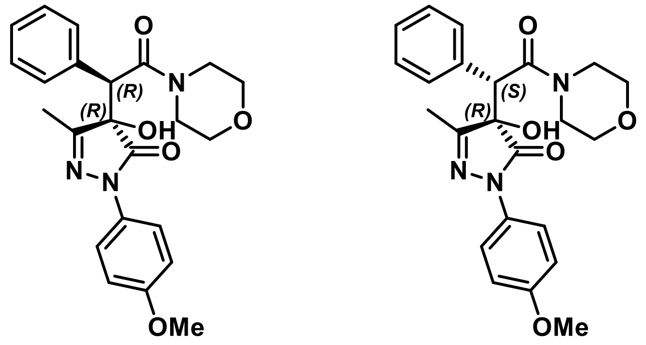

To a solution of 1-(*p*-anisyl)-3-methyl-1*H*-pyrazole-4,5-dione (54.6 mg, 0.25 mmol), 2-phenylacetic anhydride (95.4 mg, 0.375 mmol) and (2*R*,3*S*)-HyperBTM (3.9 mg, 0.125 mmol) were dissolved in ethyl acetate (6 ml, 0.04 M) at 0 °C. Diisopropylethylamine (54 µl, 0.31 mmol) was added and the reaction mixture was stirred at 0 °C for 3 h. Morpholine (66 µl, 0.75 mmol) was added and the reaction mixture was stirred for 16 h at rt. The reaction mixture was diluted with ethyl acetate and washed sequentially with 1 M aq. HCl (×2), sat. aq. $NaHCO_3$ (×2), and brine (×1). The organic layer was dried over $MgSO_4$, filtered, and concentrated *in vacuo*. Purification by column chromatography (*n*-hexane : ethyl acetate 4:1 to 1:1) gave the product as a single diastereomer (76.8 mg, 73%) as a white amorphous solid. $[\alpha]_D^{20}$ +278.3 (*c* 1.00, $CHCl_3$); **HPLC Analysis:** Chiralpak AD-H (95:5 hexane:isopropanol, flow rate 2.00 ml·min^{-1}, 211 nm, 30 °C) t_R (1′*R*,4*R*)-**37**: 57.5 min, t_R (1′*S*,4*S*)-**37**: 45.7 min, >99:1 er; **IR** ν_{max} (film) 3370 (O-H), 2963 (C-H), 2920 (C-H), 2857 (C-H), 1711 (C=O, pyrazolone), 1622, 1510, 1441, 1244, 1113, 1032, 831; **^{1}H NMR** (500 MHz, $CDCl_3$) δ_H: 1.96 (3H, s, C(5)CH_3), 3.08-3.19 (2H, m, NCH^AH^{B}CH$_2$ + NCH$_2$CH^AH^B), 3.33 (1H, ddd, J_{HH} 14.0, 7.3, 3.1, NCH^AH^BCH$_2$), 3.49 (1H, ddd, J_{HH} 11.4, 6.3, 3.1, NCH$_2$CH^AH^B), 3.54-3.68 (2H, m, NCH^CH^{D}CH$_2$ + NCH$_2$CH^CH^D), 3.69-3.79 (2H, m, NCH^CH^DCH$_2$ + NCH$_2$CH^CH^D), 3.80 (3H, s, OCH_3), 4.23 (1H, s, C(1′)*H*), 6.27 (1H, br s, O*H*), 6.85-6.92 (2H, m, NArC(3,5)*H*), 7.24-7.27 (2H, m, C(1′)HArC(2,6)*H*), 7.29-7.34 (3H, m, C(1′)HArC(3,4,5)*H*), 7.53-7.59 (2H, m, NArC(2,6)*H*); **^{13}C{^{1}H} NMR** (126 MHz, $CDCl_3$) δ_C: 15.7 (C(5)*C*H$_3$), 42.5 (N*C*H^{C}H^{D}CH$_2$), 46.6 (N*C*H^{A}H^{B}CH$_2$), 52.7 (*C*(1′)H), 55.6 (O*C*H$_3$), 66.1 (NCH$_2$*C*H^{A}H^B), 66.7 (NCH$_2$*C*H^{C}H^D), 81.7 (*C*(4)-OH), 114.1 (NAr*C*(3,5)H), 121.1 (NAr*C*(2,6)H), 128.7 (C(1′)HAr*C*(2,6)H), 129.0 (C(1′)HAr*C*(4)H), 129.4 (C(1′)HAr*C*(3,5)H), 130.8 (C(1′)HAr*C*(1)), 132.1 (NAr*C*(1)), 157.3 (NAr*C*(4)), 160.7 (*C*(5)=N), 170.3 (*C*(2′)=O), 171.3 (*C*(3)=O); **HRMS** (ESI$^+$) $C_{23}H_{25}N_3O_5$ $[M+H]^+$ found 446.16743, requires 446.16864 (–2.6 ppm).

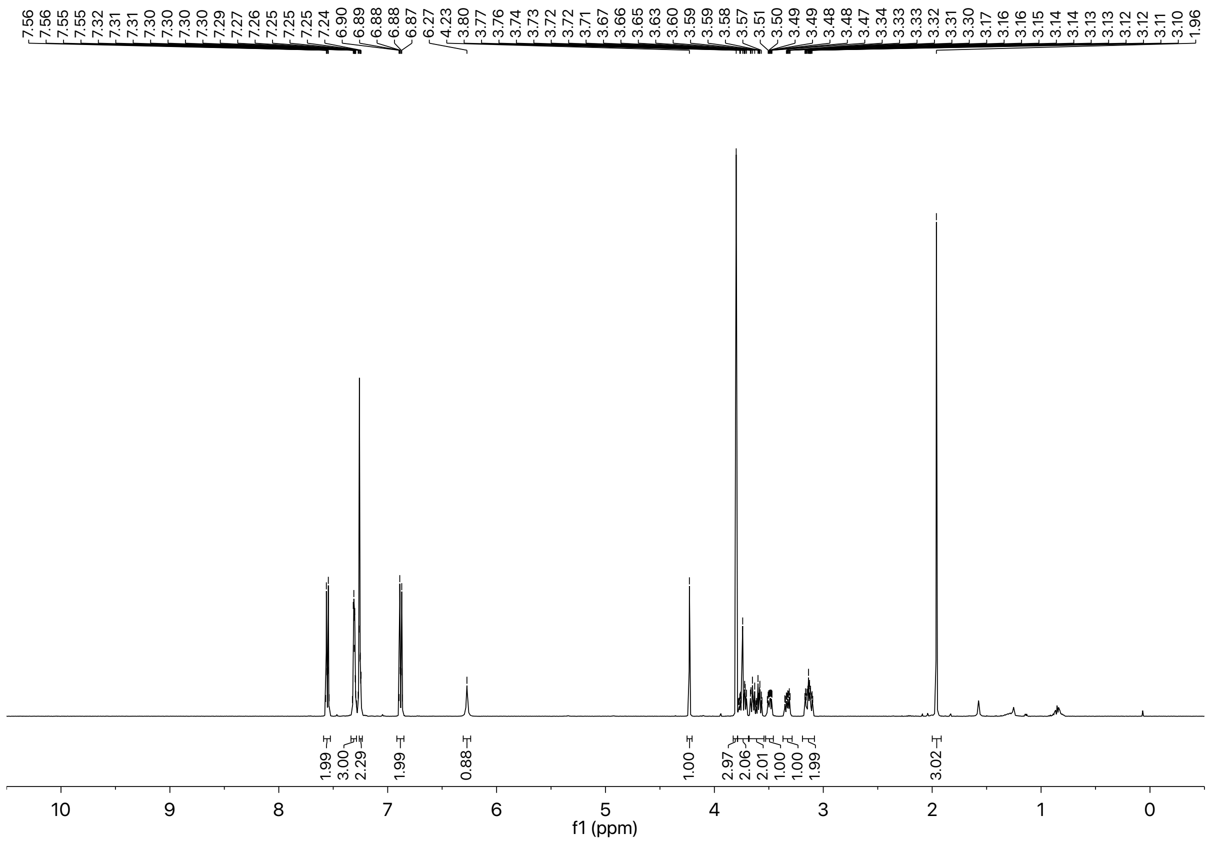

7.56
7.56
7.55
7.32
7.31
7.30
7.30
7.30
7.30
7.29
7.27
7.26
7.25
7.25
7.25
7.24
6.90
6.89
6.88
6.88
6.87
6.27
4.23
3.80
3.77
3.76
3.74
3.73
3.72
3.72
3.71
3.67
3.66
3.65
3.63
3.60
3.59
3.59
3.58
3.57
3.51
3.50
3.49
3.49
3.48
3.48
3.47
3.34
3.33
3.33
3.33
3.32
3.31
3.30
3.17
3.16
3.16
3.15
3.14
3.14
3.13
3.13
3.12
3.12
3.11
3.10
1.96
1.99
3.00
2.29
1.99
0.88
1.00
2.97
2.06
2.01
1.00
1.99
3.02
10
9
8
7
6
5
4
3
2
1
0
f1 (ppm)

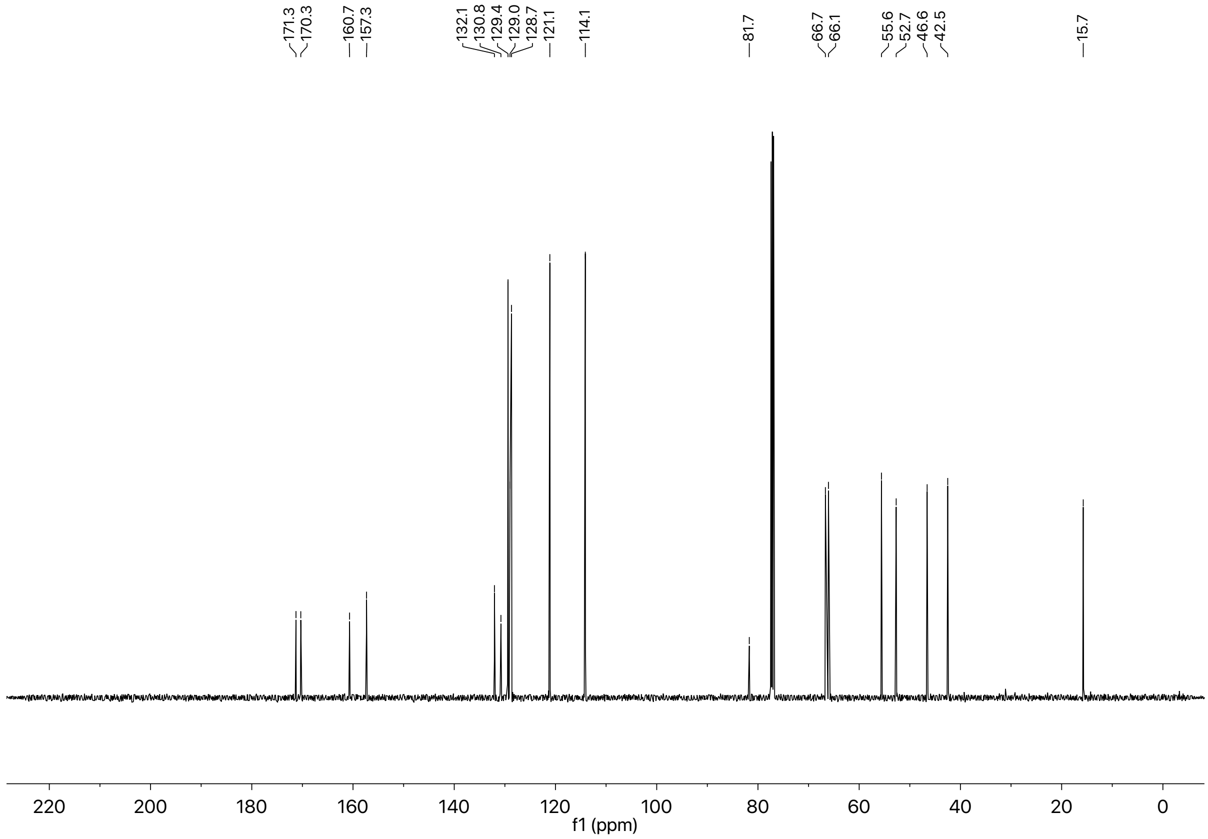

171.3
170.3
160.7
157.3
132.1
130.8
129.4
129.0
128.7
121.1
114.1
81.7
66.7
66.1
55.6
52.7
46.6
42.5
15.7
220
200
180
160
140
120
100
80
60
40
20
0
f1 (ppm)

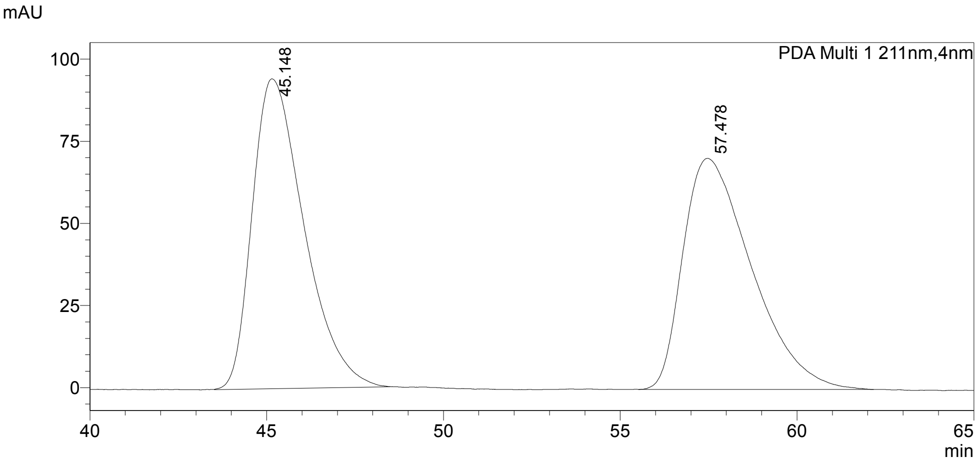

<Peak Table>

PDA Ch1 211nm

Peak#	Ret. Time	Area%
1	45.148	50.363
2	57.478	49.637
Total		100.000

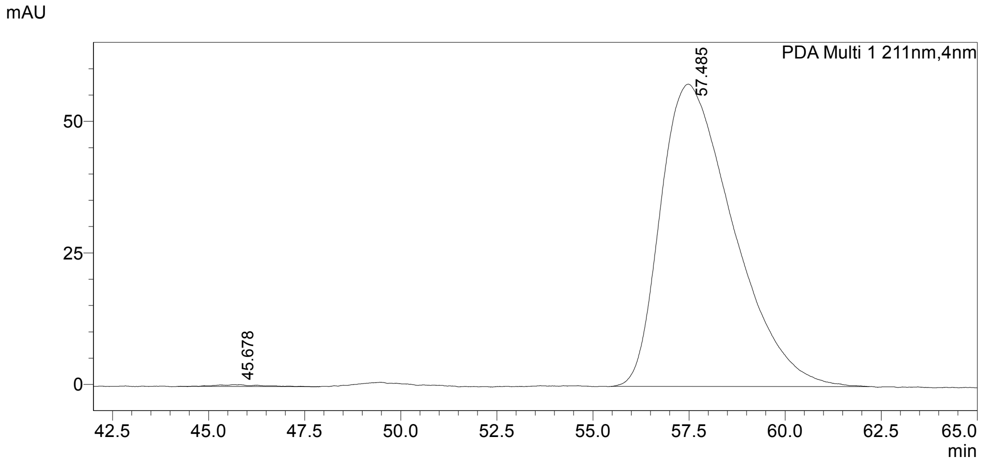

<Peak Table>

PDA Ch1 211nm

Peak#	Ret. Time	Area%
1	45.678	0.385
2	57.485	99.615
Total		100.000

5.23. (1′*R*,4*R*)- and (1′*S*,4*R*)-4-hydroxy-5-methyl-4-(2-morpholino-1-(naphtha-1-yl)-2-oxoethyl)-2-(*p*-trifluoromethylphenyl)-2,4-dihydro-3*H*-pyrazol-3-one **38**

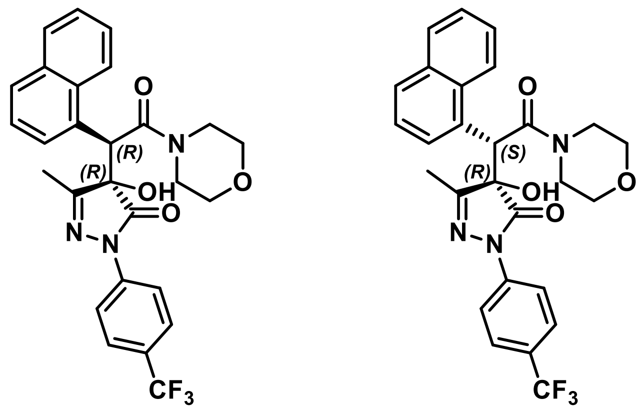

To a solution of 3-methyl-1-(*p*-trifluoromethylphenyl)-1*H*-pyrazole-4,5-dione (54.6 mg, 0.25 mmol), 2-(naphth-1-yl)acetic anhydride (132.9 mg, 0.375 mmol) and (2*R*,3*S*)-HyperBTM (3.9 mg, 0.125 mmol) were dissolved in ethyl acetate (6 ml, 0.04 M) at 0 °C. Diisopropylethylamine (54 µl, 0.31 mmol) was added and the reaction mixture was stirred at 0 °C for 3 h. Morpholine (66 µl, 0.75 mmol) was added and the reaction mixture was stirred for 16 h at rt. The reaction mixture was diluted with ethyl acetate and washed sequentially with 1 M aq. HCl (×2), sat. aq. $NaHCO_3$ (×2), and brine (×1). The organic layer was dried over $MgSO_4$, filtered, and concentrated *in vacuo*. Purification by column chromatography (*n*-hexane : ethyl acetate 4:1 to 1:1) gave the major and minor diastereomer (116.7 mg, 91%, >95:5 dr) as an inseparable mixture as a white amorphous solid. $[\alpha]_D^{20}$ +268.7 (*c* 1.00, $CHCl_3$); **IR** ν_{max} (film) 3323 (O-H), 2967 (C-H), 2924 (C-H), 2859 (C-H), 1724 (C=O, pyrazolone), 1639, 1634, 1612, 1520, 1435 1323, 1163, 1115, 1065, 908, 841, 781; **HRMS** (ESI^+) $C_{27}H_{25}N_3O_4F_3$ $[M+H]^+$ found 512.1779, requires 512.17917 (–2.4 ppm). *Data for major diastereomer anti*-**38**: **HPLC Analysis:** Chiralpak AD-H (92.5:7.5 hexane:isopropanol, flow rate 2.00 ml·min^{-1}, 211 nm, 30 °C) t_R (1′*R*,4*R*)-**38**: 13.7 min, t_R (1′*S*,4*S*)-**38**: 31.1 min, 98.5:1.5 er; **^{1}H NMR** (500 MHz, $CDCl_3$) δ_H: 1.61 (1H, s, CH_3), 2.72 (1H, ddd, J_{HH} 10.2, 7.0, 2.9, $NCH_2CH^AH^B$), 2.92 (1H, ddd, J_{HH} 13.4, 5.5, 2.9, $NCH^AH^BCH_2$), 2.17-3.29 (2H, m, $NCH^AH^BCH_2$ + $NCH_2CH^AH^B$), 3.47 (1H, ddd, J_{HH} 11.5, 7.5, 3.0, $NCH_2CH^CH^D$), 3.55 (1H, ddd, J_{HH} 13.3, 7.5, 3.0, $NCH^CH^DCH_2$), 3.66 (1H, ddd, J_{HH} 11.5, 5.7, 3.0, $NCH_2CH^CH^D$), 3.79 (1H, ddd, J_{HH} 13.3, 5.7, 3.0, $NCH^CH^DCH_2$), 4.94 (1H, s, C(1′)*H*), 6.96 (1H, br s, O*H*), 7.46-7.61 (5H, m, C(1′)HArC(3)*H* + NArC(3,5)*H* + C(1′)HAr*C*(6)H +C(1′)HAr*C*(7)H), 7.77 (1H, d, J_{HH} 7.2, C(1′)HArC(2)*H*), 7.83-7.90 (4H, m, NArC(2,6)*H* + C(1′)HArC(4)*H* + C(1′)HAr*C*(8)H), 7.94 (1H, d, J_{HH} 8.6, C(1′)HArC(5)*H*); **^{13}C{^{1}H} NMR** (126 MHz, $CDCl_3$) δ_C: 14.8 (CH_3), 42.7 ($NCH^CH^DCH_2$), 45.2 (*C*(1′)H), 46.2 ($NCH^AH^BCH_2$), 65.8 ($NCH_2CH^AH^B$), 66.5 ($NCH_2CH^CH^D$), 81.1 (*C*(4)-OH), 118.2 (NAr*C*(2,6)), 121.3 (C(1′)HAr*C*(5)H), 124.2 (q, $^1J_{CF}$ 272.2, ArCF_3) 125.5 (C(1′)HAr*C*(3)H), 126.1 (q,

$^3J_{CF}$ 3.8, NAr*C*(3,5)H), 126.3 (C(1′)HAr*C*(7)H), 126.6 (q, $^2J_{CF}$ 32.5, NAr*C*(4)CF_3), 127.3 (C(1′)HAr*C*), 127.8 (C(1′)HAr*C*(6)H), 128.1 (C(1′)HAr*C*(2)H), 129.8 (C(1′)HAr*C*(4)H), 129.9 (C(1′)HAr*C*(8)), 130.9 (NAr*C*(1)), 134.0 (C(1′)HAr*C*), 140.4 (NAr*C*(1)), 161.2 (*C*(5)=N), 170.6 (C(2′)=O), 172.9 (*C*(3)=O); **^{19}F{^{1}H} NMR** (377 MHz, $CDCl_3$) δ_F: −62.12 (C*F*$_3$); *Data for minor diastereomer syn-***38**: **HPLC Analysis:** Chiralpak AD-H (92.5:7.5 hexane:isopropanol, flow rate 2.00 ml·min^{-1}, 211 nm, 30 °C) t_R (1′*S*,4*R*)-**38**: 15.7 min, t_R (1′*R*,4*S*)-**38**: 16.4 min (not detected); **^{1}H NMR** (500 MHz, $CDCl_3$) (*selected*) δ_H: 2.12 (3H, s, C*H*$_3$), 4.91 (1H, s, C(1′)*H*), 5.93 (1H, br s, O*H*); **^{13}C{^{1}H} NMR** (126 MHz, $CDCl_3$) (*selected*) δ_C: 15.3 (*C*H$_3$), 120.9 (Ar*C*H), 127.0 (Ar*C*H); **^{19}F NMR** (377 MHz, $CDCl_3$) δ_F: −62.07 (C*F*$_3$).

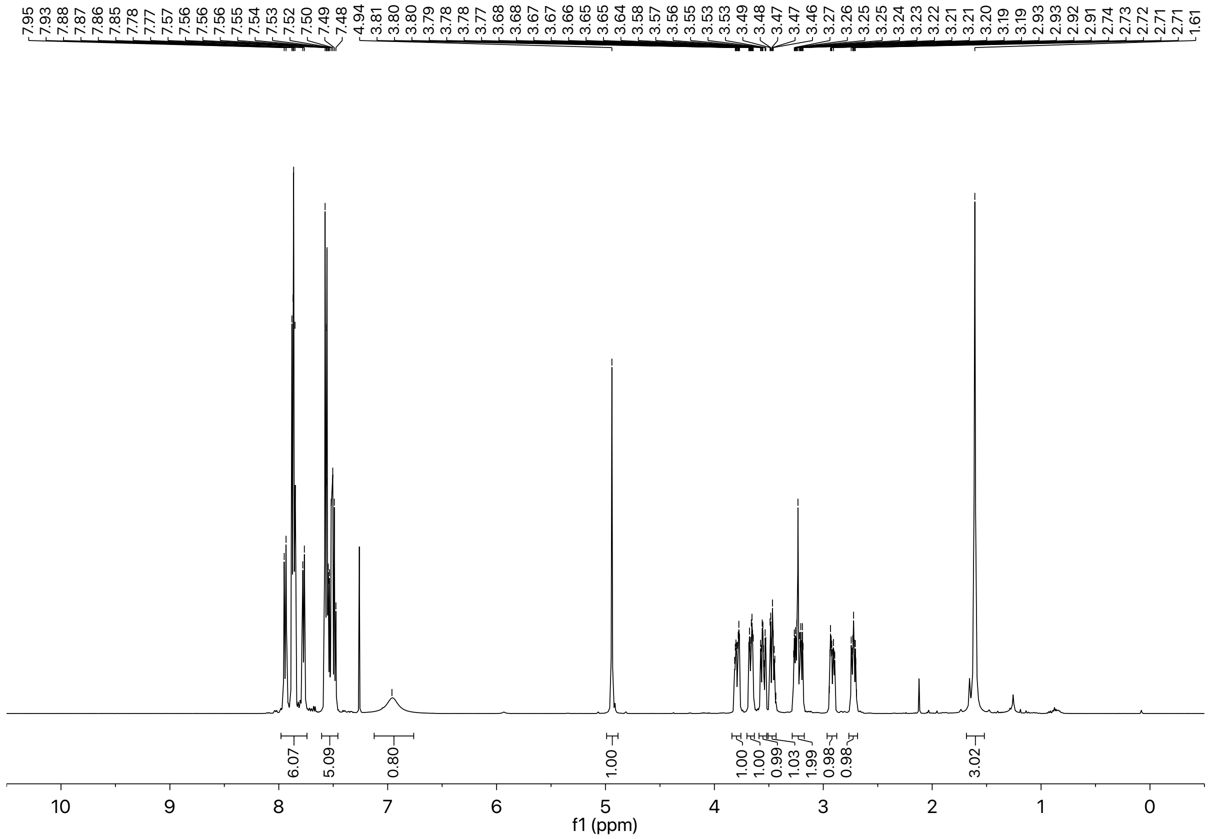

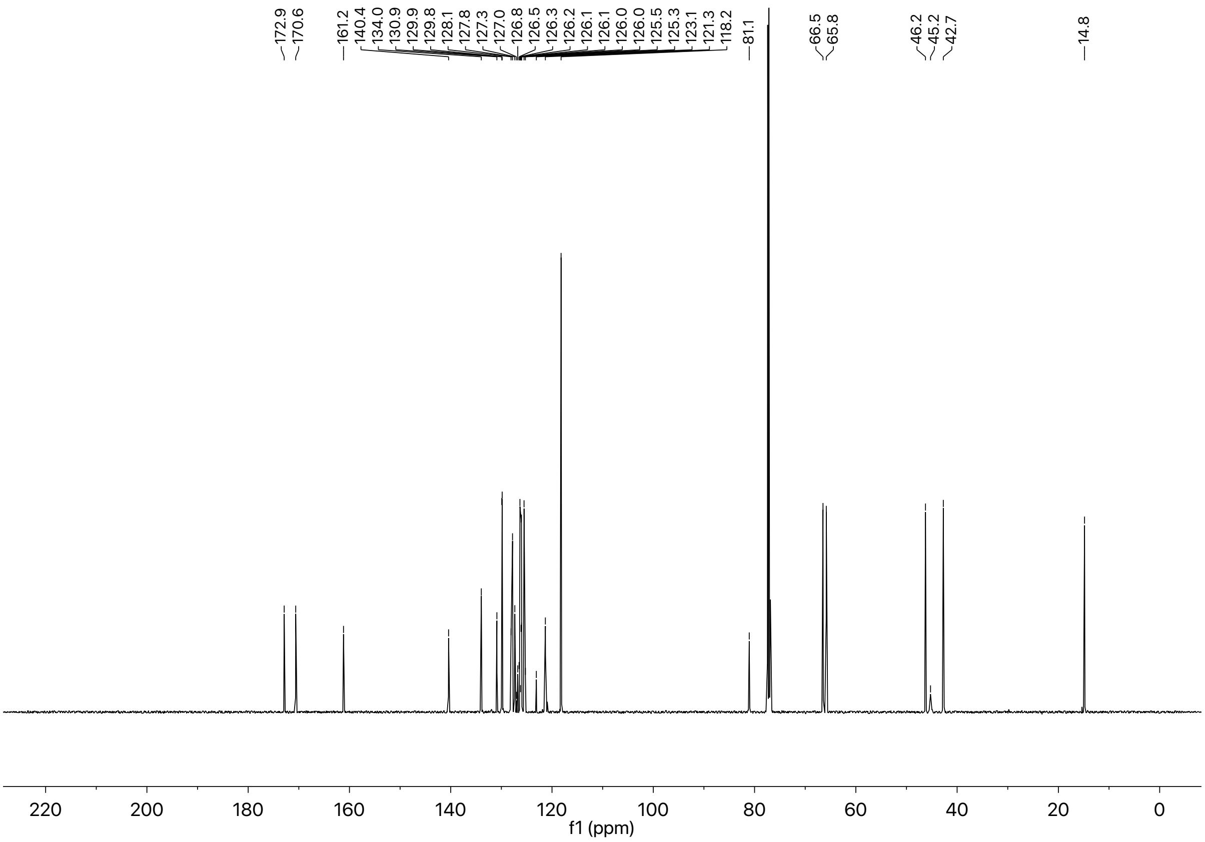

172.9
170.6
161.2
140.4
134.0
130.9
129.9
129.8
128.1
127.8
127.3
127.0
126.8
126.5
126.3
126.2
126.1
126.1
126.0
126.0
125.5
125.3
123.1
121.3
118.2
81.1
66.5
65.8
46.2
45.2
42.7
14.8
220
200
180
160
140
120
100
80
60
40
20
0
f1 (ppm)

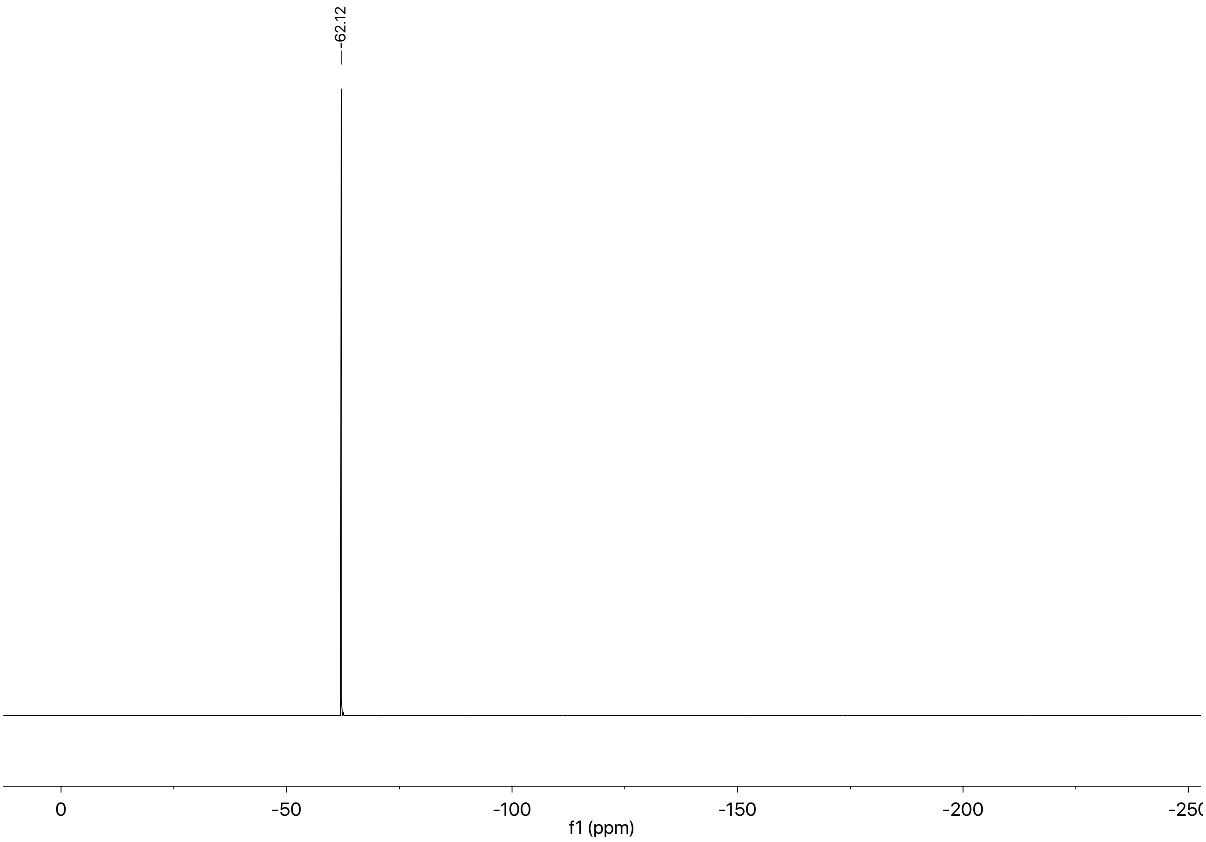

-62.12
0
-50
-100
-150
-200
f1 (ppm)

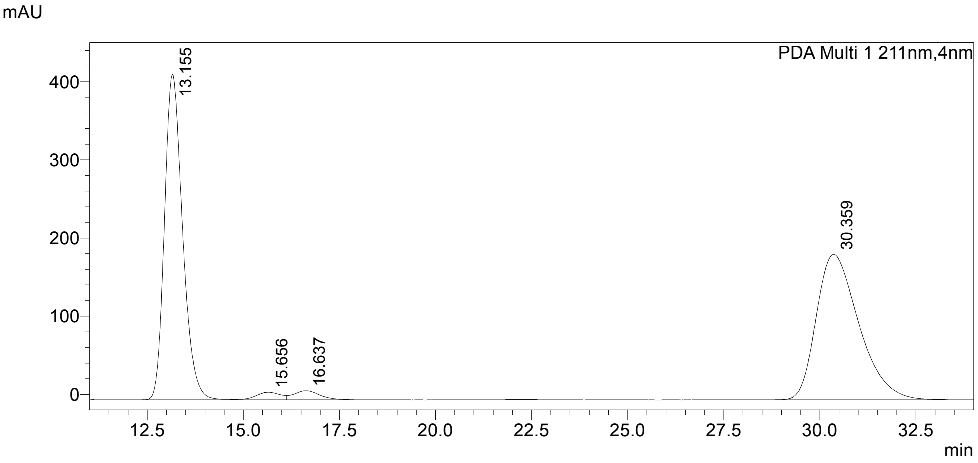

<Peak Table>

PDA Ch1 211nm

Peak#	Ret. Time	Area%
1	13.155	47.137
2	15.656	1.489
3	16.637	1.873
4	30.359	49.502
Total		100.000

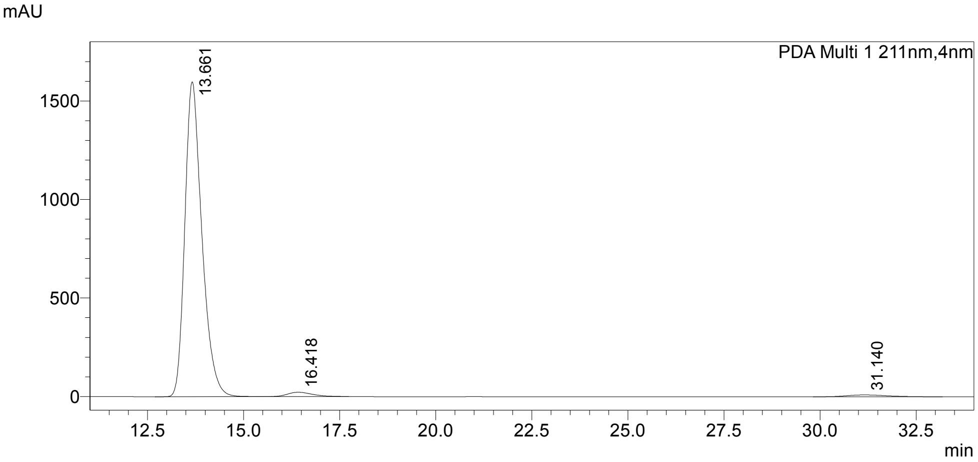

<Peak Table>

PDA Ch1 211nm

Peak#	Ret. Time	Area%
1	13.661	96.684
2	16.418	1.913
3	31.140	1.403
Total		100.000

5.24. (1′*R*,4*R*)- and (1′*S*,4*R*)-2-(4-Hydroxy-3-methyl-5-oxo-1-phenyl-4,5-dihydro-1*H*-pyrazol-4-yl)-2-phenylacetate **39**

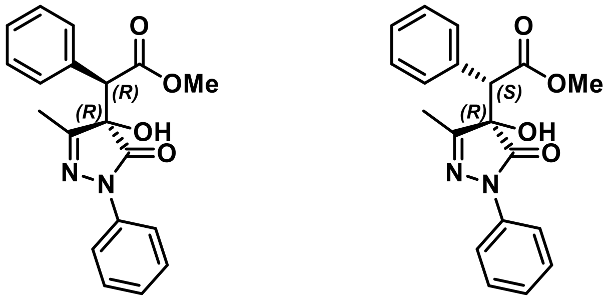

To a solution of 3-methyl-1-phenyl-1*H*-pyrazole-4,5-dione (47.1 mg, 0.25 mmol), 2-phenylacetic anhydride (95.4 mg, 0.375 mmol) and (2*R*,3*S*)-HyperBTM (3.9 mg, 0.125 mmol) were dissolved in ethyl acetate (6 ml, 0.04 M) at 0 °C. Diisopropylethylamine (54 µl, 0.31 mmol) was added and the reaction mixture was stirred at 0 °C for 3 h. DMAP (6.1 mg, 0.05 mmol) and methanol (4.0 ml) were added and the reaction mixture was stirred for 16 h at rt. The reaction mixture was diluted with ethyl acetate and washed sequentially with 1 M aq. HCl (×2), sat. aq. $NaHCO_3$ (×2), and brine (×1). The organic layer was dried over $MgSO_4$, filtered, and concentrated *in vacuo*. Purification by column chromatography (*n*-hexane : ethyl acetate 4:1 to 1:1) gave the product as a single diastereomer (55.0 mg, 65%) as an off-white semi-solid. $[\alpha]_D^{20}$ +225.9 (*c* 0.39, $CHCl_3$); **HPLC Analysis:** Chiralpak AD-H H (85:15 hexane:isopropanol, flow rate 2.00 ml·min^{-1}, 211 nm, 30 °C) t_R (2′*R*,4*R*)-**39**: 5.1 min, t_R (2′*S*,4*S*)-**39**: 10.5 min, 98:2 er; **IR** ν_{max} (film) 3377 (O-H), 2953 (C-H), 2361, 2342, 1738, 1717 (C=O, pyrazolone), 1699, 1597, 1501, 1360, 1202, 1167, 908, 754; **^{1}H NMR** (500 MHz, $CDCl_3$) δ_H: 1.88 (3H, s, C(3)C*H*$_3$), 3.78 (3H, s, OC*H*$_3$), 4.01 (1H, s, C(2′)*H*), 4.98 (1H, s, O*H*), 7.17 (1H, t, J_{HH} 7.4, NArC(4)*H*), 7.30-7.43 (7H, m, C(2′)HArC(2,3,4,5,6)*H* + NArC(3,5)*H*), 7.69 (2H, d, J_{HH} 8.1, NArC(2,6)*H*); **^{13}C{^{1}H} NMR** (126 MHz, $CDCl_3$) δ_C: 15.0 (C(3)*C*H$_3$), 53.0 (O*C*H$_3$), 54.9 (*C*(2′)H), 80.4 (*C*(4)-OH), 119.1 (NAr*C*(2,6)H), 125.6 (NAr*C*(4)H), 128.9 (C(2′)HAr*C*(3,5)H), 128.9 (NAr*C*(3,5)H), 129.1 (C(2′)HAr*C*(4)H), 129.6 (C(2′)HAr*C*(2,6)H), 131.3 (C(2′)HAr*C*(1)), 137.4 (NAr*C*(1)), 159.7 (*C*(3)=N), 171.6 (*C*(5)=O), 172.2 (*C*(1′)=O); **HRMS** (ESI$^+$) $C_{19}H_{19}N_2O_4$ $[M+H]^+$ found 339.1332, requires 339.1339 (–2.2 ppm).

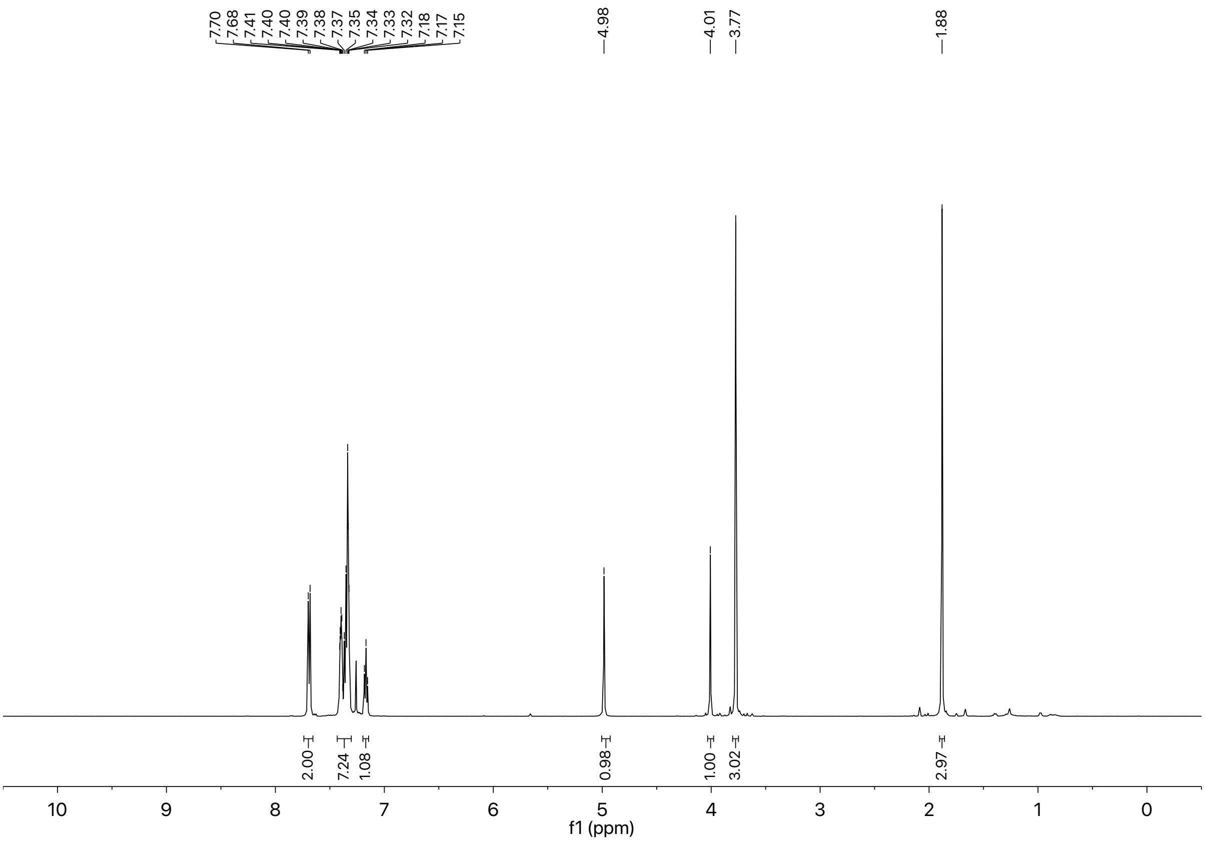
7.70
7.68
7.41
7.40
7.40
7.39
7.38
7.37
7.35
7.34
7.33
7.32
7.18
7.17
7.15
4.98
4.01
3.77
1.88
2.00
7.24
1.08
0.98
1.00
3.02
2.97
10
9
8
7
6
5
4
3
2
1
0
f1 (ppm)

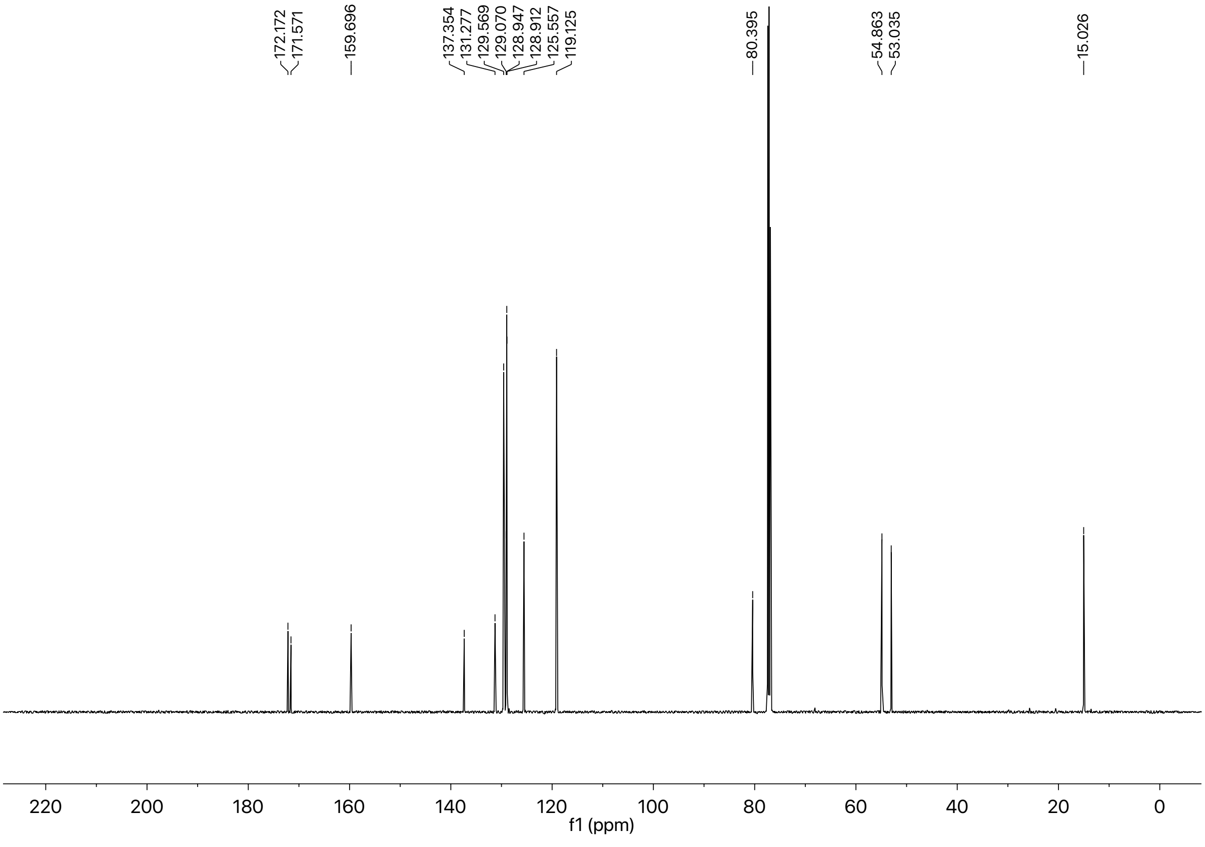
172.172
171.571
159.696
137.354
131.277
129.569
129.070
128.947
128.912
125.557
119.125
80.395
54.863
53.035
15.026
220
200
180
160
140
120
100
80
60
40
20
0
f1 (ppm)

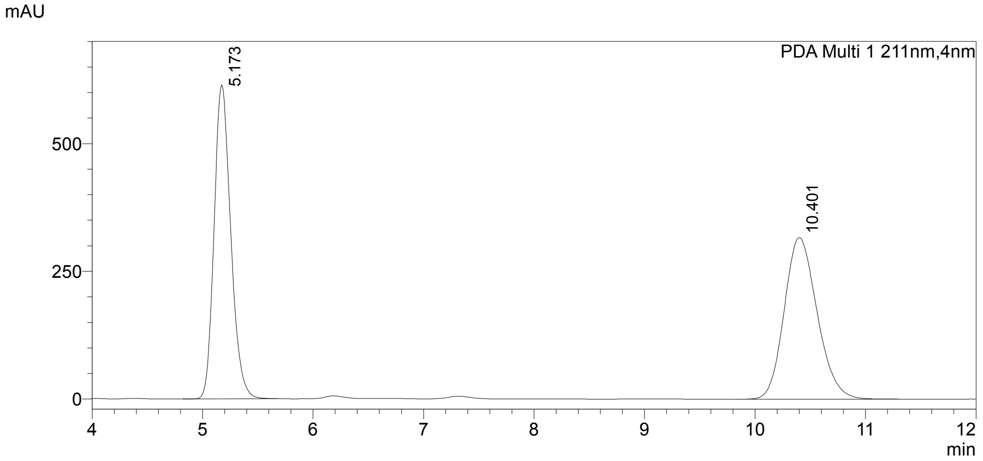

<Peak Table>

PDA Ch1 211nm

Peak#	Ret. Time	Area%
1	5.173	49.633
2	10.401	50.367
Total		100.000

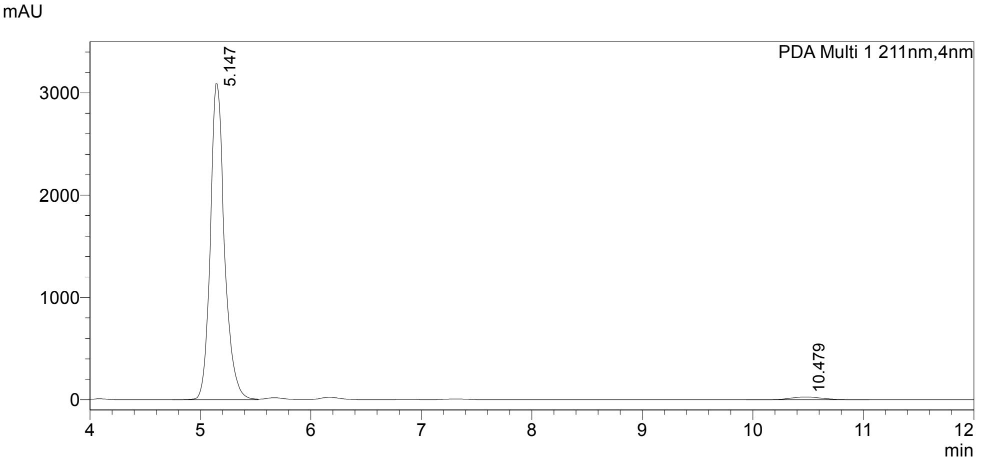

<Peak Table>

PDA Ch1 211nm

Peak#	Ret. Time	Area%
1	5.147	98.032
2	10.479	1.968
Total		100.000

5.25. *iso*-Propyl (2*R*)-2-((4*R*)-4-Hydroxy-3-methyl-5-oxy-1-phenyl-4,5-dihydro-1*H*-pyrazol-4-yl)-2-phenylacetate **40**

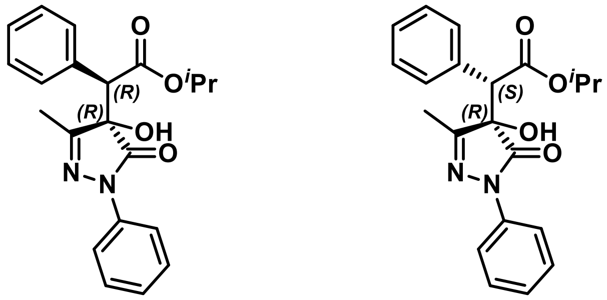

With 20 mol% DMAP: To a Schlenk tube was added 3-methyl-1-phenylpyrazol-4,5-dione (47.1 mg, 0.25 mmol), phenylacetic anhydride (95.5 mg, 0.375 mmol) and (2*R*,3*S*)-HyperBTM (3.9 mg, 1.25 µmol). EtOAc (6.0 ml, 0.04 M) was added at 0 °C followed by ^{i}Pr$_2$NEt (54 µl, 0.313 mmol). The reaction was stirred at 0 °C for 3 h. ^{i}PrOH (7.5 ml) and DMAP (6.1 mg, 50.0 µmol) were added and the mixture was left to be stirred at room temperature for 16 h. The solution was diluted with EtOAc and washed with 1 M aq. HCl twice, aq. sat. $NaHCO_3$ twice and brine. The organic layer was dried over $MgSO_4$, filtered, and concentrated under reduced pressure. The residue (94:6 d.r.) was further purified by flash column chromatography (Hexanes:EtOAc 47.5:2.5 → 45:5 → 40:10 → 35:15 → 25:25) to give the title compound as sole diastereomer as an amorphous brown solid (37.2 mg, 0.102 mmol, 41%).

With 3 mol% DMAP: To a Schlenk tube was added 3-methyl-1-phenylpyrazol-4,5-dione (47.1 mg, 0.25 mmol), phenylacetic anhydride (95.5 mg, 0.375 mmol) and (2*R*,3*S*)-HyperBTM (3.9 mg, 1.25 µmol). EtOAc (6.0 ml, 0.04 M) was added at 0 °C followed by ^{i}Pr$_2$NEt (54 µl, 0.313 mmol). The reaction was stirred at 0 °C for 3 h. ^{i}PrOH (7.5 ml) and DMAP (1.0 mg, 8.0 µmol) were added and the mixture was left to be stirred at room temperature for 16 h. The solution was diluted with EtOAc and washed with 1 M aq. HCl twice, aq. sat. $NaHCO_3$ twice and brine. The organic layer was dried over $MgSO_4$, filtered, and concentrated under reduced pressure. The residue (98:2 d.r.) was further purified by flash column chromatography (Hexanes:EtOAc 47.5:2.5 → 45:5 → 40:10 → 35:15 → 25:25) to give the title compound as sole diastereomer as an amorphous brown solid (37.5 mg, 0.102 mmol, 41%).

With 0.5 mol% DMAP: To a Schlenk tube was added 3-methyl-1-phenylpyrazol-4,5-dione (47.1 mg, 0.25 mmol), phenylacetic anhydride (95.5 mg, 0.375 mmol) and (2*R*,3*S*)-HyperBTM (3.9 mg, 1.25 µmol). EtOAc (6.0 ml, 0.04 M) was added at 0 °C followed by ^{i}Pr$_2$NEt (54 µl, 0.313 mmol). The reaction was stirred at 0 °C for 3 h. ^{i}PrOH (7.5 ml) and DMAP (0.1 M stock in EtOAc, 12 µl, 1.3 µmol) were added and the mixture was left to be stirred at room temperature for 16 h. The solution was diluted with EtOAc and washed with 1 M aq. HCl twice, aq. sat.

$NaHCO_3$ twice and brine. The organic layer was dried over $MgSO_4$, filtered, and concentrated under reduced pressure. The residue (93:7 d.r.) was further purified by flash column chromatography (Hexanes:EtOAc 47.5:2.5 → 45:5 → 40:10 → 35:15 → 25:25) to give the title compound as sole diastereomer as an amorphous brown solid (44.1 mg, 0.120 mmol, 48%).

α_D^{20}= +1.01 (*c* 1.8 in $CHCl_3$) @ 60% ee; **HPLC Analysis:** CHIRALPAK® AD-H (5% ^{i}PrOH in hexanes, flow rate 2 ml·min^{-1}, 254 nm, 30 °C) t_R (2*R*,4*R*)-**40**: 7.4 min, t_R (2*S*,4*S*)-**40**: 12.8min, 80:20 e.r. (20 mol% DMAP), 88:12 e.r. (3 mol% DMAP), 97:3 e.r. (0.5 mol% DMAP); **IR** ν_{max} (film) 3377 (br), 3063 (w), 3034 (w), 2982 (m), 2934 (w), 1721 (s), 1697 (s), 1597 (s), 1501 (s), 1456 (m), 1364 (s), 1315 (m), 1196 (s), 1173 (s), 1128 (w), 1101 (s), 1032 (w), 1005 (w), 986 (m), 905 (s), 833 (m), 750 (s); **^{1}H NMR** (400 MHz, $CDCl_3$) δ_H 1.16 (3H, d, $^3J_{HH}$ = 6.3 Hz, CH(C*H*$_3$)$_a$(CH$_3$)$_b$), 1.30 (3H, d, $^3J_{HH}$ = 6.3 Hz, CH(CH$_3$)$_a$(C*H*$_3$)$_b$), 1.89 (3H, s, N=C-C*H*$_3$), 3.95 (1H, s, C*H*-Ph), 4.95 – 5.00 (1H, m, O*H*), 5.13 (1H, app hept, $^3J_{HH}$ = 6.3 Hz, C*H*(CH$_3$)$_2$), 7.13 – 7.20 (1H, m, N-PhC^4*H*), 7.29 – 7.43 (7H, m, N-PhC^{3,5}*H*, CH-PhC^{2,3,4,5,6}*H*), 7.68 – 7.74 (2H, m, N-PhC^{2,6}*H*); **^{13}C{^{1}H} NMR** (100 MHz, $CDCl_3$) δ_C 14.9 (N=C-*C*H$_3$), 21.5 (CH(*C*H$_3$)$_a$(CH$_3$)$_b$), 21.8 (CH(CH$_3$)$_a$(*C*H$_3$)$_b$), 55.2 (*C*H-Ph), 70.2 (*C*H(CH$_3$)$_2$), 80.3 (*C*-OH), 119.1 (N-Ph*C*^{2,6}H), 125.4 (N-PhC^{4}H), 128.8 (CH-Ph*C*^{3,5}H), 128.8$_9$ (CH-Ph*C*^{4}H), 128.9$_2$(N-Ph*C*^{3,5}H), 129.6 (CH-Ph*C*^{2,6}H), 131.5 (CH-Ph*C*^{1}), 137.5 (N-Ph*C*^{1}), 159.7 (*C*=N), 171.1 (*C*(O)O^{i}Pr), 171.6 (*C*(O)NPh); ***m/z*** (ESI$^+$) 389 ([M+Na]$^+$ 55%), 405 ([M+K]$^+$ 15%), 755 ([2M+Na]$^+$ 100%), 756 ([2M(^{13}C)+Na]$^+$ 49%), 757 ([2M($^{13}C_2$)+Na]$^+$ 15%), 771 ([2M+K]$^+$ 15%); **HRMS** (ESI$^+$) $C_{21}H_{22}N_2O_4$ [M+Na]$^+$ found 389.1475, requires 389.1472 (0.7 ppm).

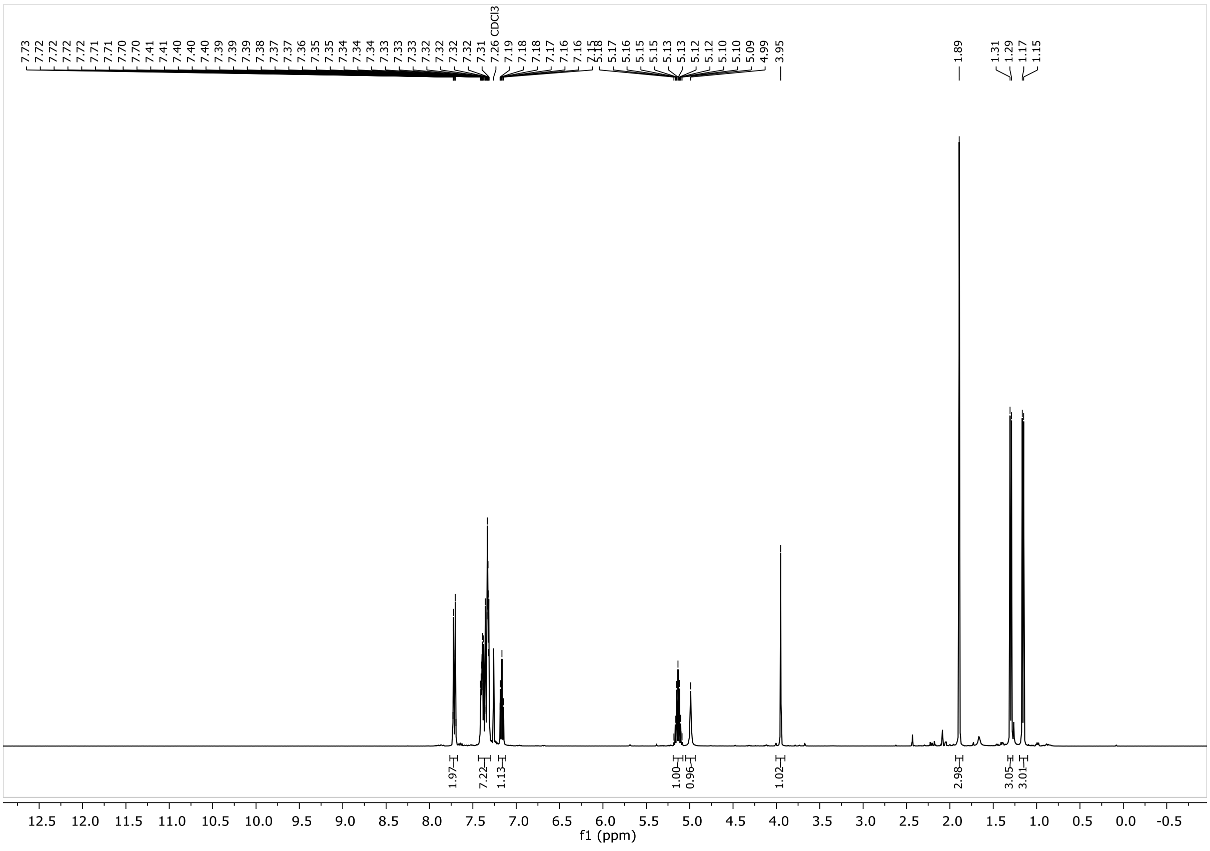
7.73 7.72 7.72 7.72 7.71 7.71 7.70 7.41 7.41 7.40 7.40 7.39 7.39 7.38 7.37 7.37 7.36 7.35 7.35 7.34 7.34 7.34 7.33 7.33 7.33 7.32 7.32 7.32 7.32 7.31 7.26 CDCl3 7.19 7.18 7.18 7.18 7.17 7.16 7.16 7.15 5.18 5.17 5.16 5.15 5.15 5.13 5.13 5.12 5.12 5.10 5.09 4.99 3.95 1.89 1.31 1.29 1.17 1.15
1.97 7.22 1.13 1.00 0.96 1.02 2.98 3.05 3.01
12.5 12.0 11.5 11.0 10.5 10.0 9.5 9.0 8.5 8.0 7.5 7.0 6.5 6.0 5.5 5.0 4.5 4.0 3.5 3.0 2.5 2.0 1.5 1.0 0.5 0.0 -0.5
f1 (ppm)

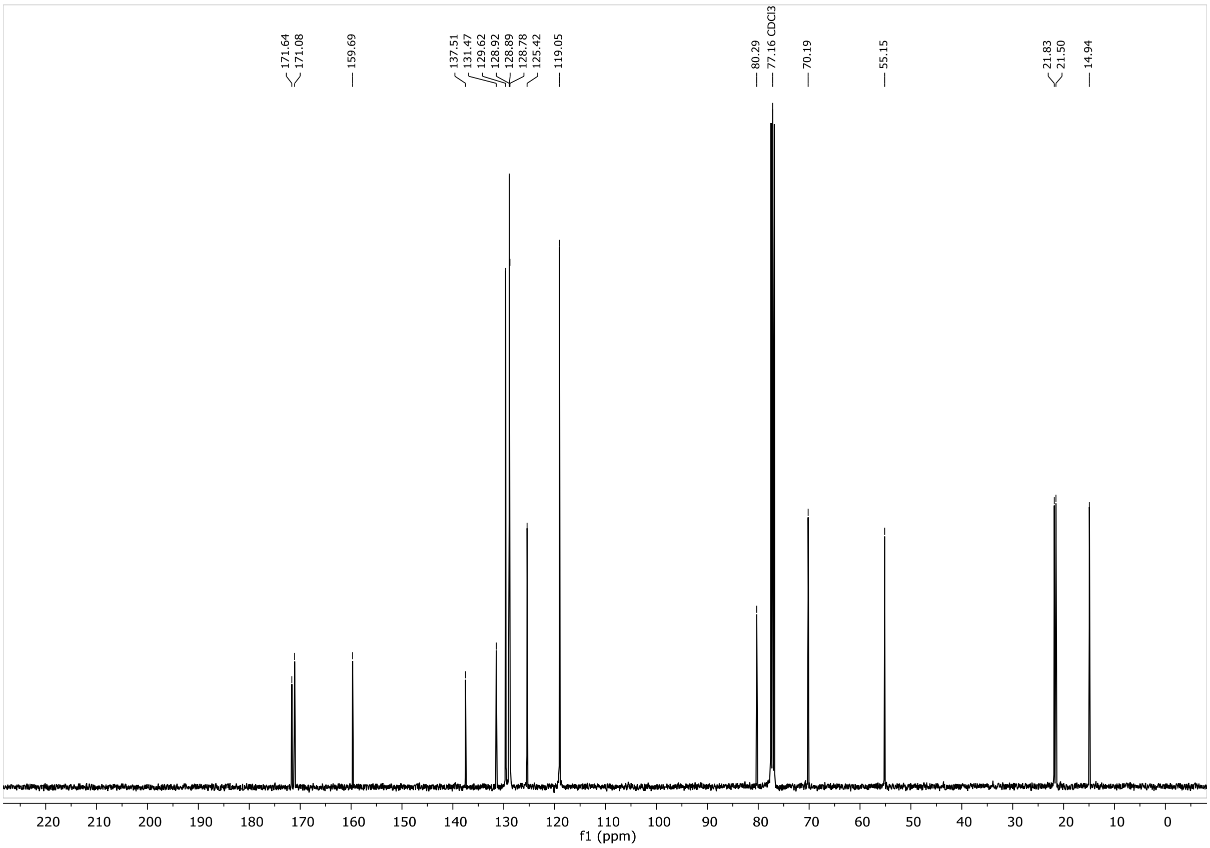
171.64 171.08 159.69 137.51 131.47 129.62 128.92 128.89 128.78 125.42 119.05 80.29 77.16 CDCl3 70.19 55.15 21.83 21.50 14.94
220 210 200 190 180 170 160 150 140 130 120 110 100 90 80 70 60 50 40 30 20 10 0
f1 (ppm)

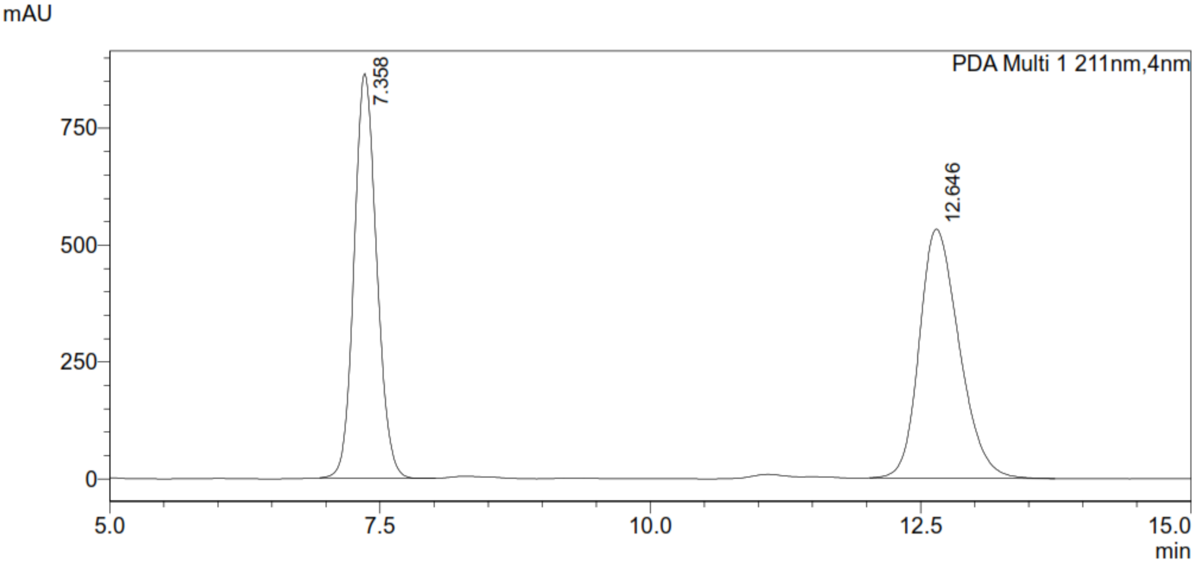

<Peak Table>

PDA Ch1 211nm

Peak#	Ret. Time	Area%
1	7.358	47.862
2	12.646	52.138
Total		100.000

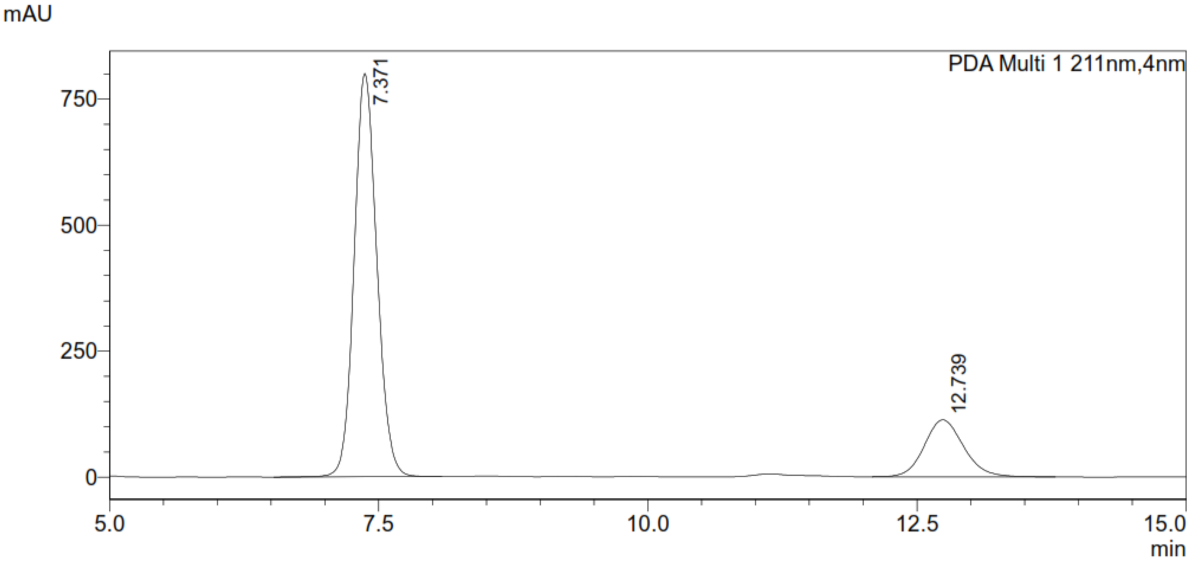

<Peak Table>

PDA Ch1 211nm

Peak#	Ret. Time	Area%
1	7.371	80.249
2	12.739	19.751
Total		100.000

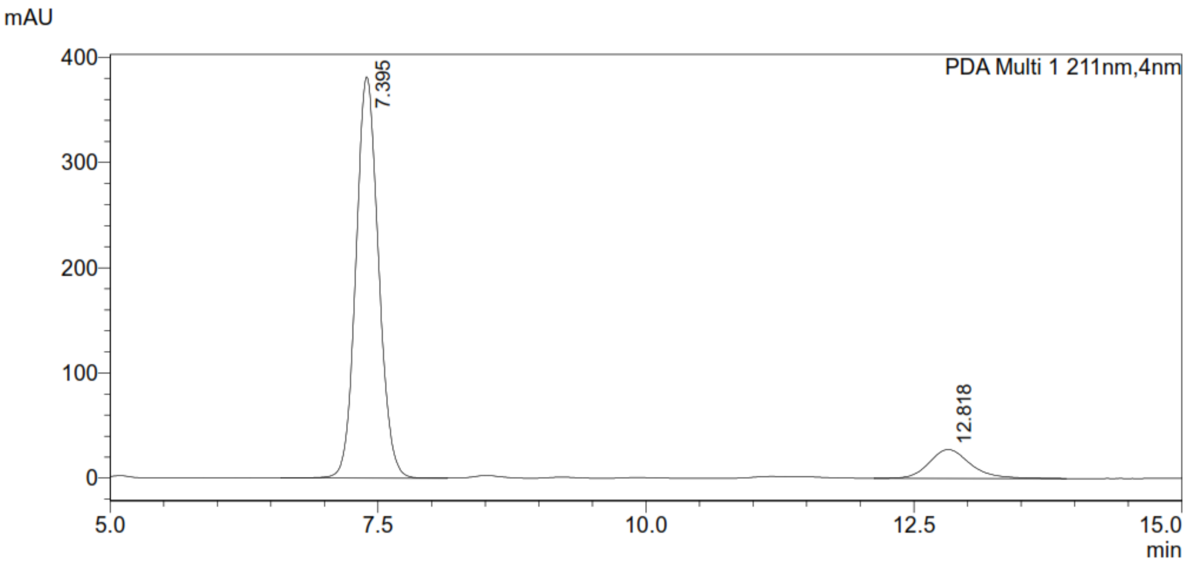

<Peak Table>

PDA Ch1 211nm

Peak#	Ret. Time	Area%
1	7.395	88.284
2	12.818	11.716
Total		100.000

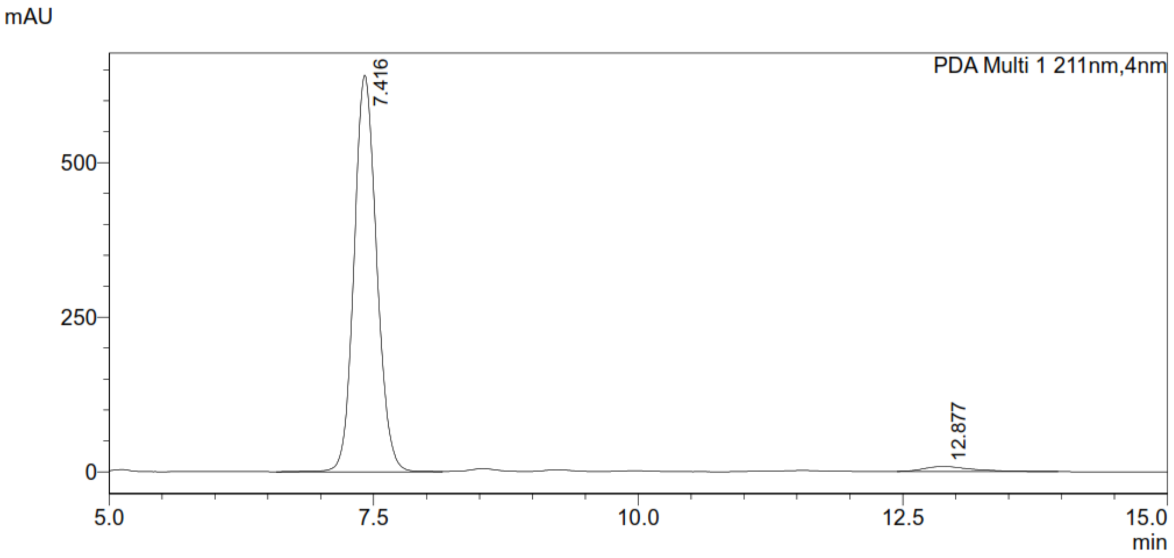

<Peak Table>

PDA Ch1 211nm

Peak#	Ret. Time	Area%
1	7.416	97.365
2	12.877	2.635
Total		100.000

5.26. (1′*R*,4*R*)- and (1′*S*,4*R*)-2-(4-Hydroxy-3-methyl-5-oxo-1-phenyl-4,5-dihydro-1*H*-pyrazol-4-yl)-2-(naphth-1-yl)-acetate **41**

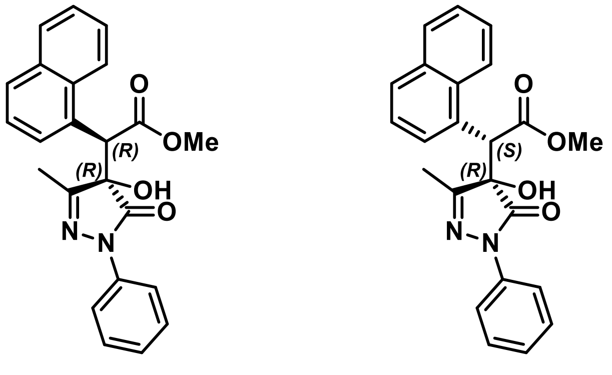

To a solution of 3-methyl-1-(naphth-1-yl)-1*H*-pyrazole-4,5-dione (47.1 mg, 0.25 mmol), 2-(naphth-1-yl)acetic anhydride (132.9 mg, 0.375 mmol) and (2*R*,3*S*)-HyperBTM (3.9 mg, 0.125 mmol) were dissolved in ethyl acetate (6 ml, 0.04 M) at 0 °C. Diisopropylethylamine (54 µl, 0.31 mmol) was added and the reaction mixture was stirred at 0 °C for 3 h. DMAP (6.1 mg, 0.05 mmol) and methanol (4.0 ml) were added and the reaction mixture was stirred for 16 h at rt. The reaction mixture was diluted with ethyl acetate and washed sequentially with 1 M aq. HCl (×2), sat. aq. $NaHCO_3$ (×2), and brine (×1). The organic layer was dried over $MgSO_4$, filtered, and concentrated *in vacuo*. Purification by column chromatography (*n*-hexane : ethyl acetate 4:1 to 1:1) gave major and minor diastereomers (55.2 mg, 57%, >95:5 dr) as an inseparable mixture as a white amorphous solid. $[\alpha]_D^{20}$ +187.0 (*c* 0.27, $CHCl_3$); **IR** ν_{max} (film) 3395 (O-H), 3061, 2953 (C-H), 1717 (C=O), 1595, 1501, 1360, 1198, 1165, 978, 783; **HRMS** (ESI^+) $C_{23}H_{21}N_2O_4$ $[M+H]^+$ found 389.1493, requires 389.14959 (–0.8 ppm). *Data for major diastereomer anti*-**41**: **HPLC Analysis:** Chiralpak AD-H (95:5 hexane:isopropanol, flow rate 2.00 ml·min^{-1}, 211 nm, 30 °C) t_R (2′*R*,4*R*)-**41**: 14.0 min, t_R (2′*S*,4*S*)-**41**: 30.6 min, 95.5:4.5 er; **^{1}H NMR** (500 MHz, $CDCl_3$) δ_H: 1.45 (3H, s, C(3)C*H*$_3$), 3.76 (3H, s, OC*H*$_3$), 4.87 (1H, s, C(2′)*H*), 5.45 (1H, br s, O*H*), 7.19 (1H, t, J_{HH} 7.4, NArC(4)*H*), 7.38 (2H, app t, J_{HH} 8.0, NAr(3,5)*H*), 7.48-7.56 (2H, m, C(2′)HArC(4)*H* + C(2′)HArC(7)*H*), 7.56-7.61 (1H, m, C(2′)HArC(3)*H*), 7.76 (2H, d, J_{HH} 7.9, NArC(2,6)*H*), 7.84-7.92 (2H, m, C(2′)HArC(6)*H* + C(2′)HArC(5)*H*), 7.96 (1H, d, J_{HH} 7.2, C(2′)HArC(8)*H*), 7.99 (1H, d, J_{HH} 8.5, C(2′)HArC(2)*H*); **^{13}C{^{1}H} NMR** (126 MHz, $CDCl_3$) δ_C: 14.7 (C(3)*C*H$_3$), 46.7 (*C*(2′)H), 53.1 (O*C*H$_3$), 80.1 (*C*(4)=O), 119.0 (NAr*C*(2,6)H), 122.3 (C(2′)HAr*C*(2)H), 125.3 (NAr*C*(4)H), 125.5 (C(2′)HAr*C*(4)H), 126.2 (C(2′)HAr*C*(7)H), 127.3 (C(2′)HAr*C*(8a)), 127.4 (C(2′)HAr*C*(3)H), 127.6 (C(2′)HAr*C*(8)H), 129.0 (NAr*C*(3,5)H), 129.4 (C(2′)HAr*C*(5)H), 129.7 (C(2′)HAr*C*(6)H), 131.9 (C(2′)HAr*C*(1)), 134.0 (C(2′)HAr*C*(4a)), 137.6 (NAr*C*(1)), 159.7 (*C*(3)=N), 172.2 (*C*(5)=O), 172.7 (*C*(1′)=O); *Data for minor diastereomer syn*-**41**: **HPLC Analysis:** Chiralpak AD-H (95:5 hexane:isopropanol, flow rate 2.00 ml·min^{-1}, 211 nm, 30 °C) t_R (2′*S*,4*R*)-**41**: 17.3

min, t_R (2′*R*,4*S*)-**41**: 23.4 min, 93.5:6.5 er; **^{1}H NMR** (500 MHz, $CDCl_3$) (*selected*) δ_H: 1.88 (3H, s, C(3)C*H*$_3$), 3.80 (3H, s, OC*H*$_3$), 5.03 (1H, s, C(2′)*H*); **^{13}C{^{1}H} NMR** (126 MHz, $CDCl_3$) (*selected*) δ_C: 13.7 (C(3)*C*H$_3$), 53.4 (O*C*H$_3$), 119.2 (NAr*C*(2,6)H).

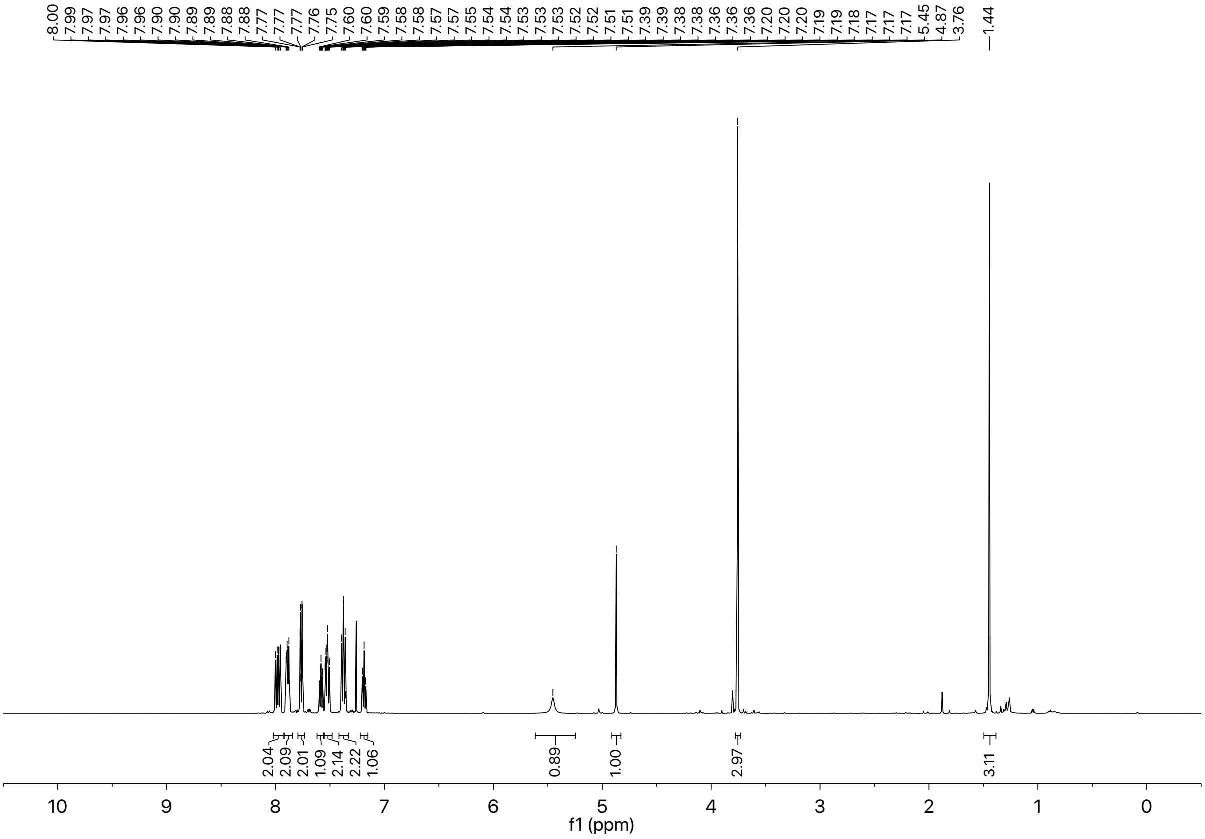

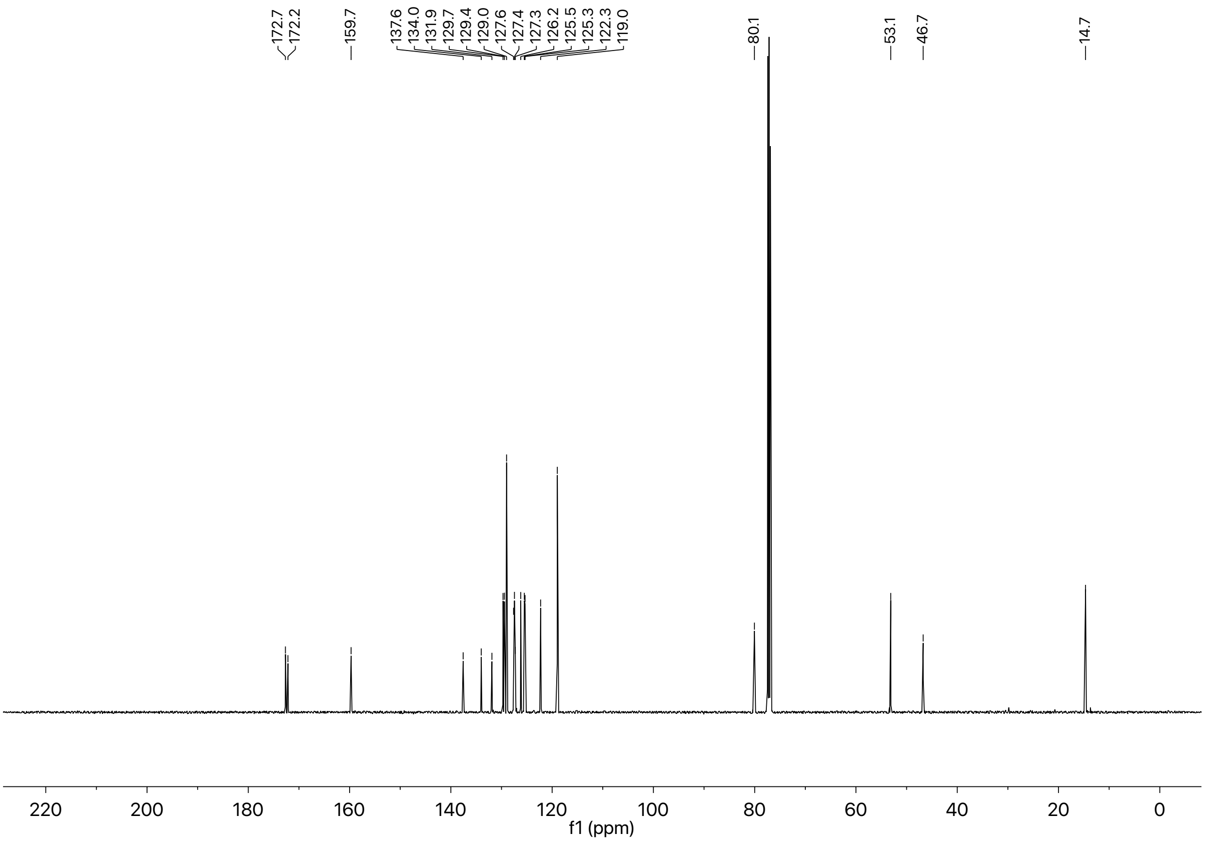

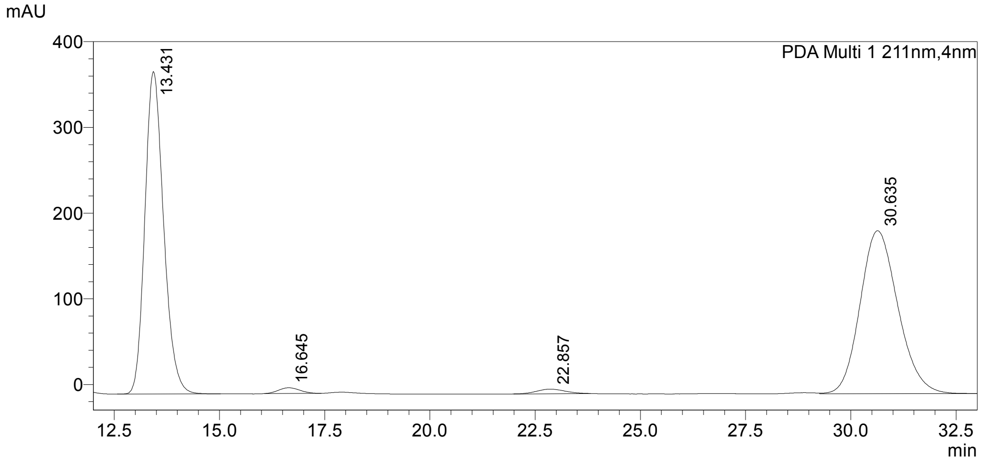

<Peak Table>

PDA Ch1 211nm

Peak#	Ret. Time	Area%
1	13.431	48.492
2	16.645	0.980
3	22.857	1.048
4	30.635	49.480
Total		100.000

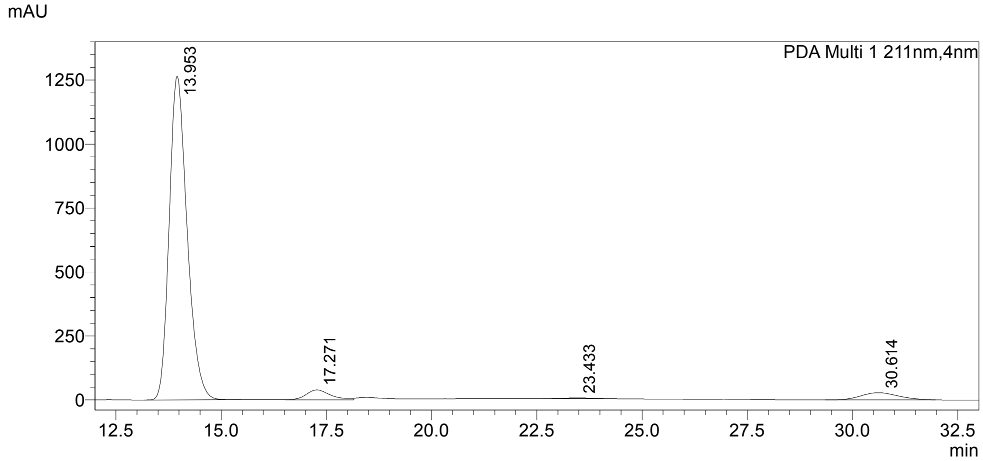

<Peak Table>

PDA Ch1 211nm

Peak#	Ret. Time	Area%
1	13.953	91.666
2	17.271	3.818
3	23.433	0.273
4	30.614	4.243
Total		100.000

5.27. Pentamethylene (2*R*)-2-((4*R*)-4-Hydroxy-3-methyl-5-oxy-1-phenyl-4,5-dihydro-1*H*-pyrazol-4-yl)-2-phenylacetamide **42**

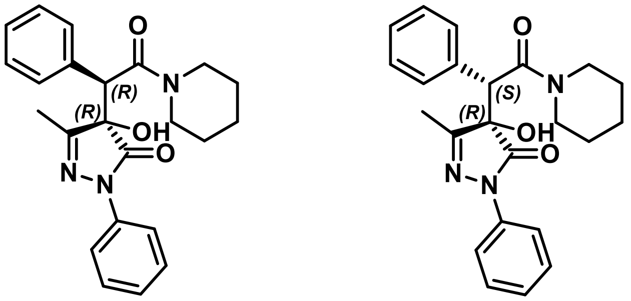

To a Schlenk tube was added 3-methyl-1-phenylpyrazol-4,5-dione (47.1 mg, 0.25 mmol), phenylacetic anhydride (95.5 mg, 0.375 mmol) and (2*R*,3*S*)-HyperBTM (3.9 mg, 1.25 µmol). EtOAc (6.0 ml, 0.04 M) was added at 0 °C followed by $^{i}Pr_2NEt$ (54 µl, 0.313 mmol). The reaction was stirred at 0 °C for 3 h. Piperidine (74 µl, 0.750 mmol) were added and the mixture was left to be stirred at room temperature for 16 h. The solution was diluted with EtOAc and washed with 1 M aq. HCl twice, aq. sat. $NaHCO_3$ twice and brine. The organic layer was dried over $MgSO_4$, filtered, and concentrated under reduced pressure. The residue (92:8 d.r.) was further purified by flash column chromatography (3/5 Enzo, Hexanes:EtOAc 47.5:2.5 → 45:5 → 40:10 → 35:15 → 25:25) to give the title compound as sole diastereomer as an amorphous brown solid (66.8 mg, 0.171 mmol, 68%). Characterisation data analysed as 90:10 mixture of diasteromers: α_D^{20}= +1.83 (*c* 3.9 in $CDCl_3$) @ 90:10 d.r., 99:1 and 98:2 e.r.; **HPLC Analysis:** CHIRALPAK® AD-H (5% ^{i}PrOH in hexanes, flow rate 1 ml·min^{-1}, 254 nm, 30 °C) t_R (2*S*,4*S*)-**42**: 15.1 min, t_R (2*R*,4*R*)-**42**: 16.9 min, 1:99 e.r. major diastereomer, t_R (2*S*,4*R*)-**42**: 24.7 min, t_R minor (2*R*,4*S*)-**42**: 16.9 min, 2:98 e.r. minor diastereomer; **IR** ν_{max} (film) 3370 (br), 3063 (w), 3030 (w), 2938 (m), 2857 (w), 1717 (s), 1614 (s), 1597 (s), 1499 (s), 1445 (s), 1362 (s), 1314 (w), 1246 (m), 1225 (m), 1190 (w), 1126 (m), 1064 (w), 1024 (m), 908 (m); **^{1}H NMR** (400 MHz, $CDCl_3$) δ_H 0.80 – 0.95 (0.1H, m, N(CH_2CH_2)$_2$CH_aH$_b$), 0.96 (0.9H, app dtt, $^2J_{HH}$ = 13.4 Hz, $^3J_{HH}$ = 7.9 Hz, 4.3 Hz, N(CH_2CH_2)$_2$CH_aH$_b$), 1.35 (1.0H, app dtt (minor obscured), $^2J_{HH}$ = 13.4 Hz, $^3J_{HH}$ = 7.0 Hz, 3.7 Hz, N(CH_2CH_2)$_2$CH$_a$$H_b$), 1.40 – 1.68 (4.0H, m, N(CH$_2$CH_2)$_2$CH$_2$), 1.91 (2.7H, s, N=C-CH_3), 2.06 (0.3H, s, N=CH_3), 3.15 (1.0H, ddd (minor obscured), $^2J_{HH}$ = 13.5 Hz, $^3J_{HH}$ = 6.8 Hz, 3.9 Hz, N(CH_aH$_b$)$_a$(CH$_2$)$_b$), 3.22 (1.0H, ddd (minor obscured), $^2J_{HH}$ = 13.5 Hz, $^3J_{HH}$ = 6.8 Hz, 3.9 Hz, N(CH$_a$$H_b$)$_a$(CH$_2$)$_b$), 3.38 (0.1H, ddd, $^2J_{HH}$ = 12.9 Hz, $^3J_{HH}$ = 8.5 Hz, 2.5 Hz, N(CH$_2$)$_a$(CH_aH$_b$)$_b$), 3.52 (0.9H, ddd, $^2J_{HH}$ = 12.9 Hz, $^3J_{HH}$ = 7.7 Hz, 3.4 Hz, N(CH$_2$)$_a$(CH_aH$_b$)$_b$), 3.71 (1.0H, ddd (minor obscured), $^2J_{HH}$ = 12.9 Hz, $^3J_{HH}$ = 7.7 Hz, 3.4 Hz, N(CH$_2$)$_a$(CH_aH$_b$)$_b$), 4.08 (0.1H, s, C*H*-Ph), 4.23 (0.9H, s, C*H*-Ph), 6.10 (0.1H, s, O*H*), 6.75 (0.9H, s, O*H*), 7.09 – 7.18 (1H, m, N-PhC^4*H*), 7.26 –

7.38 (7.0H, m, N-Ph$C^{3,5}$*H*, CH-Ph$C^{2,3,4,5,6}$*H*), 7.69 – 7.76 (2.0H, N-Ph$C^{2,6}$*H*); **^{13}C{^{1}H} NMR** (100 MHz, $CDCl_3$) δ_C 14.8 (N=C-*C*H_3, minor), 15.6 (N=C-*C*H_3, major), 24.3 (N(CH_2*C*H_2)$_a$(CH_2CH_2)$_b$), 25.4$_5$ (N(CH_2CH_2)$_a$(CH_2*C*H_2)$_b$, minor), 25.4$_9$ (N(CH_2CH_2)$_a$(CH_2*C*H_2)$_b$, major), 25.7 (N(CH_2CH_2)$_2$*C*H), 43.2 (N(CH_2CH_2)$_a$(*C*H_2CH_2)$_b$, minor), 43.3 (N(CH_2CH_2)$_a$(*C*H_2CH_2)$_b$, major), 47.2$_1$ (N(*C*H_2CH_2)$_a$(CH_2CH_2)$_b$, minor), 47.2$_4$ (N(*C*H_2CH_2)$_a$(CH_2CH_2)$_b$, major), 52.0 (*C*H-Ph, minor), 52.2 (*C*H-Ph, major), 80.7 (*C*-OH, minor), 81.9 (*C*-OH, major), 118.9 (N-Ph$C^{2,6}$H, minor), 119.1 (N-Ph$C^{2,6}$H, major), 124.9 (N-PhC^4H, minor), 125.3 (N-PhC^4H, major), 128.4 (CH-PhC_4H, minor), 128.7 (CH-PhC^4H, major), 128.8 (N-Ph$C^{3,5}$H, major), 128.9 (CH-Ph$C^{3,5}$H, major), 129.1 (CH-Ph$C^{2,6}$H, major), 129.8 (N-Ph$C^{3,5}$H, minor), 132.1 (CH-PhC^1, minor), 132.5 (CH-PhC^1, major), 137.6 (N-PhC^1, major), 137.9 (N-PhC^1, minor), 160.9 (N=*C*, minor), 161.0 (N=*C*, major), 169.1 (CH-*C*(O)O, minor), 169.7 (CH-*C*(O)O, major), 170.8 (*C*(O)N, minor), 172.0 (*C*(O)N, major), not all signals of the minor diastereomer could be resolved; ***m/z*** (ESI$^+$) 414 ([M+Na]$^+$ 100%), 415 ([M(^{13}C)+Na]$^+$ 28%), 416 ([M($^{13}C_2$)+Na]$^+$ 4%), 805 ([2M+Na]$^+$ 69%), 806 ([2M(^{13}C)+Na]$^+$ 35%), 807 (2[M($^{13}C_2$)+Na]$^+$ 10%); **HRMS** (ESI$^+$) $C_{23}H_{25}N_3O_3$ [M+Na]$^+$ found 414.1795, requires 414.1788 (1.6 ppm)

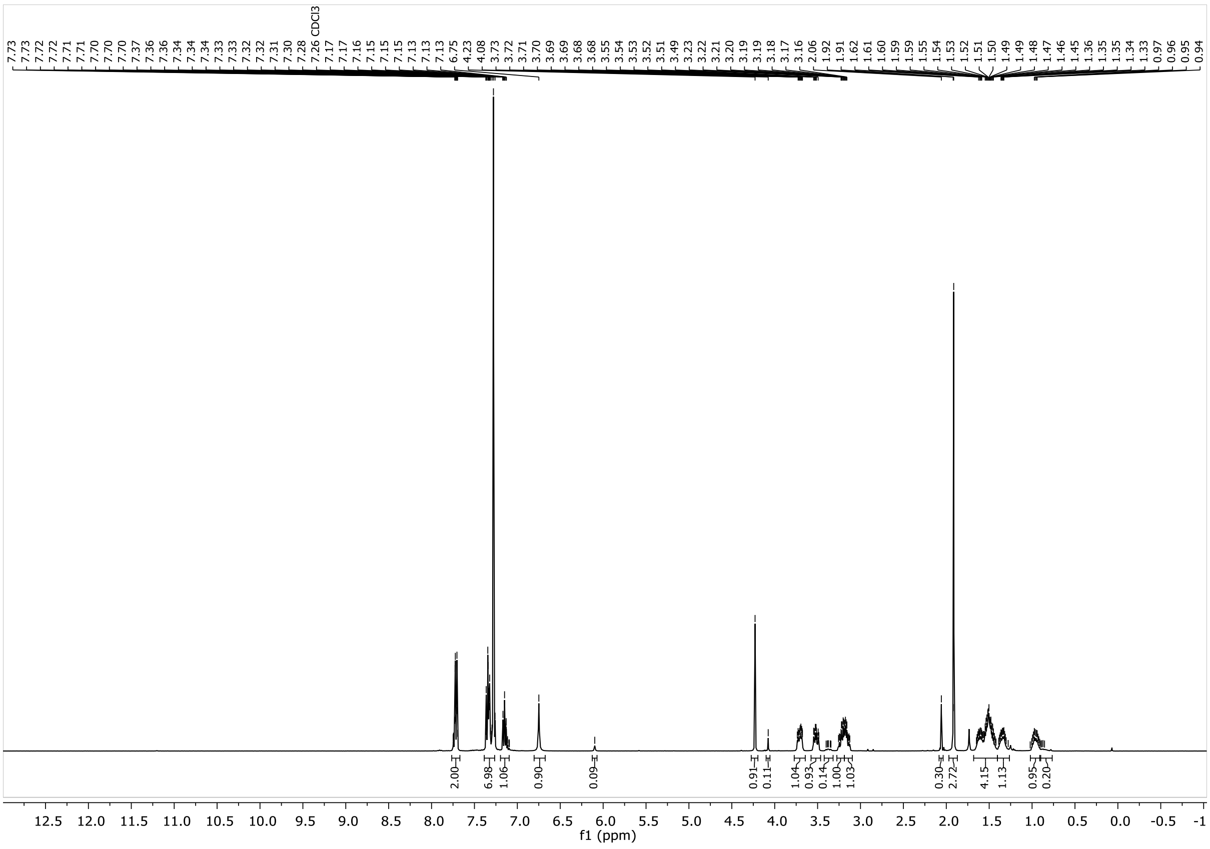

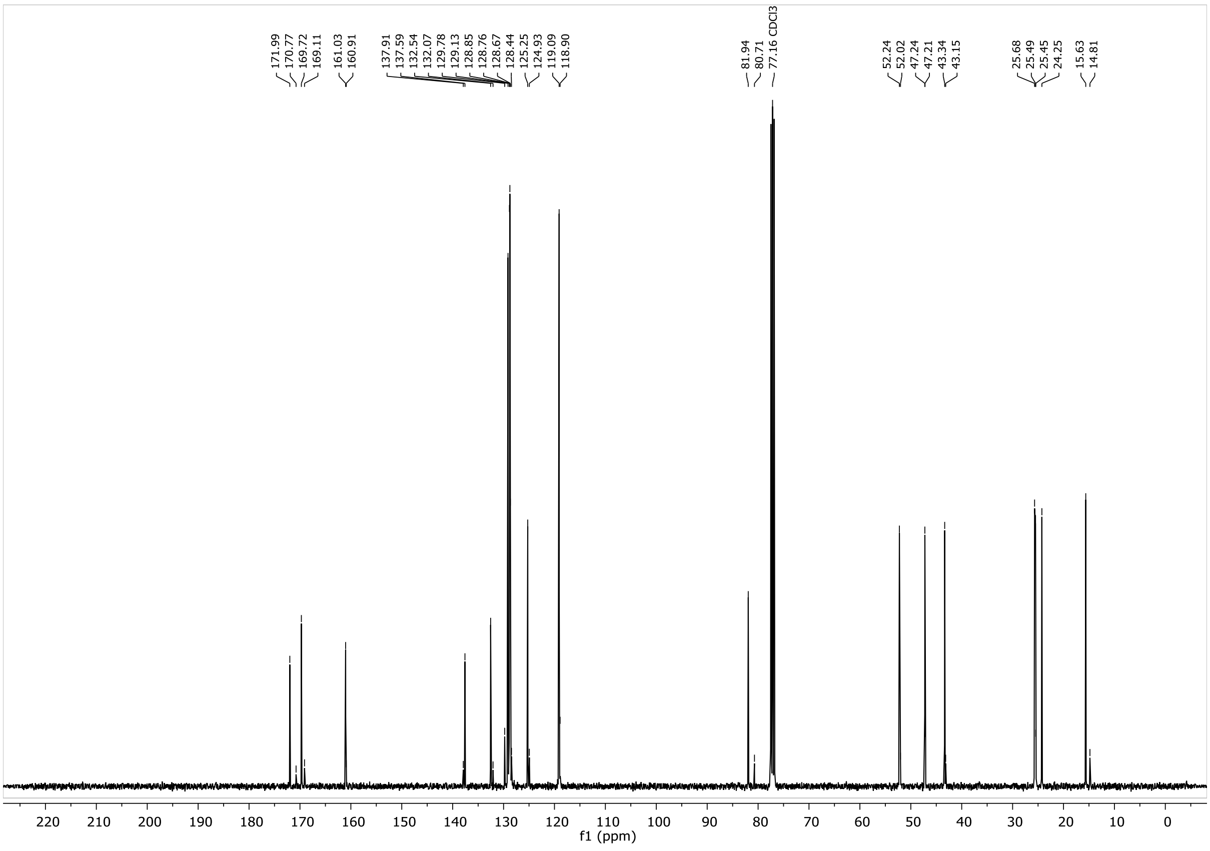

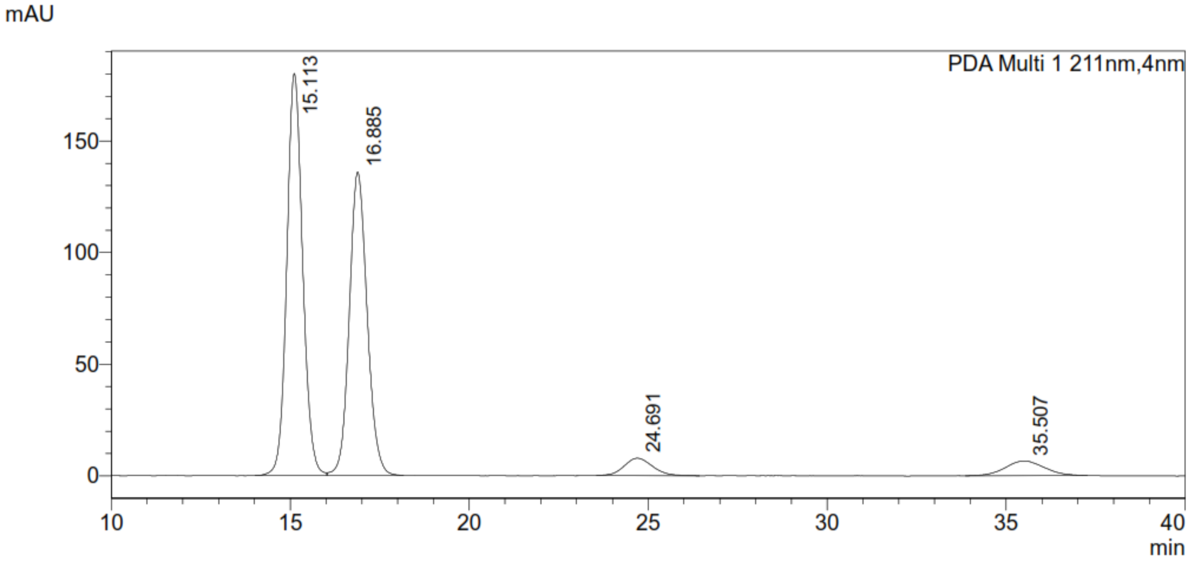

<Peak Table>

PDA Ch1 211nm

Peak#	Ret. Time	Area%
1	15.113	49.444
2	16.885	42.090
3	24.691	3.935
4	35.507	4.530
Total		100.000

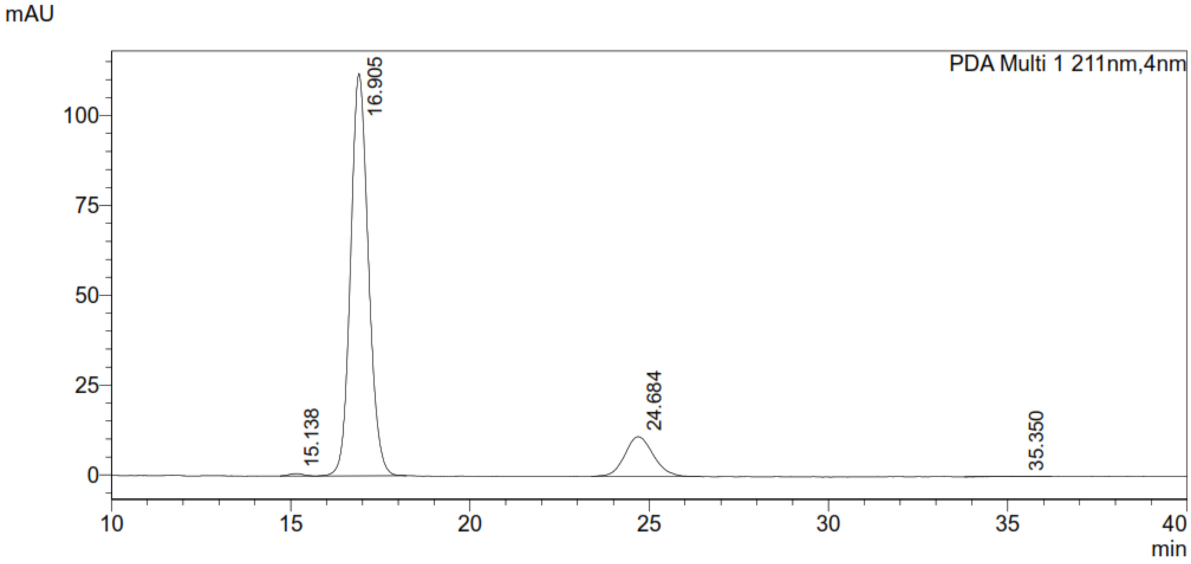

<Peak Table>

PDA Ch1 211nm

Peak#	Ret. Time	Area%
1	15.138	0.408
2	16.905	85.795
3	24.684	13.722
4	35.350	0.074
Total		100.000

5.28. (4*R*)-4-((1*R*)-2-oxo-1-phenyl-2-(4-tert-butyloxycarbonylpiperazin-1-yl)ethyl)-4-hydroxy-3-methyl-1-phenylpyrazoline-5-one **43**

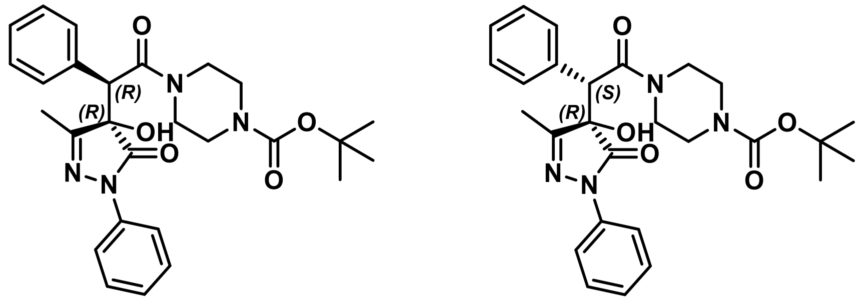

To a Schlenk tube was added 3-methyl-1-phenylpyrazol-4,5-dione (47.1 mg, 0.25 mmol), phenylacetic anhydride (95.5 mg, 0.375 mmol) and (2*R*,3*S*)-HyperBTM (3.9 mg, 1.25 µmol). EtOAc (6.0 ml, 0.04 M) was added at 0 °C followed by $^{i}Pr_2NEt$ (54 µl, 0.313 mmol). The reaction was stirred at 0 °C for 3 h. *N*-*tert*-Butyloxycarbonylpiperazine (139.8 mg, 0.750 mmol) were added and the mixture was left to be stirred at room temperature for 16 h. The precipitate (*N*-*tert*-butyloxycarbonyl-*N'*-phenacylpiperazine) was filtered off, the filtrate was diluted with EtOAc and washed with 1 M aq. HCl twice, aq. sat. $NaHCO_3$ twice and brine. The organic layer was dried over $MgSO_4$, filtered, and concentrated under reduced pressure. The residue (93:7 d.r.) was further purified by flash column chromatography (3/5 Enzo, Hexanes:EtOAc 40:10 → 30:20 → 20:30) to give the title compound as a 90:10 mixture of diastereomers as an amorphous brown solid (81.7 mg, 0.166 mmol, 66%). Characterisation data analysed as 90:10 mixture of diastereomers: α_D^{20}= +1.17 (*c* 4.9 in $CDCl_3$) @ 90:10 d.r. and 99:1 e.r.; **HPLC analysis:** CHIRALPAK® AD-H (5% ^{i}PrOH in hexanes, flow rate 1 ml·min^{-1}, 254 nm, 30 °C) t_R (1*R*,4*R*)-**43**: 23.6 min, t_R (1*S*,4*S*)-**43**: 36.4 min, >99:1 e.r. major diastereomer; t_R (1*S*,4*R*)-**43**: 43.9 min, t_R (1*R*,4*S*)-**43**: 48.5 min, >99:1 e.r. minor diastereomer; **IR** ν_{max} (film) 3353 (br), 3065 (w), 30032 (w), ,3005 (w), 2978 (m), 2926 (m), 2864 (w), 1717 (m), 1695 (s), 1624 (s), 1597 (m), 1501 (m), 1458 (m), 1418 (s), 1395 (m), 1364 (s), 1285 (m), 1250 (s), 1225 (s), 1163 (s), 1125 (s), 1092 (w), 1028 (m), 995 (m), 908 (s), 862 (m), 750 (s); **^{1}H NMR** (400 MHz, $CDCl_3$) δ_H 1.41 (9H, s, C(CH_3)$_3$), 1.92 (2.7H, s, N=CCH_3), 1.97 (0.3H, s, N=CCH_3), 2.59 – 2.70 (0.1H, m, N(CH$_2$CH_aH$_b$)NBoc), 2.78 (0.9H, ddd, $^2J_{HH}$ = 13.4 Hz, $^3J_{HH}$ = 7.4 Hz, 3.3 Hz, N(CH$_2$CH_aH$_b$)$_a$(CH$_2$CH$_2$)$_b$NBoc), 3.08 – 3.22 (0.9H, m, N(CH_aH$_b$CH$_2$)$_a$(CH$_2$CH$_2$)$_b$NBoc), 3.22 – 3.35 (2.7H, m, N(CH$_a$$H_bCH_a$$H_b$)$_a$(CH$_2CH_aH_b$)$_b$NBoc), 3.47 – 3.64 (1.8H, m, N(CH$_2$CH$_2$)$_a$(CH_aH$_b$CH$_a$$H_b$)$_b$NBoc), 3.75 (0.9H, ddd, $^2J_{HH}$ = 14.0 Hz, $^3J_{HH}$ = 6.6 Hz, 3.4 Hz, N(CH$_2$CH$_2$)$_a$(CH$_a$$H_bCH_2$)$_b$NBoc), 4.22 (1H, s, C*H*-Ph), 5.28 (0.1H, s, O*H*), 6.41 (0.9H, s(br), O*H*), 7.12 (0.1H, app t, $^3J_{HH}$ = 7.5 Hz, N-PhC^4*H*), 7.16 (0.9H, app t, $^3J_{HH}$ = 7.4 Hz, N-PhC^4*H*), 7.23 – 7.34 (5H, m, CH-PhC^{2,3,4,5,6}*H*), 7.35 (1.8H, dd, $^3J_{HH}$ = 8.1 Hz, 7.4 Hz, N-PhC^{3,5}*H*), 7.70 (1.8H, app d, $^3J_{HH}$

= 8.1 Hz, N-Ph$C^{2,6}$*H*), 7.72 (0.2H, app d, $^{3}J_{HH}$ = 8.1 Hz, N-Ph$C^{2,6}$*H*), not all signals of the minor diastereomer could be resolved; **^{13}C{^{1}H} NMR** (100 MHz, $CDCl_3$) δ_C 14.8 (minor, =CCH_3), 15.6 (major,=CCH_3), 28.4 (major, $(CH_3)_3$), 42.0 (major, N$(CH_2CH_2)_a$$(CH_2CH_2)_b$NBoc), 42.7 (broad, major, N$(CH_2CH_2)_a$$(CH_2CH_2)_b$NBoc), 43.4 (broad, major, N$(CH_2CH_2)_a$$(CH_2CH_2)_b$NBoc), 46.0 (major, N$(CH_2CH_2)_a$$(CH_2CH_2)_b$NBoc), 52.6 (broad, major, *C*H-Ph), 80.6 (major, *C*$(CH_3)_3$), 81.7 (*C*-OH), 119.0 (minor, N-Ph$C^{2,6}$H), 119.1 (major, N-Ph$C^{2,6}$H), 125.1 (minor, N-PhC^{4}H), 125.4 (major, N-PhC^{4}H), 128.8 (major, CH-Ph$C^{2,6}$H), 128.9 (major, N-Ph$C^{3,5}$H), 129.0 (major, CH-PhC^{4}H), 129.4 (major, CH-Ph$C^{3,5}$H), 129.8 (minor, Ph*C*H), 132.1 (major, CH-PhC^{1}), 137.5 (major, N-PhC^{1}), 154.4 (N*C*(=O)O^{t}Bu), 160.7 (N=*C*CH_3), 170.3 (Ph-CH*C*(=O)N), 171.8 (Ph-N*C*(=O)); ***m/z*** (ESI$^+$) 414 ([M-Boc+Na]$^+$ 11%), 515 ([M+Na]$^+$ 100%), 516 ([M(^{13}C)+Na]$^+$ 33%), 517 ([M($^{13}C_2$)+Na]$^+$ 6%), 805 (7%); **HRMS** (ESI$^+$) $C_{27}H_{32}N_4NO_5Na$ [M+Na]$^+$ found 515.2271, requires 515.2265 (1.1 ppm).

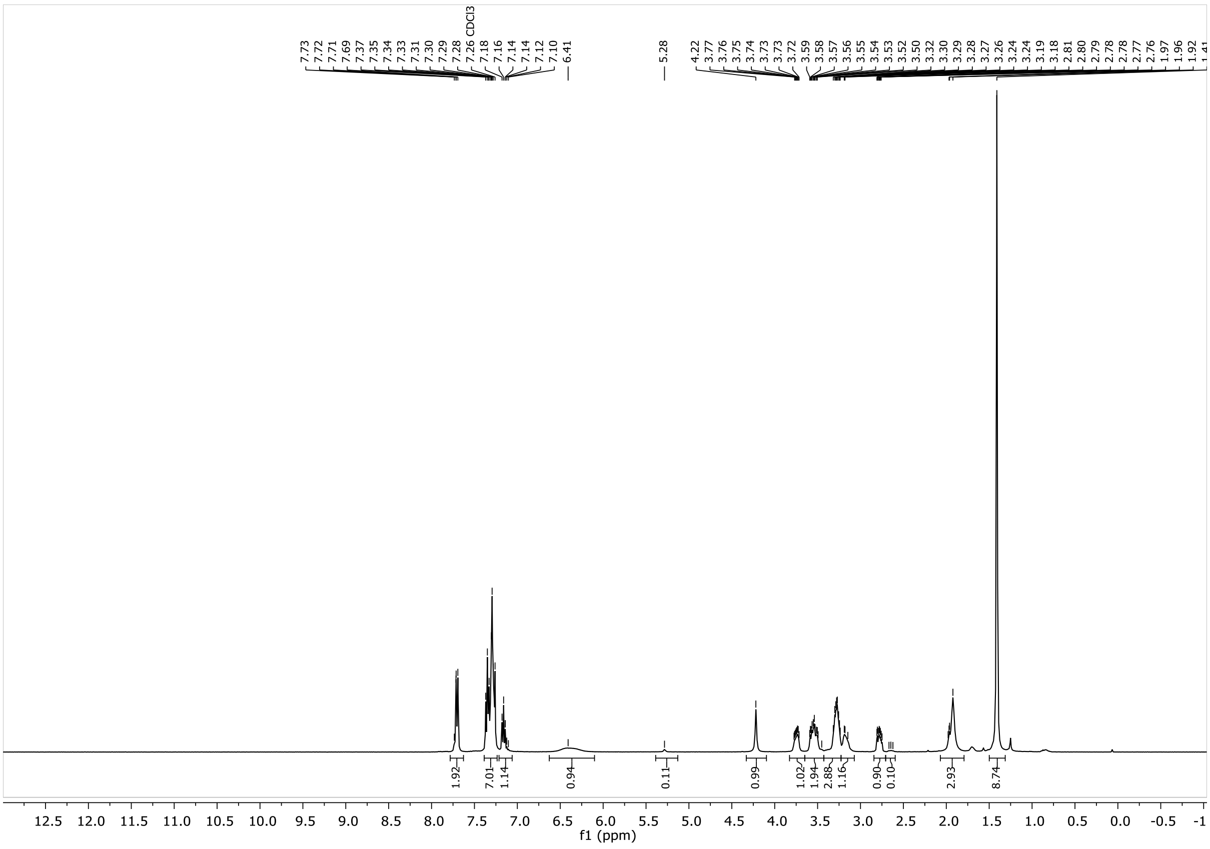
7.73
7.72
7.71
7.69
7.37
7.35
7.34
7.33
7.31
7.30
7.29
7.28
7.26 CDCl3
7.18
7.16
7.14
7.14
7.12
7.10
6.41
5.28
4.22
3.77
3.76
3.75
3.74
3.73
3.73
3.72
3.59
3.58
3.57
3.56
3.55
3.54
3.53
3.52
3.50
3.32
3.30
3.29
3.28
3.27
3.26
3.24
3.24
3.19
3.18
2.81
2.80
2.79
2.78
2.77
2.76
1.97
1.96
1.92
1.92
1.92
7.01
1.14
0.94
0.11
0.99
1.02
1.94
2.88
1.16
0.90
0.10
2.93
8.74
f1 (ppm)

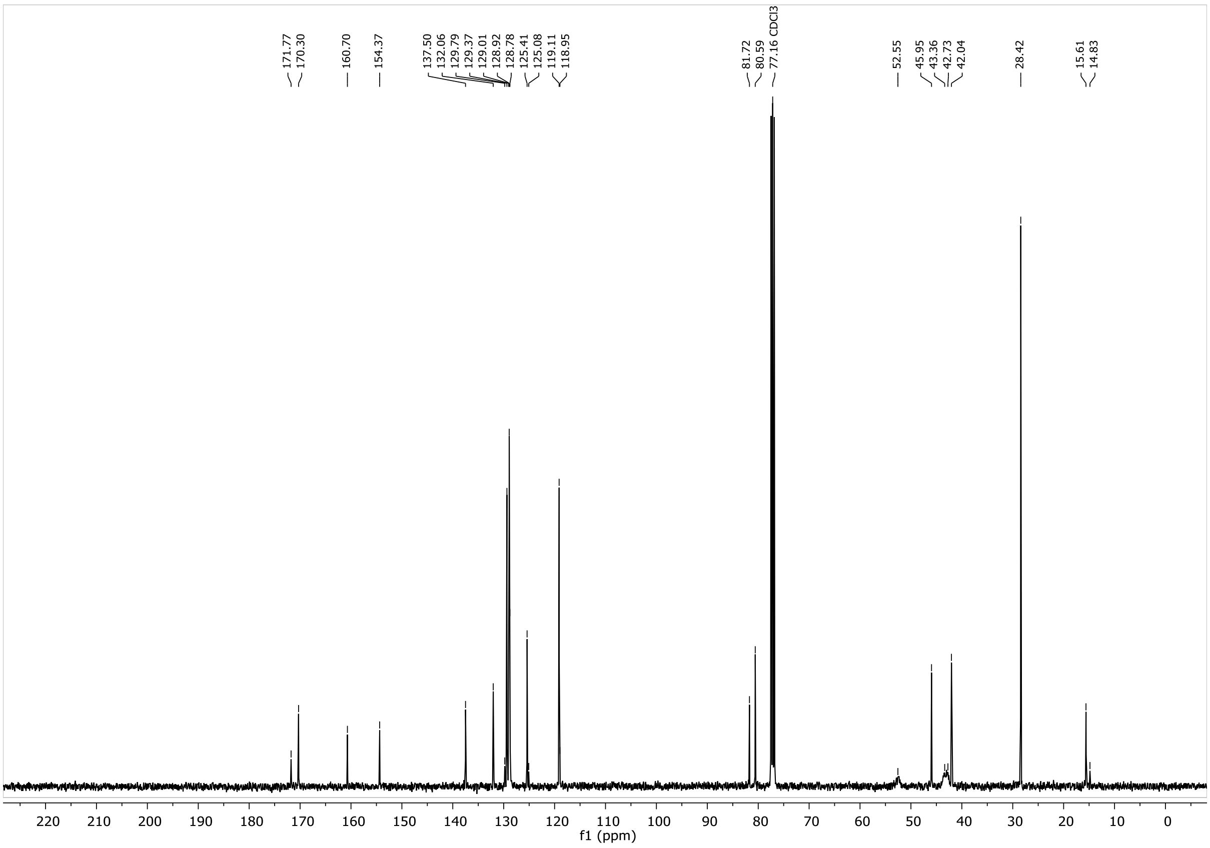
171.77
170.30
160.70
154.37
137.50
132.06
129.79
129.37
129.01
128.92
128.78
125.41
125.08
119.11
118.95
81.72
80.59
77.16 CDCl3
52.55
45.95
43.36
42.73
42.04
28.42
15.61
14.83
f1 (ppm)

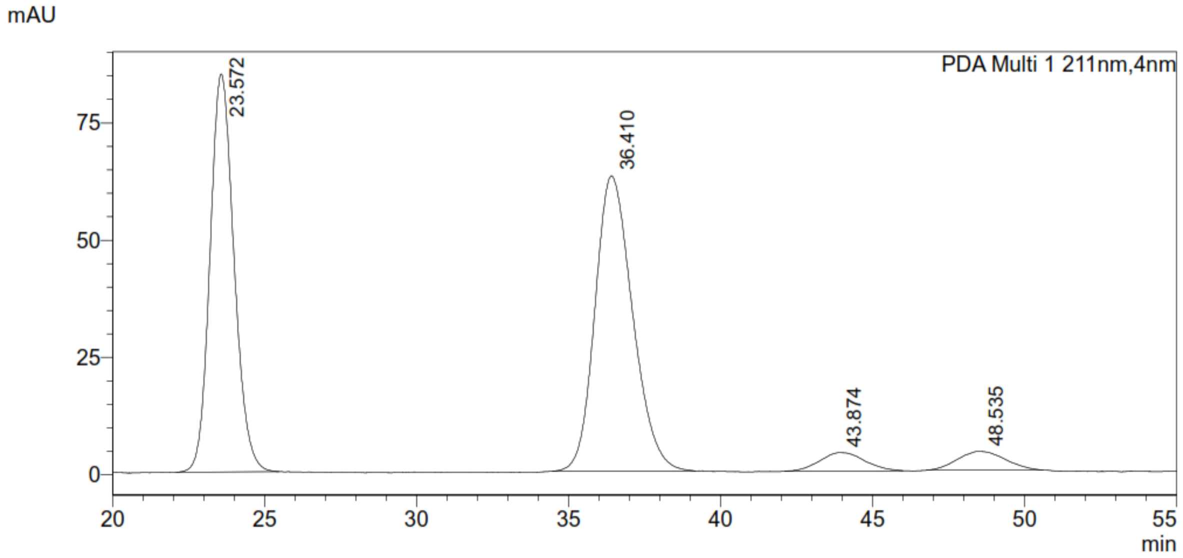

<Peak Table>

PDA Ch1 211nm

Peak#	Ret. Time	Area%
1	23.572	42.719
2	36.410	49.588
3	43.874	3.694
4	48.535	3.998
Total		100.000

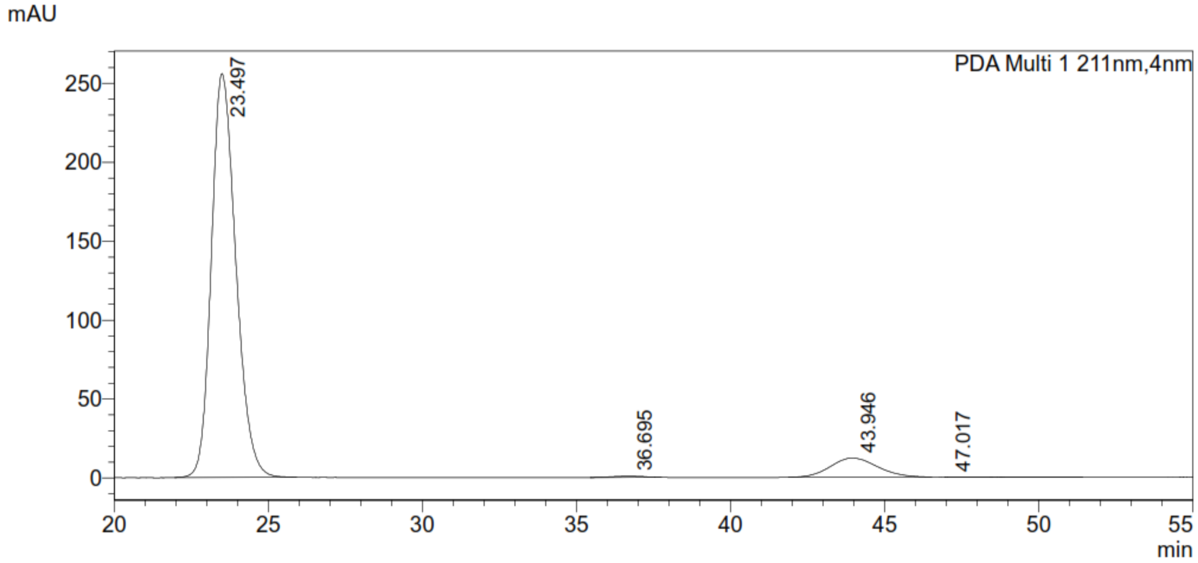

<Peak Table>

PDA Ch1 211nm

Peak#	Ret. Time	Area%
1	23.497	91.281
2	36.695	0.387
3	43.946	8.316
4	47.017	0.016
Total		100.000

6. Epimerisation Experiments

6.1. Epimerisation of Amide **14**

To a 5 ml round bottomed flask was charged with amide **14** (51.6 mg, 0.13 mmol) was added EtOAc (3.0 ml, 0.04 M), $^{i}Pr_2NEt$ (28 µl, 0.16 mmol) and rac-HyperBTM (2.0 mg, 0.01 mmol). The mixture was stirred for 3 h at 0 °C. Morpholine (33 µl, 0.38 mmol) was added and the reaction was left to be stirred overnight. The solvent was removed under reduced pressure and the mixture columned through pipette (Hexane:EtOAc 1:1). Analysis by ^{1}H NMR showed no change suggesting that compound **14** is stable under the reaction conditions.

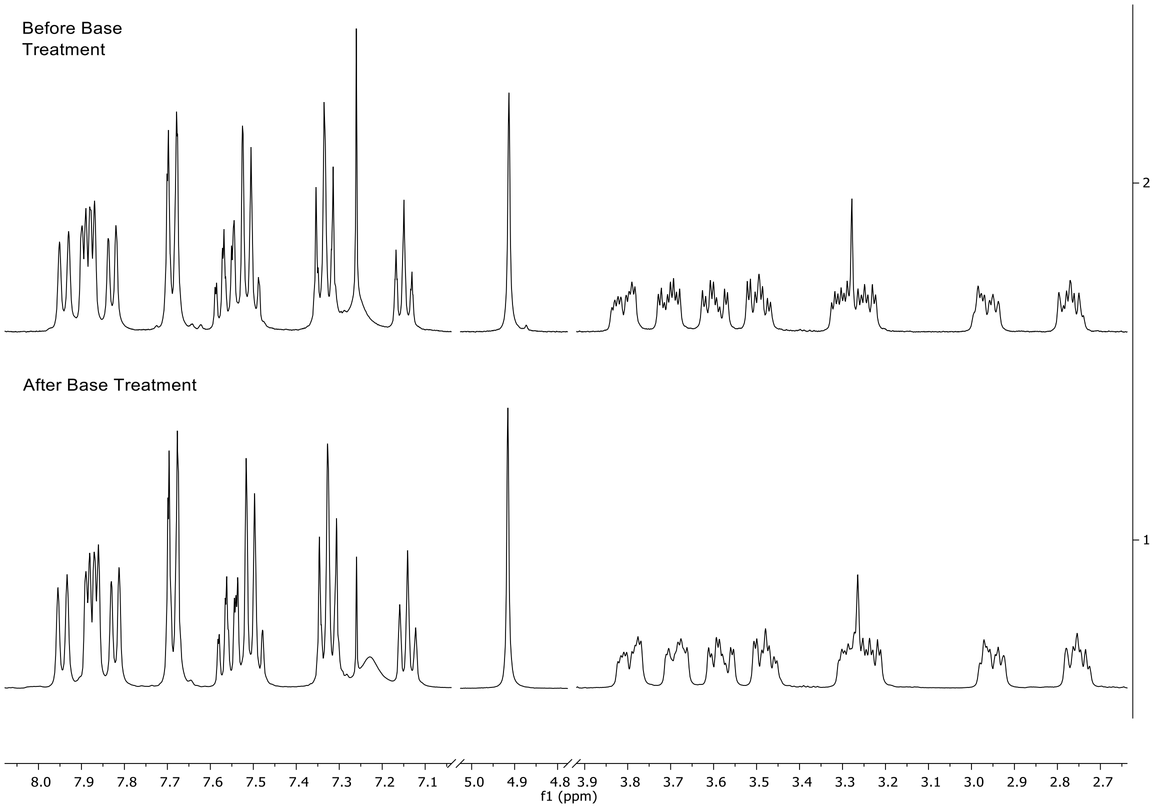

6.2. Epimerisation of Lactone **13**

A sample of Lactone **13** (1.47 µmol) was transferred to an NMR tube using 750 µl $CDCl_3$. Hünig's base (2.5 µl, 14 µmol) was added and the reaction was monitored by ^{1}H NMR.

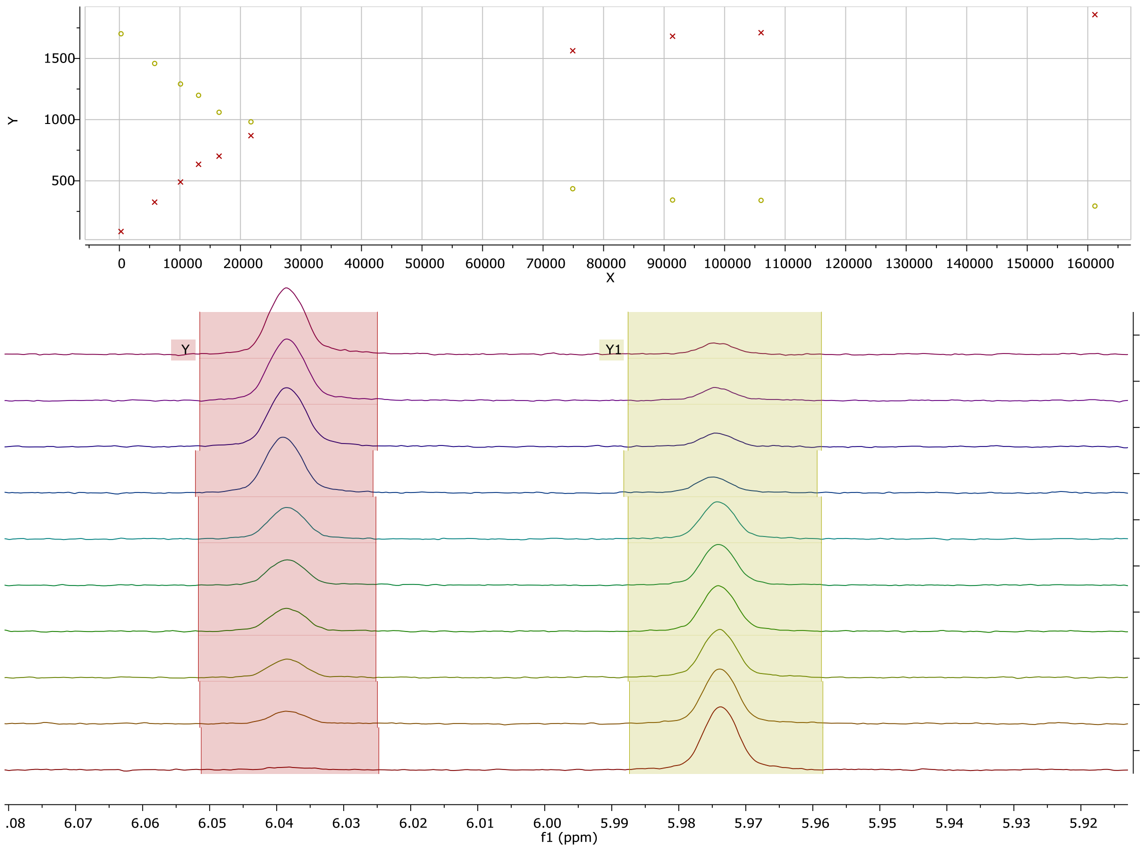

Figure S1: Monitoring of epimerisation of **13** to **12** using ^{1}H NMR analysed with MNova.

Table S2: Data for the monitoring of the epimerisation of **13** to **12** *via* ^{1}H NMR.

#	Time [h]	Integral Major (6.051,6.025)	Major [M]·10^8	Integral Minor (5.987,5.958)	Minor [M]·10^8	Integral Hünig's base (3.114,2.979)	d.r. Minor/Major	d.e. Major/Minor
0	0.0						97.0	-0.94
1	0.1	85	2.3	1701	47.0	34654	95.2	-0.90
2	1.6	325	9.0	1460	40.3	34657	81.8	-0.64
3	2.8	490	13.5	1292	35.7	34665	72.5	-0.45
4	3.6	635	17.3	1198	32.6	35190	65.4	-0.31
5	4.6	703	19.5	1059	29.4	34464	60.1	-0.20
6	6.0	868	23.2	980	26.2	35754	53.0	-0.06
7	20.8	1564	38.3	437	10.7	39123	21.8	0.56
8	25.4	1683	40.5	342	8.3	39723	16.9	0.66
9	29.5	1710	40.5	340	8.1	40414	16.6	0.67
10	44.8	1859	41.7	294	6.6	42633	13.6	0.73

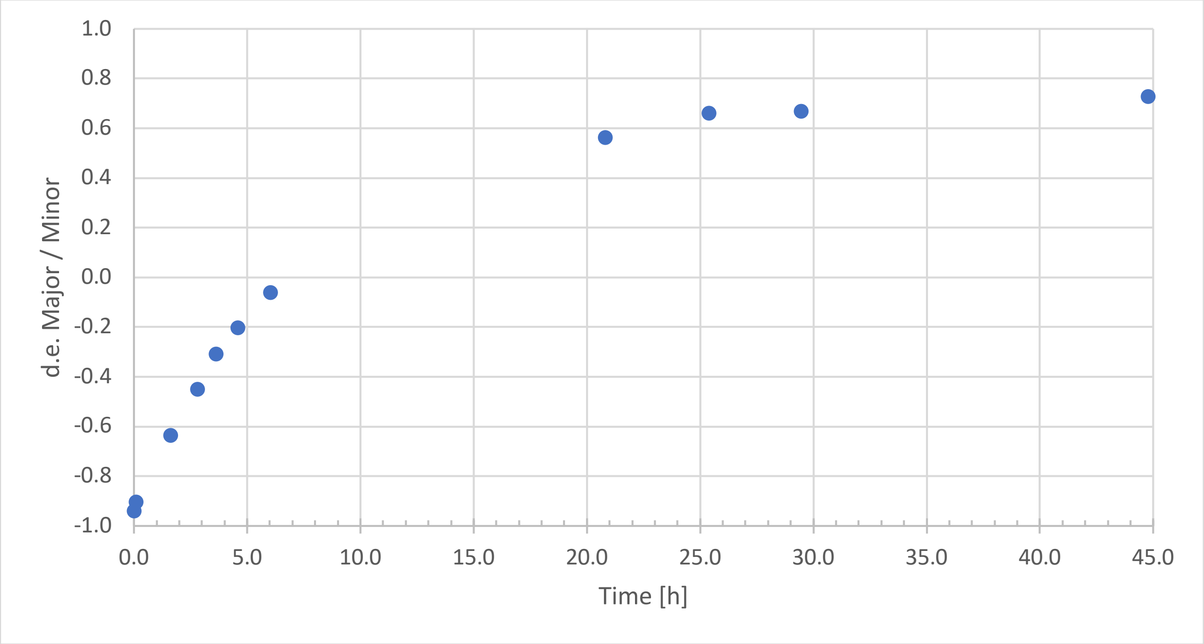

Figure S2: Plot of the change of d.e. over time. Negative d.e. indicates excess of the minor diastereomer **13**, positive d.e. indicates excess of the major diastereomer **12**.

7. Crystallographic Data

X-ray diffraction data for compound (3*S*,4*R*)-**13** were collected at 125 K using a Rigaku MM-007HF High Brilliance RA generator/confocal optics [Cu Kα radiation (λ = 1.54187 Å)] with XtaLAB P200 diffractometer. Diffraction data for compound (1'*R*,4*R*)-**21** were collected at 173 K using a Rigaku FR-X Ultrahigh Brilliance Microfocus RA generator/confocal optics [Mo Kα radiation (λ = 0.71073 Å)] with XtaLAB P200 diffractometer. Intensity data for both structures were collected using ω steps accumulating area detector images spanning at least a hemisphere of reciprocal space. Data for both compounds were collected using CrystalClear and processed (including correction for Lorentz, polarization and absorption) using either CrystalClear or CrysAlisPro.[26] The structures were solved by dual-space methods (SHELXT)[27] and refined by full-matrix least-squares against F^2 (SHELXL-2019/3).[28] Non-hydrogen atoms were refined anisotropically, and hydrogen atoms were refined using a riding model, except for the hydrogen atom bound to oxygen in (1'*R*,4*R*)-**21** which was located from the difference Fourier map and refined isotropically subject to a distance restraint. Crystals of (3*S*,4*R*)-**13** appeared to degrade under prolonged X-ray exposure, even at low temperatures, leading to lower data quality metrics, and a value of the Flack parameter showing wide error bounds. The compound was determined to be predominantly enantiopure by other analytical techniques, so the absolute structure is considered correctly assigned based on the Flack parameter, despite the value of its standard uncertainty. All calculations were performed using the Olex2 interface.[29] Selected crystallographic data are presented in Table #. CCDC 2314276-2314277 contains the supplementary crystallographic data for this paper. These data can be obtained free of charge from The Cambridge Crystallographic Data Centre via www.ccdc.cam.ac.uk/structures.

Table S3: Selected crystallographic data.

	(3*S*,4*R*)-**13**	(1'*R*,4*R*)-**21**
formula	$C_{22}H_{16}N_2O_3$	$C_{23}H_{23}Cl_4N_3O_4$
fw	356.37	547.24
crystal description	Colourless needle	Colourless prism
crystal size [mm^3]	0.24×0.02×0.01	0.21×0.12×0.03
temperature [K]	125	173
space group	$P2_12_12_1$	$P2_12_12_1$
a [Å]	6.3985(3)	8.043(2)
b [Å]	11.1440(6)	10.783(3)
c [Å]	24.0417(10)	28.473(8)
vol [Å]^3	1714.29(14)	2469.3(11)
Z	4	4
ρ (calc) [g/cm^3]	1.381	1.472
μ [mm^{-1}]	0.757	0.515
F(000)	744	1128
reflections collected	9455	32369
independent reflections (R_{int})	3412 (0.0548)	4495 (0.0574)
parameters, restraints	245, 0	312, 1
GoF on F^2	1.050	1.028
R_1 [$I > 2\sigma(I)$]	0.0539	0.0461
wR_2 (all data)	0.1545	0.1386
largest diff. peak/hole [e/Å^3]	0.243, -0.342	0.477, -0.456
Flack parameter	0.1(2)	-0.02(2)

checkCIF/PLATON report

Structure factors have been supplied for datablock(s) 1R,4R-21, 3S,4R-13

THIS REPORT IS FOR GUIDANCE ONLY. IF USED AS PART OF A REVIEW PROCEDURE FOR PUBLICATION, IT SHOULD NOT REPLACE THE EXPERTISE OF AN EXPERIENCED CRYSTALLOGRAPHIC REFEREE.

No syntax errors found. CIF dictionary Interpreting this report

Datablock: 3S,4R-13

Bond precision: C-C = 0.0061 A Wavelength=1.54184

Cell:	a=6.3985(3)	b=11.1440(6)	c=24.0417(10)
	alpha=90	beta=90	gamma=90
Temperature:	125 K		

	Calculated	Reported
Volume	1714.29(14)	1714.29(14)
Space group	P 21 21 21	P 21 21 21
Hall group	P 2ac 2ab	P 2ac 2ab
Moiety formula	C22 H16 N2 O3	C22 H16 N2 O3
Sum formula	C22 H16 N2 O3	C22 H16 N2 O3
Mr	356.37	356.37
Dx,g cm-3	1.381	1.381
Z	4	4
Mu (mm-1)	0.757	0.757
F000	744.0	744.0
F000'	746.31	
h,k,lmax	7,13,30	7,13,29
Nref	3474[2022]	3412
Tmin,Tmax	0.982,0.992	0.579,1.000
Tmin'	0.834	

Correction method= # Reported T Limits: Tmin=0.579 Tmax=1.000
AbsCorr = MULTI-SCAN

Data completeness= 1.69/0.98 Theta(max)= 74.390

R(reflections)= 0.0539(2542) wR2(reflections)= 0.1545(3412)

S = 1.050 Npar= 245

The following ALERTS were generated. Each ALERT has the format
test-name_ALERT_alert-type_alert-level.
Click on the hyperlinks for more details of the test.

Alert level C

```
PLAT340_ALERT_3_C Low Bond Precision on  C-C Bonds ...............    0.00613 Ang.
PLAT911_ALERT_3_C Missing FCF Refl Between Thmin & STh/L=     0.600          3 Report
                    2 12  0,    2 12  1,    3  0  7,
```

Alert level G

```
PLAT380_ALERT_4_G Incorrectly? Oriented X(sp2)-Methyl Moiety .....          C25 Check
PLAT398_ALERT_2_G Deviating  C-O-C   Angle From 120 for O6        .        91.7 Degree
PLAT432_ALERT_2_G Short Inter X...Y Contact  C12       ..C17      .        3.20 Ang.
                                                -1+x,y,z  =      1_455 Check
PLAT912_ALERT_4_G Missing # of FCF Reflections Above STh/L=  0.600            7 Note
PLAT941_ALERT_3_G Average HKL Measurement Multiplicity ..........           4.7 Low
PLAT978_ALERT_2_G Number C-C Bonds with Positive Residual Density.            0 Info
```

0 **ALERT level A** = Most likely a serious problem - resolve or explain
0 **ALERT level B** = A potentially serious problem, consider carefully
2 **ALERT level C** = Check. Ensure it is not caused by an omission or oversight
6 **ALERT level G** = General information/check it is not something unexpected

0 ALERT type 1 CIF construction/syntax error, inconsistent or missing data
3 ALERT type 2 Indicator that the structure model may be wrong or deficient
3 ALERT type 3 Indicator that the structure quality may be low
2 ALERT type 4 Improvement, methodology, query or suggestion
0 ALERT type 5 Informative message, check

Datablock: 1R,4R-21

Bond precision:	C-C = 0.0063 A		Wavelength=0.71073
Cell:	a=8.043(2)	b=10.783(3)	c=28.473(8)
	alpha=90	beta=90	gamma=90
Temperature:	173 K		

	Calculated	Reported
Volume	2469.4(12)	2469.3(11)
Space group	P 21 21 21	P 21 21 21
Hall group	P 2ac 2ab	P 2ac 2ab
Moiety formula	C22 H22 Cl N3 O4, C H Cl3	C22 H22 Cl N3 O4, C H Cl3
Sum formula	C23 H23 Cl4 N3 O4	C23 H23 Cl4 N3 O4
Mr	547.24	547.24
Dx,g cm-3	1.472	1.472
Z	4	4
Mu (mm-1)	0.515	0.515
F000	1128.0	1128.0
F000'	1130.78	
h,k,lmax	9,12,34	9,12,34
Nref	4513[2595]	4495
Tmin,Tmax	0.929,0.985	0.588,1.000
Tmin'	0.897	

Correction method= # Reported T Limits: Tmin=0.588 Tmax=1.000
AbsCorr = MULTI-SCAN

Data completeness= 1.73/1.00 Theta(max)= 25.338

R(reflections)= 0.0462(3977) wR2(reflections)= 0.1390(4495)

S = 1.031 Npar= 312

The following ALERTS were generated. Each ALERT has the format
test-name_ALERT_alert-type_alert-level.
Click on the hyperlinks for more details of the test.

Alert level C

```
PLAT244_ALERT_4_C Low    'Solvent' Ueq as Compared to Neighbors of      C31 Check
PLAT340_ALERT_3_C Low Bond Precision on  C-C Bonds ...............   0.0063 Ang.
PLAT911_ALERT_3_C Missing FCF Refl Between Thmin & STh/L=    0.600        8 Report
                  3  3  0,   2  0  1,   4  6  2,   4  1  3,   1  4  3,   6  2  5,
                  1  6  5,   1  3  8,
```

Alert level G

```
PLAT002_ALERT_2_G Number of Distance or Angle Restraints on AtSite          2 Note
PLAT172_ALERT_4_G The CIF-Embedded .res File Contains DFIX Records          1 Report
PLAT432_ALERT_2_G Short Inter X...Y Contact  Cl1      ..C16       .      3.22 Ang.
                                         1/2+x,3/2-y,1-z  =       4_566 Check
PLAT860_ALERT_3_G Number of Least-Squares Restraints .............          1 Note
PLAT910_ALERT_3_G Missing # of FCF Reflection(s) Below Theta(Min).          2 Note
                  0  1  1,   0  0  2,
PLAT933_ALERT_2_G Number of HKL-OMIT Records in Embedded .res File          2 Note
                  0  1  1,   2  0  1,
```

```
PLAT978_ALERT_2_G Number C-C Bonds with Positive Residual Density.         3 Info
```

```
  0 ALERT level A = Most likely a serious problem - resolve or explain
  0 ALERT level B = A potentially serious problem, consider carefully
  3 ALERT level C = Check. Ensure it is not caused by an omission or oversight
  7 ALERT level G = General information/check it is not something unexpected

  0 ALERT type 1 CIF construction/syntax error, inconsistent or missing data
  4 ALERT type 2 Indicator that the structure model may be wrong or deficient
  4 ALERT type 3 Indicator that the structure quality may be low
  2 ALERT type 4 Improvement, methodology, query or suggestion
  0 ALERT type 5 Informative message, check
```

It is advisable to attempt to resolve as many as possible of the alerts in all categories. Often the minor alerts point to easily fixed oversights, errors and omissions in your CIF or refinement strategy, so attention to these fine details can be worthwhile. In order to resolve some of the more serious problems it may be necessary to carry out additional measurements or structure refinements. However, the purpose of your study may justify the reported deviations and the more serious of these should normally be commented upon in the discussion or experimental section of a paper or in the "special_details" fields of the CIF. checkCIF was carefully designed to identify outliers and unusual parameters, but every test has its limitations and alerts that are not important in a particular case may appear. Conversely, the absence of alerts does not guarantee there are no aspects of the results needing attention. It is up to the individual to critically assess their own results and, if necessary, seek expert advice.

Publication of your CIF in IUCr journals

A basic structural check has been run on your CIF. These basic checks will be run on all CIFs submitted for publication in IUCr journals (*Acta Crystallographica*, *Journal of Applied Crystallography*, *Journal of Synchrotron Radiation*); however, if you intend to submit to *Acta Crystallographica Section C* or *E* or *IUCrData*, you should make sure that full publication checks are run on the final version of your CIF prior to submission.

Publication of your CIF in other journals

Please refer to the *Notes for Authors* of the relevant journal for any special instructions relating to CIF submission.

PLATON version of 29/11/2023; check.def file version of 14/09/2023

Datablock 3S,4R-13 - ellipsoid plot

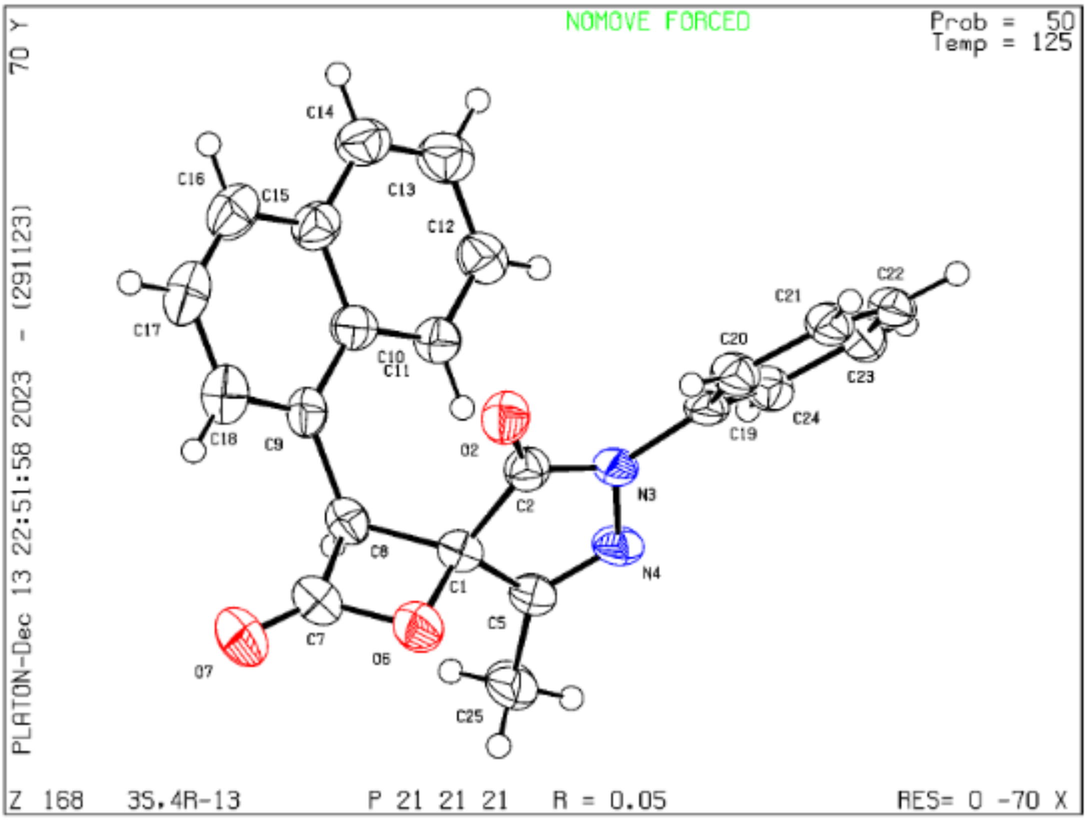

Datablock 1R,4R-21 - ellipsoid plot

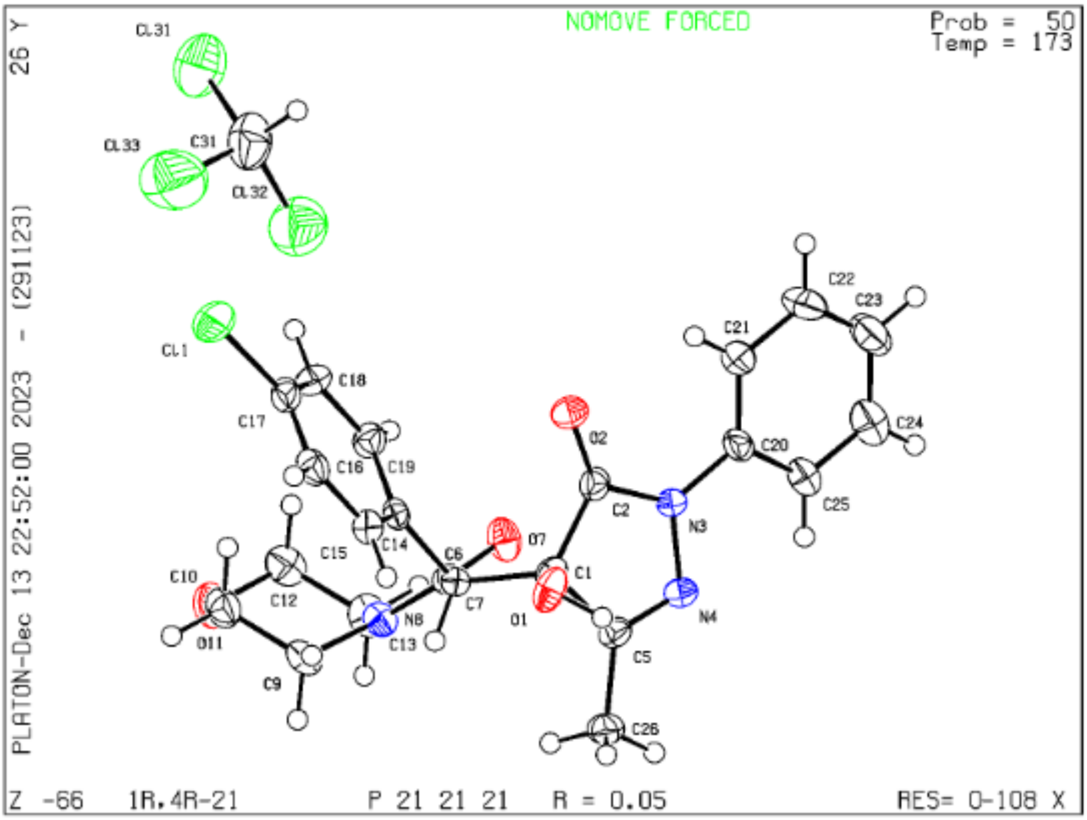

8. Computation

8.1. Computational details

Geometry optimisations were performed with the *meta*-hybrid M06-2X functional[30] using the double-ζ, def2-SVP basis set from the redefinition of the Ahlrichs family of basis sets.[31] Implicit solvation was considered through the use of the SMD model employing the parameters of dichloromethane (ε = 8.93).[32] An ultrafine integration grid (99 radial shells with 590 angular points per shell) was used for all calculations and all species were formally treated as closed-shell systems with restricted Kohn-Sham DFT used throughout. The nature of minima and transition states located were verified by the computation of harmonic frequencies at the same level of theory. Single-point energies (E_{sp}) were also evaluated using the M06-2X functional[30] with a larger, triple-ζ, def2-TZVP basis. Implicit solvation was also included at this level of theory using the same ultrafine integration grid (99,590). Additional empirical dispersion corrections were not included as the functional implicitly accounts for dispersion due to the nature of its construction. Thermochemistry was evaluated at 1 atm and 298.15 K using thermodynamic calculations at the level of geometry optimisation (thermal corrections to enthalpy, $\delta H_{298.15}$, and entropies $S_{298.15}$) in combination with energetics obtained from single-point calculations. Quasi-rigid-rotor entropies were evaluated at 298 K with *GoodVibes, v3.2*[33] following the Truhlar method and a 100 cm^{-1} cutoff.[34] Gibbs free energy was calculated at 298 K using Equation **1**, with additional Martin Hay Pratt empirical entropic corrections included (S_{MHP} = 3.52 kcal/mol per particle, evaluated at 382 atm to mimic bulk dichloromethane).[35] All computations were performed using the Gaussian16, C.01 programme[36] with visualisation of structures using CYLview20[37] and GaussView6.1.1[36] and of non-covalent interactions performed using NCIPlot 4.0.[38] This and similar levels of DFT have previously been used successfully to rationalise reactivities and selectivities of organocatalytic reactions with isothioureas.[39]

$$G_{298.15} = E_{sp} + \delta H_{298.15} - TS_{298.15} + S_{MHP} \qquad (1)$$

8.2. Interaction and Reorganisation Energies

Following the activation-strain model,[40] both interaction and reorganisation energies were calculated from the respective geometry by fragmenting into the morpholine nucleophile and spirocyclic electrophile. Single-point energies of the TS and each fragment (in the geometry of the TS) were computed in the gas-phase and the interaction energy is given by Equation **2**. Reorganisation energies are calculated by the gas-phase single-point of the relaxed geometry of each fragment (*e.g.* minima of each reactant) and this is given by Equation **3**.

$$\Delta E_{interaction} = E_{complex} - \Sigma E_{rigid\_fragments} \quad (2)$$

$$\Delta E_{reorg} = \Sigma(E_{rigid\_fragment} - E_{relaxed\_fragment}) \quad (3)$$

8.3. Computational Discussion

The energy difference between $TS1_{major/minor}$ (and tetrahedral intermediates) is larger than $TS2_{major/minor}$ due to a more pronounced reorganisation required for these species. For nucleophilic attack through the minor pathway, the aromatic group is oriented towards the approaching nucleophile and must move out of the way at a significant energetic cost for this pathway. As such, the major pathway is favoured with $\Delta\Delta E_{reorg}$ = 10.54, 6.77 and 2.32 kcal/mol for TS1, the tetrahedral intermediate and TS2 respectively.

Table S4. Comparison of N–H bond lengths across structures. Bond lengths in Å (M06-2X$_{SMD}$/def2-SVP).

	TS1	tetrahedral intermediate	TS2	morpholine (axial H)[a]	morpholine (equatorial H)
major	1.025	1.032	1.056	1.021	1.018
minor	1.026	1.031	1.054		

[a] morpholine was assumed to react in this NH-axial conformation, so that the lactone moiety will end up in the more favourable equatorial position.

8.4. Treatment of Low-Lying Vibrational Frequencies

We found that corrections to the rigid-rotor approximation of harmonic frequencies was important for this system to accurately reproduce the experimental selectivities. These were performed using *quasi-harmonic* (*qh*) corrections from the *GoodVibes, v3.2* programme.[33] In essence, in the simplest case proposed by Truhlar,[34] vibrational modes below 100 cm^{-1} are all scaled to 100 cm^{-1} allow for an identical contribution of each mode towards the entropy of the system. Alternatively, proposed by Grimme,[41] vibrational modes below 100 cm^{-1} are treated instead as free-rotors, or rotations, alongside scaling to interpolate between the rotational and harmonic vibrations (this method is used by default in ORCA calculations). More methodological details are available in the references provided.

Regardless of the entropic corrections, calculations of the spirocyclic system gave good agreement with the *enrichment* found in the DyKAT process, with raw electronic energies, enthalpies, and free energies replicating a larger selectivity between the transition states (kinetic) compared to between the two β-lactone diastereomeric [2+2] products (thermodynamic).

Table S5. Comparison of the calculated difference in thermodynamic and kinetic selectivities derived from different energy terms and employing different *quasi-harmonic* corrections to entropy. Relative energy values are in kcal/mol.

	$\Delta\Delta E_{sp}$	$\Delta\Delta H$ ($E_{sp} + \delta H$)	$\Delta\Delta G$ ($E_{sp} + \delta H - T.S$)	$\Delta\Delta G$ (*qh*-Truhlar)	$\Delta\Delta G$ (*qh*-Grimme)	$\Delta\Delta G$ (expt estimate)
thermodynamic	0.29	0.34	–0.22[a]	0.31	0.07	0.50
kinetic	0.67	1.11	0.58	0.92	0.76	1.18
enrichment	0.38	0.77	0.80	0.61	0.69	0.68

[a] negative value indicates that the computational "prediction" was in the wrong direction relative to experiment.

8.5. Computational Data

Raw data and cartesian coordinates obtained from geometry optimisation and frequency calculations with subsequent single-point energy calculations.

```
---------------------------------------
morph-axH
Frequencies, energies and thermodynamic properties:
 Lowest Vibrational Mode (1/cm) =                    249.9680
 2nd Lowest Vibrational Mode (1/cm) =                270.7806
 E(RM062X) (a.u.) =                              -287.454708902
 Thermal correction to Enthalpy (a.u.) =             0.142096
 Thermal correction to Gibbs Free Energy (a.u.) =    0.107445
 Total Entropy (cal/Kmol) =                          72.929
 Total Entropy qh-Truhlar (cal/Kmol) =               72.9281
 Total Entropy qh-Grimme (cal/Kmol) =                72.9449
 Esp(RM062X) (a.u.) =                            -287.788872074
 Esp(RM062X) gas (a.u.) =                        -287.777992668
Optimised cartesian coordinates (Angstrom):
 H    0.000000   1.519036   1.210537
 N    0.000000   1.441072   0.192168
 C   -1.199597   0.718853  -0.221335
 C   -1.168692  -0.745230   0.197982
 O    0.000000  -1.379398  -0.275319
 C    1.168692  -0.745230   0.197982
 C    1.199597   0.718853  -0.221335
 H   -1.270539   0.768286  -1.321305
 H   -2.090983   1.213439   0.192125
 H   -2.027489  -1.296212  -0.211948
 H   -1.211802  -0.812927   1.304667
 H    2.027489  -1.296213  -0.211948
 H    1.211802  -0.812927   1.304667
 H    1.270539   0.768286  -1.321305
 H    2.090983   1.213439   0.192125
```

```
---------------------------------------
morph-eqH
Frequencies, energies and thermodynamic properties:
 Lowest Vibrational Mode (1/cm) =                    267.2973
 2nd Lowest Vibrational Mode (1/cm) =                274.6043
 E(RM062X) (a.u.) =                              -287.455560158
 Thermal correction to Enthalpy (a.u.) =             0.142207
 Thermal correction to Gibbs Free Energy (a.u.) =    0.107658
 Total Entropy (cal/Kmol) =                          72.716
 Total Entropy qh-Truhlar (cal/Kmol) =               72.7155
 Total Entropy qh-Grimme (cal/Kmol) =                72.7302
 Esp(RM062X) (a.u.) =                            -287.789684184
 Esp(RM062X) gas (a.u.) =                        -287.779425383
Optimised cartesian coordinates (Angstrom):
 H    0.683423   2.247305   0.000000
 N    0.664731   1.229858   0.000000
 C   -0.006296   0.740821   1.195832
 C   -0.006296  -0.778825   1.166581
 O   -0.635498  -1.262203   0.000000
 C   -0.006296  -0.778825  -1.166581
 C   -0.006296   0.740821  -1.195832
 H   -1.059312   1.086650   1.263397
 H    0.524256   1.091294   2.093255
 H   -0.554946  -1.187643   2.026256
 H    1.039278  -1.141624   1.209945
 H   -0.554946  -1.187643  -2.026256
 H    1.039278  -1.141624  -1.209945
 H   -1.059312   1.086650  -1.263397
 H    0.524256   1.091294  -2.093255
```

----
spiro-RR
Frequencies, energies and thermodynamic properties:
Lowest Vibrational Mode (1/cm) = 22.6861
2nd Lowest Vibrational Mode (1/cm) = 26.1784
E(RM062X) (a.u.) = -1028.35189031
Thermal correction to Enthalpy (a.u.) = 0.307432
Thermal correction to Gibbs Free Energy (a.u.) = 0.239175
Total Entropy (cal/Kmol) = 143.658
Total Entropy qh-Truhlar (cal/Kmol) = 134.2616
Total Entropy qh-Grimme (cal/Kmol) = 135.7307
Esp(RM062X) (a.u.) = -1029.49738538
Esp(RM062X) gas (a.u.) = -1029.46998018
Optimised cartesian coordinates (Angstrom):

O	-1.178723	-1.984905	-1.179012
C	-0.863305	-1.217552	-0.304758
N	-1.608218	-0.235086	0.305594
C	-2.950225	0.137657	0.061946
C	-3.499752	1.214150	0.771596
C	-4.818262	1.594485	0.536137
C	-5.599409	0.917053	-0.400169
C	-5.044669	-0.152279	-1.101647
C	-3.727497	-0.551324	-0.880093
N	-0.907864	0.397641	1.328267
C	0.279934	-0.070737	1.401769
C	1.263806	0.335408	2.437890
C	0.521063	-1.092814	0.327000
O	1.091581	-2.337712	0.757088
C	2.180732	-2.184749	-0.047685
C	1.680807	-0.904058	-0.695761
C	2.515585	0.344258	-0.663875
C	3.878231	0.310139	-0.357886
C	4.610297	1.498242	-0.307286
C	3.985548	2.719518	-0.555251
C	2.623533	2.754377	-0.864333
C	1.892197	1.570838	-0.923405
O	3.127935	-2.892284	-0.105849
H	-2.891079	1.743459	1.501724
H	-5.235970	2.433897	1.094795
H	-6.631719	1.219816	-0.580708
H	-5.642421	-0.694194	-1.836651
H	-3.307105	-1.388504	-1.430714
H	2.141949	0.812550	1.977480
H	1.613486	-0.554250	2.983055
H	0.795637	1.033956	3.141555
H	1.313699	-1.130073	-1.710585
H	4.369496	-0.644294	-0.159937
H	5.674681	1.465774	-0.068805
H	4.559437	3.646566	-0.510145
H	2.129970	3.707565	-1.060981
H	0.824492	1.593382	-1.160061

----
spiro-RS
Frequencies, energies and thermodynamic properties:
Lowest Vibrational Mode (1/cm) = 16.2854
2nd Lowest Vibrational Mode (1/cm) = 21.6953
E(RM062X) (a.u.) = -1028.35135826
Thermal correction to Enthalpy (a.u.) = 0.307509
Thermal correction to Gibbs Free Energy (a.u.) = 0.238365
Total Entropy (cal/Kmol) = 145.525
Total Entropy qh-Truhlar (cal/Kmol) = 134.3437
Total Entropy qh-Grimme (cal/Kmol) = 136.6441
Esp(RM062X) (a.u.) = -1029.49692544
Esp(RM062X) gas (a.u.) = -1029.46648816
Optimised cartesian coordinates (Angstrom):

O	-0.014851	0.266063	-1.707498
C	-0.186077	-0.490193	-0.785214
N	-1.314721	-0.704203	-0.026034
C	-2.577280	-0.076450	-0.125335
C	-2.768916	1.014026	-0.985907
C	-4.024284	1.615567	-1.057337
C	-5.088819	1.150743	-0.286268
C	-4.887079	0.066896	0.568287
C	-3.641434	-0.549581	0.654379
N	-1.154142	-1.760762	0.864957
C	0.033360	-2.228449	0.778478
C	0.528287	-3.392135	1.555812
C	0.856099	-1.427215	-0.183461
O	1.616866	-2.132384	-1.171060
C	2.747514	-1.423471	-0.889333
C	2.156988	-0.720448	0.323748
C	2.230688	0.769872	0.472955
C	1.848946	1.345449	1.690773
C	1.862719	2.729759	1.849226
C	2.268369	3.548627	0.794075
C	2.654732	2.979101	-0.419148
C	2.631655	1.594330	-0.583315
O	3.772333	-1.450284	-1.480045
H	-1.946858	1.384161	-1.592774
H	-4.164614	2.463313	-1.730264
H	-6.067089	1.629102	-0.350217
H	-5.708728	-0.309798	1.180075
H	-3.484625	-1.394200	1.322050
H	0.885206	-4.173246	0.868174
H	-0.275810	-3.795731	2.182296
H	1.375373	-3.098704	2.193947
H	2.509651	-1.231256	1.233515
H	1.539005	0.701814	2.517751
H	1.561631	3.170416	2.801049
H	2.285219	4.632652	0.919090
H	2.974146	3.615192	-1.246207
H	2.931947	1.155305	-1.536604

----

spiro-TS1-RR_R

Frequencies, energies and thermodynamic properties:

Lowest Vibrational Mode (1/cm) = -109.5193
2nd Lowest Vibrational Mode (1/cm) = 20.8819
E(RM062X) (a.u.) = -1315.81517439
Thermal correction to Enthalpy (a.u.) = 0.451142
Thermal correction to Gibbs Free Energy (a.u.) = 0.369985
Total Entropy (cal/Kmol) = 170.809
Total Entropy qh-Truhlar (cal/Kmol) = 157.3959
Total Entropy qh-Grimme (cal/Kmol) = 159.5490
Esp(RM062X) (a.u.) = -1317.28568940
Esp(RM062X) gas (a.u.) = -1317.25212731
Esp(RM062X) gas nuc (a.u.) = -287.777479997
Esp(RM062X) gas spiro (a.u.) = -1029.45120475

Optimised cartesian coordinates (Angstrom):

O	-0.611590	-1.821219	-0.321327
C	-0.954194	-0.789406	0.216189
N	-2.224708	-0.308809	0.402850
C	-3.458387	-0.884339	0.020260
C	-3.505958	-2.128116	-0.626270
C	-4.740657	-2.660567	-0.994305
C	-5.926468	-1.977837	-0.727781
C	-5.868854	-0.742082	-0.083286
C	-4.646315	-0.190942	0.291158
N	-2.236787	0.875492	1.140544
C	-1.035985	1.234428	1.394683
C	-0.693909	2.404737	2.242868
C	-0.035693	0.296116	0.774677
O	1.000331	-0.149272	1.633158
C	2.018690	0.275471	0.736138
N	2.352623	-1.513308	-0.075053
H	1.468319	-1.883937	-0.437543
C	3.352371	-1.360381	-1.128877
C	3.903747	-2.708457	-1.573013
O	4.407532	-3.431155	-0.474696
C	3.412018	-3.664588	0.494958
C	2.842372	-2.353735	1.016768
O	3.102118	0.654526	1.103485
C	0.988411	0.833340	-0.278041
C	0.965942	2.304920	-0.596032
C	1.992396	3.177630	-0.222849
C	1.896995	4.538805	-0.523885
C	0.780756	5.038483	-1.192266
C	-0.244468	4.168745	-1.572681
C	-0.148009	2.810659	-1.281483
H	-2.588814	-2.671221	-0.836509
H	-4.767831	-3.629169	-1.496609
H	-6.887116	-2.404775	-1.019019
H	-6.786797	-0.193010	0.133926
H	-4.602144	0.773110	0.793274
H	0.000671	2.087226	3.034695
H	-1.603631	2.819770	2.693064
H	-0.190134	3.183916	1.651714
H	4.164111	-0.743239	-0.711516
H	2.906494	-0.823361	-1.979042
H	4.729274	-2.568868	-2.284048
H	3.106270	-3.289260	-2.077349
H	3.874745	-4.233140	1.312971
H	2.598150	-4.279670	0.062704
H	3.629174	-1.781422	1.534561
H	2.018049	-2.530511	1.720695
H	0.939240	0.248795	-1.207148
H	2.860233	2.788110	0.308797
H	2.703250	5.212389	-0.227757
H	0.708335	6.103367	-1.420112
H	-1.120787	4.550522	-2.099488
H	-0.952900	2.130425	-1.575179

----

spiro-TS1-RS_R

Frequencies, energies and thermodynamic properties:

Lowest Vibrational Mode (1/cm) = -114.3470
2nd Lowest Vibrational Mode (1/cm) = 23.1969
E(RM062X) (a.u.) = -1315.81189079
Thermal correction to Enthalpy (a.u.) = 0.451350
Thermal correction to Gibbs Free Energy (a.u.) = 0.371791
Total Entropy (cal/Kmol) = 167.446
Total Entropy qh-Truhlar (cal/Kmol) = 156.1521
Total Entropy qh-Grimme (cal/Kmol) = 157.7369
Esp(RM062X) (a.u.) = -1317.28286966
Esp(RM062X) gas (a.u.) = -1317.24728287
Esp(RM062X) gas nuc (a.u.) = -287.777406934
Esp(RM062X) gas spiro (a.u.) = -1029.43448115

Optimised cartesian coordinates (Angstrom):

O	-0.529924	-1.051461	-0.634734
C	-0.967524	-0.430708	0.310980
N	-2.277076	-0.117287	0.571992
C	-3.426344	-0.416311	-0.195833
C	-3.310477	-0.875731	-1.515812
C	-4.464476	-1.151939	-2.247458
C	-5.729097	-0.975690	-1.687700
C	-5.834016	-0.514388	-0.375412
C	-4.694048	-0.233967	0.373365
N	-2.439867	0.489046	1.817449
C	-1.295635	0.640387	2.366004
C	-1.102060	1.211259	3.722509
C	-0.174335	0.171056	1.476905
O	0.747744	-0.725456	2.072758
C	1.894680	0.019835	1.604705
N	2.241480	-0.968430	0.030173
H	1.519951	-0.768682	-0.670916
C	3.580638	-0.634822	-0.470663
C	4.024478	-1.596403	-1.563795
O	3.965861	-2.932345	-1.129186
C	2.659675	-3.279560	-0.736958
C	2.165910	-2.392718	0.393605
O	2.934107	0.059979	2.222852
C	0.956421	1.181280	1.152761
C	1.122152	1.930238	-0.136056
C	2.362078	2.544645	-0.371386
C	2.583798	3.280913	-1.532136
C	1.561912	3.428329	-2.473085
C	0.319229	2.845114	-2.235436
C	0.097474	2.103773	-1.072330
H	-2.330132	-1.019048	-1.962093
H	-4.365169	-1.509313	-3.273940
H	-6.625612	-1.195018	-2.269209
H	-6.815975	-0.369310	0.078388
H	-4.775695	0.128353	1.396026
H	-0.494448	0.521034	4.326060
H	-2.072148	1.373014	4.207052
H	-0.561812	2.168325	3.667698
H	4.260984	-0.687775	0.391750
H	3.578688	0.393413	-0.851918
H	5.064120	-1.383055	-1.846639
H	3.388340	-1.460002	-2.460708
H	2.677218	-4.327921	-0.410062
H	1.968127	-3.198435	-1.599131
H	2.794210	-2.527917	1.288107
H	1.129538	-2.634698	0.652428
H	1.005033	1.895061	1.990918
H	3.157520	2.435387	0.370241
H	3.556216	3.747194	-1.700454
H	1.732419	4.006185	-3.382950
H	-0.491941	2.969886	-2.954742
H	-0.896087	1.686737	-0.897379

----
spiro-TS2-RR_R
Frequencies, energies and thermodynamic properties:
Lowest Vibrational Mode (1/cm) = -131.2236
2nd Lowest Vibrational Mode (1/cm) = 22.4310
E(RM062X) (a.u.) = -1315.79848235
Thermal correction to Enthalpy (a.u.) = 0.451050
Thermal correction to Gibbs Free Energy (a.u.) = 0.371996
Total Entropy (cal/Kmol) = 166.383
Total Entropy qh-Truhlar (cal/Kmol) = 155.6006
Total Entropy qh-Grimme (cal/Kmol) = 156.9708
Esp(RM062X) (a.u.) = -1317.27479177
Esp(RM062X) gas (a.u.) = -1317.23403868
Esp(RM062X) gas nuc (a.u.) = -287.773630997
Esp(RM062X) gas spiro (a.u.) = -1029.33841878
Optimised cartesian coordinates (Angstrom):

O	1.111866	1.477398	-0.108336
C	1.044927	0.313330	0.278940
N	2.063846	-0.573763	0.373706
C	3.430106	-0.413148	0.050705
C	3.950585	0.841983	-0.297588
C	5.302370	0.957855	-0.617732
C	6.144546	-0.153118	-0.593388
C	5.619186	-1.396784	-0.242299
C	4.271220	-1.534523	0.078599
N	1.645371	-1.791052	0.931931
C	0.383406	-1.749167	1.132219
C	-0.318165	-2.853297	1.840701
C	-0.248026	-0.397524	0.764490
O	-0.877306	0.228754	1.771032
C	-2.175429	0.729548	-0.013632
N	-1.512224	2.085787	-0.037499
H	-0.469628	1.946036	0.057405
C	-1.751897	2.749489	-1.356711
C	-1.102782	4.123267	-1.339801
O	-1.597069	4.902904	-0.281931
C	-1.322198	4.310406	0.965478
C	-1.978023	2.947671	1.086732
O	-3.354946	0.717201	0.140631
C	-1.268753	-0.420677	-0.448357
C	-2.014242	-1.720394	-0.607288
C	-2.997116	-2.127573	0.306296
C	-3.609373	-3.372412	0.173917
C	-3.241679	-4.234534	-0.860747
C	-2.258279	-3.841064	-1.768482
C	-1.654798	-2.589205	-1.643960
H	3.303141	1.714239	-0.316552
H	5.698613	1.938769	-0.886485
H	7.201354	-0.050779	-0.843980
H	6.263781	-2.277272	-0.216979
H	3.860225	-2.503396	0.354409
H	-1.059961	-2.410423	2.519865
H	0.404300	-3.451679	2.410519
H	-0.847376	-3.510587	1.135859
H	-2.839226	2.828072	-1.490237
H	-1.324504	2.114217	-2.144474
H	-1.332740	4.640348	-2.280000
H	-0.004216	4.013889	-1.262290
H	-1.718534	4.971263	1.746559
H	-0.229289	4.217252	1.112921
H	-3.069577	3.022748	1.007487
H	-1.692549	2.425078	2.007080
H	-0.802748	-0.157402	-1.413377
H	-3.268653	-1.472897	1.133548
H	-4.374430	-3.675675	0.891042
H	-3.721199	-5.210038	-0.959651
H	-1.962892	-4.507062	-2.581242
H	-0.886703	-2.282331	-2.358126

----
spiro-TS2-RR_S
Frequencies, energies and thermodynamic properties:
Lowest Vibrational Mode (1/cm) = -180.2882
2nd Lowest Vibrational Mode (1/cm) = 18.6614
E(RM062X) (a.u.) = -1315.78461218
Thermal correction to Enthalpy (a.u.) = 0.451984
Thermal correction to Gibbs Free Energy (a.u.) = 0.372691
Total Entropy (cal/Kmol) = 166.887
Total Entropy qh-Truhlar (cal/Kmol) = 155.5985
Total Entropy qh-Grimme (cal/Kmol) = 157.1833
Esp(RM062X) (a.u.) = -1317.26278359
Esp(RM062X) gas (a.u.) = -1317.21335566
Esp(RM062X) gas nuc (a.u.) = -287.774445503
Esp(RM062X) gas spiro (a.u.) = -1029.34241351
Optimised cartesian coordinates (Angstrom):

O	1.917597	-0.592881	2.126722
C	1.611285	-0.591019	0.957332
N	2.457921	-0.558156	-0.131544
C	3.866111	-0.536342	-0.160177
C	4.527669	-0.335396	-1.381279
C	5.919260	-0.307457	-1.420936
C	6.670263	-0.476309	-0.257446
C	6.007345	-0.677976	0.952561
C	4.615252	-0.711606	1.014173
N	1.759327	-0.524764	-1.336625
C	0.502800	-0.522382	-1.100415
C	-0.488380	-0.532298	-2.212048
C	0.174820	-0.636970	0.387871
O	-0.474678	-1.796111	0.711314
C	-1.950065	-0.369426	1.382567
N	-2.749261	-0.941187	0.209190
H	-2.020604	-1.256532	-0.447412
C	-3.464179	-2.180305	0.640355
C	-4.207526	-2.764354	-0.545210
O	-5.108657	-1.830938	-1.088810
C	-4.441421	-0.687088	-1.551154
C	-3.696281	0.019911	-0.429550
O	-2.480381	-0.425055	2.450225
C	-0.696679	0.449520	1.065878
C	-0.896021	1.846841	0.519491
C	-2.026167	2.596212	0.877619
C	-2.191627	3.902206	0.420094
C	-1.221427	4.488408	-0.394155
C	-0.082949	3.760652	-0.736588
C	0.080988	2.451226	-0.281127
H	3.943323	-0.204435	-2.289598
H	6.419661	-0.149897	-2.378287
H	7.760311	-0.452350	-0.293960
H	6.578382	-0.815243	1.872773
H	4.108149	-0.868340	1.962215
H	-1.229018	0.273276	-2.082359
H	-1.015727	-1.500488	-2.236795
H	0.020150	-0.399181	-3.174895
H	-4.154132	-1.889780	1.440763
H	-2.698301	-2.862611	1.027794
H	-4.783831	-3.636713	-0.212627
H	-3.488259	-3.100352	-1.316847
H	-5.188781	-0.000963	-1.969302
H	-3.728133	-0.953588	-2.354941
H	-4.387317	0.360943	0.353479
H	-3.126291	0.872358	-0.817841
H	-0.258189	0.542884	2.074280
H	-2.786867	2.155131	1.526756
H	-3.080884	4.466170	0.707250
H	-1.348977	5.511189	-0.752479
H	0.690111	4.212790	-1.360315
H	0.989832	1.908061	-0.547535

----

spiro-TS2-RS_R
Frequencies, energies and thermodynamic properties:
Lowest Vibrational Mode (1/cm) = -82.6504
2nd Lowest Vibrational Mode (1/cm) = 13.9111
E(RM062X) (a.u.) = -1315.79701844
Thermal correction to Enthalpy (a.u.) = 0.451751
Thermal correction to Gibbs Free Energy (a.u.) = 0.371846
Total Entropy (cal/Kmol) = 168.176
Total Entropy qh-Truhlar (cal/Kmol) = 156.2341
Total Entropy qh-Grimme (cal/Kmol) = 158.1578
Esp(RM062X) (a.u.) = -1317.27372088
Esp(RM062X) gas (a.u.) = -1317.23300290
Esp(RM062X) gas nuc (a.u.) = -287.773529552
Esp(RM062X) gas spiro (a.u.) = -1029.33482033
Optimised cartesian coordinates (Angstrom):
O -0.116063 -1.456943 -0.134836
C -0.602841 -0.611615 0.612937
N -1.919474 -0.353264 0.810626
C -3.050272 -0.931765 0.192663
C -2.924489 -2.046241 -0.650079
C -4.062800 -2.583203 -1.249571
C -5.323029 -2.032208 -1.021340
C -5.439192 -0.926457 -0.178736
C -4.314601 -0.373100 0.427681
N -2.122386 0.618626 1.807841
C -0.982585 1.012172 2.229010
C -0.809211 1.983383 3.336857
C 0.195950 0.329439 1.541057
O 1.081642 -0.302194 2.326209
C 2.414048 0.676627 0.751310
N 2.514638 -0.685606 0.094673
H 1.542861 -1.077338 -0.019349
C 3.169153 -0.567911 -1.249203
C 3.296799 -1.950464 -1.865378
O 4.032889 -2.810765 -1.035969
C 3.410638 -2.969408 0.216638
C 3.282155 -1.647248 0.948798
O 3.442026 1.178963 1.081874
C 1.050506 1.371079 0.746072
C 0.572160 2.035173 -0.529967
C 0.190083 1.326370 -1.679988
C -0.267733 2.000112 -2.812514
C -0.356726 3.392003 -2.818098
C 0.015344 4.108509 -1.681219
C 0.474320 3.434047 -0.550732
H -1.947295 -2.485880 -0.829583
H -3.955594 -3.451074 -1.902831
H -6.207346 -2.461622 -1.494325
H -6.417918 -0.482402 0.011839
H -4.403142 0.489208 1.085060
H -0.079336 1.562608 4.044170
H -1.761621 2.180212 3.843644
H -0.398844 2.933763 2.961673
H 4.157241 -0.119081 -1.082149
H 2.565432 0.098158 -1.877961
H 3.826617 -1.865541 -2.822733
H 2.289878 -2.366771 -2.061962
H 4.028909 -3.649731 0.815951
H 2.411933 -3.430140 0.093413
H 4.265244 -1.195209 1.131745
H 2.715733 -1.738561 1.881744
H 1.230232 2.172409 1.475466
H 0.241774 0.236935 -1.705748
H -0.559926 1.429036 -3.695435
H -0.717174 3.915688 -3.705125
H -0.051709 5.197778 -1.671732
H 0.764593 4.001019 0.336850

----

spiro-TS2-RS_S
Frequencies, energies and thermodynamic properties:
Lowest Vibrational Mode (1/cm) = -227.4478
2nd Lowest Vibrational Mode (1/cm) = 12.4531
E(RM062X) (a.u.) = -1315.79179678
Thermal correction to Enthalpy (a.u.) = 0.451506
Thermal correction to Gibbs Free Energy (a.u.) = 0.370007
Total Entropy (cal/Kmol) = 171.529
Total Entropy qh-Truhlar (cal/Kmol) = 157.8337
Total Entropy qh-Grimme (cal/Kmol) = 160.1951
Esp(RM062X) (a.u.) = -1317.26759682
Esp(RM062X) gas (a.u.) = -1317.21402866
Esp(RM062X) gas nuc (a.u.) = -287.774715981
Esp(RM062X) gas spiro (a.u.) = -1029.36218281
Optimised cartesian coordinates (Angstrom):
O -0.919467 -0.673995 -2.032983
C -0.868349 -0.975318 -0.864625
N -1.934541 -1.173139 -0.001401
C -3.301955 -0.921089 -0.209224
C -4.205945 -1.110571 0.848133
C -5.559981 -0.845610 0.661695
C -6.035774 -0.387512 -0.567279
C -5.133329 -0.198390 -1.613404
C -3.774306 -0.461556 -1.449954
N -1.523324 -1.695277 1.226648
C -0.245472 -1.754478 1.242569
C 0.539256 -2.336497 2.360232
C 0.388132 -1.143953 0.010756
O 1.485336 -1.689559 -0.546361
C 1.950654 0.339313 -0.715160
N 3.334206 -0.058840 -0.186290
H 3.126734 -0.841459 0.452708
C 4.189382 -0.599799 -1.277707
C 5.537977 -0.987752 -0.699027
O 6.150698 0.105585 -0.058390
C 5.372982 0.585153 1.007176
C 4.013404 1.062579 0.522859
O 1.912479 0.857655 -1.788091
C 0.878298 0.320316 0.374998
C -0.131630 1.430274 0.450273
C -0.709242 1.701824 1.699032
C -1.740538 2.631646 1.822766
C -2.203833 3.311676 0.696425
C -1.631253 3.052523 -0.549478
C -0.607374 2.113993 -0.678276
H -3.835535 -1.462549 1.808577
H -6.249932 -0.998700 1.493821
H -7.097641 -0.180348 -0.707588
H -5.487197 0.159000 -2.582402
H -3.079321 -0.316066 -2.272244
H 1.222446 -1.587281 2.790979
H 1.159753 -3.157453 1.970868
H -0.122992 -2.715201 3.148427
H 4.288776 0.193401 -2.028344
H 3.650584 -1.454811 -1.703375
H 6.198773 -1.315429 -1.511097
H 5.416909 -1.829637 0.009249
H 5.908243 1.424771 1.467848
H 5.235225 -0.202768 1.772950
H 4.117090 1.881429 -0.201890
H 3.380066 1.388250 1.358433
H 1.386075 0.235624 1.347496
H -0.347345 1.170348 2.583228
H -2.181932 2.825550 2.802000
H -3.010714 4.040786 0.788892
H -1.990974 3.579657 -1.435115
H -0.180473 1.895558 -1.656198

----
spiro-prod-RR
Frequencies, energies and thermodynamic properties:
Lowest Vibrational Mode (1/cm) = 19.0296
2nd Lowest Vibrational Mode (1/cm) = 24.2115
E(RM062X) (a.u.) = -1315.86393967
Thermal correction to Enthalpy (a.u.) = 0.452861
Thermal correction to Gibbs Free Energy (a.u.) = 0.370589
Total Entropy (cal/Kmol) = 173.157
Total Entropy qh-Truhlar (cal/Kmol) = 159.7973
Total Entropy qh-Grimme (cal/Kmol) = 162.0030
Esp(RM062X) (a.u.) = -1317.33385951
Optimised cartesian coordinates (Angstrom):

O	0.326903	2.156157	1.887187
H	0.024003	2.786095	1.211955
O	1.268508	1.636981	-0.886565
O	0.102776	-1.354963	-0.260517
O	-4.421809	-2.640928	-0.941273
N	2.481805	0.205415	0.489974
N	2.300316	-0.376048	1.734204
N	-2.057456	-1.758468	0.286154
C	0.406287	0.891514	1.283004
C	1.393641	0.958920	0.105135
C	1.166203	-0.034132	2.209014
C	-1.009559	0.375227	0.938752
H	-1.520463	0.307592	1.910381
C	-0.937298	-0.998458	0.279857
C	-3.357227	-1.389311	0.834302
H	-3.328115	-0.402112	1.305746
H	-3.637638	-2.131512	1.600389
C	-4.397060	-1.398582	-0.279717
H	-5.398207	-1.227249	0.138131
H	-4.168581	-0.585026	-0.995895
C	-3.170975	-2.955391	-1.510121
H	-2.894072	-2.194588	-2.265603
H	-3.272810	-3.926803	-2.012152
C	-2.086793	-3.021035	-0.444077
H	-2.313334	-3.836338	0.262650
H	-1.102192	-3.195274	-0.890140
C	-1.784969	1.354089	0.073133
C	-2.562489	2.348797	0.678530
H	-2.612069	2.401583	1.768891
C	-3.278903	3.259813	-0.099186
H	-3.883739	4.028342	0.385185
C	-3.228348	3.180453	-1.490470
C	-2.454885	2.191262	-2.101186
H	-2.409760	2.126145	-3.189634
C	-1.733899	1.285654	-1.325106
H	-1.116711	0.524007	-1.806935
C	3.663428	-0.068586	-0.226998
C	3.892746	0.496991	-1.491029
H	3.153903	1.159430	-1.933085
C	5.073648	0.202483	-2.170855
H	5.240092	0.648238	-3.153333
C	6.032557	-0.643905	-1.616229
H	6.953223	-0.867100	-2.157184
C	5.797306	-1.201585	-0.359218
H	6.535185	-1.867440	0.092210
C	4.624542	-0.921442	0.336546
H	4.442256	-1.358469	1.315934
C	0.660363	-0.477746	3.533251
H	0.357872	0.397155	4.127793
H	1.436254	-1.038402	4.068060
H	-0.225878	-1.120709	3.409934
H	-3.792798	3.888007	-2.100276

----
spiro-prod-RS
Frequencies, energies and thermodynamic properties:
Lowest Vibrational Mode (1/cm) = 19.3398
2nd Lowest Vibrational Mode (1/cm) = 22.0651
E(RM062X) (a.u.) = -1315.86124153
Thermal correction to Enthalpy (a.u.) = 0.452812
Thermal correction to Gibbs Free Energy (a.u.) = 0.370137
Total Entropy (cal/Kmol) = 174.003
Total Entropy qh-Truhlar (cal/Kmol) = 160.0499
Total Entropy qh-Grimme (cal/Kmol) = 162.4744
Esp(RM062X) (a.u.) = -1317.33208952
Optimised cartesian coordinates (Angstrom):

O	0.309106	-2.358201	1.478301
H	0.270381	-2.030335	2.391845
O	1.661164	0.272798	2.039497
O	0.164835	1.007759	-0.883685
O	-3.913016	3.490196	-0.177404
N	2.538671	-0.585651	0.056069
N	2.079497	-1.417900	-0.955844
N	-1.638941	1.882534	0.168163
C	0.384927	-1.245405	0.619950
C	1.591381	-0.384835	1.026197
C	0.875478	-1.769637	-0.716595
C	-0.912046	-0.412118	0.698631
H	-1.002971	-0.170866	1.771546
C	-0.745999	0.894901	-0.072843
C	-2.716769	1.857896	1.149438
H	-2.487164	2.585180	1.946936
H	-2.819419	0.867262	1.603886
C	-4.025401	2.247937	0.474462
H	-4.310393	1.459520	-0.249071
H	-4.821883	2.339412	1.225008
C	-2.910711	3.466830	-1.167632
H	-2.888640	4.459019	-1.637737
H	-3.159304	2.715634	-1.942159
C	-1.553098	3.140439	-0.564765
H	-0.787260	3.044731	-1.341567
H	-1.257465	3.943027	0.132165
C	-2.143102	-1.191000	0.271722
C	-2.709547	-1.017608	-0.996980
H	-2.269511	-0.304701	-1.699448
C	-3.829166	-1.755478	-1.382312
H	-4.257965	-1.608591	-2.375134
C	-4.396563	-2.677598	-0.502854
C	-3.842542	-2.852693	0.765867
H	-4.284971	-3.566192	1.463231
C	-2.726499	-2.111082	1.151569
H	-2.302171	-2.245819	2.147954
C	3.834274	-0.037320	-0.041352
C	4.608845	-0.306971	-1.178888
H	4.198438	-0.932128	-1.968976
C	5.890782	0.225675	-1.285745
H	6.482905	0.007533	-2.176433
C	6.416905	1.028459	-0.273355
H	7.421851	1.443317	-0.362862
C	5.640763	1.292965	0.854323
H	6.036454	1.918903	1.656302
C	4.355632	0.768471	0.982170
H	3.759307	0.980765	1.865359
C	0.130939	-2.707391	-1.597812
H	-0.642794	-2.173106	-2.167301
H	-0.371863	-3.474393	-0.990688
H	0.825138	-3.183546	-2.301326
H	-5.272901	-3.254560	-0.803106

----
spiro-tet-RR_R
Frequencies, energies and thermodynamic properties:
Lowest Vibrational Mode (1/cm) = 23.8670
2nd Lowest Vibrational Mode (1/cm) = 26.0521
E(RM062X) (a.u.) = -1315.81623477
Thermal correction to Enthalpy (a.u.) = 0.452756
Thermal correction to Gibbs Free Energy (a.u.) = 0.372364
Total Entropy (cal/Kmol) = 169.201
Total Entropy qh-Truhlar (cal/Kmol) = 157.4969
Total Entropy qh-Grimme (cal/Kmol) = 159.1238
Esp(RM062X) (a.u.) = -1317.28747204
Esp(RM062X) gas (a.u.) = -1317.25031249
Esp(RM062X) gas nuc (a.u.) = -287.776614922
Esp(RM062X) gas spiro (a.u.) = -1029.42806291
Optimised cartesian coordinates (Angstrom):
O -0.708141 -1.784268 -0.281690
C -0.989353 -0.716183 0.228507
N -2.223700 -0.156556 0.407067
C -3.492219 -0.658890 0.037205
C -3.617891 -1.906691 -0.590970
C -4.883579 -2.366790 -0.950746
C -6.024237 -1.607627 -0.693519
C -5.889036 -0.368706 -0.066908
C -4.634050 0.110880 0.299035
N -2.154536 1.047954 1.111506
C -0.930575 1.332093 1.352808
C -0.513196 2.517112 2.146345
C 0.005901 0.310099 0.763489
O 0.993112 -0.214902 1.618468
C 2.072636 0.012952 0.636711
N 2.222030 -1.546572 0.005108
H 1.288946 -1.849576 -0.314904
C 3.182276 -1.546111 -1.115224
C 3.456551 -2.966525 -1.582686
O 3.911263 -3.770224 -0.522582
C 2.964459 -3.835547 0.516441
C 2.670068 -2.456100 1.080222
O 3.167791 0.415711 1.018701
C 1.048444 0.729949 -0.322062
C 1.188137 2.200402 -0.612536
C 2.179358 3.007816 -0.042296
C 2.209936 4.377509 -0.314624
C 1.253369 4.957109 -1.146884
C 0.264135 4.156250 -1.722253
C 0.238575 2.788280 -1.461534
H -2.735712 -2.507761 -0.793644
H -4.971924 -3.338802 -1.439300
H -7.009957 -1.977764 -0.978699
H -6.770853 0.239424 0.142468
H -4.528360 1.077646 0.786566
H 0.308550 2.234513 2.819861
H -1.361458 2.898043 2.728130
H -0.144569 3.315766 1.484850
H 4.097625 -1.073163 -0.734303
H 2.777503 -0.934261 -1.933190
H 4.237516 -2.955998 -2.354066
H 2.538542 -3.399561 -2.025921
H 3.378051 -4.476285 1.306032
H 2.027058 -4.298879 0.151042
H 3.573054 -2.003850 1.512973
H 1.882719 -2.491324 1.842937
H 0.872970 0.191392 -1.264617
H 2.918780 2.551046 0.613995
H 2.988785 4.996819 0.134484
H 1.278816 6.029003 -1.351168
H -0.486759 4.598427 -2.379570
H -0.536346 2.163153 -1.914029

----
spiro-tet-RS_R
Frequencies, energies and thermodynamic properties:
Lowest Vibrational Mode (1/cm) = 22.2416
2nd Lowest Vibrational Mode (1/cm) = 25.5032
E(RM062X) (a.u.) = -1315.81225293
Thermal correction to Enthalpy (a.u.) = 0.452620
Thermal correction to Gibbs Free Energy (a.u.) = 0.371597
Total Entropy (cal/Kmol) = 170.526
Total Entropy qh-Truhlar (cal/Kmol) = 158.2399
Total Entropy qh-Grimme (cal/Kmol) = 160.0793
Esp(RM062X) (a.u.) = -1317.28435345
Esp(RM062X) gas (a.u.) = -1317.24682809
Esp(RM062X) gas nuc (a.u.) = -287.776590530
Esp(RM062X) gas spiro (a.u.) = -1029.41730018
Optimised cartesian coordinates (Angstrom):
O -0.534848 -1.063222 -0.663435
C -0.960973 -0.448092 0.295797
N -2.260559 -0.121266 0.569706
C -3.421608 -0.395311 -0.189982
C -3.324644 -0.860097 -1.509588
C -4.489294 -1.113883 -2.232396
C -5.745885 -0.910082 -1.663984
C -5.831812 -0.443653 -0.352138
C -4.680975 -0.185037 0.387877
N -2.402300 0.480418 1.822464
C -1.250175 0.601833 2.362545
C -1.031881 1.159904 3.720206
C -0.144442 0.111639 1.465105
O 0.741630 -0.831465 2.028708
C 1.946971 -0.136744 1.495235
N 2.205068 -1.038864 0.087932
H 1.390635 -0.923899 -0.534380
C 3.447623 -0.611334 -0.588813
C 3.778882 -1.537978 -1.747711
O 3.877032 -2.875196 -1.327126
C 2.670417 -3.311960 -0.751680
C 2.292846 -2.471984 0.454844
O 2.974709 -0.088133 2.159510
C 1.013740 1.085631 1.145066
C 1.152741 1.933458 -0.084713
C 2.362101 2.630442 -0.247834
C 2.573573 3.446391 -1.354782
C 1.569793 3.596170 -2.315744
C 0.356900 2.932583 -2.149831
C 0.146725 2.108473 -1.040550
H -2.350554 -1.025227 -1.962039
H -4.404835 -1.475827 -3.258601
H -6.650946 -1.112252 -2.238436
H -6.807289 -0.277325 0.108310
H -4.747600 0.180957 1.410278
H -0.426680 0.455104 4.309177
H -1.992140 1.334958 4.219459
H -0.475183 2.107266 3.663919
H 4.235811 -0.632881 0.175461
H 3.322998 0.417325 -0.945680
H 4.746596 -1.249324 -2.178404
H 3.007901 -1.440751 -2.536991
H 2.803851 -4.357095 -0.443221
H 1.853766 -3.274664 -1.499390
H 3.056048 -2.545250 1.242756
H 1.324585 -2.780331 0.863089
H 1.135241 1.726485 2.031788
H 3.144339 2.514840 0.506956
H 3.522711 3.973699 -1.466182
H 1.731228 4.237809 -3.183542
H -0.441493 3.057331 -2.883360
H -0.826368 1.628951 -0.924508
----

9. References

[1] a) A. Krasovskiy, P. Knochel, *Synthesis* **2006**, *2006*, 890-891; b) A. F. Burchat, J. M. Chong, N. Nielsen, *J. Organomet. Chem.* **1997**, *542*, 281-283.

[2] *Pure Appl. Chem.* **1974**, *37*, 445-462.

[3] W. C. Still, M. Kahn, A. Mitra, *J. Org. Chem.* **1978**, *43*, 2923-2925.

[4] a) G. R. Fulmer, A. J. M. Miller, N. H. Sherden, H. E. Gottlieb, A. Nudelman, B. M. Stoltz, J. E. Bercaw, K. I. Goldberg, *Organometallics* **2010**, *29*, 2176-2179; b) H. E. Gottlieb, V. Kotlyar, A. Nudelman, *J. Org. Chem.* **1997**, *62*, 7512-7515; c) R. K. Harris, E. D. Becker, S. M. Cabral de Menezes, P. Granger, R. E. Hoffman, K. W. Zilm, *Pure Appl. Chem.* **2008**, *80*, 59-84; d) R. K. Harris, E. D. Becker, S. M. Cabral de Menezes, R. Goodfellow, P. Granger, *Pure Appl. Chem.* **2001**, *73*, 1795-1818.

[5] a) *Pure Appl. Chem.* **1976**, *45*, 217-220; b) *Pure Appl. Chem.* **1972**, *29*, 625-628; c) R. K. Harris, J. Kowalewski, S. M. Cabral de Menezes, *Pure Appl. Chem.* **1997**, *69*, 2489-2496.

[6] a) T. R. Hoye, H. Zhao, *J. Org. Chem.* **2002**, *67*, 4014-4016; b) T. R. Hoye, P. R. Hanson, J. R. Vyvyan, *J. Org. Chem.* **1994**, *59*, 4096-4103.

[7] a) K. K. Murray, R. K. Boyd, M. N. Eberlin, G. J. Langley, L. Li, Y. Naito, *Pure Appl. Chem.* **2013**, *85*, 1515-1609; b) J. F. J. Todd, *Pure Appl. Chem.* **1991**, *63*, 1541-1566.

[8] a) H. A. Favre, W. H. Powell, *Nomenclature of Organic Chemistry: IUPAC Recommendations and Preferred Names 2013*, Royal Society of Chemistry, Cambridge, UK, **2014**; b) M. A. Brimble, D. S. Black, R. Hartshorn, A. P. Rauter, C.-K. Sha, L. K. Sydnes, *Pure Appl. Chem.* **2013**, *85*, 307-313; c) W. Mormann, K.-H. Hellwich, *Pure Appl. Chem.* **2008**, *80*, 201-232; d) J. Brecher, *Pure Appl. Chem.* **2008**, *80*, 277-410; e) J. Brecher, *Pure Appl. Chem.* **2006**, *78*, 1897-1970; f) G. P. Moss, *Pure Appl. Chem.* **1999**, *71*, 531-558; g) H. A. Favre, K.-H. Hellwich, G. P. Moss, W. H. Powell, J. G. Trynham, *Pure Appl. Chem.* **1999**, *71*, 1327-1330; h) G. P. Moss, *Pure Appl. Chem.* **1998**, *70*, 143-216; i) G. P. Moss, *Pure Appl. Chem.* **1996**, *68*, 2193-2222; j) W. H. Powell, *Pure Appl. Chem.* **1993**, *65*, 1357-1455; k) W. Drenth, *Pure Appl. Chem.* **1992**, *64*, 989-990; l) G. P. Moss, *Pure Appl. Chem.* **1989**, *61*, 1783-1822; m) J. S. Littler, *Pure Appl. Chem.* **1989**, *61*, 57-81; n) R. A. Y. Jones, J. F. Bunnett, *Pure Appl. Chem.* **1989**, *61*, 725-768; o) R. D. Guthrie, *Pure Appl. Chem.* **1989**, *61*, 23-56; p) W. H. Powell, *Pure Appl. Chem.* **1988**, *60*, 1395-1401; q) J. F. Bunnett, R. A. Y. Jones, *Pure Appl. Chem.* **1988**, *60*, 1115-1116; r) G. P. Moss, *Pure Appl. Chem.* **1987**, *59*, 779-832; s) N. Lozac'h, *Pure Appl. Chem.* **1986**, *58*, 1693-1696; t) W. H. Powell, *Pure Appl. Chem.* **1984**, *56*, 769-778; u) *Pure Appl. Chem.* **1984**, *56*, 595-624; v) W. H. Powell, *Pure Appl. Chem.* **1983**, *55*, 409-416; w) J. E. Merritt, K. L. Loening, *Pure Appl. Chem.* **1983**, *51*, 2251-2304; x) N. Lozac'h, *Pure Appl. Chem.* **1983**, *55*, 1463-1466; y) W. H. Powell, *Pure Appl. Chem.* **1982**, *54*, 217-228; z) *Pure Appl. Chem.* **1982**, *54*, 211-216; aa) *Pure Appl. Chem.* **1982**, *54*, 207-210; ab) J. F. Bunnett, *Pure Appl. Chem.* **1981**, *53*, 305-322; ac) *Pure Appl. Chem.* **1981**, *53*, 1901-1905; ad) *Pure Appl. Chem.* **1979**, *51*, 1995-2003; ae) *Pure Appl. Chem.* **1979**, *51*, 353-380; af) L. C. Cross, W. Klyne, *Pure Appl. Chem.* **1976**, *45*, 11-30; ag) *Pure Appl. Chem.* **1974**, *40*, 315-332; ah) *Pure Appl. Chem.* **1972**, *31*, 283-322; ai) *Pure Appl. Chem.* **1972**, *31*, 639-646; aj) *Pure Appl. Chem.* **1971**, *28*, 1-110; ak) *Pure Appl. Chem.* **1965**, *11*, 1-260.

[9] L. C. Morrill, L. A. Ledingham, J.-P. Couturier, J. Bickel, A. D. Harper, C. Fallan, A. D. Smith, *Org. Biomol. Chem.* **2014**, *12*, 624-636.

[10] D. G. Stark, L. C. Morrill, D. B. Cordes, A. M. Z. Slawin, T. J. C. O'Riordan, A. D. Smith, *Chem. Asian J.* **2016**, *11*, 395-400.

[11] J. A. King, F. H. McMillan, *J. Am. Chem. Soc.* **1951**, *73*, 4911-4915.

[12] A. A. M. Roof, H. F. van Woerden, H. Cerfontain, *Tetrahedron* **1976**, *32*, 2967-2971.

[13] C. Rüchardt, O. Krätz, S. Eichler, *Chem. Ber.* **1969**, *102*, 3922-3946.

[14] Y. Abdelhamid, K. Kasten, J. Dunne, W. C. Hartley, C. M. Young, D. B. Cordes, A. M. Z. Slawin, S. Ng, A. D. Smith, *Org. Lett.* **2022**, *24*, 5444-5449.

[15] D.-J. Barrios Antúnez, M. D. Greenhalgh, A. C. Brueckner, D. M. Walden, P. Elías-Rodríguez, P. Roberts, B. G. Young, T. H. West, A. M. Z. Slawin, P. Ha-Yeon Cheong, A. D. Smith, *Chem. Sci.* **2019**, *10*, 6162-6173.

[16] H. Sharghi, J. Aboonajmi, M. Mozaffari, M. M. Doroodmand, M. Aberi, *Appl. Organomet. Chem.* **2018**, *32*, e4124.

[17] Y.-Y. Huang, H.-C. Lin, K.-M. Cheng, W.-N. Su, K.-C. Sung, T.-P. Lin, J.-J. Huang, S.-K. Lin, F. F. Wong, *Tetrahedron* **2009**, *65*, 9592-9597.

[18] A. L. Nivorozhkin, M. S. Korobov, L. E. Konstantinovskii, L. E. Nivorozhkin, V. I. Minkin, *J. Gen. Chem.* **1985**, *55*, 757-768.

[19] J. P. Phelan, J. A. Ellman, *Adv. Synth. Catal.* **2016**, *358*, 1713-1718.

[20] S. Fuse, M. Inaba, S. Sato, M. Joshi, H. Nakamura, *Synthesis* **2018**, *50*, 1493-1498.

[21] a) A. Kimata, H. Nakagawa, R. Ohyama, T. Fukuuchi, S. Ohta, T. Suzuki, N. Miyata, *J. Med. Chem.* **2007**, *50*, 5053-5056; b) S. Zhou, J. Ren, M. Liu, L. Ren, Y. Liu, P. Gong, *Bioorg. Chem.* **2014**, *57*, 30-42.

[22] F. Marchetti, C. Pettinari, A. Cingolani, L. Brocanelli, M. Rossi, F. Caruso, *J. Organomet. Chem.* **1999**, *580*, 344-353.

[23] S. Mahajan, P. Chauhan, U. Kaya, K. Deckers, K. Rissanen, D. Enders, *Chem. Commun.* **2017**, *53*, 6633-6636.

[24] P. Chauhan, S. Mahajan, U. Kaya, A. Peuronen, K. Rissanen, D. Enders, *J. Org. Chem.* **2017**, *82*, 7050-7058.

[25] R.-H. Wang, Y.-L. Li, H.-J. He, Y.-C. Xiao, F.-E. Chen, *Chem. Eur. J.* **2021**, *27*, 4302-4306.

[26] a) *CrystalClear-SM Expert*, v2.1.: Rigaku Americas, *The Woodlands*, *Texas*, *USA*, and Rigaku Corporation, *Tokyo*, *Japan*, **2015**; b) *CrysAlisPro*, v1.171.42.49, v1.171.42.82a, v1.171.42.93a: Rigaku Oxford Diffraction, Rigaku Corporation, *Oxford*, *U.K.*, **2020-2023**.

[27] G. Sheldrick, *Acta Crystallogr. A* **2015**, *71*, 3-8.

[28] G. Sheldrick, *Acta Crystallogr. C* **2015**, *71*, 3-8.

[29] O. V. Dolomanov, L. J. Bourhis, R. J. Gildea, J. A. K. Howard, H. Puschmann, *J. Appl. Crystallogr.* **2009**, *42*, 339-341.

[30] Y. Zhao, D. G. Truhlar, *Theor. Chem. Acc.* **2008**, *120*, 215-241.

[31] a) A. Schäfer, H. Horn, R. Ahlrichs, *J. Chem. Phys.* **1992**, *97*, 2571-2577; b) A. Schäfer, C. Huber, R. Ahlrichs, *J. Chem. Phys.* **1994**, *100*, 5829-5835; c) F. Weigend, *Phys. Chem. Chem. Phys.* **2006**, *8*, 1057-1065; d) F. Weigend, R. Ahlrichs, *Phys. Chem. Chem. Phys.* **2005**, *7*, 3297-3305.

[32] A. V. Marenich, C. J. Cramer, D. G. Truhlar, *J. Phys. Chem. B* **2009**, *113*, 6378-6396.

[33] G. Luchini, J. Alegre-Requena, I. Funes-Ardoiz, R. Paton, *F1000Research* **2020**, *9*.

[34] R. F. Ribeiro, A. V. Marenich, C. J. Cramer, D. G. Truhlar, *J. Phys. Chem. B* **2011**, *115*, 14556-14562.

[35] R. L. Martin, P. J. Hay, L. R. Pratt, *J. Phys. Chem. A* **1998**, *102*, 3565-3573.

[36] M. J. Frisch, G. W. Trucks, H. B. Schlegel, G. E. Scuseria, M. A. Robb, J. R. Cheeseman, G. Scalmani, V. Barone, G. A. Petersson, H. Nakatsuji, X. Li, M. Caricato, A. V. Marenich, J. Bloino, B. G. Janesko, R. Gomperts, B. Mennucci, H. P. Hratchian, J. V. Ortiz, A. F. Izmaylov, J. L. Sonnenberg, Williams, F. Ding, F. Lipparini, F. Egidi, J. Goings, B. Peng, A. Petrone, T. Henderson, D. Ranasinghe, V. G. Zakrzewski, J. Gao, N. Rega, G. Zheng, W. Liang, M. Hada, M. Ehara, K. Toyota, R. Fukuda, J. Hasegawa, M. Ishida, T. Nakajima, Y. Honda, O. Kitao, H. Nakai, T. Vreven, K. Throssell, J. A. Montgomery Jr., J. E. Peralta, F. Ogliaro, M. J. Bearpark, J. J. Heyd, E. N. Brothers, K. N. Kudin, V. N. Staroverov, T. A. Keith, R. Kobayashi, J. Normand, K. Raghavachari, A. P. Rendell, J. C. Burant, S. S. Iyengar, J. Tomasi, M. Cossi, J. M. Millam, M. Klene, C. Adamo, R. Cammi, J. W. Ochterski, R. L. Martin, K. Morokuma, O. Farkas, J. B. Foresman, D. J. Fox *Gaussian 16 Rev. C.01*: Wallingford, CT, **2016**.

[37] C. Y. Legault *CYLview20*, Build 0001; Université de Sherbrooke: Sherbrooke, Canada, **2020**.

[38] R. A. Boto, F. Peccati, R. Laplaza, C. Quan, A. Carbone, J.-P. Piquemal, Y. Maday, J. Contreras-García, *J. Chem. Theory Comput.* **2020**, *16*, 4150-4158.

[39] a) C. Wang, S.-J. Li, Q.-C. Zhang, D. Wei, L. Ding, *Catal. Sci. Technol.* **2020**, *10*, 3664-3669; b) J. Wu, C. M. Young, A. A. Watts, A. M. Z. Slawin, G. R. Boyce, M. Bühl, A. D. Smith, *Org. Lett.* **2022**, *24*, 4040-4045.
[40] F. M. Bickelhaupt, K. N. Houk, *Angew. Chem. Int. Ed.* **2017**, *56*, 10070-10086.
[41] S. Grimme, *Chem. Eur. J.* **2012**, *18*, 9955-9964.
